# A Chiral Pentafluorinated Isopropyl Group via Iodine(I)/(III) Catalysis

**DOI:** 10.1002/anie.202015946

**Published:** 2021-02-09

**Authors:** Stephanie Meyer, Joel Häfliger, Michael Schäfer, John J. Molloy, Constantin G. Daniliuc, Ryan Gilmour

**Affiliations:** ^1^ Organisch Chemisches Institut Westfälische Wilhelms-Universität Münster Corrensstraße 36 48149 Münster Germany

**Keywords:** agrochemistry, bioisostere, conformation, fluorine, organocatalysis

## Abstract

An I(I)/(III) catalysis strategy to construct an enantioenriched fluorinated isostere of the ^*i*^Pr group is reported. The difluorination of readily accessible α‐CF_3_‐styrenes is enabled by the in situ generation of a chiral ArIF_2_ species to forge a stereocentre with the substituents F, CH_2_F and CF_3_ (up to 95 %, >20:1 *vicinal:geminal* difluorination). The replacement of the metabolically labile benzylic proton results in a highly preorganised scaffold as was determined by X‐ray crystallography (π→σ* and stereoelectronic *gauche* σ→σ* interactions). A process of catalyst editing is disclosed in which preliminary validation of enantioselectivity is placed on a structural foundation.

## Graphical abstract

The iodine(I)/(III) catalysed construction of an enantioenriched fluorinated isostere of the ^*i*^Pr group is reported. The difluorination of readily accessible α‐CF_3_‐styrenes is enabled by the in situ generation of a chiral ArIF_2_ species to forge a stereocentre with the substituents F, CH_2_F and CF_3_ (up to 95 %, >20:1 *vic:gem* difluorination). Catalyst editing for preliminary validation of enantioselectivity is placed on a structural foundation.

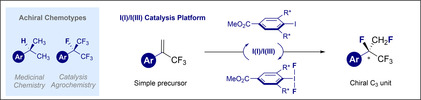

Short, unfunctionalised aliphatic groups (C_1_–C_4_) are ubiquitous structural features in the natural product repertoire, and are particularly conspicuous in polyketides and terpenes.^[1]^ This is a logical consequence of iterative biosynthesis algorithms that process low molecular weight fragments into higher homologues.^[2]^ Introduced under the auspices of acetyl‐ or propionyl‐CoA,^[3]^ complemented by electrophilic paradigms involving methyltransferases (SAM),^[4]^ these motifs appear to be vestigial in nature. However, they often encode for a highly specific function and thus delineating their biosynthetic origins has been intensively pursued. Indeed the value of harnessing small aliphatic groups to enhance the physicochemical profiles of drug candidates is exemplified by the “*magic methyl effect*”.^[5]^ Interrogating the stereochemical course of enzymatic methylation has a venerable history, due to the achiral nature of this motif and the pre‐conditions associated with designing a chiral bioisostere to track the possible translation of stereochemical information.[^6^, ^7^] Arigoni's celebrated synthesis of chiral acetic acid remains a *tour de force* in stereocontrolled synthesis, and a master class in orbital symmetry to craft an isotopically orthogonal motif (^1^H, ^2^H and ^3^H, Figure 1, top).^[8]^ Whilst this isotope strategy remains expansive in the field of mechanistic enzymology, small fragment‐based bioisosterism in drug design relies on stable isotopes to enhance the pharmaco‐kinetics and ‐dynamics of drug candidate performance.^[9]^ Molecular editing with fluorine (H→F) has proven to be particularly effective,^[10]^ and is reflected in the increasing number of fluorinated small molecules reaching the market.^[11]^ This is a consequence of fluorine's low steric demand, low polarisability and the stability of the C‐F bond. Given the success of achiral perfluoroalkyl groups in drug discovery, catalysis and agrochemistry,^[12]^ routes to small, chiral, 3D fluoroalkane motifs would be advantageous to expand the available chemical space. This includes the C_2_ (BITE group)^[13]^ which is a bioisosteric hybrid of the ethyl and trifluoromethyl groups.^[14]^ Cognisant of the prevalence of (C_3_) isopropyl units in bioactive natural product leads and small molecule pharmaceuticals (Figure 1 centre), a catalysis‐based strategy to access a differentially fluorinated analogue of the ^*i*^Pr group was initiated. Harnessing I(I)/(III) catalysis,^[15]^ it was envisaged that a formal 1,2‐addition of fluorine across the alkene moiety^[16]^ of simple α‐trifluoromethyl styrenes would generate a stereogenic centre bearing F, CH_2_F and CF_3_ groups (Figure 1, bottom).

**Figure 1:**
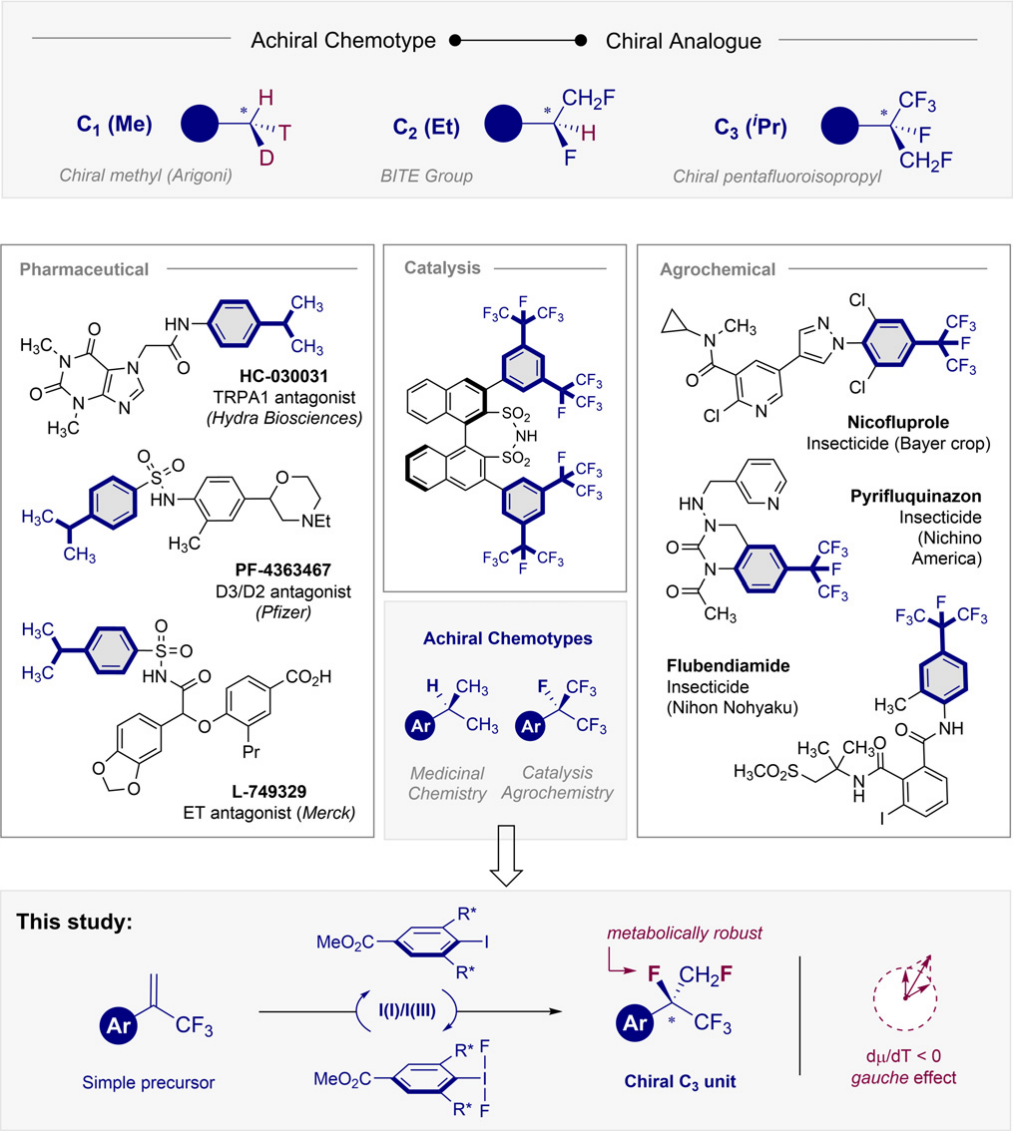
Top: Chiral bioisosteres of common aliphatic chemotypes. Centre: Selected functional small molecules containing the achiral ^*i*^Pr (pharmaceutical) and CF(CF_3_)_2_ (catalysis and agrochemical) units.^[12]^ Bottom: Design of a main group catalysis approach to generate a chiral fluorinated analogue of ^*i*^Pr.

The success of this catalysis‐based approach would be contingent on the in situ oxidation of a chiral aryl iodide organocatalyst to generate an ArIF_2_ species^[17]^ that would be sufficiently active to engage a sterically‐congested, electron‐deficient alkene. If successful, the resulting pentafluoroisopropyl surrogate would constitute a chiral C_3_ building block in which the lability of the methine proton is mitigated.^[18]^ Moreover, the constituent hyperconjugative interactions intrinsic to the internal *vicinal*‐difluoro motif^[19]^ would manifest themselves in conformation. To identify conditions that would enable the target fluorinated isopropyl motif to be generated from simple α‐trifluoromethyl styrenes, a process of reaction optimisation was conducted (Figure 2, **1 a**→**2 a**). To that end, simple aryl iodides were investigated as inexpensive catalysts in conjunction with Selectfluor^®^ as the terminal oxidant to generate the key ArI(III)F_2_ species.^[17]^ Initial studies were performed in chloroform at ambient temperature using an amine:HF ratio of 1:7.5 and the reactions were examined by ^19^F NMR spectroscopy using an internal standard. Iodobenzene proved to be a perfectly effective catalyst for this transformation to generate **(±)‐2 a** and **3 a** in a 2.5:1 ratio (86 % combined yield). The latter product arises from phenonium ion rearrangement and has been exploited in a range of catalysis‐based *geminal* difluorination processes.^[20]^ Repeating the reaction with *p*‐iodotoluene (**5**) led to a notable improvement in yield (>95 %) with comparable regioselectivity in favour of the desired *vicinal* product **2 a** (2.2:1). Electronic modulation was not well tolerated with the ester derivative **6** proving to be a less active catalyst under comparable conditions (26 %). Control experiments in the absence of catalyst led to <5 % yield and demonstrate the strongly deactivating nature of the trifluoromethyl group that inhibits background reactions such as those reported by Lal and co‐workers using HF sources and Selectfluor^®^.^[21]^

**Figure 2:**
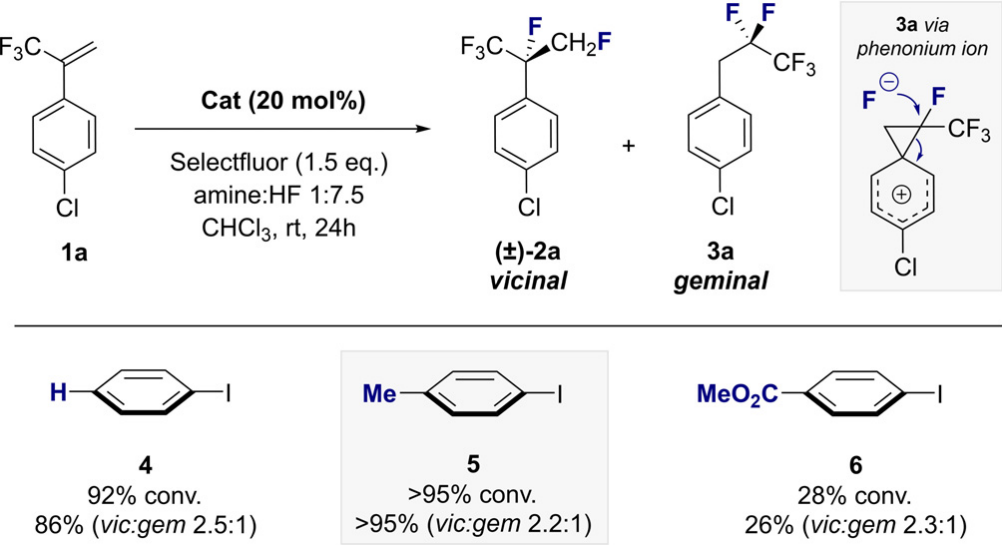
Catalyst identification. Standard reaction conditions: α‐CF_3_‐*p*‐chlorostyrene **1 a** (0.2 mmol), catalyst (20 mol %), Selectfluor® (1.5 equiv), amine:HF 1:7.5 (0.5 mL), CHCl_3_ (0.5 mL), ambient temperature, 24 h. The yield is the sum of *vicinal* and *geminal* difluorination products. The regioselectivity ratio (*vic*:*gem*) and yield were determined by ^19^F NMR spectroscopy using α,α,α‐trifluorotoluene as internal standard. Control experiment without catalyst: yield <5 %.

To explore the scope and limitations of this catalysis‐based difluorination of α‐CF_3_‐styrenes, the effect of Br*ø*nsted acidity^[22]^ was probed as a function of the amine:HF ratio.^[16b]^ This led to the identification of methods A, B and C, reflecting amine:HF ratios of 1:4.5, 1:6 and 1:7.5 respectively (Figures 3 and 4). Method A proved to be highly effective in generating the electron rich products **2 b**–**2 d** with high levels of regioselectivity favouring formation of the desired *vicinal* product (>20:1, up to 86 %). The presence of the CF_3_ group clearly distinguish this substrate class from the parent styrenes, which rearrange to generate the *geminal* product.^[20]^ Control reactions again confirmed the necessity for the catalyst. The seemingly subtle change to Method B proved to be optimal for substrates **2 e**–**2 k**, enabling the generation of alkyl derivatives (**2 e**–**2 h**, up to >20:1, *vic:gem*) as well as the electron deficient aniline derivative **2 i** (74 %, 15.5:1).

**Figure 3:**
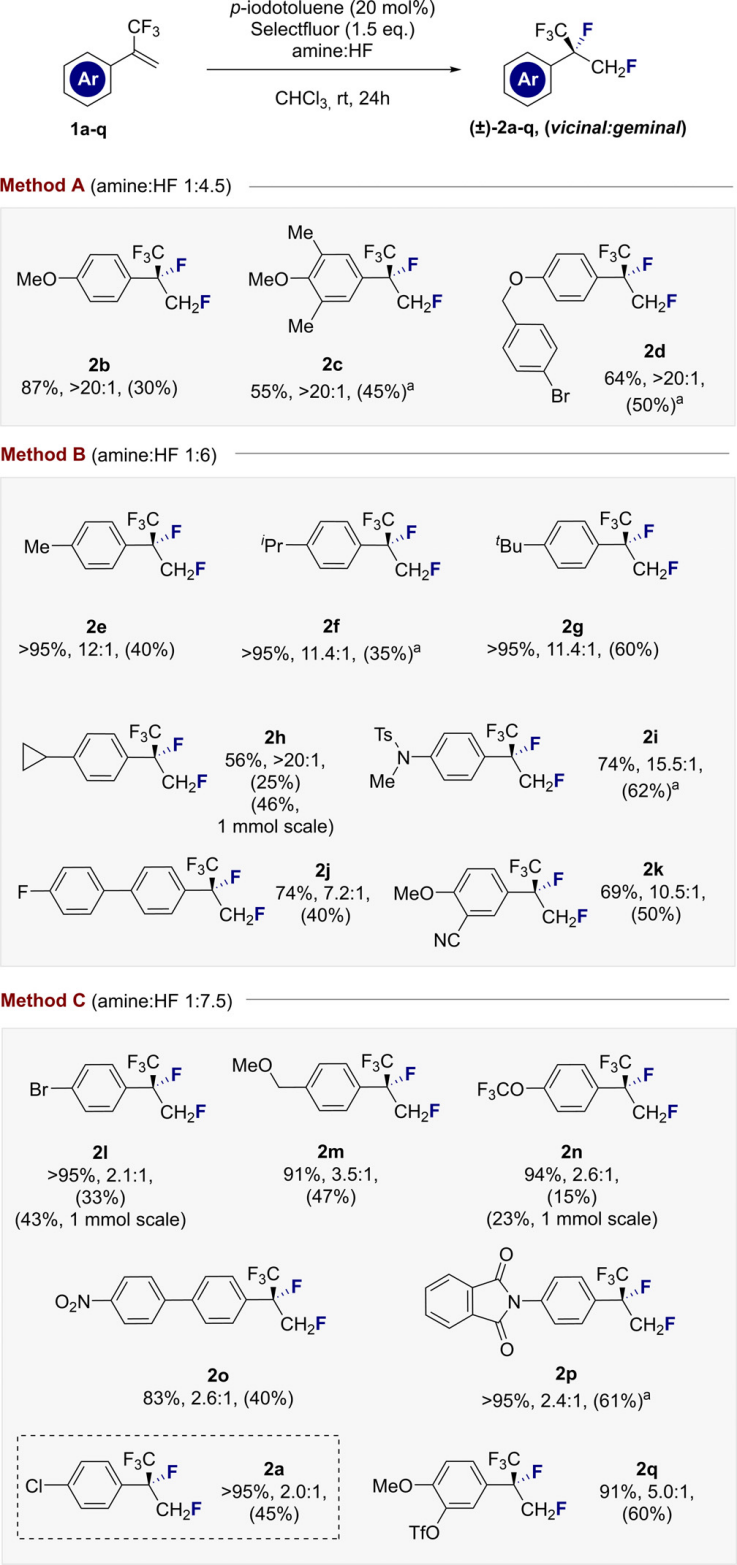
Establishing the scope of the *vicinal* difluorination of α‐CF_3_‐ styrenes to generate a highly fluorinated isopropyl group. The yield is the sum of *vicinal* and *geminal* difluorination products. The regioselectivity ratio (*vic:gem*) and yield were determined by ^19^F NMR spectroscopy using α,α,α‐trifluorotoluene as internal standard. Isolated yields of the *vicinal* products are given in parentheses. ^a^ Reaction time increased to 48 h. Arbitrary enantiomer of the product shown. N.B.: The products are often highly volatile and care must be taken in the isolation.

**Figure 4:**
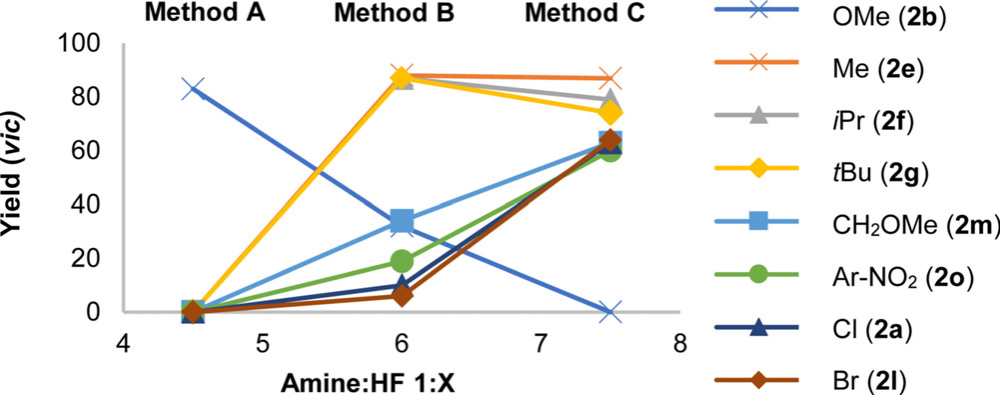
The effect of Br*ø*nsted acidity on catalysis.

Comparable efficiency and selectivity were also noted for the biphenyl system **2 j** and adduct **2 k**. Augmenting the amine:HF ratio further to Method C provided optimised conditions to access products **2 a,l**–**2 q**. Whereas employing higher HF ratios/ Br*ø*nsted acidities under the conditions developed by this laboratory tend to favour 1,1‐difluorination,^[20f]^ electron‐deficient α‐CF_3_‐styrenes proved to be notably more recalcitrant to rearrangement and the *vicinal* products predominated throughout. Given the importance of aryl bromides in contemporary medicinal chemistry, where the C(sp^2^)‐Br provides a handle for subsequent cross‐coupling, the synthesis of **2 l** was conducted on a 1 mmol scale. Despite the volatility of the product, the *vicinal* product could be isolated in 43 % yield. Products **2 m**, **2 n** and **2 o** behaved similarly and were generated in a *vicinal:geminal* ratio of ca. 3:1. Given the prominence of aniline fragments bearing isopropyl units in drug and agrochemical discovery (See Figure 1), the phthalimide **2 p** was generated cleanly in 61 % yield. Finally, access to the disubstituted aryl **2 q** was realised, this time with an improvement in regioselectivity (5.0:1). Having established conditions to enable the *vicinal* difluorination of α‐CF_3_‐styrenes via I(I)/(III) catalysis, attention was focussed on a preliminary validation of an enantioselective variant. Whilst catalyst *p*‐iodotoluene **5** is a highly competent catalyst, sites to append stereodirecting groups are conspicuously absent. The investigation was therefore repeated with resorcinol derivatives **7**–**9** in which proximal stereocentres might induce enantioinduction. Whereas catalysts **7** and **8** proved to be moderately effective, balancing the electronic effects of the resorcinol with a *p*‐CO_2_Me in catalyst **9** led to notably superior catalysis (87 % yield, *vicinal:geminal* 3:1). As the logical next step, C_2_‐symmetric resorcinol derivatives were investigated as summarised in Figure 5.[^23^, ^24^] Reactions were performed under standard conditions with an amine:HF ratio of 1:7.5 in CHCl_3_ at ambient temperature. Initially, the effect of modifying the substituent X was assessed using the methyl esters **10**–**13**. Counterintuitively, augmenting the steric footprint at site X had a detrimental effect on selectivity. Catalyst **10** (X=Me) proved to be most effective, generating compound **2 a** with 86:14 *e.r*. (>95 % conversion, 88 % combined yield). Structural editing at site Y was not tolerated as exemplified by catalysts **14**–**16**. As a control series, the C_1_‐symmetric catalysts **17**–**19** were examined (Figure 5, lower). Direct comparison of **17** with the most promising scaffold **10** confirmed the importance of C_2_ symmetry (72:28 versus 86:14 *e.r*.). Interestingly, substituting the methyl ester for benzyl (catalyst **18**) did not erode selectivity, although efficiency was decreased. Moreover, the α‐Bn catalyst (**19**) proved to be less efficient than the C_2_‐symmetric derivative **12**. Having identified catalyst **10** as the most promising scaffold to validate an enantioselective process (please see the ESI for additional details) a representative selection of α‐CF_3_‐styrenes were subjected to the general catalysis conditions using **10** (Figure 6).

**Figure 5:**
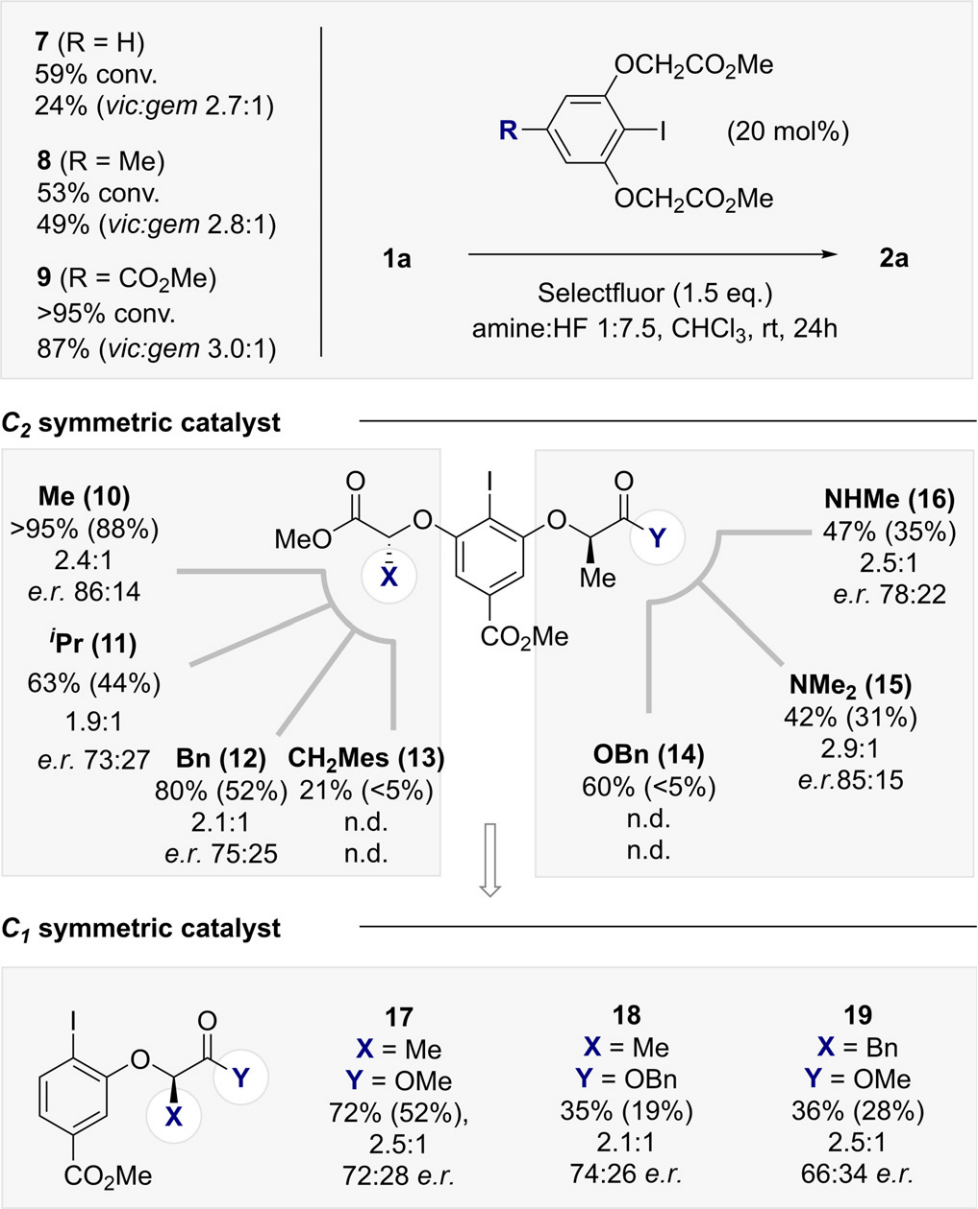
Catalyst optimisation to enable preliminary validation of enantioselection. The conversion and combined yield (in parentheses) was determined by ^19^F NMR spectroscopy of the crude reaction mixture using α,α,α‐trifluorotoluene as internal standard. Enantioselectivity determined by chiral HPLC. Standard reaction conditions: α‐CF_3_‐*p*‐chlorostyrene **1 a** (0.2 mmol), catalyst (20 mol %), Selectfluor^®^ (1.5 equiv), amine:HF 1:7.5 (0.5 mL), CHCl_3_ (0.5 mL), ambient temperature, 24 h.

**Figure 6:**
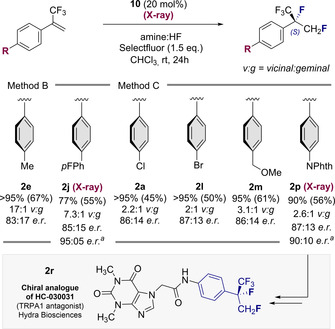
The yield is the sum of *vicinal* and *geminal* difluorination products. The regioselectivity ratio (*vic:gem*) and yield were determined by ^19^F NMR spectroscopy using α,α,α‐trifluorotoluene as internal standard. Isolated yield of the *vicinal* product is given in parentheses. Enantioselectivity determined by chiral HPLC. ^a^ After recrystallisation. N.B.: The products are often highly volatile and care must be taken in the isolation.

Gratifyingly, the methyl derivative underwent smooth difluorination to generate **2 e** (>95 %, 17.1 *vicinal:geminal*, 83:17 *e.r*.). The fluorinated biaryl system **2 j** was compatible with the conditions and could be prepared with a regioselectivity of 7:1 *vicinal:geminal* and 85:15 *e.r* (95:05 *e.r*. after recrystallisation). The *p*‐Cl and *p*‐Br derivatives **2 a** and **2 l** were prepared with 86:14 and 87:13 *e.r*., respectively, **2 m** in 86:14 *e.r*. and the protected amine **2 p** in 87:13 *e.r*. (90:10 *e.r*. after recrystallisation). Gratifyingly, compounds **2 j** and **2 p** were crystalline allowing the (*S*)‐configuration of the new stereocentre to be assigned (vide infra).^[25]^ Finally, the phthalimide derivative **2 p** was processed to an analogue of the TRPA1 antagonist HC‐030031 **2 r** in a short synthetic sequence (Figure 6, lower. Full details in the ESI).

To complement the plenum of methods available to construct short, unfunctionalised aliphatic groups for drug discovery, a catalysis‐based strategy to access chiral, fluorinated surrogates of the isopropyl group has been developed. This serves to expands the current portfolio of fluorine drug modules for drug discovery (Figure 7, centre).^[26]^ Despite the intrinsic steric and electronic challenges associated with generating highly fluorinated stereocentres, this I(I)/I(III) catalysis platform enables α‐CF_3_‐styrenes to undergo smooth *vicinal* difluorination (up to >20:1 *vicinal:geminal*). Importantly, the CF_3_ group effectively inhibits the dominant phenonium ion rearrangement associated with electron rich styrenes, allowing products such as **2 b** to be generated with excellent levels of regiocontrol (>20:1). Finally, preliminary validation of an enantioselective variant is disclosed. Whilst the sterically demanding phenyl and trifluoromethyl substituents (V_vdW_ (CF_3_)=39.8 Å^3^)[^10e^, ^27^] render this intermolecular process challenging, it is gratifying to observe encouraging levels of enantioselectivity. A tentative induction model is proposed in which facial discrimination in the enantiodetermining fluorination is a precondition of selectivity. Since X‐ray analyses of **2 j** and **2 p** confirm that the major enantiomer is (*S*)‐configured (Figure 7), it is conceivable that stabilising electrostatic interactions (RCF_2_^δ−^F⋅⋅⋅^δ+^H‐CH_2_R),^[28]^ may bias catalyst‐substrate preorganisation.^[29]^ Simple steric discrimination (CF_3_ vs. Ph) is not consistent with the selectivities observed the C_1_‐symmetric catalysts. The solid‐state analysis also reveals a stereoelectronic *gauche* effect (σ→σ*; φ_FCCF_=69.9° and 51° for **2 j** and **2 p**, respectively) and that the CF_3_ group is orthogonal to the plane of the π system (π→σ*).^[30]^ Exploring the physicochemical profile of this new motif in the context of drug discovery and contemporary agrochemistry is the focus of ongoing studies and will be reported in due course.

**Figure 7:**
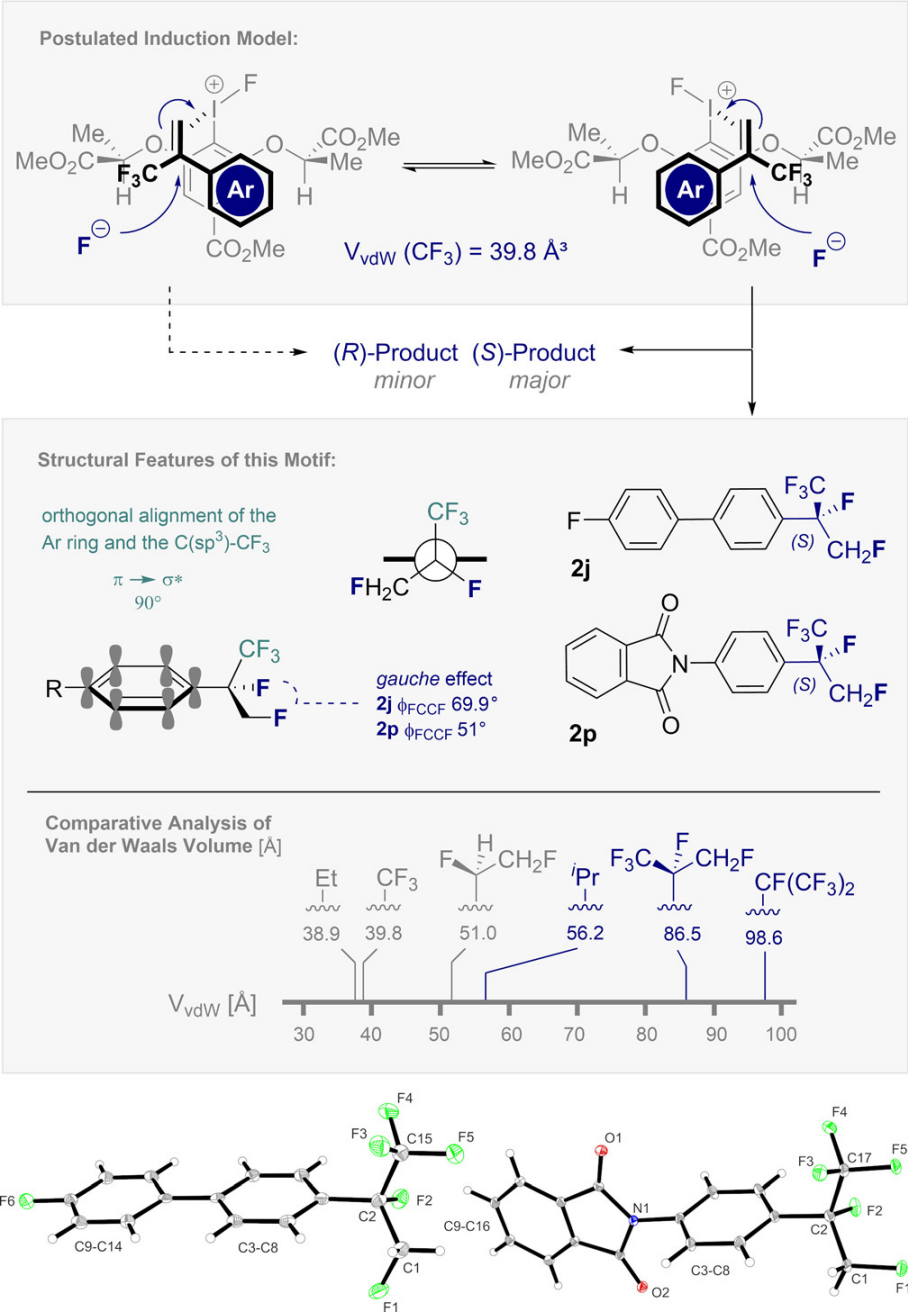
Postulated induction model and the X‐ray structure of compound **2 j** and **2 p**. Thermal ellipsoids are shown at 15 % probability. CCDC 2044630 (**2 j**) and 2044631 (**2 p**).

## Conflict of interest

The authors declare no conflict of interest.

## Supporting information

As a service to our authors and readers, this journal provides supporting information supplied by the authors. Such materials are peer reviewed and may be re‐organized for online delivery, but are not copy‐edited or typeset. Technical support issues arising from supporting information (other than missing files) should be addressed to the authors.

Supplementary
