# Supplementary material for: A Chiral Pentafluorinated Isopropyl Group via Iodine(I)/(III) Catalysis
Source: Angew Chem Int Ed Engl. 2021 Feb 9;60(12):6430–4. doi: 10.1002/anie.202015946 (PMC7986799; doi:10.1002/anie.202015946)
Supplement: Supplementary file 1 — Supplementary [file ANIE-60-6430-s001.pdf]

Angewandte Chemie
Eine Zeitschrift der Gesellschaft Deutscher Chemiker

Supporting Information

A Chiral Pentafluorinated Isopropyl Group via Iodine(I)/(III) Catalysis

*Stephanie Meyer, Joel Häfliger, Michael Schäfer, John J. Molloy, Constantin G. Daniliuc, and Ryan Gilmour**

anie_202015946_sm_miscellaneous_information.pdf

Table of Contents

Table of Contents 2
1. General Information 3
2. Experimental Section 4
2.1. Synthesis of Starting Materials 4
2.2. Synthesis of Catalysts 16
2.3. Hammett Correlation Study 26
2.4. Synthesis of Racemic Pentafluoroisopropyl Surrogates 27
2.5. Optimisation of the Enantioselective Reaction 46
2.6. Synthesis of Chiral Pentafluoroisopropyl Surrogates 47
2.7. Synthesis of the Chiral Analogue of a TRPA1 Antagonist 55
3. X-ray Crystallographic Analysis 57
4. NMR-Spectra of Key Compounds 61
5. References 129

1. General Information

All commercially available reagents were purchased as reagent grade from *Sigma Aldrich, Merck*, *Alfa Aesar*, *TCI, Fluorochem* or *abcr* and were used without further purification unless otherwise stated. Where indicated tetrahydrofuran and dichloromethane were dried by a solvent purification system including columns packed with molecular sieves and aluminium oxide. All reactions with HF were run in Teflon® vials. Solvents for extractions or chromatographic purifications were bought as technical grade and distilled on a rotary evaporator prior to use. For analytical thin layer chromatography, glass plates coated with SiO_2-60 F254 were used from *Merck*. They were visualised with UV-light (254 nm) or with $KMnO_4$ or CAM solution. Column chromatography was performed using silica gel (40-63 µm, *VWR Chemicals*). For preparative thin layer chromatography, glass plates coated with SiO_2-60 F254 and 2 mm thickness were used from *Merck.* The obtained products are often volatile and care must be taken in the isolation. The NMR measurements were performed on a *Bruker AV300, AV400, Agilent DD2 500* or an *Agilent DD2 600* by the NMR service department of the Organisch-Chemisches Institut, Westfälische Wilhelms-Universität Münster. The chemical shifts were referenced to the residual solvent peak as the internal standard (7.26 ppm for $CDCl_3$, 2.50 ppm for DMSO-d_6 for ^{1}H-NMR and 77.16 ppm for $CDCl_3$ for ^{13}C-NMR). The multiplicity is abbreviated as follows: s (singlet), d (doublet), t (triplet), q (quartet), p (pentet), h (heptet), m (multiplet) and br (broad). The given assignments are supported by additional 1D and 2D NMR experiments. The melting points were determined on a *Büchi* B-545 melting point apparatus with open glass capillaries. The IR measurements were performed on a *Perkin-Elmer 100 FT-IR* spectrometer and the intensities of the bands are assigned as follows: w (weak), m (medium), s (strong). The optical rotations were measured on a *JASCO P2000* polarimeter. High resolution mass spectrometry was performed by the MS service of the Organisch-Chemisches Institut, Westfälische Wilhelms-Universität Münster on a Bruker Daltonics MicroTof (HRMS-ESI), a Triplequad TSQ 7000 (MS-EI), Triplequad Quattro Micro GC (GC-EI-MS), a Qp5050 Single Quad (GC-EI-MS) or a LTQ Orbitap LTQ XL (HRMS-APCI). Enantiomeric ratios were determined on an *Agilent* Infinity 1260 HPLC system using a diode array detector (DAD). The chiral stationary phases were OJ, AS-H, OM and AM. The eluent system (*n*-hexane and *i*-propanol) is specified for each compound. The column temperature measured 25 to 35 °C.

HF sources

$Et_3N{\cdot}3HF$ = amine:HF / 1:3.0

Pyr·$(HF)_x$ (Olah's reagent) = amine:HF / 1:9.23 (calculated based on the physical data provided by the supplier, Sigma-Aldrich)

Generally, stock solutions of larger volumina were prepared and stored in PE containers at -20 °C:

A mixture of amine:HF / 1:4.5 was obtained by mixing 0.159 mL of Pyr·$(HF)_x$ and 0.341 mL of $Et_3N{\cdot}3HF$.

A mixture of amine:HF / 1:6.0 was obtained by mixing 0.211 mL of Pyr·$(HF)_x$ and 0.289 mL of $Et_3N{\cdot}3HF$.

A mixture of amine:HF / 1:7.5 was obtained by mixing 0.402 mL of Pyr·$(HF)_x$ and 0.098 mL of $Et_3N{\cdot}3HF$.

2. Experimental Section

2.1. Synthesis of Starting Materials

General Procedure A:

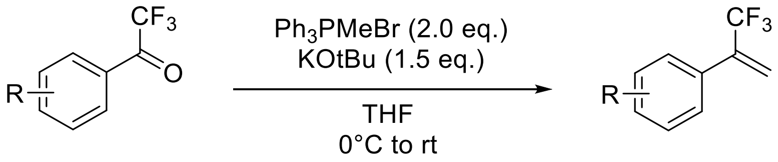

According to a procedure by this laboratory,[1] a Schlenk flask was charged with THF (0.3 M) and Ph_3PMeBr (2.0 eq.). KOtBu (1.5 eq.) was added portionwise and the resulting yellow solution was stirred for 1 h before cooling to 0 °C. The phenyl-2,2,2-trifluoroethan-1-one derivative (1.0 eq.) was added dropwise and the reaction mixture was allowed to warm to ambient temperature and was stirred overnight. EtOAc and water was added and the organics were extracted with EtOAc (3x) and washed with brine (1x). The combined organic layers were dried over $MgSO_4$, the solvent was evaporated under reduced pressure and the crude residue was purified by column chromatography (SiO_2, specified combination of solvents).

General Procedure B:

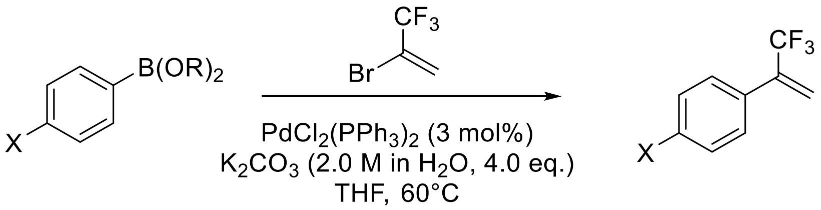

According to a procedure by *Zhou et al.*,[2] aqueous K_2CO_3 (2.0 M) was added to a pressure tube followed by THF (0.3 M). The solution was degassed followed by the addition of the boronic acid or boronic ester derivative (1.0 eq.), bis(triphenylphosphine)palladium(II) dichloride (0.03 eq.) and 2-bromo-3,3,3-trifluoro-1-propene (2.0 eq.). The resulting solution was heated to 60 °C and stirred overnight. After cooling to ambient temperature, a solution of saturated aqueous NH_4Cl was added and the organics were extracted with Et_2O (3x) and dried over Na_2SO_4. The solvent was evaporated under reduced pressure and the crude residue was purified by column chromatography (SiO_2, specified combination of solvents).

1-Chloro-4-(3,3,3-trifluoroprop-1-en-2-yl)benzene (1a)

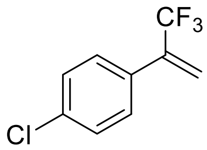

Compound **1a** was prepared according to General Procedure **A** using 1-(4-chlorophenyl)-2,2,2-trifluoroethan-1-one (3.00 g, 14.4 mmol, 1.0 eq.). The crude residue was purified by column chromatography (*n*-pentane) to yield the title compound as a colourless oil (2.20 g, 10.6 mmol, 74%).

R*f* = 0.66 (*n*-pentane).

^{1}H NMR (300 MHz, $CDCl_3$): δ [ppm] = 7.41 – 7.34 (m, 4H), 5.98 (q, *J* = 1.4 Hz, 1H), 5.77 (q, *J* = 1.7 Hz, 1H).

^{19}F{^{1}H} NMR (376 MHz, $CDCl_3$): δ [ppm] = -64.9 (s, 3F).

GC-EI-MS: (*m/z*) requires: [($C_9H_6ClF_3$)] = 206.01, (*m/z*) found: [($C_9H_6ClF_3$)] = 206.06.

Analytical data in agreement with the literature data.[3]

1-Methoxy-4-(3,3,3-trifluoroprop-1-en-2-yl)benzene (1b)

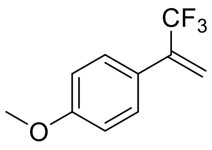

Compound **1b** was prepared according to General Procedure **A** using 2,2,2-trifluoro-1-(4-methoxyphenyl)ethan-1-one (3.06 g, 15 mmol, 1.0 eq.). The crude residue was purified by column chromatography (*n*-pentane) to yield the title compound as a colourless oil (2.01 g, 9.97 mmol, 66%).

R*f* = 0.36 (*n*-pentane).

^{1}H NMR (400 MHz, $CDCl_3$): δ [ppm] = 7.40 (m, 2H), 6.91 (m, 2H), 5.87 (q, J = 1.4 Hz, 1H), 5.70 (q, J = 1.7 Hz, 1H), 3.83 (s, 3H).

^{19}F NMR (376 MHz, $CDCl_3$): δ [ppm] = -64.9 (3F).

GC-EI-MS: (*m/z*) requires: [($C_{10}H_9F_3O$)] = 202.06, (*m/z*) found: [($C_{10}H_9F_3O$)] = 202.11.

Analytical data in agreement with the literature data.[4]

2-Methoxy-1,3-dimethyl-5-(3,3,3-trifluoroprop-1-en-2-yl)benzene (1c)

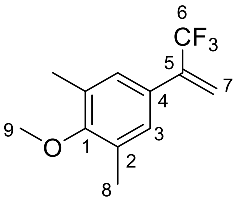

Compound **1c** was prepared according to General Procedure **B** using (4-methoxy-3,5-dimethylphenyl)boronic acid (900 mg, 5.0 mmol, 1.0 eq.). The crude residue was purified by column chromatography (10% DCM in *n*-pentane) to yield the title compound as a yellow liquid (1.08 g, 4.7 mmol, 94%).

R_f = 0.29 (10% DCM in *n*-pentane).

FT-IR ($\tilde{\nu}$ = cm^{-1}): 2938 (w), 1488 (m), 1403 (w), 1378 (w), 1357 (m), 1277 (m), 1228 (m), 1167 (m), 1144 (s), 1118 (s), 1100 (s), 1012 (m), 939 (m), 881 (m), 812 (w), 766 (w), 734 (w).

^{1}H NMR (599 MHz, $CDCl_3$): δ [ppm] = 7.11 (s, 2H, H-C3), 5.88 (q, $^4J_{HF}$ = 1.2 Hz, 1H, H^a-C7), 5.70 (q, $^4J_{HF}$ = 1.7 Hz, 1H, H^b-C7), 3.74 (s, 3H, H-C9), 2.31 (s, 6H, H-C8).

^{13}C{^{1}H} NMR (151 MHz, $CDCl_3$): δ [ppm] = 157.9 (C1), 138.8 (q, $^2J_{CF}$ = 29.9 Hz, C5), 131.2 (C2), 129.3 (C4), 128.1 (C3), 123.6 (q, $^1J_{CF}$ = 274.0 Hz, C6), 119.7 (q, $^3J_{CF}$ = 5.8 Hz, C7), 59.8 (C9), 16.3 (C8).

^{19}F{^{1}H} NMR (564 MHz, $CDCl_3$): δ [ppm] = −64.8 (s, 3F, F-C6).

GC-EI-MS: (*m/z*) requires: [($C_{12}H_{13}F_3O$)]$^+$ = 230.0913, (*m/z*) found: [($C_{12}H_{13}F_3O$)]$^+$ = 230.0910.

4-(3,3,3-Trifluoroprop-1-en-2-yl)phenol (S1)

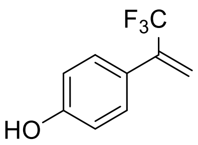

Compound **S1** was prepared according to General Procedure **B** using (4-hydroxyphenyl)boronic acid (1.38 g, 10.0 mmol, 1.0 eq.). The crude residue was purified by column chromatography (10% EtOAc in *n*-pentane) to yield the title compound as a pale yellow liquid (541 mg, 2.9 mmol, 29%).

R_f = 0.30 (10% EtOAc in *n*-pentane).

^{1}H NMR (300 MHz, $CDCl_3$): δ [ppm] = 7.42 – 7.32 (m, 2H), 6.88 – 6.81 (m, 2H), 5.87 (q, J = 1.3 Hz, 1H), 5.69 (q, J = 1.7 Hz, 1H). 4.91 (br s, 1H).

^{19}F{^{1}H} NMR (282 MHz, $CDCl_3$): δ [ppm] = -64.9 (s, 3F)

ESI-MS (negative mode): requires: [($C_9H_7F_3O$)]$^-$ = 187.0376, (*m/z*) found: [($C_9H_7F_3O$)]$^-$ = 187.0383.

Analytical data in agreement with the literature data.[5]

1-Bromo-4-((4-(3,3,3-trifluoroprop-1-en-2-yl)phenoxy)methyl)benzene (1d)

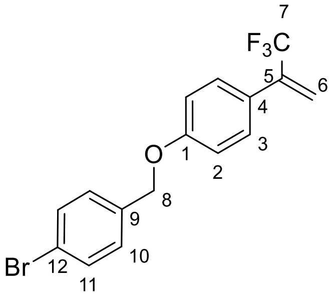

Compound **1d** was prepared according to a modified procedure from *Chai et al.*[6] 4-(3,3,3-Trifluoroprop-1-en-2-yl)phenol (**S1**) (226 mg, 1.2 mmol, 1.2 eq.) was dissolved in DMF (3 mL, 0.4 M). 4-Bromobenzyl bromide (250 mg, 1.0 mmol, 1.0 eq.) was added to the solution, followed by K_2CO_3 (207 mg, 1.5 mmol, 1.5 eq). The reaction was stirred at ambient temperature and monitored by TLC. After 14 h, EtOAc (2 mL) and water (2 mL) were added. The organics were extracted with EtOAc (3x 50 mL), washed with NH_4Cl and dried over $MgSO_4$. The solvent was evaporated under reduced pressure and the crude residue was purified by column chromatography (5% EtOAc in *n*-pentane) to yield the title compound as a white solid (339 mg, 1.0 mmol, 100%).

R_f = 0.60 (5% EtOAc in *n*-pentane).

M.p.: 69-71 °C.

FT-IR ($\tilde{\nu}$ = cm^{-1}): 2922 (w), 1515 (m), 1356 (w), 1247 (w), 1190 (m), 1113 (s), 1078 (w), 1018 (m), 949 (s), 830 (m), 812 (m).

^{1}H NMR (599 MHz, $CDCl_3$): δ [ppm] = 7.54 – 7.50 (m, 2H, H-C11), 7.40 (m, 2H, H-C3), 7.33 – 7.29 (m, 2H, H-C10), 6.98 – 6.93 (m, 2H, H-C2), 5.88 (q, $^4J_{HF}$ = 1.3 Hz, 1H, H^a-C6), 5.70 (q, $^4J_{HF}$ = 1.7 Hz, 1H, H^b-C6), 5.04 (s, 2H, H-C8).

^{13}C{^{1}H} NMR (151 MHz, $CDCl_3$): δ [ppm] = 159.2 C1), 138.4 (q, $^2J_{CF}$ = 29.9 Hz, C5), 135.8 (C12), 131.9 (C11), 129.2 (C10), 128.9 (q, $^4J_{CF}$ = 1.2 Hz, C3), 126.6 (C4), 123.6 (q, $^1J_{CF}$ = 274.1 Hz, C7), 122.2 (C9), 119.2 (q, $^3J_{CF}$ = 5.9 Hz, C6), 115.0 (C2), 69.4 (C8).

^{19}F{^{1}H} NMR (564 MHz, $CDCl_3$): δ [ppm] = -64.9 (m, 3F, F-C7).

GC-EI-MS: (*m/z*) requires: [($C_{16}H_{12}OBrF_3$)] = 356.0024, (*m/z*) found: [($C_{16}H_{12}OBrF_3$)] = 356.0020.

1-Methyl-4-(3,3,3-trifluoroprop-1-en-2-yl)benzene (1e)

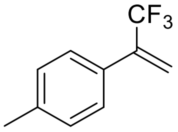

Compound **1e** was prepared according to General Procedure **A** using 2,2,2-trifluoro-1-(p-tolyl)ethan-1-one (941 mg, 5 mmol, 1.0 eq.). The crude residue was purified by column chromatography (*n*-pentane) to yield the title compound as a colourless oil (719 mg, 3.9 mmol, 77%).

R_f = 0.85 (*n*-pentane).

^{1}H NMR (400 MHz, $CDCl_3$): δ [ppm] = 7.36 (m, 2H), 7.20 (m, 2H), 5.91 (q, J = 1.4 Hz, 1H), 5.74 (q, J = 1.7 Hz, 1H), 2.38 (s, 3H).

^{19}F NMR (376 MHz, $CDCl_3$): δ [ppm] = -64.8 (3F).

GC-EI-MS: (*m/z*) requires: [($C_{10}H_9F_3$)] = 186.06509, (*m/z*) found: [($C_{10}H_9F_3$)] = 186.06563.

Analytical data in agreement with the literature data.[4]

1-Isopropyl-4-(3,3,3-trifluoroprop-1-en-2-yl)benzene (1f)

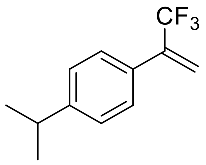

Compound **1f** was prepared according to General Procedure **A** using 2,2,2-trifluoro-1-(4-isopropylphenyl)ethan-1-one (2.45 g, 9.5 mmol, 1.0 eq.). The crude residue was purified by column chromatography (*n*-pentane) to yield the title compound as a colourless oil (545 mg, 2.5 mmol, 26%).

$\mathbf{R_f}$ = 0.72 (*n*-pentane).

^{1}H NMR (300 MHz, $CDCl_3$): δ [ppm] = 7.43 – 7.37 (m, 2H), 7.27 – 7.22 (m, 2H), 5.91 (q, *J* = 1.4 Hz, 1H), 5.75 (q, *J* = 1.7 Hz, 1H), 2.93 (h, *J* = 6.9 Hz, 1H), 1.26 (d, *J* = 6.9 Hz, 6H).

^{19}F{^{1}H} NMR (282 MHz, $CDCl_3$): δ [ppm] = -64.7 (s, 3F).

GC-EI-MS: (*m/z*) requires: [($C_{12}H_{13}F_3$)] = 214.10, (*m/z*) found: [($C_{12}H_{13}F_3$)] = 214.16.

Analytical data in agreement with the literature data.[7]

1-(*Tert*-butyl)-4-(3,3,3-trifluoroprop-1-en-2-yl)benzene (1g)

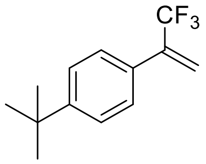

Compound **1g** was prepared according to General Procedure **A** using 1-(4-(*tert*-butyl)phenyl)-2,2,2-trifluoroethan-1-one (3.45 g, 15.0 mmol, 1.0 eq.). The crude residue was purified by column chromatography (*n*-pentane) to yield the title compound as a colourless oil (2.58 g, 11.3 mmol, 75%)

$\mathbf{R_f}$ = 0.81 (*n*-pentane).

^{1}H NMR (300 MHz, $CDCl_3$): δ [ppm] = 7.41 (m, 4H), 5.92 (q, *J* = 1.4 Hz, 1H), 5.76 (q, *J* = 1.7 Hz, 1H), 1.34 (s, 9H).

^{19}F{^{1}H} NMR (377 MHz, $CDCl_3$): δ [ppm] = -64.7 (s, 3F)

GC-EI-MS: (*m/z*) requires: [($C_{13}H_{15}F_3$)] = 228.11, (*m/z*) found: [($C_{13}H_{15}F_3$)] = 228.15.

Analytical data in agreement with the literature data.[8]

1-Cyclopropyl-4-(3,3,3-trifluoroprop-1-en-2-yl)benzene (1h)

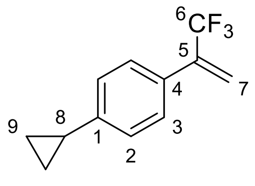

Compound **1h** was prepared according to General Procedure **B** using cyclopropylboronic acid (644 mg, 7.5 mmol, 1.0 eq.) and 1-bromo-4-(3,3,3-trifluoroprop-1-en-2-yl)benzene (1.25 g, 5 mmol). The crude residue was purified by column chromatography (*n*-pentane) to yield the title compound as colourless oil (293 mg, 1.34 mmol, 27%).

$\mathbf{R_f}$ = 0.91 (*n*-pentane).

FT-IR ($\tilde{\nu}$ = cm^{-1}): 3009 (w), 1910 (w), 1615 (w), 1519 (m), 1460 (w), 1404 (w), 1351 (w), 1281 (w), 1190 (s), 1163 (s), 1115 (s), 1079 (s), 1046 (m), 1018 (w), 939 (m), 901 (m), 852 (w), 828 (m), 748 (w), 699 (m).

^{1}H NMR (599 MHz, $CDCl_3$): δ [ppm] = 7.44 (m, 2H, H-C3), 7.15 (m, 2H, H-C2), 5.97 (m, 1H, H^a-C7), 5.84 (m, 1H, H^b-C7), 1.98 (tt, $^3J_{HH}$ = 8.50, 5.05 Hz, 1H, H-C8), 1.08 (m, 2H, H^a-C9), 0.81 (m, 2H, H^b-C9).
^{13}C NMR {^{1}H} (126 MHz, $CDCl_3$): δ [ppm] = 145.4 (C1), 139.0 (q, $^2J_{CF}$ = 29.9 Hz, C5), 130.8 (C4), 127.4 (q, $^4J_{CF}$ = 1.1 Hz, C3), 125.9 (C2), 123.7 (q, $^1J_{CF}$ = 274.0 Hz, C6), 119.5 (q, $^3J_{CF}$ = 5.8 Hz, C7), 15.3 (C8), 9.7 (C9).
^{19}F NMR (564 MHz, $CDCl_3$): δ [ppm] = - 64.7 (s, F-C6).
GC-EI-MS: (*m/z*) requires: [($C_{12}H_{11}F_3$)] = 212.08074, (*m/z*) found: [($C_{12}H_{11}F_3$)] = 212.08127.

1-Nitro-4-(3,3,3-trifluoroprop-1-en-2-yl)benzene (S2)

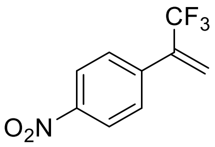

Compound **S2** was prepared according to General Procedure **B** using (4-nitrophenyl)boronic acid (1.67 g, 10.0 mmol, 1.0 eq.). The crude residue was purified by column chromatography (5-10% EtOAc in cyclohexane) to yield the title compound as a yellow liquid (1.74 g, 8.0 mmol, 80%).
R_f = 0.40 (10% EtOAc in cyclohexane).
^{1}H NMR (400 MHz, $CDCl_3$): δ [ppm] = 8.28 – 8.23 (m, 2H), 7.67 – 7.60 (m, 2H), 6.15 (q, J = 1.5 Hz, 1H), 5.93 (q, J = 1.7 Hz, 1H).
^{19}F{^{1}H} NMR (376 MHz, $CDCl_3$): δ [ppm] = -64.7 (s, 3F).
GC-EI-MS: (*m/z*) requires: [($C_9H_6NO_2F_3$)]$^+$ = 217.0345, (*m/z*) found: [($C_9H_6NO_2F_3$)]$^+$ = 217.0344.
Analytical data in agreement with the literature data.[9]

4-(3,3,3-Trifluoroprop-1-en-2-yl)aniline (S3)

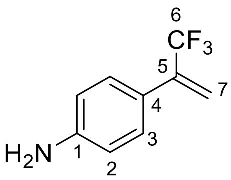

1-Nitro-4-(3,3,3-trifluoroprop-1-en-2-yl)benzene (**S2**) (1.63 g, 7.50 mmol, 1.0 eq.) was dissolved in a mixture of water (5.6 mL), concentrated HCl (0.23 mL) and MeOH (9.4 mL). Iron powder (1.67 g, 30 mmol, 4.0 eq.) was added portionwise to the vigorously stirred solution. The reaction was stirred for 7.5 h at ambient temperature. After completion (indicated by TLC), the reaction mixture was filtered and the brown solid was washed with water (3x 100 mL) and DCM (3x 100 mL) multiple times. The layers of the filtrate were separated and the aqueous layer was extracted with DCM (2x). The combined organic layers were dried over $MgSO_4$, the solvent was removed under reduced pressure and the crude product was purified by column chromatography (20% EtOAc in cyclohexane) to yield the title compound as a yellow oil (924 mg, 4.94 mmol, 66%).
R_f = 0.39 (30% EtOAc in cyclohexane)
FT-IR ($\tilde{\nu}$ = cm^{-1}): 3390 (w), 3225 (w), 3040 (w), 1892 (w), 1622 (s), 1519 (s), 1404 (w), 1354 (m), 1288 (m), 1182 (s), 1158 (s), 1109 (s), 1079 (s), 1013 (w), 855 (m), 831 (m), 732 (m).

^{1}H NMR (599 MHz, $CDCl_3$): δ [ppm] = 7.28 (d, $^{3}J_{HH}$ = 8.8 Hz, 2H, H-C2), 6.67 (d, $^{3}J_{HH}$ = 8.8 Hz, 2H, H-C3), 5.79 (q, $^{4}J_{HF}$ = 1.3 Hz, 1H, H^{a}-C7), 5.66 (q, $^{4}J_{HF}$ = 1.8 Hz, 1H, H^{b}-C7), 3.79 (br. s, 2H, NH_2).

^{13}C{^{1}H} NMR (151 MHz, $CDCl_3$): δ [ppm] = 147.3 (C1), 138.6 (q, $^{2}J_{CF}$ = 29.5 Hz, C5), 128.5 (q, $^{4}J_{CF}$ = 1.3 Hz, C3), 123.8 (q, $^{1}J_{CF}$ = 274.2 Hz, C6), 123.7 (C4), 117.6 (q, $^{3}J_{CF}$ = 5.9 Hz, C7), 114.9 (C2).

^{19}F{^{1}H} NMR (564 MHz, $CDCl_3$): δ [ppm] = −64.7 (s, 3F, F-C6).

ESI-MS: (*m/z*) requires: $[(C_9H_8NF_3H)]^+$ = 188.0682, (*m/z*) found: $[(C_9H_8NF_3H)]^+$ = 188.0680.

4-Methyl-*N*-(4-(3,3,3-trifluoroprop-1-en-2-yl)phenyl)benzenesulfonamide (S4)

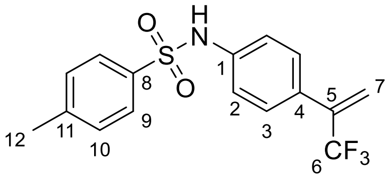

A solution of 4-(3,3,3-trifluoroprop-1-en-2-yl)aniline (**S3**) (468 mg, 2.50 mmol, 1.0 eq.) in pyridine (12.6 mL, 0.2 M) was cooled to 0 °C. TsCl (524 mg, 2.75 mmol, 1.1 eq). was added portionwise. After 1 h, the cooling bath was removed, and the mixture was stirred for additional 4 h at ambient temperature. After completion, the solvent was evaporated under reduced pressure and water was added to the residual slurry. DCM (50 mL) was added and the layers were separated. The aqueous layer was extracted with DCM (2x 50 mL) and the combined organic layers were dried over $MgSO_4$.The solvent was removed under reduced pressure and the crude product was purified by column chromatography (DCM) to yield the title compound as a yellow oil (751 mg, 2.20 mmol, 88%).

R_f = 0.44 (DCM)

FT-IR ($\tilde{\nu}$ = cm^{-1}): 3257 (m), 1611 (m), 1598 (w), 1515 (m), 1495 (m), 1457 (w), 1399 (m), 1333 (m), 1301 (m), 1230 (w), 1185 (m), 1156 (s), 1117 (s), 1091 (s), 1078 (s), 1018 (w), 915 (m), 840 (m), 812 (m), 737 (m), 704 (m), 659 (s).

^{1}H NMR (599 MHz, $CDCl_3$): δ [ppm] = 7.74 (d, $^{3}J_{HH}$ = 8.3 Hz, 2H, H-C9), 7.38 –7.34 (m, 1H, H-N), 7.32 (d, $^{3}J_{HH}$ = 8.4 Hz, 2H, H-C), 7.25 (d, $^{3}J_{HH}$ = 8.4 Hz, 2H, H-C10), 7.11 (d, $^{3}J_{HH}$ = 8.4 Hz, 2H, H-C), 5.89 (q, $^{4}J_{HF}$ = 1.2 Hz, 1H, H^{a}-C7), 5.70 (q, $^{4}J_{HF}$ = 1.8 Hz, 1H, H^{b}-C7), 2.38 (s, 3H, H-C12).

^{13}C{^{1}H} NMR (151 MHz, $CDCl_3$): δ [ppm] = 144.3 (C8), 138.1 (q, $^{2}J_{CF}$ = 30.1 Hz, C5), 137.5 (C1), 136.1 (C11), 130.2 (C4), 130.0 (C10), 128.5 (q, $^{4}J_{CF}$ = 1.2 Hz, C3), 127.4 (C9), 123.3 (q, $^{1}J_{CF}$ = 274.1 Hz, C6), 120.6 (C2), 120.2 (q, $^{3}J_{CF}$ = 5.8 Hz , C7), 21.7 (C12).

^{19}F NMR (564 MHz, $CDCl_3$): δ [ppm] = −64.8 (s, 3F, F-C6).

ESI-MS: (*m/z*) requires: $[(C_{16}H_{14}NO_2SF_3Na)]^+$ = 364.0590, (*m/z*) found: $[(C_{16}H_{14}NO_2SF_3Na)]^+$ = 364.0587.

N,4-Dimethyl-N-(4-(3,3,3-trifluoroprop-1-en-2-yl)phenyl)benzenesulfonamide (1i)

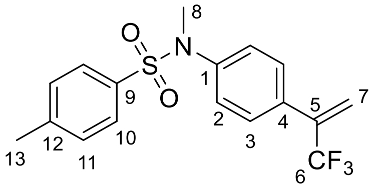

Methyl iodide (157 µL, 2.4 mmol, 1.2 eq.) was added dropwise to a stirred suspension of 4-methyl-*N*-(4-(3,3,3-trifluoroprop-1-en-2-yl)phenyl)benzenesulfonamide (**S4**) (682 mg, 2.00 mmol, 1.0 eq.) and K_2CO_3 (1.63 g, 12.00 mmol, 6.0 eq.) in dry DMF (8 mL). The reaction was stirred for 4 h at ambient temperature. Water (50 mL) was added and the mixture was extracted with DCM (3x 50 mL), dried over $MgSO_4$ and purified by column chromatography (DCM) to yield the title compound as a yellow oil (702 mg, 1.97 mmol, 98%).

R_f = 0.61 (DCM)

FT-IR ($\tilde{\nu}$ = cm^{-1}): 2975 (w), 1598 (w), 1510 (m), 1452 (w), 1404 (w), 1347 (s), 1306 (w), 1265 (w), 1189 (m), 1155 (s), 1115 (s), 1079 (s), 1061 (m), 1017 (w), 947 (w), 870 (m), 849 (m), 813 (m), 758 (w), 707 (m), 684 (s).

^{1}H NMR (599 MHz, $CDCl_3$): δ [ppm] = 7.44 (d, $^3J_{HH}$ = 8.3 Hz, 2H, H-C11), 7.40 (d, $^3J_{HH}$ = 8.3 Hz, 2H, H-C3), 7.25 (d, $^3J_{HH}$ = 8.5 Hz, 2H, H-C10), 7.14 (d, $^3J_{HH}$ = 8.6 Hz, 2H, H-C2), 5.97 (q, $^4J_{HF}$ = 1.3 Hz, 1H, H^a-C7), 5.84 (q, $^4J_{HF}$ = 1.7 Hz, 1H, H^b-C7), 3.16 (s, 3H, H-C8), 2.42 (s, 3H, H-C13).

^{13}C{^{1}H} NMR (151 MHz, $CDCl_3$): δ [ppm] = 143.9 (C12), 142,45 (C1), 138.2 (q, $^2J_{CF}$ = 30.5 Hz, C5), 133.6 (C9), 132.4 (C4), 129.6 (C10), 128.0 (C11), 128.0 (q, $^4J_{CF}$ = 1.2 Hz, C3), 126.5 (C2), 123.3 (q, $^1J_{CF}$ = 274.0 Hz, C6), 120.9 (q, $^3J_{CF}$ = 5.7 Hz, C7), 38.0 (C8), 21.7 (C13).

^{19}F NMR (376 MHz, $CDCl_3$): δ [ppm] = −64.8 (s, 3F, F-C6).

ESI-MS: (*m/z*) requires: $[(C_{17}H_{16}NO_2SF_3Na)]^+$ = 378.0746, (*m/z*) found: $[(C_{17}H_{16}NO_2SF_3Na)]^+$ = 378.0744.

4-Fluoro-4'-(3,3,3-trifluoroprop-1-en-2-yl)-1,1'-biphenyl (1j)

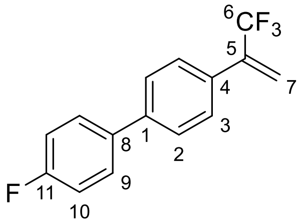

Compound **1j** was prepared according to General Procedure **B** using (4-fluorophenyl)boronic acid (1.12 g, 8 mmol, 1.0 eq.) and 1-bromo-4-(3,3,3-trifluoroprop-1-en-2-yl)benzene (1.00 g, 4 mmol). The crude residue was purified by column chromatography (1% Et_2O in *n*-pentane) to yield the title compound as a yellow solid (0.97 g, 3.62 mmol, 91%).

R_f = 0.54 (*n*-pentane).

M.p.: 52 – 54°C

FT-IR ($\tilde{\nu}$ = cm^{-1}): 3133 (w), 3045 (w), 3020 (w), 1923 (w), 1897 (w), 1668 (w), 1597 (m), 1531 (w), 1496 (m), 1441 (w), 1407 (w), 1356 (m), 1326 (m), 1247 (m), 1165 (s), 1114 (s), 1100 (s), 1081 (s), 1014 (w), 1003 (w), 969 (w), 950 (m), 856 (w), 825 (s), 754 (w), 707 (w), 683 (m).

^{1}H NMR (500 MHz, $CDCl_3$): δ [ppm] = 7.56 (m, 6H, H-C9, H-C2, H-C3), 7.17 (m, 2H, H-C10), 6.00 (q, $^4J_{FH}$ = 1.4 Hz, 1H, H^a-C7), 5.84 (q, $^4J_{FH}$ = 1.7 Hz, 1H, H^b-C7).

^{13}C NMR {^{1}H} (126 MHz, $CDCl_3$): δ [ppm] = 162.9 (d, $^{1}J_{CF}$ = 247.1 Hz, C11), 141.0 (C1), 138.7 (q, $^{2}J_{CF}$ = 30.1 Hz, C5), 136.5 (d, $^{4}J_{CF}$ = 3.3 Hz, C8), 132.6 (C4), 128.8 (d, $^{2}J_{CF}$ = 8.1 Hz, C9), 128.0 (q, $^{4}J_{CF}$ = 1.2 Hz, C3), 127.3 (C2), 123.5 (q, $^{1}J_{CF}$ = 274.0 Hz, C6), 120.4 (q, $^{3}J_{CF}$ = 5.8 Hz, C7), 115.9 (d, $^{2}J_{CF}$ = 21.4 Hz, C10).

^{19}F NMR (470 MHz, $CDCl_3$): δ [ppm] = - 64.7 (dd, $^{4}J_{FH}$ = 1.7 Hz, 1.4 Hz, 3F, F-C6), -115.0 (tt, $^{3}J_{FH}$ = 8.5, $^{4}J_{FH}$ = 5.3 Hz, 1F, F-C11).

GC-EI-MS: (*m/z*) requires: [($C_{15}H_{10}F_4$)] = 266.07131, (*m/z*) found: [($C_{15}H_{10}F_4$)] = 266.07194.

4-Methoxy-5-(3,3,3-trifluoroprop-1-en-2-yl)benzonitrile (**1k**)

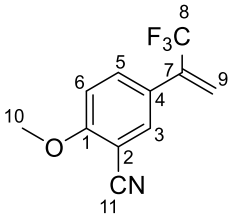

Compound **1k** was prepared according to General Procedure **B** using 3-cyano-4-methoxyphenylboronic acid pinacol ester (1.30 g, 5.0 mmol, 1.0 eq.). The crude residue was purified by column chromatography (20% EtOAc in *n*-pentane) to yield the title compound as a white solid (804 mg, 3.5 mmol, 71%).

R_f = 0.75 (20% EtOAc in *n*-pentane).

M.p.: 45 – 47°C.

FT-IR ($\tilde{\nu}$ = cm^{-1}): 2940 (w), 2849 (w), 2230 (s), 1888 (w), 1610 (s), 1508 (s), 1468 (s), 1444 (s), 1416 (s), 1394 (s), 1360 (s), 1343 (s), 1301 (s), 1283 (s), 1211 (s), 1163 (s), 1123 (s), 1107 (s), 1092 (s), 1082 (s), 1016 (s), 941 (s), 898 (s), 832 (s), 755 (s), 742 (s), 710 (s), 683 (m).

^{1}H NMR (599 MHz, $CDCl_3$): δ [ppm] = 7.65 – 7.61 (m, 2H, H-C4 and H-C5), 7.00 (d, $^{3}J_{HH}$ = 8.8 Hz, 1H, H-C6), 5.97 (q, $^{4}J_{HF}$ = 1.4 Hz, 1H, H^{a}-C9), 5.75 (q, $^{4}J_{HF}$ = 1.7 Hz, 1H, H^{b}-C9), 3.96 (s, 3H, H-C10).

^{13}C{^{1}H} NMR (151 MHz, $CDCl_3$): δ [ppm] = 161.6 (C11), 136.9 (q, $^{2}J_{CF}$ = 30.6 Hz, C7), 133.5 (q, $^{4}J_{CF}$ = 1.2 Hz, C5), 132.9 (q, $^{4}J_{CF}$ = 1.2 Hz, C3), 126.6 (C4), 123.1 (q, $^{1}J_{CF}$ = 273.9 Hz, C8), 121.0 (q, $^{3}J_{CF}$ = 5.7 Hz, C9), 115.9 (C1), 111.7 (C6), 102.4 (C2), 56.4 (C10).

^{19}F{^{1}H} NMR (564 MHz, $CDCl_3$): δ [ppm] = -65.2 (s, 3F, F-C8).

ESI-MS: (*m/z*) requires: [($C_{11}H_8NOF_3$)Na]$^{+}$ = 250.0461, (*m/z*) found: [($C_{11}H_8NOF_3$)Na]$^{+}$ = 250.0448.

1-Bromo-4-(3,3,3-trifluoroprop-1-en-2-yl)benzene (1l)

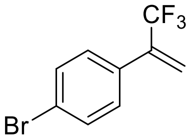

Compound **1l** was prepared according to General Procedure **A** using 4′-bromo-2,2,2-trifluoroacetophenone (6.33 g, 25.0 mmol, 1.0 eq.). The reaction was run in duplicate and the combined crude residue was purified by column chromatography (*n*-pentane) to yield the title compound as a colourless oil (10.62 g, 42.3 mmol, 85%).

R_f = 0.65 (*n*-pentane).

^{1}H NMR (300 MHz, $CDCl_3$): δ [ppm] = 7.55 – 7.49 (m, 2H), 7.32 (d, J = 8.3 Hz, 2H), 5.98 (q, J = 1.4 Hz, 1H), 5.78 (q, J = 1.7 Hz, 1H).

^{19}F{^{1}H} NMR (282 MHz, $CDCl_3$): δ [ppm] = -64.9 (s, 3F).

GC-EI-MS: (*m/z*) requires: [($C_9H_6BrF_3$)] = 249.96, (*m/z*) found: [($C_9H_6BrF_3$)] = 250.01.

Analytical data in agreement with the literature data.[10]

1-(Methoxymethyl)-4-(3,3,3-trifluoroprop-1-en-2-yl)benzene (1m)

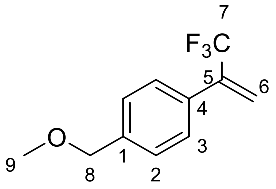

Compound **1m** was prepared according to General Procedure **B** using (4-(methoxymethyl)phenyl)boronic acid (830 mg, 5.0 mmol, 1.0 eq.). The crude residue was purified by column chromatography (4% EtOAc in *n*-pentane) to yield the title compound as a colourless oil (902 mg, 4.2 mmol, 83%).

R$_f$ = 0.42 (4% EtOAc in *n*-pentane).

FT-IR ($\tilde{\nu}$ = cm^{-1}): 2930 (m), 2826 (m), 1517 (m), 1454 (m), 1404 (s), 1383 (s), 1351 (s), 1283 (w), 1164 (s), 1116 (s), 1077 (s), 1021 (s), 945 (s), 830 (s), 733 (s).

^{1}H NMR (599 MHz, $CDCl_3$): δ [ppm] = 7.47 – 7.43 (m, 2H, H-C3), 7.38 – 7.35 (m, 2H, H-C2), 5.95 (q, $^4J_{HF}$ = 1.4 Hz, 1H, H^a-C6), 5.77 (q, $^4J_{HF}$ = 1.7 Hz, 1H, H^b-C6), 4.48 (s, 2H, H-C8), 3.41 (s, 3H, H-C9)

^{13}C{^{1}H} NMR (151 MHz, $CDCl_3$): δ [ppm] = 139.4 (C1), 138.9 (q, $^2J_{CF}$ = 30.1 Hz, C5), 133.1 (C4), 127.9 (C2), 127.6 (q, $^4J_{CF}$ = 1.2 Hz, C3), 123.5 (q, $^1J_{CF}$ = 274.0 Hz, C7), 120.4 (q, $^3J_{CF}$ = 5.8 Hz, C6), 74.3 (C8), 58.4 (C9).

^{19}F{^{1}H} NMR (564 MHz, $CDCl_3$): δ [ppm] = -64.8 (s, 3F).

GC-EI-MS: (*m/z*) requires: [($C_{11}H_{11}F_3O$)] = 216.07565, (*m/z*) found: [($C_{11}H_{11}F_3O$)] = 216.07564.

1-(Trifluoromethoxy)-4-(3,3,3-trifluoroprop-1-en-2-yl)benzene (1n)

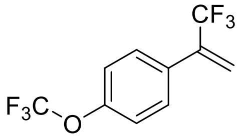

Compound **1n** was prepared according to General Procedure **B** using (4-(trifluoromethoxy)phenyl)boronic acid (824 mg, 4.0 mmol, 1.0 eq.) The crude residue was purified by column chromatography (*n*-pentane) to yield the title compound as a colourless liquid (684 mg, 2.7 mmol, 67%).

R$_f$ = 0.70 (*n*-pentane).

^{1}H NMR (300 MHz, $CDCl_3$): δ [ppm] = 7.52 – 7.45 (m, 2H), 7.27 – 7.21 (m, 2H), 6.00 (q, J = 1.4 Hz, 1H), 5.78 (q, J = 1.7 Hz, 1H).

^{19}F{^{1}H} NMR (282 MHz, $CDCl_3$): δ [ppm] = -57.85 (s, 3F), -65.05 (s, 3F).

GC-EI-MS: (*m/z*) requires: [($C_{10}H_6F_6O$)] = 256.03, (*m/z*) found: [($C_{10}H_6F_6O$)] = 256.09.

Analytical data in agreement with the literature data.[4]

4-Nitro-4'-(3,3,3-trifluoroprop-1-en-2-yl)-1,1'-biphenyl (1o)

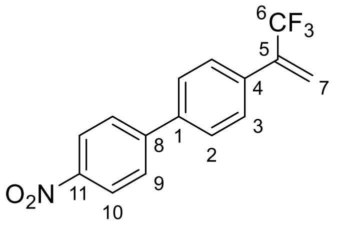

Compound **1o** was prepared according to general procedure **C** using (4-nitrophenyl)boronic acid (1.34 g, 8 mmol, 1.0 eq.) The crude residue was purified by column chromatography (10% Et_2O in *n*-pentane) to yield the title compound as a yellow solid (0.69 g, 2.35 mmol, 59%).

R*f* = 0.36 (10% EtOAc in *n*-pentane).

M.p.: 41.0 – 43.0°C

FT-IR ($\tilde{\nu}$ = cm^{-1}): 3132 (w), 2323 (w). 1923 (w), 1897 (w), 1668 (w), 1597 (m), 1531 (w), 1496 (m), 1440 (w), 1406 (w), 1355 (m), 1325 (w), 1246 (w), 1165 (s), 1114 (s), 1100 (s), 1080 (s), 1015 (w), 1003 (w), 969 (w), 950 (s), 824 (s), 753 (w), 708 (w), 683 (m).

^{1}H NMR (500 MHz, $CDCl_3$): δ[ppm] = 8.32 (m, 2H, H-C10), 7.75 (m, 2H, H-C9), 7.66 (m, 2H, H-C2), 7.60 (m, 2H, H-C3), 6.04 (q, $^4J_{FH}$ = 1.4 Hz, 1H, H^a-C7), 5.88 (q, $^4J_{FH}$ = 1.7 Hz, 1H, H^b-C7).

^{13}C NMR {^{1}H} (126 MHz, $CDCl_3$): δ [ppm] = 147.5 (C11), 146.7 (C8), 139.4 (C1), 138.4 (q, $^2J_{CF}$ = 30.3 Hz, C5), 134.3 (C4), 128.3 (q, $^4J_{CF}$ = 1.2 Hz, C3), 127.9 (C9), 127.7 (C2), 124.3 (C10), 123.4 (q, $^1J_{CF}$ = 274.0 Hz, C6), 121.2 (q, $^3J_{CF}$ = 5.8 Hz, C7).

^{19}F NMR (470 MHz, $CDCl_3$): δ [ppm] = - 64.7 (dd, $^4J_{FH}$ = 1.7 Hz, 1.3 Hz, 3F, F-C6).

HR-ESI-MS: *m/z*: 316.0559 ($[M+Na]^+$, calcd. for $C_{15}H_{10}NO_2F_3Na^+$: 316.0555).

2-(4-(3,3,3-Trifluoroprop-1-en-2-yl)phenyl)isoindoline-1,3-dione (1p)

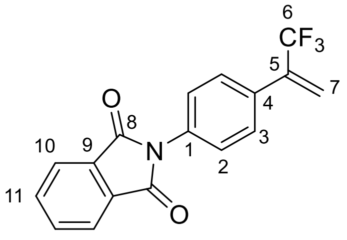

To a solution of 4-(3,3,3-trifluoroprop-1-en-2-yl)aniline (**S3**) (336 mg, 1.80 mmol, 1.0 eq.) in AcOH (8 mL) phthalic anhydride (278 mg, 1.90 mmol, 1.05 eq.) was added as a solid. The suspension was heated to reflux with stirring for 5 h. After completion, the reaction mixture was cooled to ambient temperature and diluted with water (20 mL). The obtained suspension was poured carefully into saturated $NaHCO_3$ (200 mL). The mixture was extracted with DCM (3x 50 mL), and the combined organic layers were dried over $MgSO_4$. The crude product was purified by flash column chromatography (20% EtOAc in cyclohexane) to yield the title compound as a white solid (496 mg, 1.56 mmol, 87%).

R*f* = 0.34 (20% EtOAc in cyclohexane)

M.p.: 136-138 °C.

FT-IR ($\tilde{\nu}$ = cm^{-1}): 1787 (w), 1754 (w), 1705 (s), 1606 (w), 1520 (m), 1467 (w), 1387 (m), 1355 (m), 1322 (w), 1279 (w), 1187 (m), 1174 (s), 1160 (s), 1111 (s), 1074 (s), 1018 (w), 950 (m), 886 (m), 837 (s), 790 (s), 748 (w), 713 (s), 695 (m).

^{1}H NMR (599 MHz, $CDCl_3$, 299 K): δ [ppm] = 7.97 (dd, $^{3}J_{HH}$ = 5.4 Hz, $^{4}J_{HH}$ = 3.2 Hz, 2H, H-C11), 7.81 (dd, $^{3}J_{HH}$ = 5.4 Hz, $^{4}J_{HH}$ = 2.9 Hz, 2H, H-C10), 7.60 (d, $^{3}J_{HH}$ = 8.3 Hz, 2H, H-C2), 7.51 (d, $^{3}J_{HH}$ = 8.6 Hz, 2H, H-C3), 6.02 (q, $^{4}J_{HF}$ = 1.4 Hz, 1H, H^{a}-C7), 5.84 (q, $^{4}J_{HF}$ = 1.7 Hz, 1H, H^{b}-C7).

^{13}C{^{1}H} NMR (151 MHz, $CDCl_3$, 299 K): δ [ppm] = 167.2 (C8), 138.4 (q, $^{2}J_{CF}$ = 30.3 Hz, C5), 134.7 (C11), 133.3 (C4), 132.5 (C1), 131.8 (C9), 128.3 (C3), 126.6 (C2), 124.0 (C10), 123.3 (q, $^{1}J_{CF}$ = 274.0 Hz, C6), 121.3 (q, $^{3}J_{CF}$ = 5.7 Hz , C7).

^{19}F {^{1}H} NMR (376 MHz, $CDCl_3$, 299 K): δ [ppm] = −64.8 (s, 3F, F-C6).

ESI-MS: (*m/z*) requires: $[(C_{17}H_{10}NO_2F_3Na)]^+$ = 340.0556, (*m/z*) found: $[(C_{17}H_{10}NO_2F_3Na)]^+$ = 340.0557.

2-Methoxy-5-(3,3,3-trifluoroprop-1-en-2-yl)phenol (S5)

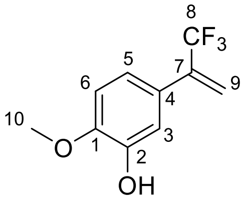

Compound **S5** was prepared according to General Procedure **B** using 4-hydroxy-3-methoxyphenylboronic acid pinacol ester (1.00 g, 4.0 mmol, 1.0 eq.). The crude residue was purified by column chromatography (10% Et_2O in *n*-pentane) to yield the title compound as a colourless liquid (661 mg, 3.0 mmol, 75%).

R_f = 0.55 (20% EtOAc in *n*-pentane).

FT-IR ($\tilde{\nu}$ = cm^{-1}): 3548 (w), 2943 (w), 1621 (w), 1583 (m), 1515 (s), 1463 (m), 1444 (m), 1406 (w), 1365 (m), 1325 (s), 1266 (s), 1238 (m), 1160 (s), 1124 (s), 1081 (m), 1026 (s), 944 (s), 877 (w), 809 (s), 765 (m).

^{1}H NMR (599 MHz, $CDCl_3$): δ [ppm] = 7.06 (dd, $^{4}J_{HH}$ = 2.3 Hz, $^{5}J_{FH}$ =0.7 Hz, 1H, H-C3), 6.99 – 6.96 (m, 1H, H-C5), 6.84 (d, $^{3}J_{HH}$ = 8.4 Hz, 1H, H-C6), 5.87 (q, $^{4}J_{FH}$ = 1.3 Hz, 1H, H^{a}-C9), 5.70 (q, $^{4}J_{FH}$ = 1.7 Hz, 1H, H^{b}-C9), 5.64 (s, 1H, OH), 3.91 (s, 3H, H-C10).

^{13}C{^{1}H} NMR (151 MHz, $CDCl_3$): δ [ppm] = 147.3 (C1), 145.6 (C2), 138.5 (q, $^{2}J_{CF}$ = 29.9 Hz, C7), 127.1 (C4), 123.5 (q, ^{2}JCF = 274.0 Hz, C8), 119.6 (q, ^{4}JCF = 1.4 Hz, C5), 119.4 (q, ^{3}JCF = 5.8 Hz, C7), 113.9 (q, ^{4}JCF = 1.1 Hz, C3), 110.6 (C6), 56.1 (C10).

^{19}F{^{1}H} NMR (564 MHz, $CDCl_3$): δ [ppm] = -64.8 (s, 3F, F-C6).

ESI-MS: (*m/z*) requires: $[(C_{10}H_8O_2F_3)]^-$ = 217.0482, (*m/z*) found: $[(C_{10}H_8O_2F_3)]^-$= 217.0479.

2-Methoxy-5-(3,3,3-trifluoroprop-1-en-2-yl)phenyl trifluoromethanesulfonate (1q)

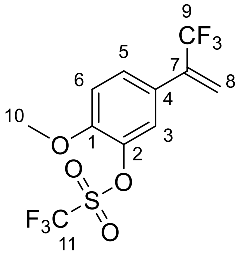

2-Methoxy-5-(3,3,3-trifluoroprop-1-en-2-yl)phenol (**S5**) (589 mg, 2.7 mmol, 1.0 eq.) was added to a round bottom flask. The flask was sealed and purged with argon before the addition of pyridine (5.4 mL, 0.5 M). The reaction mixture was cooled to 0 °C before the dropwise addition of trifilic anhydride (0.5 mL, 2.97 mmol, 1.1 eq.). The reaction mixture was allowed to warm and stirred for 16 h. The solvent was

evaporated under reduced pressure and the crude residue was purified by column chromatography (5% EtOAc in *n*-pentane) to yield the title compound as a colourless liquid (765 mg, 2.2 mmol, 81%).

R_f = 0.35 (5% EtOAc in *n*-pentane).

FT-IR ($\tilde{\nu}$ = cm^{-1}): 2947 (w), 1620 (m), 1519 (s), 1463 (w), 1362 (m), 1299 (s), 1269 (s), 1249 (s), 1199 (s), 1170 (s), 1107 (s), 1084 (m), 1022 (m), 947 (s), 889 (s), 845 (s), 820 (s), 805 (s), 771 (s), 747 (s), 711 (s), 683 (m).

^{1}H NMR (599 MHz, $CDCl_3$): δ [ppm] = 7.43 (ddd, $^3J_{HH}$ = 8.7 Hz, $^4J_{HH}$ = 2.3 Hz, $^5J_{HF}$ = 0.7 Hz, 1H, H-C5), 7.32 (d, $^4J_{HH}$ = 2.2 Hz, 1H, H-C3), 7.05 (d, $^3J_{HH}$ = 8.7 Hz, 1H, H-C6), 5.97 (q, $^4J_{HF}$ = 1.4 Hz, 1H, H^a-C8), 5.75 (q, $^4J_{HF}$ = 1.7 Hz, 1H, H^b-C8), 3.95 (s, 3H, H-C10).

^{13}C{^{1}H} NMR (151 MHz, $CDCl_3$): δ [ppm] = 151.9 (C1), 138.5 (C2), 136.9 (q, $^2J_{CF}$ = 30.6 Hz, C7), 128.3 (q, $^4J_{CF}$ = 1.3 Hz, C5), 126.5 (C4), 123.0 (q, $^1J_{CF}$ = 273.8 Hz, C9), 121.7 (C3), 120.7 (q, $^3J_{CF}$ = 5.7 Hz, C8), 118.7 (q, $^1J_{CF}$ = 320.4 Hz, C11), 113.1 (C6), 56.3 (C10).

^{19}F{^{1}H} NMR (564 MHz, $CDCl_3$): δ [ppm] = -65.1 (s, 3F, F-C9), -73.8 (s, 3F, F-C11).

ESI-MS: (*m/z*) requires: $[(C_{11}H_8O_4SF_6)Na]^+$ = 372.9940, (*m/z*) found: $[(C_{11}H_8O_4SF_6)Na]^+$ = 372.9940.

2.2. Synthesis of Catalysts

General Procedure C:

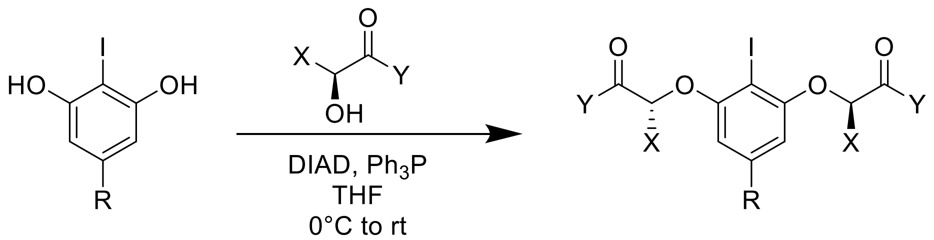

According to a procedure by *Jacobsen et al.,*[11], a resorcinol derivative (1.0 eq.), acyloin derivative (2.1 eq.) and triphenylphosphine (2.7 eq.) were added to an oven-dried Schlenk flask under argon and dissolved in dry THF (0.1 M). The resulting solution was cooled to 0 °C and diisopropyl azodicarboxylate (DIAD) (2.3 eq.) was added dropwise. The reaction mixture was allowed to warm to ambient temperature and stirred overnight. The solvent was evaporated under reduced pressure and the crude residue was purified by column chromatography.

2-Iodobenzene-1,3-diol (S6)

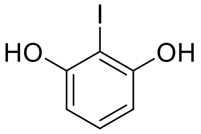

According to a procedure by *Muñiz et al.*[12], resorcinol (5.51 g, 50 mmol, 1.0 eq.) was dissolved in H_2O (50 mL) and cooled to 0 °C. Iodine (13.58 g, 53.5 mmol, 1.07 eq.) was added followed by $NaHCO_3$ (4.66 g, 55.5 mmol, 1.11 eq.) in small portions. The reaction mixture turned light brown with releasing of gas while stirring at ambient temperature for 40 min. The aqueous layer was extracted with EtOAc (3x), the combined organic layers were washed with saturated thiosulfate (aq.) and dried over $MgSO_4$. The solvent was evaporated under reduced pressure and the resulting solid was triturated with ice cold chloroform to yield the title compound as an off-white solid after filtration (7.34 g, 31.3 mmol, 62%).

R_f = 0.74 (50% EtOAc in *n*-pentane).

M.p.: 92-94 °C.

^{1}H NMR (300 MHz, $CDCl_3$): δ [ppm] = 7.11 (t, *J* = 8.1 Hz, 1H), 6.56 (d, *J* = 8.1 Hz, 2H), 5.37 (s, 2H).

ESI-MS (negative mode): (*m/z*) requires: $[(C_6H_5IO_2)]^-$ = 234.9261 (*m/z*) found: $[(C_6H_5IO_2)]^-$ = 234.9277. Analytical data in agreement with the literature data.[12]

2-Iodo-5-methylbenzene-1,3-diol (S7)

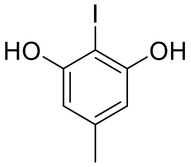

According to a procedure by *Berliner et al.*[13], 5-methylresorcinol (1.24 g, 10.0 mmol, 1.0 eq.) was dissolved in MeCN (0.2 M) and *N*-iodosuccinimide (2.25 g, 10.0 mmol, 1.0 eq.) was added at 0 °C. The reaction mixture was stirred at 0 °C for 15 min. After completion, the reaction mixture was quenched by the addition of a saturated aqueous solution of $Na_2S_2O_3$ (20 mL). The aqueous layer was extracted with EtOAc (3x 50 mL) and the combined organic layers were washed with brine (30 mL), dried over Na_2SO_4, filtered and the solvent was evaporated under reduced pressure. The crude mixture was purified by column chromatography (50% EtOAc in *n*-pentane) to yield the title compound as a white solid (2.34 g, 9.4 mmol, 93%).

R_f = 0.54 (50% EtOAc in *n*-pentane).

M.p.: 105-107 °C.

^{1}H NMR (400 MHz, $CDCl_3$): δ [ppm] = 6.46 – 6.36 (m, 2H), 5.27 (s, 2H), 2.27 – 2.25 (m, 3H).

ESI-MS (negative mode): (*m/z*) requires: $[(C_7H_6IO_2)]^-$ = 248.9418 (*m/z*) found: $[(C_7H_6IO_2)]^-$ = 248.9457. Analytical data in agreement with the literature data.[12]

(*S*)-2-Hydroxy-*N,N*-dimethylpropanamide (S8)

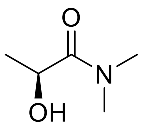

According to a procedure by *Terashima et al.*[14], methyl-*L*-lactate (1.91 mL, 20 mmol, 1.0 eq.) was added to a round bottom flask and dissolved in MeOH (20 mL). The solution was cooled to 0 °C and a 2 M solution of dimethyl amine in EtOH (15 mL, 30 mmol, 1.5 eq.) was added slowly. The reaction mixture was allowed to warm to ambient temperature and was stirred for 5 days. The solvent was evaporated under reduced pressure and the crude residue was purified by column chromatography (10% EtOAc in *n*-pentane) to yield the title compound) as a colourless oil (314 mg, 2.7 mmol, 14%).

R*f* = 0.26 (10% EtOAc in *n*-pentane).

^{1}H NMR (400 MHz, $CDCl_3$): δ [ppm] = 4.46 (p, *J* = 6.7 Hz, 1H), 3.82 (d, *J* = 7.3 Hz, 1H), 3.00 (d, *J* = 10.6 Hz, 6H), 1.32 (d, *J* = 6.6 Hz, 3H).

EI-MS: (*m/z*) requires: $[(C_5H_{11}NO_2H)]^+$ = 118.0863, (*m/z*) found: $[(C_5H_{11}NO_2H)]^+$ = 118.0860; (*m/z*) requires: $[(C_5H_{11}NO_2Na)]^+$ = 140.0682, (*m/z*) found: $[(C_5H_{11}NO_2Na)]^+$ = 140.0679.

Analytical data in agreement with the literature data.[14]

Methyl (*S*)-2-hydroxy-3-mesitylpropanoate (S9)

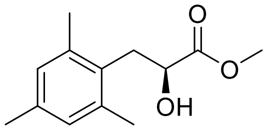

This compound was prepared in a previous report from our research group.[1]

Dimethyl 2,2'-((2-iodo-1,3-phenylene)bis(oxy))diacetate (7)

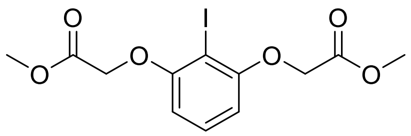

According to a procedure by *Muñiz et al.,*[15] K_2CO_3 (2.76 g, 20.0 mmol, 4.0 eq.) was added to a solution of 2-iodobenzene-1,3-diol (**S6**) (1.18 g, 5.0 mmol, 1.0 eq.) in acetone (40 mL) giving a dark suspension. After addition of methyl bromoacetate (1.89 mL, 20.0 mmol, 4.0 eq.) the reaction was heated to 75 °C for 16 h. The mixture was diluted with H_2O (10 mL) and Et_2O (10 mL) and the separated aqueous layer was extracted with Et_2O (3x 50 mL). The combined organic layers were dried over $MgSO_4$ and concentrated under reduced pressure. The obtained residue was dissolved in CH_2Cl_2 and precipitated by addition of *n*-pentane affording the title compound as a white powder (1.73 g, 4.6 mmol, 91%).

R*f* = 0.67 (50% EtOAc in *n*-pentane).

M.p. : 126-128 °C.

^{1}H NMR (400 MHz, $CDCl_3$): δ [ppm] = 7.21 (t, *J* = 8.3 Hz, 1H), 6.42 (d, *J* = 8.3 Hz, 2H), 4.72 (s, 4H), 3.80 (s, 6H).

ESI-MS: (*m/z*) requires: $[(C_{12}H_{13}IO_6)Na]^+$ = 402.9649, (*m/z*) found: $[(C_{12}H_{13}IO_6)Na]^+$ = 402.9638.
Analytical data in agreement with the literature data.[15]

Dimethyl 2,2'-((2-iodo-5-methyl-1,3-phenylene)bis(oxy))diacetate (8)

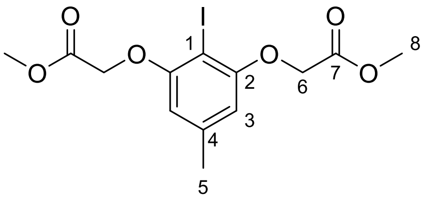

According to a procedure by *Muñiz et al.,*[15] 2-iodo-5-methylbenzene-1,3-diol (**S7**) (1.00 g, 4.0 mmol, 1.0 eq.) was dissolved in acetone (35 mL). Upon addition of K_2CO_3 (2.21 g, 16.0 mmol, 4.0 eq.) a purple suspension was obtained. 1-Bromopropane-2-one (1.51 mL, 16.0 mmol, 4.0 eq.) was added and the mixture was heated to 75°C for 16 h. The mixture was diluted with H_2O (10 mL) and Et_2O (10 mL) and the aqueous layer was extracted with Et_2O (3x). The combined organic layers were dried over $MgSO_4$ and concentrated under reduced pressure to give an orange-brown solid. The residue was dissolved in hot EtOAc and then precipitated by addition of *n*-hexane at ambient temperature to yield the title compound as a white crystalline solid (1.09 g, 2.77 mmol, 69%).

R*f* = 0.62 (50% EtOAc in *n*-pentane).

M.p. : 118-120 °C.

FT-IR ($\tilde{\nu}$ = cm^{-1}): 2954 (w), 1756 (m), 1738 (m), 1580 (m), 1435 (m), 1378 (w), 1327 (w), 1285 (w), 1206 (s), 1130 (s), 1086 (w), 1021 (w), 1002 (w), 848 (w), 810 (w), 706 (w).

^{1}H NMR (599 MHz, $CDCl_3$): δ [ppm] = 6.24 (d, $^4J_{HH}$ = 0.7 Hz, 2H, H-C3), 4.68 (s, 4H, H-C6), 3.80 (s, 6H, H-C8), 2.29 (s, 3H, H-C5).

^{13}C{^{1}H} NMR (126 MHz, $CDCl_3$): δ [ppm] = 169.0 (C7), 158.2 (C2), 140.6 (C4), 107.5 (C3), 75.4 (C1), 66.6 (C6), 52.4 (C8), 22.0 (C5).

ESI-MS: (*m/z*) requires: $[(C_{13}H_{15}IO_6)Na]^+$ = 416.9806, (*m/z*) found: $[(C_{13}H_{15}IO_6)Na]^+$ = 416.9832.

Dimethyl 2,2'-((2-iodo-5-(methoxycarbonyl)-1,3-phenylene)bis(oxy))diacetate (9)

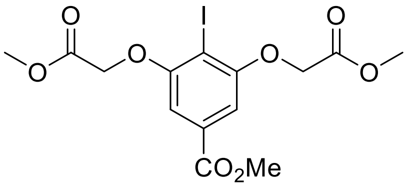

Methyl 3,5-dihydroxy-4-iodobenzoate (1.18 g, 4.0 mmol, 1.0 eq.) was dissolved in acetone and K_2CO_3 (2.21 g, 16.0 mmol, 4.0 eq.), was added giving an orange-brown suspension. After the addition of bromopropane-2-one (1.51 mL, 16.0 mmol, 4.0 eq.) the reaction was heated to 75 °C for 20 h. The mixture was diluted with H_2O (50 mL) and Et_2O (50 mL). The combined organic layers were dried over $MgSO_4$ and concentrated under reduced pressure to give a yellow solid. Recrystallisation from boiling EtOAc afforded a white crystalline solid (1.42 g, 3.24 mmol, 81%).

R*f* = 0.44 (50% EtOAc in *n*-pentane).

M.p. : 142-144 °C.

^{1}H NMR (400 MHz, $CDCl_3$): δ [ppm] = 7.07 (d, *J* = 1.2 Hz, 1H), 4.78 (s, 2H), 3.90 (d, *J* = 1.3 Hz, 2H), 3.82 (d, *J* = 1.2 Hz, 3H).

ESI-MS: (*m/z*) requires: $[(C_{14}H_{15}IO_8)Na]^+$ = 460.9706, (*m/z*) found: $[(C_{14}H_{15}IO_8)Na]^+$ = 460.9705.

Analytical data in agreement with the literature data.[16]

Dimethyl 2,2'-((2-iodo-5-(methoxycarbonyl)-1,3-phenylene)bis(oxy))(*2R,2'R*)-dipropionate (10)

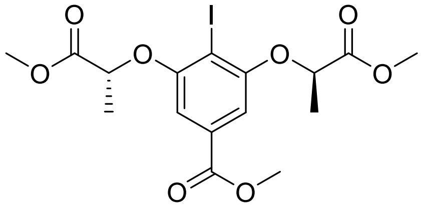

Compound **10** was prepared according to General Procedure **C** using methyl 3,5-dihydroxy-4-iodobenzoate (5.88 g, 20.0 mmol, 1.0 eq.) and methyl-*L*-lactate (4.0 mL, 42.0 mmol). The crude residue was purified by column chromatography (30% EtOAc in *n*-pentane) to yield the title compound as an off-white solid (5.49 g, 11.8 mmol, 59%).

Single crystals suitable for X-ray crystallographic analysis were obtained *via* slow evaporation of a solution of *n*-pentane / cyclohexane.

R_f = 0.23 (30% EtOAc in *n*-pentane).

M.p.: 86-88 °C.

ORD ($CHCl_3$, c 1.00) $[\alpha]_D^{25}$ = +10.159.

^{1}H NMR (300 MHz, $CDCl_3$): δ [ppm] = 7.01 (s, 2H), 4.88 (q, *J* = 6.8 Hz, 2H), 3.88 (s, 3H), 3.76 (s, 6H), 1.71 (d, *J* = 6.8 Hz, 6H).

^{13}C NMR (75 MHz, $CDCl_3$): δ [ppm] = 171.8, 166.2, 158.3, 131.8, 107.3, 87.4, 74.2, 52.6, 18.6.

ESI-MS: (*m/z*) requires: $[(C_{16}H_{19}IO_8)Na]^+$ = 489.0017, (*m/z*) found: $[(C_{16}H_{19}IO_8)Na]^+$ = 489.0022.

Analytical data in agreement with the literature data.[17]

Dimethyl 2,2'-((2-iodo-5-(methoxycarbonyl)-1,3-phenylene)bis(oxy))(*2R,2'R*)-dipropionate (11)

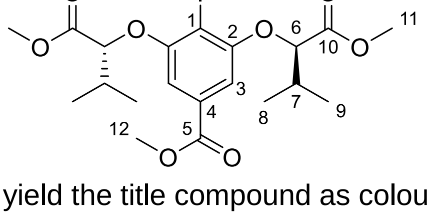

Compound **11** was prepared according to General Procedure **C** using methyl 3,5-dihydroxy-4-iodobenzoate (294 mg, 1 mmol, 1.0 eq.) and methyl-*L*-lactate (277.5 mg, 2.1 mmol). The crude mixture was purified by column chromatography (20% EtOAc in *n*-pentane) to yield the title compound as colourless oil (441 mg, 0.85 mmol, 85%).

R_f = 0.44 (20% EtOAc in *n*-pentane)

ORD ($CHCl_3$, c 1.00) $[\alpha]_D^{25}$ = +31.896

FT-IR ($\tilde{\nu}$ = cm^{-1}): 2969 (w), 1756 (s), 1727 (s), 1578 (m), 1421 (s), 1331 (m), 1245 (s), 1136 (s), 116 (s), 1007 (w), 766 (w).

^{1}H NMR (599 MHz, $CDCl_3$): δ [ppm] = 6.91 (s, 2H, H-C3), 4.58 (d, $^{3}J_{HH}$ = 4.5 Hz, 2H, H-C6), 3.87 (s, 3H, H-C12), 3.75 (s, 6H, H-C11), 2.38 (pd, $^{3}J_{HH}$ = 6.9, 4.5 Hz, 2H, H-C7), 1.17 (m, 6H, H-C8), 1.14 (d, $^{3}J_{HH}$ = 6.9 Hz, 6H, H-C9).

^{13}C{^{1}H} NMR (151 MHz, $CDCl_3$): δ [ppm] = 170.9 (C10), 166.3 (C5), 158.3 (C2), 131.8 (C4), 105.9 (C3), 86.0 (C1), 82.2 (C6), 52.6 (C12), 52.3 (C11), 32.0 (C7), 19.2 (C9), 17.6 (C8).

ESI-MS: (*m/z*) requires: $[(C_{20}H_{27}IO_8)Na]^+$ = 545.06428, (*m/z*) found: $[(C_{20}H_{27}IO_8)Na]^+$ = 545.06365.

Dimethyl 2,2'-((2-iodo-5-methyl-1,3-phenylene)bis(oxy))(*2R,2'R*)-bis(3-phenylpropanoate) (12)

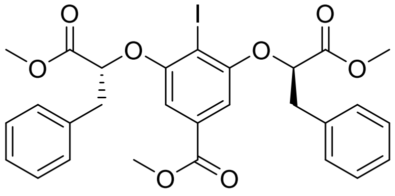

This compound was prepared in a previous report from our research group.[18]

Dimethyl 2,2'-((2-iodo-5-methyl-1,3-phenylene)bis(oxy))(*2R,2'R*)-bis(3-phenylpropanoate) (13)

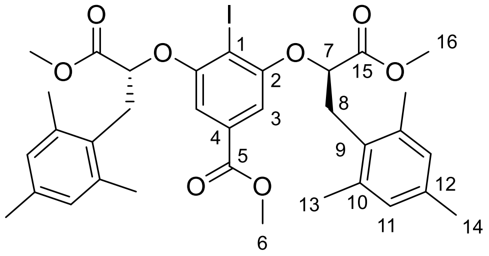

Compound **13** was prepared according to General Procedure **C** using methyl 3,5-dihydroxy-4-iodobenzoate (294 mg, 1.0 mmol, 1.0 eq.) and methyl (*S*)-2-hydroxy-3-mesitylpropanoate (**S9**) (466.8 mg, 1.6 mmol 2.1 eq). The crude residue was purified by column chromatography (30% EtOAc in *n*-pentane) to yield the title compound as an off-white solid (322.0 mg, 0.5 mmol, 50%).

R_f = 0.71 (30% EtOAc in *n*-pentane).

M.p.: 62-64 °C.

ORD ($CHCl_3$, c 1.00) $[\alpha]_D^{25}$ = +11.702.

FT-IR ($\tilde{\nu}$ = cm^{-1}): 2952 (w), 1756 (m), 1724 (m), 1613 (w), 1575 (m), 1485 (w), 1435 (m), 1417 (m), 1378 (w), 1323 (m), 1282 (w), 1241 (s), 1177 (m), 1107 (s), 1009 (m), 909 (m), 853 (w), 765 (m), 730 (s).

^{1}H NMR (500 MHz, $CDCl_3$): δ [ppm] = 6.85 (s, 4H, H-C11), 6.80 (s, 2H, H-C3), 4.92 (dd, ,$^{3}J_{HH}$ = 9.1, 4.5 Hz, 2H, H-C7), 3.82 (s, 3H, H-C6), 3.70 (s, 6H, H-C16), 3.46 (dd, $^{2}J_{HH}$ = 14.6 Hz, $^{3}J_{HH}$ = 9.1 Hz, 2H, H^a-C8), 3.30 (dd, $^{2}J_{HH}$ = 14.6 Hz, $^{3}J_{HH}$ = 4.4 Hz, 2H, H^b-C8), 2.45 (s, 12H, H-C13), 2.24 (s, 6H, H-C14).

^{13}C{^{1}H} NMR (126 MHz, $CDCl_3$): δ [ppm] = 171.2 (C15), 166.1 (C5), 158.5 (C4), 137.3 (C12), 136.5 (C10), 131.6 (C2), 130.2 (C9), 129.3 (C11), 106.9 (C3), 86.6 (C1), 78.0 (C7), 52.5 (C16), 52.5 (C6), 32.7 (C8), 21.0 (C14), 20.8 (C13).

ESI-MS: (*m/z*) requires: $[(C_{34}H_{39}IO_8)Na]^+$ = 725.1582, (*m/z*) found: $[(C_{34}H_{39}IO_8)Na]^+$ = 725.1576.

Dibenzyl 2,2'-((2-Iodo-5-(methoxycarbonyl)-1,3-phenylene)bis(oxy))(*2R,2'R*)-dipropionate (14)

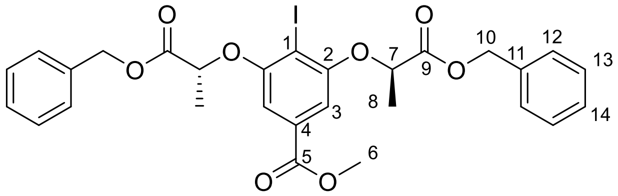

Compound **14** was prepared according to General Procedure **C** using methyl 3,5-dihydroxy-4-iodobenzoate (660 mg, 2.3 mmol, 1.0 eq.) and benzyl-*S*-lactate (761 mL, 4.7 mmol). The crude residue was purified by column chromatography (30% EtOAc in *n*-pentane) to yield the title compound as a colourless oil (1.26 g, 2.03 mmol, 90%).

R*f* = 0.55 (30% EtOAc in *n*-pentane).

ORD ($CHCl_3$, c 1.00) $[\alpha]_D^{25}$ = +3.646.

FT-IR ($\tilde{\nu}$ = cm^{-1}): 2953 (w), 1751 (m), 1722 (m), 1576 (m), 1498 (w), 1456 (w), 1419 (m), 1322 (w), 1244 (s), 1191 (m), 1131 (s), 1109 (s), 1073 (w), 1012 (w); 953 (w), 915 (w), 864 (w), 751 (w), 698 (m).

^{1}H NMR (600 MHz, $CDCl_3$): δ [ppm] = 7.32 – 7.28 (m, 10H, H-C_{Ar}), 7.00 (s, 2H, H-C3), 5.19 (dd, $^3J_{HH}$ = 12.3 Hz, 4H, H-C10), 4.92 (q, $^3J_{HH}$ = 6.8 Hz, 2H, H-C7), 3.82 (s, 3H, H-C6), 1.73 (d, $^3J_{HH}$ = 6.8 Hz, 6H, H-C8).

^{13}C{^{1}H} NMR (126 MHz, $CDCl_3$): δ [ppm] = 171.1 (C9), 166.0 (C5), 158.2 (C2), 135.3 (C11), 131.8 (C4), 128.7 (C13), 128.5 (C14), 128.3 (C12), 107.2 (C3), 87.3 (C1), 74.1 (C7), 67.2 (C10), 52.5 (C6), 18.5 (C8).

ESI-MS: (*m/z*) requires: $[(C_{28}H_{27}IO_8)Na]^+$ = 641.0643, (*m/z*) found: $[(C_{28}H_{27}IO_8)Na]^+$ = 641.0648.

Methyl 3,5-bis(((*R*)-1-(dimethylamino)-1-oxopropan-2-yl)oxy)-4-iodobenzoate (15)

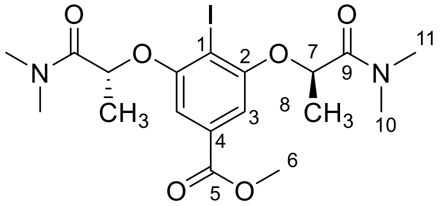

Compound **15** was prepared according to General Procedure **C** using methyl 3,5-dihydroxy-4-iodobenzoate (323 mg, 1.1 mmol, 1.0 eq.) and (*S*)-2-hydroxy-*N*,*N*-dimethylpropanamide (**S8**) (258 mg, 2.2 mmol). The crude residue was purified by column chromatography (10% EtOAc in cyclohexane) to yield the title compound as a white solid (291 mg, 0.59 mmol, 54%).

R*f* = 0.13 (10% EtOAc in cyclohexane).

M.p.: 132-134 °C**.**

ORD (CHCl3, c 1.00) $[\alpha]_D^{25}$ = -102.531.

FT-IR ($\tilde{\nu}$ = cm^{-1}): 2953 (w), 1721 (m), 1650 (s), 1575 (m), 1418 (m), 1361 (w), 1343 (w), 1234 (s), 1171 (w), 1103 (s), 1017 (m), 922 (w), 856 (w), 765 (w), 731 (m), 665 (w).

^{1}H NMR (600 MHz, $CDCl_3$): δ [ppm] = 7.06 (s, 2H, H-C3), 5.06 (q, $^3J_{HH}$ = 6.7 Hz, 2H, H-C7), 3.87 (s, 3H, H-C6), 3.11 (s, 6H, H-C10), 2.94 (s, 6H, H-C11), 1.67 (d, $^3J_{HH}$ = 6.7 Hz, 6H, H-C8).

^{13}C{^{1}H} NMR (126 MHz, $CDCl_3$): δ [ppm] = 170.0 (C9), 166.3 (C5), 157.8 (C2), 132.3 (C4), 106.7 (C3), 86.1 (C1), 75.1 (C7), 52.7 (C6), 37.1 (C10), 36.5 (C11), 17.6 (C8).

ESI-MS: (*m/z*) requires: $[(C_{18}H_{25}IN_2O_6)Na]^+$ = 515.0650, (*m/z*) found: $[(C_{18}H_{25}IN_2O_6)Na]^+$ = 515.0672.

Methyl 4-iodo-3,5-bis(((*R*)-1-(methylamino)-1-oxopropan-2-yl)oxy)benzoate (16)

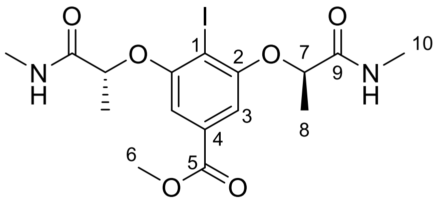

A round bottom flask was charged with dimethyl 2,2'-((2-iodo-5-(methoxycarbonyl)-1,3-phenylene)bis(oxy))(*2R,2'R*)-dipropionate (**10**) (233 mg, 0.5 mmol, 1.0 eq.) and a 2 M solution of methyl amine in EtOH (15 mL, 30 mmol, 60 eq.) was added. The reaction was monitored by TLC and stopped after 25 min. The solvent was evaporated under reduced pressure and the off white solid was suspended in acetone. The residue was filtered off and dried before column chromatography (EtOAc) afforded the product (160 mg, 0.35 mmol, 70%) as a pale yellow solid.

$\mathbf{R_f}$ = 0.31 (EtOAc).

M.p.: 130-132 °C.

ORD ($CHCl_3$, c 1.00) $[\alpha]_D^{25}$ = -109.204.

FT-IR ($\tilde{\nu}$ = cm^{-1}): 3279 (w), 1721 (m), 1661 (s), 1571 (m), 1416 (m), 1232 (m), 1155 (m), 1106 (m), 1012 (w), 762 (w).

^{1}H NMR (600 MHz, $CDCl_3$): δ [ppm] = 7.14 (s, 2H, H-C3), 6.80 (broad s, 2H, NH), 4.87 (q, $^3J_{HH}$ = 6.7 Hz, 2H, H-C7), 3.90 (s, 3H, H-C6), 2.89 (d, $^3J_{HH}$ = 4.9 Hz, 6H, H-C10), 1.64 (d, $^3J_{HH}$ = 6.7 Hz, 6H, H-C8).

^{13}C{^{1}H} NMR 126 MHz, $CDCl_3$): δ [ppm] = 171.3 (C9), 165.7 (C5), 157.1 (C4), 132.9 (C4), 107.6 (C3), 87.1 (C1), 76.4 (C7), 52.8 (C6), 26.2 (C10), 18.5 (C8).

ESI-MS: (*m/z*) requires: $[(C_{16}H_{21}IN_2O_6)Na]^+$ = 487.0337, (*m/z*) found: $[(C_{16}H_{21}IN_2O_6)Na]^+$ = 487.0344.

Methyl (*R*)-4-iodo-3-((1-methoxy-1-oxopropan-2-yl)oxy)benzoate (17)

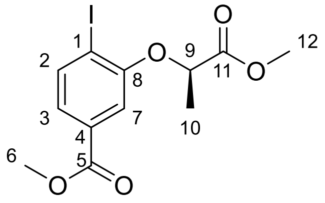

Compound **17** was prepared according to General Procedure **C** using methyl 3-hydroxy-4-iodobenzoate (2.70 g, 9.7 mmol, 1.0 eq.) and methyl-*L*-lactate (1.02 mL, 10.7 mmol). The crude mixture was purified by column chromatography (10% EtOAc: *n*-pentane) to yield the title compound as a white solid (3.28 g, 9.0 mmol, 93%).

$\mathbf{R_f}$ = 0.50 (10% EtOAc: *n*-pentane).

M.p.: 71 – 73°C

FT-IR ($\tilde{\nu}$ = cm^{-1}): 2954 (w), 1757 (s), 1722 (s), 1571 (m), 1475 (m), 1436 (s), 1407 (s), 1290 (s), 1234 (s), 1138 (s), 1115 (s), 1052 (m), 1020 (s), 1003 (m), 879 (w), 795 (w), 756 (s).

ORD ($CHCl_3$, c 1.00) $[\alpha]_D^{25}$ = +16.616

^{1}H NMR (500 MHz, $CDCl_3$): δ [ppm] = 7.87 (d, $^3J_{HH}$ = 8.1 Hz, 1H, H-C2), 7.38 (dd, $^3J_{HH}$ = 8.1 Hz, $^4J_{HH}$ = 1.8 Hz, 1H, H-C3), 7.32 (d, $^4J_{HH}$ = 1.8 Hz, 1H, H-C7), 4.88 (q, $^3J_{HH}$ = 6.8 Hz, 1H, H-C9), 3.89 (s, 3H, H-C6), 3.77 (s, 3H; H-C12), 1.71 (d, $^3J_{HH}$ = 6.8 Hz, 3H).

^{13}C{^{1}H} NMR (126 MHz, $CDCl_3$): δ [ppm] = 171.7 (C11), 166.4 (C5), 156.8 (C8), 140.0 (C2), 131.7 (C4), 124.3 (C3), 113.4 (C7), 94.1 (C1), 74.1 (C9), 52.6 (C12), 52.5 (C6), 18.6 (C10).

ESI-MS: (*m/z*) requires: $[(C_{12}H_{13}IO_5)Na]^+$ = 386.9700, (*m/z*) found: $[(C_{12}H_{13}IO_5)Na]^+$ = 386.9697.

Methyl (*R*)-3-((1-(benzyloxy)-1-oxopropan-2-yl)oxy)-4-iodobenzoate (18)

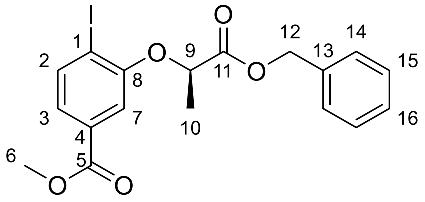

Compound **18** was prepared according to General Procedure **C** using methyl 3-hydroxy-4-iodobenzoate (556.1 mg, 2.0 mmol, 1.0 eq.) and benzyl-*L*-lactate (354 µL, 2.2 mmol, 1.1 equiv.) The crude residue was purified by column chromatography (5% EtOAc in *n*-pentane) to yield the title compound as a colourless oil (708.2 g, 1.61 mmol, 81%).

R$_f$ = 0.19 (5% EtOAc in *n*-pentane).

FT-IR ($\tilde{\nu}$ = cm^{-1}): 2950 (w), 1754(s), 1722 (s), 1586 (m), 1571 (m), 1475 (m), 1436 (s), 1407 (s), 1289 (s), 1257 (s), 1234 (s), 1190 (s), 1136 (s), 1115 (s), 1049 (m), 1020 (s), 1003 (m), 957 (w), 878 (w), 794 (w), 759 (s), 698 (m).

ORD ($CHCl_3$, c 1.00) $[\alpha]_D^{25}$ = +17.985

^{1}H NMR (599 MHz, $CDCl_3$): δ [ppm] = 7.87 (d, $^3J_{HH}$ = 8.1 Hz, 1H, H-C2), 7.38 (dd, $^3J_{HH}$ = 8.1 Hz, $^4J_{HH}$ =1.8 Hz, 1H, H-C3), 7.34 – 7.27 (m, 6H, H-C$_{Ar}$), 5.24 – 5.14 (m, 2H, H-C12), 4.93 (q, $^3J_{HH}$ = 6.8 Hz, 1H, H-C9), 3.87 (s, 3H, H-C6), 1.72 (d, $^3J_{HH}$ = 6.8 Hz, 3H, H-C10).

^{13}C{^{1}H} NMR (151 MHz, $CDCl_3$): δ [ppm] = 171.0 (C11), 166.3 (C5), 156.7 (C8), 140.0 (C2), 135.3 (C13), 131.6 (C4), 128.7 (C$_{Ar}$), 128.5 (C$_{Ar}$), 128.4 (C$_{Ar}$), 124.3 (C3), 113.3 (C7), 94.0 (C1), 74.0 (C9), 67.3 (C12), 52.5 (C6), 18.5 (C10).

ESI-MS: (*m/z*) requires: $[(C_{18}H_{17}IO_5)Na]^+$ = 463.00129, (*m/z*) found: $[(C_{12}H_{13}IO_5)Na]^+$ = 463.00052.

Methyl (*R*)-4-iodo-3-((1-methoxy-1-oxo-3-phenylpropan-2-yl)oxy)benzoate (19)

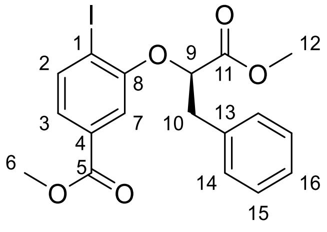

Compound **19** was prepared according to General Procedure **C** using methyl 3-hydroxy-4-iodobenzoate (367.0 mg, 1.3 mmol, 1.0 eq.) and methyl (*S*)-2-hydroxy-3-phenylpropanoate (239.1 mg, 1.33 mmol, 1.1 equiv.) The crude residue was purified by column chromatography (10% EtOAc in *n*-pentane) to yield the title compound as a white solid (319.0 mg, 0.72 mmol, 55%)

R$_f$ = 0.32 (10% EtOAc in *n*-pentane).

M.p.: 83 – 85°C

FT-IR ($\tilde{\nu}$ = cm^{-1}): 2952 (w), 1757 (s), 1722 (s), 1570 (m), 1475 (m), 1436 (m), 1407 (m), 1292 (s), 1257 (s), 1233 (s), 1112 (s), 1082 (m), 1019 (s), 876 (w), 790 (w), 759 (s), 701 (m).

ORD ($CHCl_3$, c 1.00) $[\alpha]_D^{25}$ = 32.499

^{1}H NMR (599 MHz, $CDCl_3$): δ [ppm] = 7.85 (d, $^3J_{HH}$ = 8.1 Hz, 1H, H-C2), 7.44 – 7.40 (m, 2H, H-C14), 7.36 (dd, $^3J_{HH}$ = 8.1 Hz, $^4J_{HH}$ = 1.7 Hz, 1H, H-C3), 7.34 – 7.30 (m, 2H, H-C15), 7.29 – 7.24 (m, 1H, H-C16), 7.22 (d, $^4J_{HH}$ = 1.8 Hz, 1H, H-C7), 4.97 (dd, $^3J_{HH}$ = 7.3 Hz, $^3J_{HH}$ = 5.2 Hz, 1H, H-C9), 3.88 (s, 3H, H-C6), 3.73 (s, 3H, H-C12), 3.37 (m, 2H, HC-10)

^{13}C{^{1}H} NMR (151 MHz, $CDCl_3$): δ [ppm] = 170.5 (C11), 166.4 (C5), 156.7 (C8), 140.1 (C2), 136.0 (C13), 131.5 (C4), 130.0 (C14), 128.6 (C15), 127.2 (C16), 124.2 (C3), 112.7 (C7), 93.5, 78.7 (C9), 52.5 (C6), 52.5 (C12), 39.1 (C10).

ESI-MS: (*m/z*) requires: $[(C_{18}H_{17}IO_5)Na]^+$ = 463.00129, (*m/z*) found: $[(C_{12}H_{13}IO_5)Na]^+$ = 463.00077.

2.3. Hammett Correlation Study

The reactivity of electronically different styrene derivatives was explored by modifying the amine:HF ratio. Based on the following table, a Hammett plot to correlate selectivity is demonstrated showing that for high σ_p^+ values, high amine:HF ratios are necessary to form the *vicinal* fluorinated product.

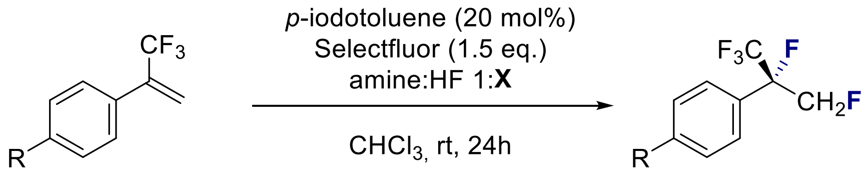

Table S1. Different *para*-substituents and their corresponding Hammett values and experimental results.

Entry	R	σ_p^+	amine:HF ratio	^{19}F-NMR yield (*vic*) [%]
1	OMe	-0.78	1:4.5	83
			1:6	32
			1:7.5	0
2	Me	-0.31	1:4.5	0
			1:6	88
			1:7.5	87
3	*i*Pr	-0.28	1:4.5	0
			1:6	87
			1:7.5	79
4	*t*Bu	-0.26	1:4.5	0
			1:6	87
			1:7.5	74
5	CH_2OMe	-0.05	1:4.5	0
			1:6	34
			1:7.5	63
6	Ar-NO_2	0.04	1:4.5	0
			1:6	19
			1:7.5	60
7	Cl	0.11	1:4.5	0
			1:6	0
			1:7.5	63
8	Br	0.15	1:4.5	0
			1:6	0
			1:7.5	64

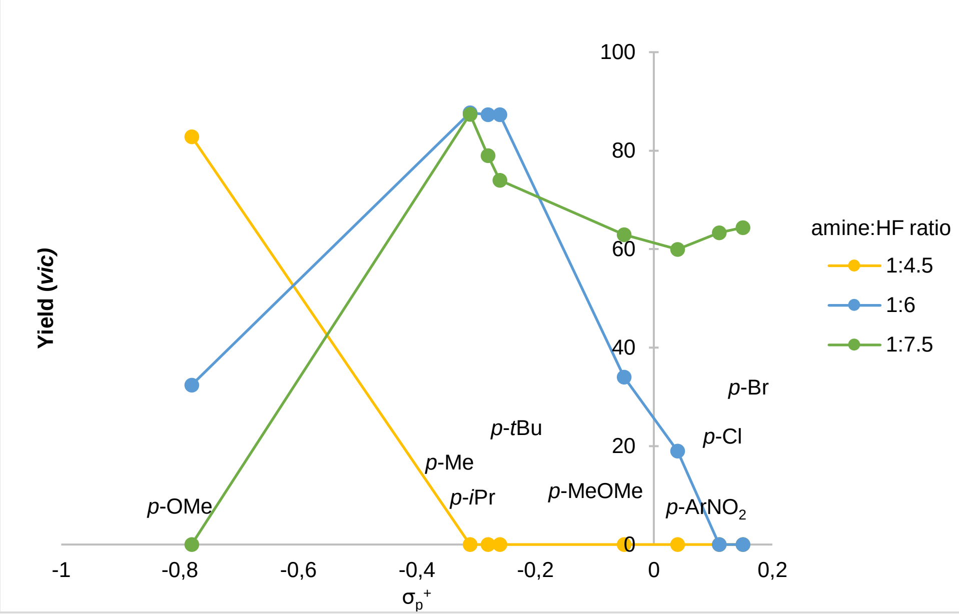

Figure S1. Effect of arene electron-density and Brønsted acidity on yield.

2.4. Synthesis of Racemic Pentafluoroisopropyl Surrogates

Caution Note

Working with hydrofluoric acid (HF) requires several safety guidelines and the prevention of exposure must be the primary goal. Skin contact must be avoided and a tube of 2.5% calcium gluconate gel must be present. For further information please see the corresponding SDS-Datasheet. Please also note that the products are often highly volatile and care must be taken in the isolation.

General procedure D for the racemic 1,2-difluorination

Unless stated otherwise, a Teflon® vial was equipped with a 1 cm stirring bar followed by the addition of styrene (0.2 mmol, 1.0 eq.), *p*-iodotoluene (9 mg, 0.04 mmol, 20 mol%) and $CHCl_3$ (0.5 mL). The mixture was stirred and the stated amine:HF mixture was added (0.5 mL) via syringe. After stirring for 1 min, Selectfluor® (106 mg, 0.3 mmol, 1.5 eq.) was added in one portion. The reaction vessel was then sealed with a Teflon® screw cap. After stirring (350 rpm) at ambient temperature for 24 h, DCM (1 mL) was added to dilute the reaction and a saturated aqueous solution of $NaHCO_3$ (1 mL) was added via a long glass pipette to prequench the reaction. The mixture was poured in an Erlenmeyer flask charged with 100 mL of a saturated aqueous solution of $NaHCO_3$ (CAUTION, strong generation of CO_2!). The Teflon® vial was rinsed with DCM and dropped into another flask with saturated aqueous solution of $NaHCO_3$ to guarantee the removal of excess HF. The organics were extracted with DCM (3x 30 mL) and the combined organic layers were dried over $MgSO_4$ and solvent was carefully removed under reduced pressure. An internal standard (α-, α-, α-trifluorotoluene) was added to the crude residue and the NMR

yield and regioselectivity ratio (*vicinal:geminal*) was analysed by ^{19}F NMR spectroscopy against the internal standard. The NMR sample was recombined with the crude residue and purification by column chromatography or preparative thin layer chromatography yielded the desired product.

NMR Analysis

To support the correct description of the ^{19}F NMR experiment, especially the coupling constants *values J* and the spin multiplicities of 1-methoxy-4-(1,1,1,2,3-pentafluoropropan-2-yl)benzene (**2b**), the respective spectra were simulated with the program MestReNova version 14.2.0-26256. The coupling constants were taken from the corresponding ^{1}H NMR and $^{19}F\{^{1}H\}$ NMR data and optimised with the program.

^{1}H NMR (500 MHz, $CDCl_3$): δ [ppm] = 4.93 (ddd, $^{2}J_{HF}$ = 47.3 Hz, $^{3}J_{HF}$ = 21.0 Hz, $^{2}J_{HH}$ = 11.4 Hz, 1H, H^{a}-C7), 4.83 (dddq, $^{2}J_{HF}$ = 46.5 Hz, $^{3}J_{HF}$ = 21.4 Hz, $^{2}J_{HH}$ = 11.1 Hz, $^{4}J_{HF}$ = 1.3 Hz, 1H, H^{b}-C7).

^{19}F{^{1}H} NMR (376 MHz, $CDCl_3$): δ [ppm] = -77.6 (dd, $^{4}J_{FF}$ = 8.6 Hz, $^{3}J_{FF}$ = 7.1 Hz, F-C6), -178.4 (dq, $^{3}J_{FF}$ = 14.0 Hz, $^{3}J_{FF}$ = 7.1 Hz, F-C5), -233.4 (dq, $^{3}J_{FF}$ = 13.3 Hz, $^{4}J_{FF}$ = 8.6 Hz, F-C7).

In Figures S2-S4, the experimental data of the ^{19}F NMR and $^{19}F\{^{1}H\}$ NMR are stacked with the corresponding simulation and the following parameters:

Table S2. Experimental shifts

Label	Shift (ppm)	N	Spin	Line Width (Hz)
F-C6	-77.6521	3	1/2	2
F-C7	-233.446	1	1/2	2
F-C5	-178.425	1	1/2	2
H^{a}-C7	4.925	1	1/2	3
H^{b}-C7	4.836	1	1/2	2.5

Table S3. Corresponding coupling constants *J*

	F-C6	F-C7	F-C5	H^a-C7	H^b-C7
F-C6					
F-C7	8.7				
F-C5	7.04	13.6			
H^a-C7		47	21		
H^b-C7	1.2	47	21	11.3	

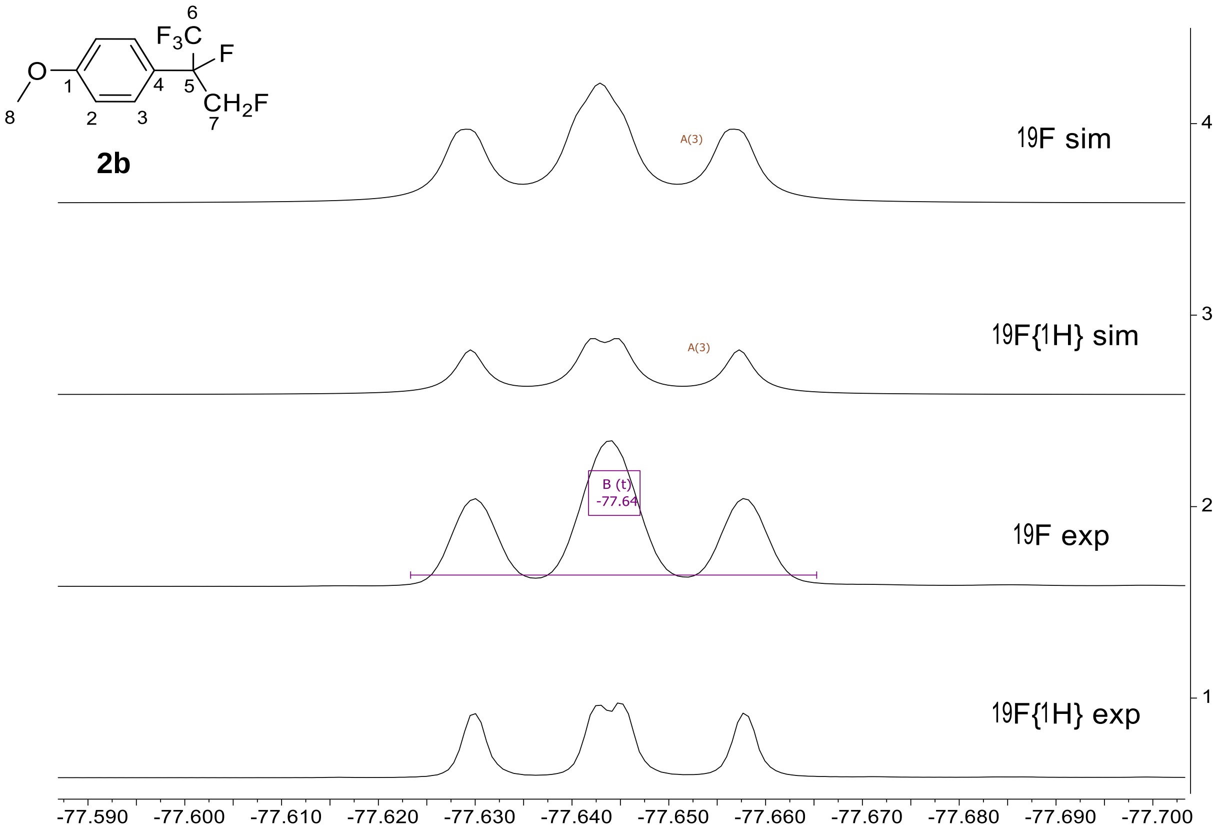

Figure S2. Stacked ^{19}F spectra of the resonance of the CF_3 group (F-C6) of compound **2b**. Top) data of the simulation (sim); bottom) data of the experimental measurement (exp).

The CF_3 group at C6 is adjacent to 2 fluorine atoms and 2 protons. As the two protons of C7 are diastereotopic, the signal should appear as a dddd. Because of steric reasons, only one $^4J_{HF}$ coupling constant is visible in the ^{1}H NMR, the observed multiplicity is an unresolved doublet of doublet of doublet (ddd).

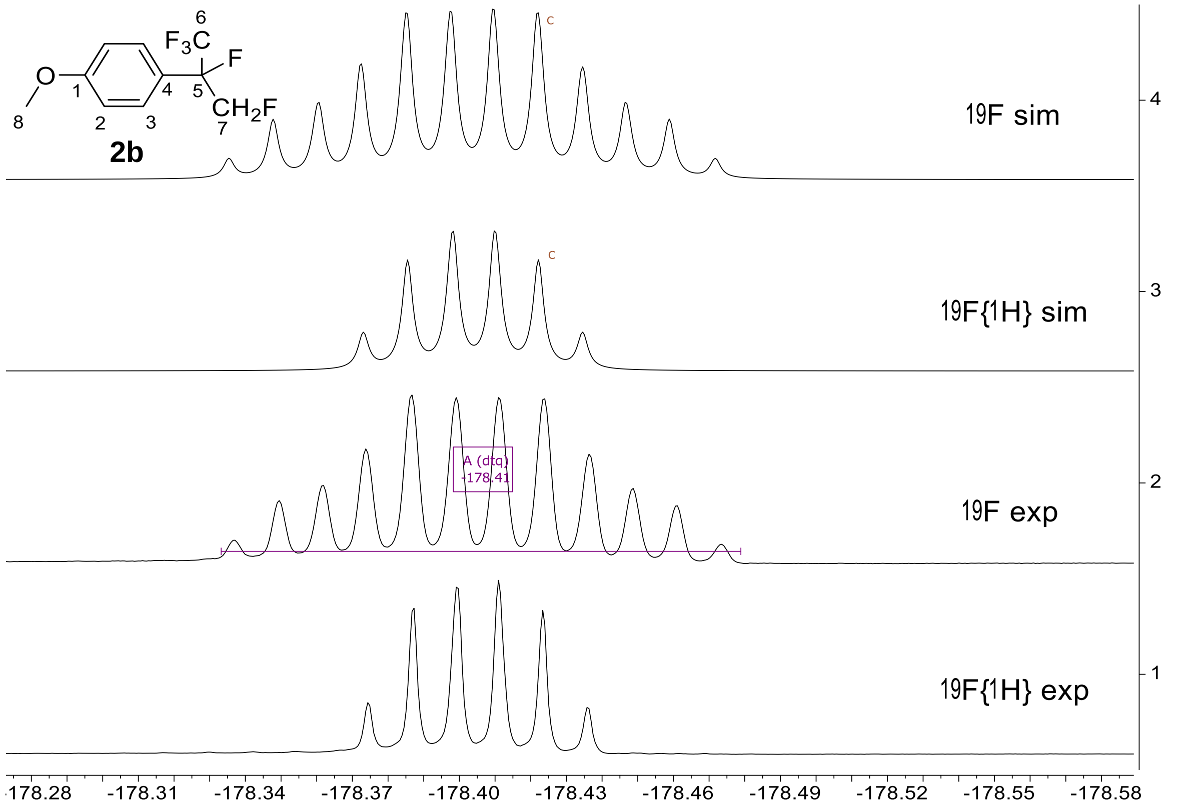

Figure S3. Stacked ^{19}F spectra of the resonance of the CF group (F-C5) of compound **2b**. Top) data of the simulation (sim); bottom) data of the experimental measurement (exp).

The fluorine at C5 is neighboured by 4 fluorine atoms and two diastereotopic protons. Therefore, the observed multiplicity is an unresolved doublet of doublet of doublet of quartet (dddq).

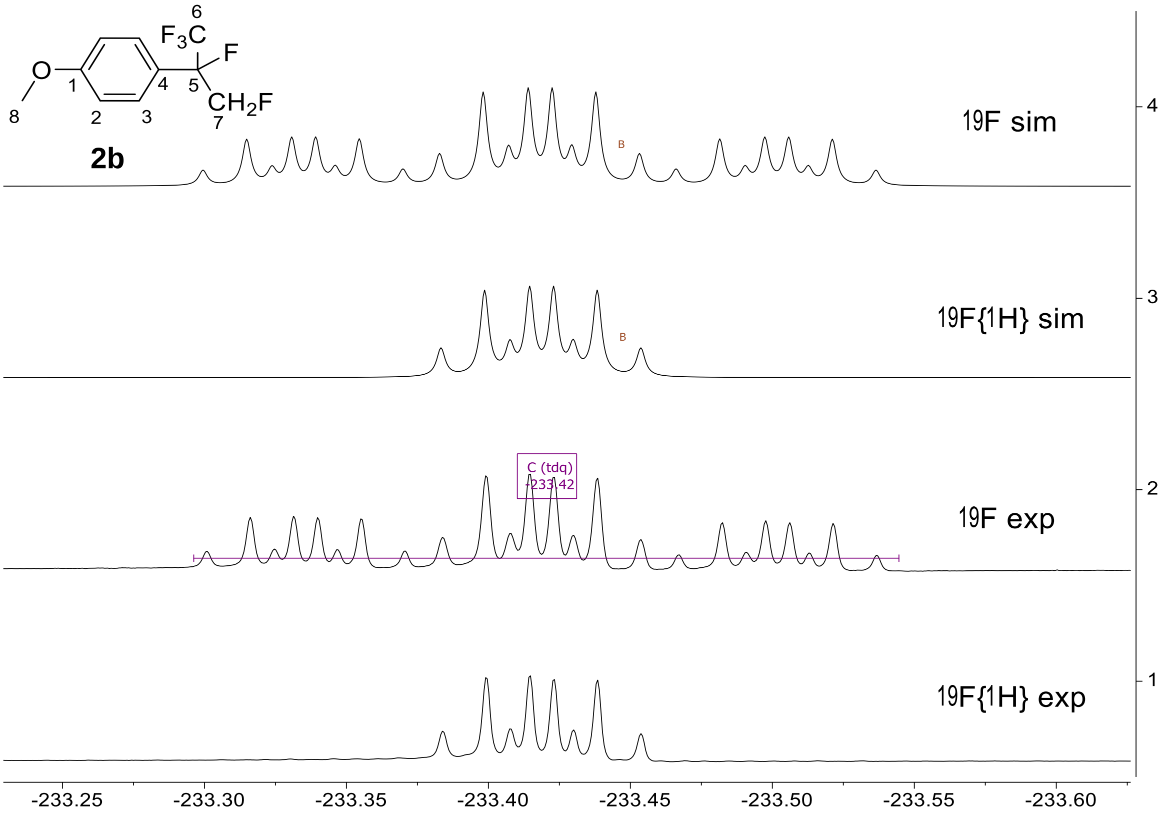

Figure S4. Stacked ^{19}F spectra of the resonance of the CF group (F-C7) of compound **2b**. Top) data of the simulation (sim); bottom) data of the experimental measurement (exp).

The fluorine at C7 is neighboured by 4 fluorine atoms and two diastereotopic protons. Therefore, the observed multiplicity is an unresolved doublet of doublet of doublet of quartet (dddq).

Regioselectivity Ratio

Geminal product **3a** was isolated and fully characterised to provide conclusive proof of regioselectivity assignment. Subsequent ratios were determined by analogy.

1-Chloro-4-(2,2,3,3,3-pentafluoropropyl)benzene (3a)

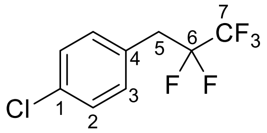

Compound **3a** was prepared according to a modification of General Procedure **D** with an amine:HF ratio of 1:7.5 using 1-chloro-4-(3,3,3-trifluoroprop-1-en-2-yl)benzene (**1a**) (103.3 mg, 0.5 mmol, 1.0 eq.) and *para*-iodotoluene (21.8 mg, 0.1 mmol) as a catalyst. The reaction was run two times this scale and were combined for the purification. The crude mixture was purified by column chromatography (*n*-pentane) to yield the title compound as a colourless oil (51 mg, 0.21 mmol, 21%).

R_f = 0.62 (*n*-pentane).

FT-IR ($\tilde{\nu}$ = cm^{-1}): 2923 (w), 1494 (w), 1316 (w), 1243 (w), 1196 (s), 1098 (w), 1031 (m), 798 (w), 787 (w), 739 (w).

^{1}H NMR (500 MHz, $CDCl_3$): δ [ppm] = 7.37 – 7.33 (m, 2H, H-C2), 7.24 – 7.20 (m, 2H, H-C3), 3.30 (t, $^2J_{HF}$ = 18.1 Hz, 2H, H-C5).

^{13}C{^{1}H,^{19}F} NMR (151 MHz, $CDCl_3$) δ [ppm] = 134.6 (C1), 132.1 (C3), 129.0 (C2), 127.7 (C4), 119.3 (C7), 114.4 (C6), 36.6 (C5).

^{19}F NMR (470 MHz, $CDCl_3$): δ [ppm] = -84.7 (m, F-C7), -117.0 (t, $^3J_{HF}$ = 18.1 Hz, 2F, F-C6).

^{19}F{^{1}H} NMR (470 MHz, $CDCl_3$): δ [ppm] = -84.7 (s, 3F, F-C7), -117.0 (s, 2F, F-C6).

GC-EI-MS: (*m/z*) requires: [($C_9H_6ClF_5$)] = 244.0078, (*m/z*) found: [($C_9H_6ClF_5$)] = 244.0075.

1-Chloro-4-(1,1,1,2,3-pentafluoropropan-2-yl)benzene (2a)

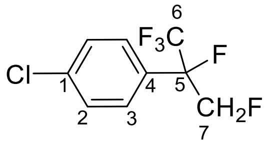

Compound **2a** was prepared according to the General Procedure **D** with an amine:HF ratio of 1:7.5 using 1-chloro-4-(3,3,3-trifluoroprop-1-en-2-yl)benzene (**1a**) (41 mg, 0.2 mmol, 1.0 eq.). After workup, the crude mixture was analysed by ^{19}F NMR (>95% NMR yield, 2.0:1 *vic:gem*). Purification by column chromatography (*n*-pentane) yielded the title compound as a colourless oil (22 mg, 0.09 mmol, 45%).

R_f = 0.53 (*n*-pentane).

FT-IR ($\tilde{\nu}$ = cm^{-1}): 1603 (w), 1497 (w), 1410 (w), 1395 (w), 1327 (w), 1298 (m), 1276 (w), 1258 (w), 1239 (w); 1177 (s), 1152 (s), 1096 (s), 1053 (m), 1016 (m), 973 (m), 957 (m), 909 (m), 859 (w), 824 (s), 764 (w), 733 (m), 720 (w), 705 (w).

^{1}H NMR (599 MHz, $CDCl_3$): δ [ppm] = 7.46 (broad s, 4H, H-C2, H-C3), 4.93 (ddd, $^2J_{HF}$ = 47.1 Hz, $^3J_{HF}$ = 19.2 Hz, $^3J_{HH}$ = 11.0 Hz, 1H, H^a-C7), 4.87 (dddq, $^2J_{HF}$ = 46.4 Hz, $^3J_{HF}$ = 21.8 Hz, $^3J_{HH}$ = 11.1 Hz, $^4J_{HF}$ = 1.0 Hz, 1H, H^b-C7).

$^{13}C\{^1H\}$ NMR (151 MHz, $CDCl_3$): δ [ppm] = 136.7 (d, $^5J_{CF}$ = 1.5 Hz, C1), 129.3 (d, $^4J_{CF}$ = 1.7 Hz, C2), 129.0 (dd, $^2J_{CF}$ = 21.6 Hz, $^3J_{CF}$ = 2.9 Hz, C4), 127.3 (dq, $^3J_{CF}$ = 9.6 Hz, $^4J_{CF}$ = 1.3 Hz, C3), 122.2 (qdd, $^1J_{CF}$ = 285.3 Hz, $^2J_{CF}$ = 29.6 Hz, $^3J_{CF}$ = 4.3 Hz, C6), 93.6 (dqd, $^1J_{CF}$ = 192.4 Hz, $^2J_{CF}$ = 31.5 Hz, $^2J_{CF}$ = 19.2 Hz, C5), 81.4 (ddq, $^1J_{CF}$ = 185.8 Hz, $^2J_{CF}$ = 24.3 Hz, $^3J_{CF}$ = 1.3 Hz, C7).

^{19}F NMR (376 MHz, $CDCl_3$): δ [ppm] = -77.5 (ddd, $^3J_{FF}$ = 8.0 Hz, $^3J_{FF}$ = 7.0 Hz, $^4J_{HF}$ = 1.0 Hz, 3F, F-C6), -179.0 (dddq, $^3J_{HF}$ = 21.8 Hz, $^3J_{HF}$ = 19.2 Hz, $^3J_{FF}$ = 14.1 Hz, $^3J_{FF}$ = 7.0 Hz, 1F, F-C5), -234.3 (dddq, $^2J_{HF}$ = 47.1 Hz, $^2J_{HF}$ = 46.4 Hz, $^3J_{FF}$ = 13.3 Hz, $^4J_{FF}$ = 8.0 Hz, 1F, F-C7).

$^{19}F\{^1H\}$ NMR (376 MHz, $CDCl_3$): δ [ppm] = -77.5 (dd, $^3J_{FF}$ = 8.0 Hz, $^3J_{FF}$ = 7.0 Hz, 3F, F-C6), -179.0 (dq, $^3J_{FF}$ = 14.1, 7.0 Hz, 1F, F-C5), -234.3 (dq, $^3J_{FF}$ = 13.2 Hz, $^4J_{FF}$ = 8.0 Hz, 1F, F-C7).

EI-MS: (*m/z*) requires: $[(C_9H_6ClF_5)]^+$ = 244.0073, (*m/z*) found: $[(C_9H_6ClF_5)]^+$ = 244.0082.

1-Methoxy-4-(1,1,1,2,3-pentafluoropropan-2-yl)benzene (2b)

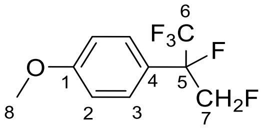

Compound **2b** was prepared according to the General Procedure **D** with an amine:HF ratio of 1:4.5 using 1-methoxy-4-(3,3,3-trifluoroprop-1-en-2-yl)benzene (**1b**) (40 mg, 0.2 mmol, 1.0 eq.). After workup, the crude mixture was analysed by ^{19}F NMR (87% NMR yield, >20:1 *vic:gem*). Purification by column chromatography (*n*-pentane) yielded the title compound as a colourless oil (14 mg, 0.06 mmol, 30%).

R_f = 0.51 (2% Et_2O in *n*-pentane).

FT-IR ($\tilde{\nu}$ = cm^{-1}): 2917 (w), 1615 (w), 1518 (m), 1465 (w), 1304 (w), 1259 (m), 1180 (m), 1010 (w), 1033 (w), 957 (w), 831 (m), 739 (w).

^1H NMR (500 MHz, $CDCl_3$): δ [ppm] = 7.42 (m, 2H, H-C3), 6.98 (m, 2H, H-C2), 4.93 (ddd, $^2J_{HF}$ = 47.3 Hz, $^3J_{HF}$ = 21.0 Hz, $^2J_{HH}$ = 11.4 Hz, 1H, H^a-C7), 4.83 (dddq, $^2J_{HF}$ = 46.5 Hz, $^3J_{HF}$ = 21.4 Hz, $^2J_{HH}$ = 11.1 Hz, $^4J_{HF}$ = 1.3 Hz, 1H, H^b-C7), 3.84 (s, 3H, H-C8).

$^{13}C\{^1H\}$ NMR (151 MHz, $CDCl_3$): δ [ppm] = 160.8 (d, $^5J_{CF}$ = 1.2 Hz, C1), 127.1 (dq, $^3J_{CF}$ = 9.6 Hz, $^3J_{CF}$ = 1.1 Hz, C3), 122.3 (qdd, $^1J_{CF}$ = 285.1 Hz, $^2J_{CF}$ = 30.0 Hz, $^3J_{CF}$ = 4.4 Hz, C6), 122.1 (dd, $^2J_{CF}$ = 21.6 Hz, $^3J_{CF}$ = 3.2 Hz, C4), 114.2 (d, $^4J_{CF}$ = 1.6 Hz, C2), 93.6 (dqd, $^1J_{CF}$ = 190.8 Hz, $^2J_{CF}$ = 31.2 Hz, $^2J_{CF}$ = 18.5 Hz, C5), 81.4 (ddq, $^1J_{CF}$ = 185.7 Hz, $^2J_{CF}$ = 24.1 Hz, $^3J_{CF}$ = 1.4 Hz, C7), 55.3 (C8).

^{19}F NMR (564 MHz, $CDCl_3$): δ [ppm] = -77.6 (ddd, $^4J_{FF}$ = 8.6 Hz, $^3J_{FF}$ = 7.1 Hz, $^4J_{HF}$ = 1.3 Hz, 3F, F-C6), -178.4 (dddq, $^3J_{HF}$ = 21.4 Hz, $^3J_{HF}$ = 21.0 Hz, $^3J_{FF}$ = 14.0 Hz, $^3J_{FF}$ = 7.1 Hz, 1F, F-C5), -233.4 (dddq, $^2J_{HF}$ = 47.3 Hz, $^2J_{HF}$ = 46.5 Hz, $^3J_{FF}$ = 13.4 Hz, $^4J_{FF}$ = 8.6 Hz, 1F, F-C7).

$^{19}F\{^1H\}$ NMR (376 MHz, $CDCl_3$): δ [ppm] = -77.6 (dd, $^4J_{FF}$ = 8.6 Hz, $^3J_{FF}$ = 7.1 Hz, F-C6), -178.4 (dq, $^3J_{FF}$ = 14.0 Hz, $^3J_{FF}$ = 7.1 Hz, F-C5), -233.4 (dq, $^3J_{FF}$ = 13.3 Hz, $^4J_{FF}$ = 8.6 Hz, F-C7).

GC-EI-MS: (*m/z*) requires: $[(C_{10}H_9OF_5)]$ = 240.05736, (*m/z*) found: $[(C_{10}H_9OF_5)]$ = 240.05640.

2-Methoxy-1,3-dimethyl-5-(1,1,1,2,3-pentafluoropropan-2-yl)benzene (2c)

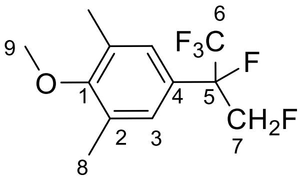

Compound **2c** was prepared according to the General Procedure **D** with an amine: HF ratio of 1:4.5 and an extended reaction time of 48 h using 2-methoxy-1,3-dimethyl-5-(3,3,3-trifluoroprop-1-en-2-yl)benzene (**1c**) (46 mg, 0.2 mmol, 1.0 eq.). After workup, the crude mixture was analysed by ^{19}F NMR (55% NMR yield, >20:1 *vic:gem*). Purification by column chromatography (2% Et_2O in *n*-pentane) yielded the title compound as a yellow oil (24mg, 0.09 mmol, 45%).

R_f = 0.57 (5% Et_2O in *n*-pentane)

FT-IR ($\tilde{\nu}$ = cm^{-1}): 2961 (w), 1489 (m), 1454 (w), 1380 (w), 1327 (m), 1304 (m), 1238 (m), 1206 (m), 1179 (s), 1153 (s), 1101 (m), 1065 (w), 1039 (w), 1011 (m), 944 (m), 911 (w), 874 (w), 852 (m), 832 (m), 766 (m), 753 (m), 729 (m), 715 (m), 703 (m).

^{1}H NMR (599 MHz, $CDCl_3$): δ [ppm] = 7.13 (s, 2H, H-C3), 4.91 (ddd, $^2J_{HF}$ = 47.1 Hz, $^3J_{HF}$ = 21.4 Hz, $^3J_{HH}$ = 11.2 Hz, 1H, H^a-C7), 4.81 (dddq, $^2J_{HF}$ = 46.6 Hz, $^3J_{HF}$ = 21.4 Hz, $^3J_{HH}$ = 11.3 Hz, $^4J_{HF}$ = 1.0 Hz, 1H, H^b-C7), 3.74 (s, 3H, H-C9), 2.32 (s, 6H, H-C8).

^{13}C{^{1}H} NMR (151 MHz, $CDCl_3$): δ [ppm] = 158.5 (d, $^5J_{CF}$ = 1.4 Hz, C1), 131.8 (d, $^4J_{CF}$ = 1.6 Hz, C2), 126.2 (d, $^4J_{CF}$ = 9.6 Hz, C3), 125.4 (dd, $^3J_{CF}$ = 21.1 Hz, $^4J_{CF}$ = 3.2 Hz, C4), 122.5 (qdd, $^1J_{CF}$ = 285.2 Hz, $^2J_{CF}$ = 30.0 Hz, $^3J_{CF}$ = 3.7 Hz, C6), 93.7 (dqd, $^1J_{CF}$ = 191.2 Hz, $^2J_{CF}$ = 31.2 Hz, $^2J_{CF}$ = 18.5 Hz, C5), 81.8 (ddq, $^1J_{CF}$ = 185.8 Hz, $^2J_{CF}$ = 23.7 Hz, $^3J_{CF}$ = 1.4 Hz, C7), 59.8 (C9), 16.5 (C8).

^{19}F NMR (376 MHz, $CDCl_3$): δ [ppm] = -77.3 (ddd, $^4J_{FF}$ = 8.7 Hz, $^3J_{FF}$ = 7.0 Hz, $^4J_{HF}$ = 1.0 Hz, 3F, F-C6), -179.1 (dddq, $^3J_{HF}$ = 21.4 Hz, $^3J_{HF}$ = 21.4 Hz, $^3J_{FF}$ = 13.8, 6.9 Hz, 1F, F-C5), -233.2 (dddq, $^2J_{HF}$ = 47.1 Hz, $^2J_{HF}$ = 46.6 Hz, $^3J_{FF}$ = 13.2 Hz, $^4J_{FF}$ = 8.7 Hz, 1F, F-C7).

^{19}F{^{1}H} NMR (376 MHz, $CDCl_3$): δ [ppm] = -77.26 (dd, $^4J_{FF}$ = 8.7 Hz, $^3J_{FF}$ = 7.0 Hz, 3F, F-C6), -179.06 (dq, $^3J_{FF}$ = 13.7 Hz, $^3J_{FF}$ = 6.9 Hz, 1F, F-C5), -233.16 (dq, $^3J_{FF}$ = 13.1 Hz, $^4J_{FF}$ = 8.7 Hz, 1F, F-C7).

EI-MS: (*m/z*) requires: $[(C_{12}H_{13}OF_5)]^+$ = 268.0881, (*m/z*) found: $[(C_{12}H_{13}OF_5)]^+$ = 268.0886.

1-Bromo-4-((4-(1,1,1,2,3-pentafluoropropan-2-yl)phenoxy)methyl)benzene (2d)

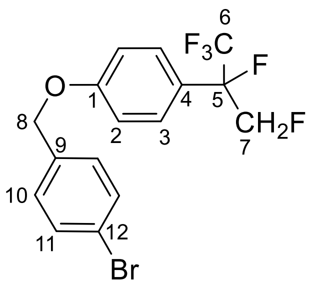

Compound **2d** was prepared according to the General Procedure **D** with an amine:HF ratio of 1:4.5 and an extended reaction time of 48 h using 1-bromo-4-((4-(3,3,3-trifluoroprop-1-en-2-yl)phenoxy)methyl)benzene (**1d**) (71 mg, 0.2 mmol, 1.0 eq.). After workup, the crude mixture was analysed by ^{19}F NMR (64% NMR yield, >20:1 *vic:gem*). Purification by column chromatography (*n*-pentane) yielded the title compound as colourless oil (41 mg, 0.10 mmol, 50%).

R_f = 0.41 (*n*-pentane).

FT-IR ($\tilde{\nu}$ = cm^{-1}): 1613 (w), 1515 (m), 1257 (w), 1182 (s), 1099 (w), 1012 (w), 957 (w), 829 (w), 808 (w).

^{1}H NMR (599 MHz, CDCl$_3$): δ [ppm] = 7.53(m, 2H, H-C11), 7.43 (m, 2H, H-C13), 7.31 (m, 2H, H-C10), 7.03 (m, 2H, H-C2), 5.04 (s, 2H, H-C8), 4.93 (ddd, $^2J_{FH}$ = 47.1 Hz, $^3J_{FH}$ = 20.7 Hz, $^2J_{HH}$ = 11.2 Hz, 1H, H^a-C7), 4.84 (dddq, $^2J_{FH}$ = 46.6 Hz, $^3J_{FH}$ = 21.6 Hz, $^2J_{HH}$ = 11.2 Hz, $^4J_{FH}$ = 1.3 Hz, 1H H^b-C7).

^{13}C{^{1}H} NMR (151 MHz, CDCl$_3$): δ [ppm] = 159.9 (d, $^5J_{CF}$ = 1.3 Hz, C1), 135.6 (C9), 132.0 (C11), 129.2 (C10), 127.3 (dt, $^3J_{CF}$ = 9.6 Hz, $^3J_{CF}$ = 1.2 Hz, C3), 122.8 (dd, $^2J_{CF}$ = 21.7, $^3J_{CF}$ = 3.2 Hz, C4), 122.4 (qdd, $^1J_{CF}$ = 285.4 Hz, $^2J_{CF}$ = 29.1 Hz, $^3J_{CF}$ = 3.8 Hz, C6), 122.3 (C12), 115.2 (d, $^4J_{CF}$ = 1.6 Hz, C2), 93.8 (ddd, $^1J_{CF}$ = 190.9 Hz, $^2J_{CF}$ = 31.3 Hz, $^2J_{CF}$ = 18.6 Hz, C5), 81.7 (ddq, $^1J_{CF}$ = 185.5 Hz, $^2J_{CF}$ = 24.1 Hz, $^3J_{CF}$ = 1.0 Hz, C7), 69.5 (C8).

^{19}F NMR (564 MHz, CDCl$_3$): δ [ppm] = -77.6 (ddd, $^4J_{FF}$ = 8.5 Hz, $^3J_{FF}$ = 7.1 Hz, $^4J_{FH}$ = 1.3 Hz, 3F, F-C6), -178.4 (dddq, $^3J_{FH}$ = 21.6 Hz, $^3J_{FH}$ = 20.7 Hz, $^3J_{FF}$ = 14.1 Hz, $^3J_{FF}$ = 7.2 Hz, 1F, F-C5), -233.5 (dddq, $^2J_{FH}$ = 47.1 Hz, $^2J_{FH}$ = 46.6 Hz, $^3J_{FF}$ = 13.3 Hz, $^4J_{FF}$ = 8.5 Hz, 1F, F-C7).

^{19}F{^{1}H} NMR (564 MHz, CDCl$_3$): δ [ppm] = -77.6 (dd, $^4J_{FF}$ = 8.5 Hz, $^3J_{FF}$ = 7.1 Hz, 3F, F-C6), -178.4 (dq, $^3J_{FF}$ = 14.1 Hz, $^3J_{FF}$ = 7.2 Hz, 1F, F-C5), -233.5 (dq, $^3J_{FF}$ = 13.3 Hz, $^4J_{FF}$ = 8.5 Hz, 1F, F-C7).

GC-EI-MS: (*m/z*) requires: [($C_{16}H_{12}F_5OBr$)] = 393.99862, (*m/z*) found: [($C_{16}H_{12}F_5OBr$)] = 393.99864.

1-Methyl-4-(1,1,1,2,3-pentafluoropropan-2-yl)benzene (2e)

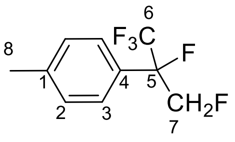

Compound **2e** was prepared according to the General Procedure **D** with an amine:HF ratio of 1:6 using 1-methyl-4-(3,3,3-trifluoroprop-1-en-2-yl)benzene (**1e**) (37 mg, 0.2 mmol, 1.0 eq.). After workup, the crude mixture was analysed by ^{19}F NMR (>95% NMR yield, 12.0:1 *vic:gem*). Purification by preparative thin layer chromatography (*n*-pentane) yielded the title compound as a colourless oil (17 mg, 0.08 mmol, 40%).

R_f = 0.68 (*n*-pentane).

FT-IR ($\tilde{\nu}$ = cm^{-1}): 1177 (m), 1100 (w), 1053 (w), 957 (w), 909 (w), 813 (m), 734 (m), 669 (w).

^{1}H NMR (599 MHz, CDCl$_3$): δ [ppm] = 7.39 (m, 2H, H-C3), 7.28 (m, 2H, H-C2), 4.94 (ddd, $^2J_{HF}$ = 47.2 Hz, $^3J_{HF}$ = 21.3 Hz, $^2J_{HH}$ = 11.2 Hz, 1H, H^a-C7), 4.84 (dddq, $^2J_{HF}$ = 46.6 Hz, $^3J_{HF}$ = 21.4 Hz, $^2J_{HH}$ = 11.2 Hz, $^4J_{HF}$ = 1.2 Hz, 1H, H^b-C7), 2.39 (s, 3H, H-C8).

^{13}C{^{1}H} NMR (151 MHz, CDCl$_3$): δ [ppm] = 140.3 (s), C1), 129.7 (d, $^4J_{CF}$ = 1.7 Hz,, C2), 127.5 (dd, $^2J_{CF}$ = 21.2 Hz, $^3J_{CF}$ = 3.3 Hz, C4), 125.6 (dq, $^3J_{CF}$ = 9.5 Hz, $^4J_{CF}$ = 1.2 Hz, C3), 122.5 (qdd, $^1J_{CF}$ = 285.2 Hz, $^2J_{CF}$ = 30.0 Hz, $^3J_{CF}$ = 3.7 Hz, C6), 93.9 (ddd*, $^1J_{CF}$ = 191.2 Hz, $^2J_{CF}$ = 31.2 Hz, $^2J_{CF}$ = 18.6 Hz, C5), 81.8 (ddq, $^1J_{CF}$ = 185.5 Hz, $^2J_{CF}$ = 23.7 Hz, $^3J_{CF}$ = 1.3 Hz, C7), 21.3 (1C, C8).

^{19}F NMR (564 MHz, CDCl$_3$): δ [ppm] = -77.4 (ddd, $^4J_{FF}$ = 8.7 Hz, $^3J_{FF}$ = 6.9 Hz, $^4J_{HF}$ = 1.2 Hz, 3F, F-C6), -179.6 (dddq, $^3J_{HF}$ = 21.4 Hz, $^3J_{HF}$ = 21.3 Hz, $^3J_{FF}$ = 13.7 Hz, $^3J_{FF}$ = 7.0 Hz, 1F, F-C5), -233.5 (dddq, $^2J_{HF}$ = 47.2 Hz, $^2J_{HF}$ = 46.6 Hz, $^3J_{FF}$ = 13.1 Hz, $^4J_{FF}$ = 8.6 Hz, 1F, F-C7).

^{19}F {^{1}H} NMR (564 MHz, CDCl$_3$): δ [ppm] = -77.4 (dd, $^4J_{FF}$ = 8.7 Hz, $^3J_{FF}$ = 6.9 Hz, 3F, F-C6), -179.6 (dq, $^3J_{FF}$ = 13.7 Hz, $^3J_{FF}$ = 7.0 Hz, 1F, F-C6), -233.5 (dq, $^3J_{FF}$ = 13.1 Hz, $^4J_{FF}$ = 8.6 Hz, 1F, F-C7).

GC-EI-MS: (*m/z*) requires: [($C_9H_6ClF_5$)] = 244.0078, (*m/z*) found: [($C_9H_6ClF_5$)] = 244.0077.

*the q for the $^2J_{CF}$ could not be detected.

1-Isopropyl-4-(1,1,1,2,3-pentafluoropropan-2-yl)benzene (2f)

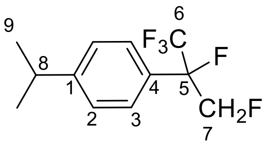

Compound **2f** was prepared according to the General Procedure **D** with an amine:HF ratio of 1:6 and an extended reaction time of 48 h using 1-isopropyl-4-(3,3,3-trifluoroprop-1-en-2-yl)benzene (**1f**) (43 mg, 0.2 mmol, 1.0 eq.). After workup, the crude mixture was analysed by ^{19}F NMR (>95% NMR yield, 11.4:1 *vic:gem*). Purification by preparative thin layer chromatography (*n*-pentane) yielded the title compound as a colourless oil (18 mg, 0.07 mmol, 35%).

R_f = 0.73 (*n*-pentane).

FT-IR ($\tilde{\nu}$ = cm^{-1}): 2965 (m), 1518 (w), 1463 (m), 1420 (w), 1328 (w), 1296 (m), 1180 (s), 1154 (m), 1100 (s), 1051 (s), 1020 (w), 958 (s), 908 (m), 830 (s), 747 (w), 720 (m), 667 (m).

^{1}H NMR (500 MHz, CDCl$_3$): δ [ppm] = 7.42 (m, 2H, H-C3), 7.32 (m, 2H, H-C2), 4.95 (ddd, $^2J_{HF}$ = 47.1 Hz, $^3J_{HF}$ = 21.5 Hz, $^3J_{HH}$ 11.2 Hz, 1H, H^a-C7), 4.84 (dddq, $^2J_{HF}$ = 46.7 Hz, $^3J_{HF}$ = 21.1 Hz, $^2J_{HH}$ = 11.3 Hz, $^4J_{HF}$ = 1.1 Hz, 1H, H^b-C7), 2.95 (h, $^3J_{HH}$ = 6.9 Hz, 1H, H-C8), 1.27 (d, $^3J_{HH}$ = 6.9 Hz, 3H, H-C9).

^{13}C{^{1}H} NMR (126 MHz, CDCl$_3$): δ [ppm] = 151.1 (d, $^5J_{CF}$ = 1.1 Hz, C1), 127.8 (dd, $^2J_{CF}$ = 21.3 Hz, $^3J_{CF}$ = 3.2 Hz, C4), 127.1 (d, $^4J_{CF}$ = 1.7 Hz, C2), 125.7 (dp, $^3J_{CF}$ = 9.6 Hz, $^3J_{CF}$ = 1.2 Hz, C3), 122.5 (qdd, $^1J_{CF}$ = 285.1 Hz, $^2J_{CF}$ = 30.0 Hz, $^3J_{CF}$ = 3.6 Hz, C6), 93.9 (dqd, $^1J_{CF}$ = 191.0 Hz, $^2J_{CF}$ = 31.2 Hz, $^2J_{CF}$ = 18.5 Hz, C5), 81.9 (ddq, $^1J_{CF}$ = 185.4 Hz, $^2J_{CF}$ = 23.7 Hz, $^3J_{CF}$ = 1.1 Hz, C7), 34.0 (C8), 23.9 (C9).

^{19}F NMR (470 MHz, CDCl$_3$): δ [ppm] = -77.3 (ddd, $^4J_{FF}$ = 8.8 Hz, $^3J_{FF}$ = 6.9 Hz, $^4J_{HF}$ = 1.1 Hz, 3F, F-C6), -179.5 (dddq, $^3J_{HF}$ = 21.5 Hz, $^3J_{HF}$ = 21.1 Hz, $^3J_{FF}$ = 13.7 Hz, $^3J_{FF}$ = 6.9 Hz, 1F, F-C5), -233.3 (dddq, $^2J_{HF}$ = 47.1 Hz, $^2J_{HF}$ = 46.7 Hz, $^3J_{FF}$ = 13.0 Hz, $^4J_{FF}$ = 8.8 Hz, 1F, F-C7).

^{19}F {^{1}H} NMR (470 MHz, CDCl$_3$): δ [ppm] = -77.3 (dd, $^4J_{FF}$ = 8.8 Hz, $^3J_{FF}$ = 7.0 Hz, 3F, F-C6), -179.5 (dq, $^3J_{FF}$ = 13.7 Hz, $^3J_{FF}$ = 6.9 Hz, 1F, F-C5), -233.3 (dq, $^3J_{FF}$ = 13.1 Hz, $^4J_{FF}$ = 8.8 Hz, 1F, F-C7).

GC-EI-MS: (*m/z*) requires: [($C_{12}H_{13}F_5$)] = 252.09319, (*m/z*) found: [($C_{12}H_{13}F_5$)] = 252.09319.

1-(Tert-butyl)-4-(1,1,1,2,3-pentafluoropropan-2-yl)benzene (2g)

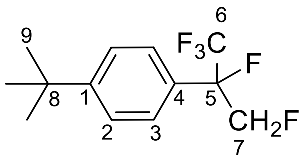

Compound **2g** was prepared according to the General Procedure **D** with an amine:HF ratio of 1:6 using 1-tert-butyl-4-(3,3,3-trifluoroprop-1-en-2-yl)benzene (**1g**) (46 mg, 0.2 mmol, 1.0 eq.). After workup, the crude mixture was analysed by ^{19}F NMR (>95% NMR yield, 11.4:1 *vic:gem*). Purification by preparative thin layer chromatography (*n*-pentane) yielded the title compound as a colourless oil (26 mg, 0.12 mmol, 60%).

R*f* = 0.72 (*n*-pentane).

FT-IR ($\tilde{\nu}$ = cm^{-1}): 2965 (m), 2927 (w), 2870 (w), 1464 (w), 1366 (w), 1328 (w), 1301 (w), 1285 (w), 1244 (w), 1197 (s), 1179 (s), 1113 (m), 1100 (m), 1055(m), 1019 (w), 973 (m), 959 (m), 911 (w), 832 (m), 722 (w), 714 (m), 699 (w).

^{1}H NMR (500 MHz, $CDCl_3$): δ [ppm] = 7.49 (m, 2H, H-C3), 7.43 (m, 2H, H-C2), 4.95 (ddd, $^{2}J_{HF}$ = 47.0 Hz, $^{3}J_{HF}$ = 21.4 Hz, $^{3}J_{HH}$ = 11.1 Hz, 1H, H^{a}-C7), 4.85 (dddq, $^{2}J_{HF}$ = 46.6 Hz, $^{3}J_{HF}$ = 20.9 Hz, $^{3}J_{HH}$ = 11.2, $^{4}J_{HF}$ 1.3 Hz, 1H, H^{b}-C7), 1.35 (s, 9H, H-C9).

^{13}C{^{1}H} NMR (151 MHz, $CDCl_3$): δ [ppm] = 153.4 (d, $^{5}J_{CF}$ = 1.1 Hz, C1), 127.4 (dd, $^{2}J_{CF}$ = 21.3 Hz, $^{3}J_{CF}$ = 3.2 Hz, C4), 127.1 (d, $^{4}J_{CF}$ = 1.7 Hz, C2), 125.7 (d), $^{3}J_{CF}$ = 9.6 Hz, C3), 122.5 (qdd, $^{1}J_{CF}$ = 285.1 Hz, $^{2}J_{CF}$ = 30.0 Hz, $^{3}J_{CF}$ = 3.6 Hz, C6), 93.9 (dqd, $^{1}J_{CF}$ = 191.0 Hz, $^{2}J_{CF}$ = 31.3 Hz, $^{2}J_{CF}$ = 18.5 Hz, C5), 81.8 (ddq, $^{1}J_{CF}$ = 185.4 Hz, $^{2}J_{CF}$ = 23.7 Hz, $^{3}J_{CF}$ = 1.1 Hz, C7), 34.9 (C8), 31.3 (C9).

^{19}F NMR (564 MHz, $CDCl_3$): δ [ppm] = -77.3 (ddd, $^{4}J_{FF}$ = 8.8 Hz, $^{3}J_{FF}$ = 7.0 Hz, 3F, F-C6), -179.6 (dddq, $^{3}J_{HF}$ = 21.4 Hz, $^{3}J_{HF}$ = 20.9 Hz, $^{3}J_{FF}$ = 13.7 Hz, $^{3}J_{FF}$ = 6.9 Hz, 1F, F-C5), -233.3 (dddq, $^{2}J_{HF}$ = 47.0 Hz, $^{2}J_{HF}$ = 46.6 Hz, $^{3}J_{FF}$ = 13.0 Hz, $^{4}J_{FF}$ = 8.8 Hz, 1F, F-C7).

^{19}F{^{1}H} NMR (376 MHz, $CDCl_3$): δ [ppm] = -77.3 (dd, $^{4}J_{FF}$ = 8.8 Hz, $^{3}J_{FF}$ = 7.0 Hz, 3F, F-C6), -179.6 (dq, $^{3}J_{FF}$ = 13.2 Hz, $^{3}J_{FF}$ = 6.9 Hz, 1F, F-C5), -233.3 (dq, $^{3}J_{FF}$ = 13.1 Hz, $^{4}J_{FF}$ = 8.8 Hz, 1F, F-C7).

EI-MS: (*m/z*) requires: $[(C_{13}H_{15}F_5)]^+$ = 266.1088, (*m/z*) found: $[(C_{13}H_{15}F_5)]^+$ = 266.1094

1-Cyclopropyl-4-(1,1,1,2,3-pentafluoropropan-2-yl)benzene (2h)

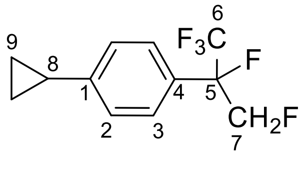

Compound **2h** was prepared according to the General Procedure **D** with an amine:HF ratio of 1:6 using 1-cyclopropyl-4-(3,3,3-trifluoroprop-1-en-2-yl)benzene (**1h**) (42 mg, 0.2 mmol, 1.0 eq). After workup, the crude mixture was analysed by ^{19}F NMR (56% NMR yield, >20:1 *vic:gem*). Purification by preparative thin layer chromatography (*n*-pentane) yielded the title compound as acolourless oil (12 mg, 0.05 mmol, 25%).

Large scale synthesis: Compound **2h** was prepared according to the General Procedure **D** with an amine:HF ratio of 1:6 using 1-cyclopropyl-4-(3,3,3-trifluoroprop-1-en-2-yl)benzene (**1h**) (212 mg,

1.0 mmol, 1.0 eq). Purification by column chromatography (*n*-pentane) yielded the title compound as a colourless oil (115 mg, 0.46 mmol, 46%).

R*f* = 0.66 (*n*-pentane).

FT-IR ($\tilde{\nu}$ = cm^{-1}): 3010 (w), 1617 (m), 1520 (m), 1463 (m), 1421 (m), 1393 (m), 1329 (s), 1285 (s), 1172 (s), 1153 (s) 1100 (s), 1047 (s), 1019 (s), 957 (s), 903 (s), 821 (s), 764 (m), 741 (s), 716 (s), 622 (s).

^{1}H NMR (599 MHz, $CDCl_3$): δ [ppm] = 7.38 (m, 2H, H-C3), 7.15 (m, 2H, H-C2), 4.93 (dddd, $^2J_{HF}$ = 47.1 Hz, $^3J_{HF}$ = 21.3 Hz, $^3J_{HH}$ = 11.2 Hz, $^4J_{HF}$ = 0.8 Hz, 1H, H^a-C7), 4.82 (dddq, $^2J_{HF}$ = 46.7 Hz, $^3J_{HF}$ = 21.2 Hz, $^3J_{HH}$ = 11.2, $^4J_{HF}$ 1.2 Hz, 1H, H^b-C7), 1.93 (tt, $^3J_{HH}$ = 8.4 Hz, $^3J_{HH}$ = 5.0 Hz, 1H, H-C8), 1.05 – 0.98 (m, 2H, H^a-C9), 0.76 – 0.70 (m, 2H, H^b-C9).

^{13}C{^{1}H} NMR (151 MHz, $CDCl_3$): δ [ppm] = 146.6 (d, $^5J_{CF}$ = 1.1 Hz, C1), 127.3 (dd, $^2J_{CF}$ = 21.3 Hz, $^3J_{CF}$ = 3.2 Hz, C4), 126.1 (d, $^4J_{CF}$ = 1.7 Hz, C2), 125.6 (dt, $^3J_{CF}$ = 9.5 Hz, $^4J_{CF}$ = 1.3 Hz, C3), 122.5 (qdd, $^1J_{CF}$ = 285.1 Hz, $^2J_{CF}$ = 30.1 Hz, $^3J_{CF}$ = 3.7 Hz, C6), 93.9 (dqd, $^1J_{CF}$ = 191.2, $^2J_{CF}$ = 31.4 Hz, $^2J_{CF}$ = 18.8 Hz, C5), 81.7 (ddq, $^1J_{CF}$ = 185.7 Hz, $^2J_{CF}$ = 23.6 Hz, $^3J_{CF}$ = 0.9 Hz, C7), 15.3 (C8), 9.8 (C9).

^{19}F NMR (564 MHz, $CDCl_3$): δ [ppm] = -77.4 (ddd, $^4J_{FF}$ = 8.7 Hz, $^3J_{FF}$ = 6.9 Hz, 3F, F-C6), -179.4 (dddq, $^3J_{HF}$ = 21.3 Hz, $^3J_{HF}$ = 21.2 Hz, $^3J_{FF}$ = 13.9 Hz, $^3J_{FF}$ = 7.0 Hz, 1F, F-C5), -233.4 (dddq, $^2J_{HF}$ = 47.1 Hz, $^2J_{HF}$ = 46.7 Hz, $^3J_{FF}$ = 13.2 Hz, $^4J_{FF}$ = 8.7 Hz, 1F, F-C7).

^{19}F{^{1}H} NMR (564 MHz, $CDCl_3$): δ [ppm] = -77.4 (dd, $^4J_{FF}$ = 8.7 Hz, $^3J_{FF}$ = 6.9 Hz, 3F, F-C6), -179.6 (dq, $^3J_{FF}$ = 13.9 Hz, $^3J_{FF}$ = 7.0 Hz, 1F, F-C5), -233.4 (dq, $^3J_{FF}$ = 13.1 Hz, $^4J_{FF}$ = 8.7 Hz, 1F, F-C7).

GC-EI-MS: (*m/z*) requires: [($C_{12}H_{11}F_5$)] = 250.07754 (*m/z*) found: [($C_{12}H_{11}F_5$)] = 250.07761.

N,4-Dimethyl-N-(4-(1,1,1,2,3-pentafluoropropan-2-yl)phenyl)benzenesulfonamide (2i)

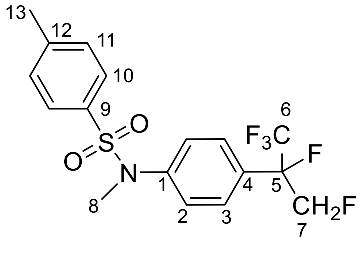

Compound **2i** was prepared according to the General Procedure **D** with an amine:HF ratio of 1:6 and an extended reaction time of 48 h using *N*,4-Dimethyl-*N*-(4-(3,3,3-trifluoroprop-1-en-2-yl)phenyl)benzene sulfonamide (**1i**) (71 mg, 0.2 mmol, 1.0 eq.). After workup, the crude mixture was analysed by ^{19}F NMR (74% NMR yield, 15.5:1 *vic:gem*). Purification by column chromatography (20% Et_2O in *n*-pentane) yielded the title compound as a pale yellow solid (49 mg, 0.12 mmol, 62%).

R*f* = 0.28 (20% Et_2O in *n*-pentane)

M.p.: 87-89 °C.

FT-IR ($\tilde{\nu}$ = cm^{-1}): 1598 (w), 1510 (w), 1339 (s), 1178 (s), 1155 (s), 1097 (m), 1065 (m), 1019 (w), 953 (m), 880 (m), 813 (m), 721 (s).

^{1}H NMR (500 MHz, $CDCl_3$): δ [ppm] = 7.44 (m, 2H, H-C3), 7.41 (m, 2H, H-C10), 7.24 (m, 2H, H-C11), 7.23 (m, 2H, H-C2), 4.93 (ddd, $^2J_{HF}$ = 47.0 Hz, $^3J_{HF}$ = 19.8 Hz, $^3J_{HH}$ = 11.0 Hz, 1H, H^a-C7), 4.86 (dddq,

$^2J_{HF}$ = 46.4 Hz, $^3J_{HF}$ = 21.7 Hz, $^3J_{HH}$ = 11.0 Hz, $^4J_{HF}$ = 0.9 Hz, 1H, H^b-C7), 3.17 (s, 3H, H-C8), 2.41 (s, 3H, H-C13).

^{13}C{^{1}H} NMR (126 MHz, $CDCl_3$): δ [ppm] = 144.1 (C12), 143.5 (d, $^5J_{CF}$ = 1.2 Hz, C1), 133.4 (C9), 129.6 (C11), 128.9 (dd, $^2J_{CF}$ = 21.5 Hz, $^3J_{CF}$ = 3.0 Hz, C4), 127.9 (C10), 126.5 (d, $^4J_{CF}$ = 1.7 Hz, C2), 126.4 (d), $^3J_{CF}$ = 9.7 Hz, C3), 122.2 (qdd, $^1J_{CF}$ = 285.5 Hz, $^2J_{CF}$ = 29.7 Hz, $^3J_{CF}$ = 4.2 Hz, C6), 93.7 (dqd, $^1J_{CF}$ = 191.9 Hz, $^2J_{CF}$ = 31.4 Hz, $^2J_{CF}$ = 18.9 Hz, C5), 81.5 (ddq, $^1J_{CF}$ = 185.6 Hz, $^2J_{CF}$ = 24.1 Hz, $^3J_{CF}$ = 1.3 Hz, C7), 37.8 (C8), 21.6 (C13).

^{19}F NMR (470 MHz, $CDCl_3$): δ [ppm] = -77.4 (ddd, $^4J_{FF}$ = 8.3 Hz, $^3J_{FF}$ = 7.1 Hz, $^4J_{HF}$ = 0.9 Hz, 3F, F-C6), -179.0 (dddq, $^3J_{HF}$ = 21.7 Hz, $^3J_{HF}$ = 19.8 Hz, $^3J_{FF}$ = 13.9 Hz, $^3J_{FF}$ = 7.1 Hz, 1F, F-C5), -234.1 (dddq, $^2J_{HF}$ = 47.0 Hz, $^2J_{HF}$ = 46.4 Hz, $^3J_{FF}$ = 13.1 Hz, $^4J_{FF}$ = 8.3 Hz, 1F, F-C7).

^{19}F{^{1}H} NMR (470 MHz, $CDCl_3$): δ [ppm] = -77.4 (dd, $^4J_{FF}$ = 8.3 Hz, $^3J_{FF}$ = 7.1 Hz, 3F, F-C6), -179.0 (dq, $^3J_{FF}$ = 13.8 Hz, $^3J_{FF}$ = 7.1 Hz, 1F, F-C5), -234.1 (dq, $^3J_{FF}$ = 13.2 Hz, $^4J_{FF}$ = 8.4 Hz, 1F, F-C7).

ESI-MS: (*m/z*) requires: $[(C_{17}H_{16}NO_2SF_5Na)]^+$ = 416.0714, (*m/z*) found: $[(C_{17}H_{16}NO_2SF_5Na)]^+$ = 416.0705.

4-Fluoro-4'-(1,1,1,2,3-pentafluoropropan-2-yl)-1,1'-biphenyl (2j)

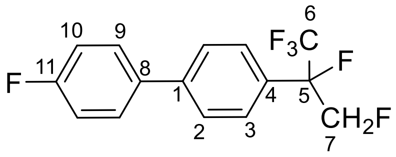

Compound **2j** was prepared according to the General Procedure **D** with an amine:HF ratio of 1:6 using 4-fluoro-4'-(3,3,3-trifluoroprop-1-en-2-yl)-1,1'-biphenyl (**1j**) (53 mg, 0.2 mmol, 1.0 eq). After workup, the crude mixture was analysed by ^{19}F NMR (74% NMR yield, 7.2:1 *vic:gem*). Purification by column chromatography (10% DCM in *n*-pentane) yielded the title compound as a white solid (24 mg, 0.08 mmol, 40%).

R_f = 0.56 (10% DCM in *n*-pentane)

M.p.: 55 – 60°C

FT-IR ($\tilde{\nu}$ = cm^{-1}): 1600 (w), 1502 (m), 1399 (w), 1297 (m), 1242 (s), 1199 (s), 1155 (s), 1125 (m), 1059 (s), 1021 (w), 974 (s), 916 (s), 818 (s), 745 (m), 714 (m), 654 (m).

^{1}H NMR (599 MHz, $CDCl_3$): δ [ppm] = 7.64 (m, 2H, H-C2), 7.58 (m, 2H, H-C3), 7.56 (m, 2H, H-C9), 7.15 (m, 2H, H-C10), 5.00 (ddd, $^2J_{HF}$ = 47.6 Hz, $^3J_{HF}$ = 20.8 Hz, $^2J_{HH}$ = 11.3 Hz, 1H, H^a-C7), 4.90 (dddq, $^2J_{HF}$ = 46.3 Hz, $^3J_{HF}$ = 22.1 Hz, $^2J_{HH}$ = 11.2 Hz, $^4J_{HF}$ = 1.5 Hz, 1H, H^b-C7).

^{13}C{^{1}H} NMR (151 MHz, $CDCl_3$): δ [ppm] =163.0 (d, $^1J_{CF}$ = 247.4 Hz, C11), 142.2 (d, $^5J_{CF}$ = 1.1 Hz, C1), 136.2 (d, $^4J_{CF}$ = 3.3 Hz, C8), 129.3 (dd, $^2J_{CF}$ = 21.3 Hz, $^3J_{CF}$ = 3.0 Hz, C4), 129.0 (d, $^3J_{CF}$ = 8.2 Hz, C9), 127.5 (d, $^4J_{CF}$ = 1.9 Hz, C2), 126.3 (dt), $^3J_{CF}$ = 9.6 Hz, $^4J_{CF}$ = 1.2 Hz, C3), 122.4 (qdd, $^1J_{CF}$ = 285.2 Hz, $^2J_{CF}$ = 29.7 Hz, $^3J_{CF}$ = 3.7 Hz, C6), 116.0 (d, $^2J_{CF}$ = 21.6 Hz, C10), 93.9 (ddd, $^1J_{CF}$ = 191.8 Hz, $^2J_{CF}$ = 31.3 Hz, $^2J_{CF}$ = 18.9 Hz, C5), 81.7 (ddd) $^1J_{CF}$ = 185.5 Hz, $^2J_{CF}$ = 23.9 Hz, $^3J_{CF}$ = 1.3 Hz, C7).

^{19}F NMR (564 MHz, $CDCl_3$): δ [ppm] = -77.3 (ddd, $^3J_{FF}$ = 8.4 Hz, $^4J_{FF}$ = 7.1 Hz, $^4J_{HF}$ = 1.5 Hz, 3F, F-C6), -114.6 (tt, $^3J_{HF}$ = 8.6 Hz, $^4J_{HF}$ = 5.3 Hz, 1F, F-C11), -179.3 (dddq, $^3J_{HF}$ = 22.1 Hz, $^3J_{HF}$ = 20.8 Hz,

$^{3}J_{FF}$ = 13.9 Hz, $^{3}J_{FF}$ = 7.1 Hz, 1F, F-C5), -233.9 (dddq, $^{2}J_{HF}$ = 47.6 Hz, $^{2}J_{HF}$ = 46.3 Hz, $^{3}J_{FF}$ = 13.2 Hz, $^{4}J_{FF}$ = 8.4 Hz, 1F, F-C7).

^{19}F {^{1}H} NMR (470 MHz, $CDCl_3$): δ [ppm] = -77.3 (dd, $^{4}J_{FF}$ = 8.4 Hz, $^{3}J_{FF}$ = 7.1 Hz, 3F, F-C6), -114.6 (s, 1F, F-C11), -179.3 (dq, $^{3}J_{FF}$ = 13.9 Hz, $^{3}J_{FF}$ = 7.1 Hz, 1F, F-C5), -233.9 (dq, $^{3}J_{FF}$ = 13.2 Hz, $^{4}J_{FF}$ = 8.4 Hz, 1F, F-C7).

GC-EI-MS: (*m/z*) requires: [($C_{15}H_{10}F_6$)] = 304.06812 (*m/z*) found: [($C_{15}H_{10}F_6$)] = 304.06800.

2-Methoxy-5-(1,1,1,2,3-pentafluoropropan-2-yl)benzonitrile (2k)

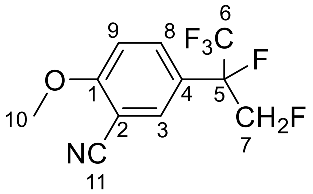

Compound **2k** was prepared according to the General Procedure **D** with an amine:HF ratio of 1:6 using 4-methoxy-5-(3,3,3-trifluoroprop-1-en-2-yl)benzonitrile (**1k**) (45 mg, 0.2 mmol, 1.0 eq.). After workup, the crude mixture was analysed by ^{19}F NMR (69% NMR yield, 10.5:1 *vic:gem*). Purification by column chromatography (20% DCM in *n*-pentane) yielded the title compound as a colourless oil (29 mg, 0.10 mmol, 50%).

R_f = 0.26 (20% DCM in *n*-pentane).

FT-IR ($\tilde{\nu}$ = cm^{-1}): 2979 (w), 2232 (m), 1615(s), 1509 (s), 1464 (m), 1283 (s), 1188 (s), 1150 (s), 1129 (s), 1100 (m), 1057 (m), 1020 (m), 981 (s), 904 (w), 824 (m), 743 (m), 726 (m), 681 (w), 653 (m).

^{1}H NMR (599 MHz, $CDCl_3$): δ [ppm] = 7.71 (d, $^{4}J_{HH}$ = 2.4 Hz, 1H, H-C3), 7.68 (dd, $^{3}J_{HH}$ = 8.8 Hz, $^{4}J_{HH}$ = 2.5 Hz, 1H, H-C8), 7.08 (d, $^{4}J_{HH}$ = 8.9 Hz, 1H, H-C9), 4.99 – 4.82 (m, 1H, H^a-C7), 4.97 – 4.80 (m, 1H, H^b-C7), 3.98 (s, 3H, H-C10).

^{13}C{^{1}H} NMR (151 MHz, $CDCl_3$): δ [ppm] = 162.4 (d, $^{5}J_{CF}$ = 1.1 Hz, C1), 132.1 (dt, $^{3}J_{CF}$ = 9.6 Hz, $^{4}J_{CF}$ = 1.4 Hz, C8), 131.6 (dt, $^{3}J_{CF}$ = 10.1 Hz, $^{4}J_{CF}$ = 1.3 Hz, C3), 123.1 (dd, $^{3}J_{CF}$ = 22.5 Hz, $^{4}J_{CF}$ = 2.8 Hz, C4), 122.0 (qdd, $^{1}J_{CF}$ = 284.7 Hz, $^{2}J_{CF}$ = 29.1 Hz, $^{3}J_{CF}$ = 4.9 Hz, C6), 115.5 (C11), 111.9 (d, $^{4}J_{CF}$ = 1.7 Hz, C9), 102.9 (d, $^{4}J_{CF}$ = 1.9 Hz, C2), 93.1 (dqd, $^{1}J_{CF}$ = 192.4 Hz, $^{2}J_{CF}$ = 31.6, $^{2}J_{CF}$ = 19.4 Hz, C5), 81.1 (ddd, $^{1}J_{CF}$ = 185.2 Hz, $^{2}J_{CF}$ = 24.9, $^{3}J_{CF}$ = 1.4 Hz, C7), 56.5 (C10).

^{19}F NMR (564 MHz, $CDCl_3$): δ [ppm] = -78.0 (m, 3F, F-C6), -178.0 (m, 1F, F-C5), -234.9 (m, 1F, F-C7).

^{19}F {^{1}H} NMR (470 MHz, $CDCl_3$): δ [ppm] = -78.0 (m, 3F, F-C6), -178.0 (dq, $^{3}J_{FF}$ = 14.6 Hz, $^{3}J_{FF}$ = 7.4 Hz, 1F, F-C5), -234.9 (m, 1F, F-C7).

ESI-MS: (*m/z*) requires: [($C_{11}H_8NOF_5Na$)]$^+$ = 288.04292, (*m/z*) found: [($C_{11}H_8NOF_5Na$)]$^+$ = 288.04185.

1-Bromo-4-(1,1,1,2,3-pentafluoropropan-2-yl)benzene (2l)

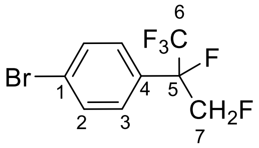

Compound **2l** was prepared according to the General Procedure **D** with an amine:HF ratio of 1:7.5 using 1-bromo-4-(3,3,3-trifluoroprop-1-en-2-yl)benzene (**1l**) (50 mg, 0.2 mmol, 1.0 eq.). After workup, the crude mixture was analysed by

^{19}F NMR (>95% NMR yield, 2.1:1 *vic:gem*). Purification by column chromatography (*n*-pentane) yielded the title compound as a colourless oil (21 mg, 0.07 mmol, 33%). Large scale synthesis: Compound **2l** was prepared according to the General Procedure **D** with an amine:HF ratio of 1:7.5 using 1-bromo-4-(3,3,3-trifluoroprop-1-en-2-yl)benzene (**1l**) (251 mg, 1.0 mmol, 1.0 eq.). Purification by column chromatography (*n*-pentane) yielded the title compound as a colourless oil (124 mg, 0.43 mmol, 43%).

R*f* = 0.45 (*n*-pentane)

FT-IR ($\tilde{\nu}$ = cm^{-1}): 2930 (w), 1596 (w), 1497 (w), 1405 (w), 1327 (w), 1295 (m), 1239 (w), 1199 (s), 1183 (s), 1153 (m), 1112 (m), 1110 (m), 1077 (m), 1054 (m), 1013 (m), 973 (w), 957 (w), 908 (s), 821 (s), 730 (s), 699 (w).

^{1}H NMR (599 MHz, CDCl$_3$): δ [ppm] = 7.65 – 7.61 (m, 2H, H-C2), 7.42 – 7.36 (m, 2H, H-C3), 5.03 – 4.92 (m, 1H, H^a-C7), 4.91 – 4.79 (m, 1H, H^b-C7).

^{13}C{^{1}H} NMR (151 MHz, CDCl$_3$): δ [ppm] = 132.3 (d, ^5JCF = 1.7 Hz, C1), 129.5 (dd, ^2JCF = 21.6 Hz, , ^3JCF = 2.9 Hz, C4), 127.5 (dd, , ^3JCF = 9.8 Hz, ^4JCF = 1.4 Hz, C3), 124.9 (d, ^5JCF = 1.5 Hz, C1), 122.1 (qdd, ^1JCF = 285.2 Hz, ^2JCF = 29.4 Hz, ^3JCF = 4.3 Hz, C6), 93.7 (dqd, ^1JCF = 192.3 Hz, ^2JCF = 31.4 Hz, ^2JCF = 19.1 Hz), 81.4 (ddq, ^1JCF = 185.5 Hz, ^2JCF = 31.4 Hz, ^3JCF = 1.3 Hz, C7).

^{19}F NMR (470 MHz, CDCl$_3$): δ [ppm] = 77.6 (m, 3F, F-C6), -179.2 (m, 1F, F-C5), -234.4 (m, 1F, F-C6).

^{19}F {^{1}H} NMR (470 MHz, CDCl$_3$): δ [ppm] = -77.6 (dd, , $^4J_{FF}$ = 8.0, $^3J_{FF}$ = 7.1 Hz), -179.2 (dq, , $^3J_{FF}$ = 13.3 Hz, $^3J_{FF}$ = 7.1 Hz), -234.4 (dq, $^3J_{FF}$ = 13.3 Hz, , $^4J_{FF}$ = 8.1 Hz).

GC-EI-MS: (*m/z*) requires: [($C_9H_6BrF_5$)] = 287.9568, (*m/z*) found: [($C_9H_6BrF_5$)] = 287.9567.

1-(Methoxymethyl)-4-(1,1,1,2,3-pentafluoropropan-2-yl)benzene (2m)

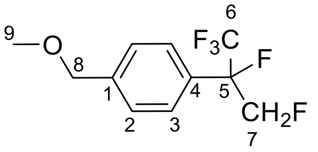

Compound **2m** was prepared according to the General Procedure **D** with an amine:HF ratio of 1:7.5 using 1-(methoxymethyl)-4-(3,3,3-trifluoroprop-1-en-2-yl)benzene (**1m**) (43 mg, 0.2 mmol, 1.0 eq). After workup, the crude mixture was analysed by ^{19}F NMR (91% NMR yield, 3.5:1 *vic:gem*). Purification by preparative thin layer chromatography (5% Et_2O in *n*-pentane) yielded the title compound as a colourless oil (24mg, 0.09 mmol, 47%).

R*f* = 0.29 (5% Et_2O in *n*-pentane)

FT-IR ($\tilde{\nu}$ = cm^{-1}): 2930 (w), 1518 (w), 1454 (w), 1419 (w), 1383 (w), 1328 (w), 1287 (w), 1237 (w), 1176 (s), 1151 (m), 1097 (s), 1052 (m), 1021 (w), 957 (m), 909 (m), 813 (w), 759 (m), 733 (w).

^{1}H NMR (599 MHz, CDCl$_3$): δ [ppm] = 7.49 (m, 2H, H-C3), 7.44 (m, 2H, H-C2), 4.95 (ddd, $^2J_{HF}$ = 47.1 Hz, $^3J_{HF}$ = 21.2 Hz, $^3J_{HH}$ = 11.3 Hz, 1H, H^a-C7), 4.85 (dddq, $^2J_{HF}$ = 46.5 Hz, $^3J_{HF}$ = 21.5 Hz, $^3J_{HH}$ = 11.0 Hz, $^4J_{HF}$ = 1.3 Hz, 1H, H^b-C7), 4.50 (s, 2H, H-C8), 3.42 (s, 3H, H-C9).

^{13}C{^{1}H} NMR (151 MHz, CDCl$_3$): δ [ppm] = 140.7 (d, $^5J_{CF}$ = 1.0 Hz, C1), 129.7 (dd, $^2J_{CF}$ = 21.3 Hz, $^3J_{CF}$ = 3.2 Hz, C4), 129.0 (d, $^4J_{CF}$ = 1.8 Hz, C2), 125.8 (d) $^3J_{CF}$ = 9.6 Hz, C3), 122.4 (qdd, $^1J_{CF}$ = 285.2 Hz,

$^{2}J_{CF}$ = 29.9 Hz, $^{3}J_{CF}$ = 3.7 Hz, C6), 93.9 (dqd, $^{1}J_{CF}$ = 191.7 Hz, $^{2}J_{CF}$ = 31.2 Hz, $^{2}J_{CF}$ = 18.6 Hz, 1C, C5), 81.7 (ddq, $^{1}J_{CF}$ = 185.6 Hz, $^{2}J_{CF}$ = 23.8 Hz, $^{3}J_{CF}$ = 1.2 Hz, C7), 74.06 (C8), 58.5 (C9).

^{19}F NMR (564 MHz, $CDCl_3$): δ [ppm] = -77.3 (ddd, $^{4}J_{FF}$ = 8.3 Hz, $^{3}J_{FF}$ = 6.9 Hz, $^{4}J_{HF}$ = 1.3 Hz, 3F, F-C6), -179.6 (dddq, $^{3}J_{HF}$ = 21.5 Hz, $^{3}J_{HF}$ = 21.2 Hz, $^{3}J_{FF}$ = 13.7 Hz, $^{3}J_{FF}$ = 6.9 Hz, 1F, F-C5), -233.7 (dddq, $^{2}J_{HF}$ = 47.1 Hz, $^{2}J_{HF}$ = 46.5 Hz, $^{3}J_{FF}$ = 13.1 Hz, $^{4}J_{FF}$ = 8.5 Hz, 1F, F-C7).

^{19}F{^{1}H} NMR (376 MHz, $CDCl_3$): δ [ppm] = -77.3 (dd, $^{4}J_{FF}$ = 8.3 Hz, $^{3}J_{FF}$ = 6.9 Hz, 3F, F-C6), -179.6 (dq, $^{3}J_{FF}$ = 13.7, 6.9 Hz, 1F, F-C5), -233.7 (dq, $^{3}J_{FF}$ = 13.0 Hz, $^{4}J_{FF}$ = 8.7 Hz, 1F, F-C7).

EI-MS: (*m/z*) requires: $[(C_{11}H_{11}F_5O)]^+$ = 254.0725, (*m/z*) found: $[(C_{11}H_{11}F_5O)]^+$ = 254.0735; (*m/z*) requires: $[(C_{11}H_{10}F_5O)]^+$ = 253.0646, (*m/z*) found: $[(C_{11}H_{10}F_5O)]^+$ = 253.0650.

1-(1,1,1,2,3-Pentafluoropropan-2-yl)-4-(trifluoromethoxy)benzene (2n)

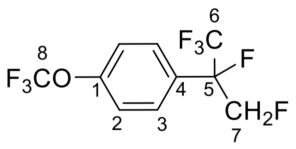

Compound **2n** was prepared according to the General Procedure **D** with an amine:HF ratio of 1:7.5 using 1-(trifluoromethoxy)-4-(3,3,3-trifluoroprop-1-en-2-yl)benzene (**1n**) (51 mg, 0.2 mmol, 1.0 eq.). After workup, the crude mixture was analysed by ^{19}F NMR (94% NMR yield, 2.6:1 *vic:gem*). Purification by preparative thin layer chromatography (*n*-pentane) yielded the title compound as a colourless oil (9 mg, 0.03 mmol, 15%).

Large scale synthesis: Compound **2n** was prepared according to the General Procedure **D** with an amine:HF ratio of 1:7.5 using 1-(trifluoromethoxy)-4-(3,3,3-trifluoroprop-1-en-2-yl)benzene (**1n**) (256 mg, 1.0 mmol, 1.0 eq.). Purification by column chromatography (*n*-pentane) yielded the title compound as a colourless oil (32 mg, 0.23 mmol, 23%).

R_f = 0.67 (*n*-pentane).

FT-IR ($\tilde{\nu}$ = cm^{-1}): 1615 (w), 1515 (s), 1466 (w), 1396 (w), 1327 (s), 1257 (s), 1167 (s), 1112 (s), 1100 (s), 1055 (s), 1020 (s), 974 (s), 960 (s), 911 (s), 852 (s), 809 (s), 761 (m), 737 (s), 707 (s), 684 (s).

^{1}H NMR (599 MHz, $CDCl_3$): δ [ppm] = 7.57 (m, 2H, H-C3), 7.32 (m, 2H, H-C2), 5.01 – 4.91 (m, 1H, H^a-C7), 4.91 – 4.81 (m, 1H, H^b-C7).

^{13}C{^{1}H} NMR (151 MHz, $CDCl_3$): δ [ppm] = 150.7 (m, C1), 129.0 (dd, $^{2}J_{CF}$ = 21.8 Hz, $^{3}J_{CF}$ = 2.9 Hz, C4), 127.7 (dt, $^{3}J_{CF}$ = 9.9 Hz, $^{4}J_{CF}$ = 1.4 Hz, C3), 122.2 (qdd, $^{1}J_{CF}$ = 285.2 Hz, $^{2}J_{CF}$ = 29.4 Hz, $^{2}J_{CF}$ = 4.4 Hz, C6), 121.2 (d, $^{4}J_{CF}$ = = 1.4 Hz, C2), 120.5 (q, $^{1}J_{CF}$ = 258.4 Hz, C8), 93.6 (dqd, $^{1}J_{CF}$ = 192.2 Hz, $^{2}J_{CF}$ = 31.4 Hz, $^{2}J_{CF}$ = 19.0 Hz, C5), 81.4 (ddq, $^{1}J_{CF}$ = 185.6 Hz, $^{2}J_{CF}$ = 24.6 Hz, $^{3}J_{CF}$ = 1.4 Hz, C7).

^{19}F NMR (564 MHz, $CDCl_3$): δ [ppm] = -58.0 (m, 1F, F-C8), -77.7(m, 3F, F-C6), -178.7 (m, 1F, F-C5), -234.5 (m, 1F, F-C7).

^{19}F{^{1}H} NMR (564 MHz, $CDCl_3$): δ [ppm] = 57.96, -77.7 (dd, $^{4}J_{FF}$ = 8.0 Hz, $^{3}J_{FF}$ = 7.1 Hz, 3F, F-C6), -178.7 (dq, $^{3}J_{FF}$ = 14.1, $^{3}J_{FF}$ = 7.2 Hz, 1F, F-C5), -234.5 (dq, $^{3}J_{FF}$ = 13.3 Hz, $^{4}J_{FF}$ =8.0 Hz, 1F, F-C7).

GC-EI-MS: (*m/z*) requires: $[(C_{10}H_6OF_8)]$ = 294.02854, (*m/z*) found: $[(C_{10}H_6OF_8)]$ = 294.02844.

4-Nitro-4'-(1,1,1,2,3-pentafluoropropan-2-yl)-1,1'-biphenyl (2o)

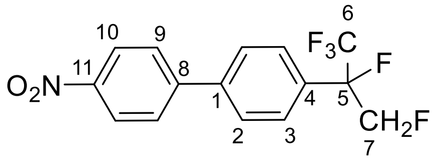

Compound **2o** was prepared according to the General Procedure **D** with an amine:HF ratio of 1:7.5 using 4-nitro-4'-(3,3,3-trifluoroprop-1-en-2-yl)-1,1'-biphenyl (**1o**) (59 mg, 0.2 mmol, 1.0 eq.). After workup, the crude mixture was analysed by ^{19}F NMR (83% NMR yield, 2.6:1 *vic:gem*). Purification by column chromatography (20% DCM in *n*-pentane) yielded the title compound as a yellow solid (26 mg, 0.08 mmol, 40%).

R*f* = 0.36 (20% DCM in *n*-pentane)

M.p.: 82 – 84°C

FT-IR ($\tilde{\nu}$ = cm^{-1}): 1599 (s), 1571 (w), 1518 (s), 1489 (m), 1394 (w), 1345 (s), 1291 (s), 1238 (m), 1196 (s), 1156 (s), 1112 (s), 1101 (s), 1054 (s), 1007 (w), 957 (s), 911 (m), 855 (s), 828 (s), 769 (w), 758 (m), 738 (s), 693 (s), 676 (w).

^{1}H NMR (599 MHz, $CDCl_3$): δ [ppm] = 8.32 (m, 2H, H-C10), 7.76 (m, 2H, H-C9), 7.74 – 7.71 (m, 2H, H-C2), 7.66 (m, 2H, H-C3), 5.01 (ddd, $^2J_{HF}$ = 46.5 Hz, $^3J_{HF}$ = 20.0 Hz, $^2J_{HH}$ = 11.1 Hz, 1H, H^a-C7), 4.94 (dddq, $^2J_{HF}$ = 46.3 Hz, $^3J_{HF}$ = 22.3 Hz, $^2J_{HH}$ = 11.5, $^4J_{HF}$ = 1.2 Hz, 1H, H^b-C7).

^{13}C{^{1}H} NMR (151 MHz, $CDCl_3$): δ [ppm] = 147.7 (C11), 146.3 (C8), 140.7 (d, $^5J_{CF}$ = 1.1 Hz, C1), 131.0 (dd, $^2J_{CF}$ = 21.5 Hz, $^3J_{CF}$ = 2.9 Hz, C4), 128.1 (C9), 127.9 (d, $^4J_{CF}$ = 1.8 Hz, C2), 126.7 (dt), $^3J_{CF}$ = 9.8 Hz, $^4J_{CF}$ = 1.4 Hz, C3), 124.3 (C10), 122.3 (qdd, $^1J_{CF}$ = 285.1 Hz, $^2J_{CF}$ = 29.5 Hz, $^4J_{CF}$ = 4.1 Hz, C6), 93.8 (dqd, $^1J_{CF}$ = 192.3 Hz, $^2J_{CF}$ = 31.3 Hz, $^2J_{CF}$ = 19.1 Hz, C5), 81.5 (ddd) $^1J_{CF}$ = 186.1 Hz, $^2J_{CF}$ = 23.9 Hz, $^3J_{CF}$ = 1.0 Hz, C7).

^{19}F NMR (564 MHz, $CDCl_3$): δ [ppm] = -77.3 (ddd, $^4J_{FF}$ = 8.2 Hz, $^3J_{FF}$ = 7.4 Hz, $^4J_{HF}$ = 1.2 Hz, 3F, F-C6), -179.3 (dddq, $^3J_{HF}$ = 22.2 Hz, $^3J_{HF}$ = 20.4 Hz, $^3J_{FF}$ = 13.4 Hz, $^3J_{FF}$ = 7.1 Hz, 1F, F-C5), -234.3 (dddq, $^2J_{HF}$ = 46.5 Hz, $^2J_{HF}$ = 46.3 Hz, $^3J_{FF}$ = 13.1 Hz, $^3J_{FF}$ = 8.0 Hz, 1C, F-C7).

^{19}F {^{1}H} NMR (564 MHz, $CDCl_3$): δ [ppm] = -77.3 (m, 3F, F-C6), -179.3 (dq, $^3J_{FF}$ = 14.1 Hz, $^3J_{FF}$ = 7.1 Hz, 1F, F-C5), -234.3 (dq, $^3J_{FF}$ = 13.1 Hz, $^3J_{FF}$ = 8.0 Hz, 1F, F-C7).

GC-EI-MS: (*m/z*) requires: [($C_{15}H_{10}NO_2F_5$)] = 331.06262, (*m/z*) found: [($C_{15}H_{10}NO_2F_5$)] = 331.06266

2-(4-(1,1,1,2,3-pentafluoropropan-2-yl)phenyl)isoindoline-1,3-dione (2p)

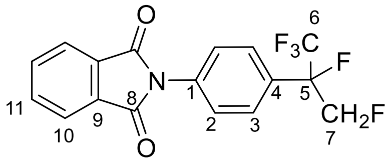

Compound **2p** was prepared according to the General Procedure **D** with an amine:HF ratio of 1:7.5 and an extended reaction time of 48 h using 2-(4-(3,3,3)-trifluoroprop-1-en-2-yl)phenyl)isoindoline-1,3-dione (**1p**) (63 mg, 0.2 mmol, 1.0 eq.). After workup, the crude mixture was analysed by ^{19}F NMR (>95% NMR yield, 2.4:1 *vic:gem*). Purification by column chromatography (25% Et_2O in *n*-pentane) yielded the title compound as a white solid (43 mg, 0.12 mmol, 61%).

R*f* = 0.28 (25% Et_2O in *n*-pentane)

M.p.: 118-120 °C.

FT-IR ($\tilde{\nu}$ = cm^{-1}): 1711 (s), 1519 (w), 1378 (m), 1329 (w), 1180 (s), 1155 (s), 1123 (m), 1099 (s), 955 (m), 885 (m), 832 (m), 791 (w), 761 (w), 718 (s), 700 (w).

^{1}H NMR (500 MHz, $CDCl_3$): δ [ppm] = 7.97 (dd, $^3J_{HH}$ = 5.5 Hz, $^4J_{HH}$ = 3.0 Hz, 2H, H-C10), 7.81 (dd, $^3J_{HH}$ = 5.5 Hz, $^4J_{HH}$ = 3.1 Hz, 2H, H-C11), 7.66 (d, $^3J_{HH}$ = 8.9 Hz, 2H, H-C2), 7.62 (d, $^3J_{HH}$ = 8.8 Hz, 2H, H-C3), 4.97 (ddd, $^2J_{HF}$ = 47.0 Hz, $^3J_{HF}$ = 20.4 Hz, $^3J_{HH}$ = 11.0, 1H, H^a-C7), 4.89 (dddq, $^2J_{HF}$ = 46.4 Hz, $^3J_{HF}$ = 21.2 Hz, $^3J_{HH}$ = 11.2 Hz, $^4J_{HF}$ = 1.2 Hz, 1H, H^b-C7).

^{13}C{^{1}H} NMR (126 MHz, $CDCl_3$): δ [ppm] = 167.0 (C8), 134.8 (C11), 133.7 (d, $^5J_{CF}$ = 1.4 Hz, C1), 131.7 (C9), 129.9 (dd, $^2J_{CF}$ = 21.4 Hz, $^3J_{CF}$ = 3.0 Hz, C4), 126.7 (d, $^3J_{CF}$ = 10.0 Hz, C3), 126.6 (d, $^4J_{CF}$ = 1.9 Hz, C2), 124.1 (C10), 122.3 (qdd, $^1J_{CF}$ = 285.5 Hz, $^2J_{CF}$ = 29.7 Hz, $^3J_{CF}$ = 4.0 Hz, C6), 93.7 (dqd, $^1J_{CF}$ = 192.2 Hz, $^2J_{CF}$ = 31.4 Hz, $^2J_{CF}$ = 18.8 Hz, C5), 81.6 (ddq, $^1J_{CF}$ = 185.6 Hz, $^2J_{CF}$ = 24.1 Hz, $^3J_{CF}$ = 1.0 Hz, C7).

^{19}F NMR (377 MHz, $CDCl_3$): δ [ppm] = -77.1 (ddd, $^4J_{FF}$ = 8.5 Hz, $^3J_{FF}$ = 7.0 Hz, $^4J_{HF}$ = 1.2 Hz, 3F, F-C6), -179.4 (dddq, $^3J_{HF}$ = 21.2 Hz, $^3J_{HF}$ = 20.4 Hz, $^3J_{FF}$ = 13.9 Hz, $^3J_{FF}$ = 7.1 Hz, 1F, F-C5), -233.6 (dddq, $^2J_{HF}$ = 47.0 Hz, $^2J_{HF}$ = 46.4 Hz, $^3J_{FF}$ = 13.1 Hz, $^4J_{FF}$ = 8.5 Hz, 1F, F-C7).

^{19}F{^{1}H} NMR (470 MHz, $CDCl_3$): δ [ppm] = -77.1 (dd, $^4J_{FF}$ = 8.5 Hz, $^3J_{FF}$ = 7.0 Hz, 3F, F-C6), -179.4 (dq, $^3J_{FF}$ = 14.3 Hz, $^3J_{FF}$ = 7.5 Hz, 1F, F-C5), -233.6 (dq, $^3J_{FF}$ = 13.1 Hz, $^4J_{FF}$ = 8.5 Hz, 1F, F-C7).

ESI-MS: (*m/z*) requires: $[(C_{17}H_{10}NO_2F_5Na)]^+$ = 378.0524, (*m/z*) found: $[(C_{17}H_{10}NO_2F_5Na)]^+$ = 378.0526.

2-Methoxy-5-(1,1,1,2,3-pentafluoropropan-2-yl)phenyl trifluoromethanesulfonate (2q)

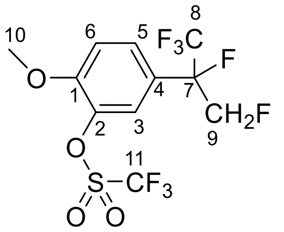

Compound **2q** was prepared according to the general procedure **D** with an amine:HF ratio of 1:7.5 using 2-methoxy-5-(3,3,3-trifluoroprop-1-en-2-yl)phenyl trifluoromethanesulfonate (**1q**) (70 mg, 0.2 mmol, 1.0 eq.). After workup, the crude mixture was analysed by ^{19}F NMR (91% NMR yield, 5.0:1 *vic:gem*). Purification by column chromatography (*n*-pentane) yielded the title compound as a colourless oil (47 mg, 0.12 mmol, 60%).

R*f* = 0.17 (*n*-pentane)

FT-IR ($\tilde{\nu}$ = cm^{-1}): 2973 (w), 1624 (m), 1521 (s), 1465 (m), 1423 (s), 1307 (s), 1282 (s), 1250 (s), 1198 (s), 1182 (s), 1137 (s), 1112 (s), 1098 (s), 1058 (s), 1022 (s), 981 (s), 947 (s), 918 (s), 879 (s), 852 (s), 809 (s), 772 (m), 749 (m), 733 (s), 667 (s).

^{1}H NMR (599 MHz, $CDCl_3$): δ [ppm] = 7.47 (dd, $^3J_{HH}$ = 8.8 Hz, $^4J_{HH}$ = 2.3 Hz, 1H, H-C5), 7.38 (d, $^4J_{HH}$ = 2.3 Hz, 1H, H-C3), 7.13 (d, $^3J_{HH}$ = 8.6 Hz, 1H, H-C6), 4.89 (dddq, $^2J_{HF}$ = 46.2 Hz, $^3J_{HF}$ = 21.9 Hz, $^3J_{HH}$ = 9.5 Hz, $^4J_{HF}$ = 1.2 Hz, 1H, H^a-C7), 4.88 (dddq, $^2J_{HF}$ = 46.8 Hz, $^3J_{HF}$ = 18.8 Hz, $^3J_{HH}$ = 11.7 Hz, $^4J_{HF}$ = 0.8 Hz, 1H, H^b-C7)3.96 (s, 3H, H-C10).

$^{13}C\{^1H\}$ NMR (151 MHz, $CDCl_3$): δ [ppm] = 153.0 (d, $^5J_{CF}$ = 1.1 Hz, 1C, C1), 138.9 (d, $^4J_{CF}$ = 2.1 Hz, 1C, C2), 127.0 (dt), $^3J_{CF}$ = 9.6 Hz, $^4J_{CF}$ = 1.4 Hz, 1C, C5), 123.2 (dd, $^2J_{CF}$ = 22.4, $^3J_{CF}$ = 2.9 Hz, 1C, C4), 122.1 (qdd, $^1J_{CF}$ = 285.4 Hz, $^2J_{CF}$ = 29.6 Hz, $^3J_{CF}$ = 4.1 Hz,1C, C8), 120.8 (d, $^3J_{CF}$ = 10.8 Hz, 1C,C3), 118.9 (q, $^1J_{CF}$ = 320.5 Hz, 1C, C11), 113.5 (d, $^4J_{CF}$ = 1.5 Hz, 1C, C6), 93.0 (ddd*, $^1J_{CF}$ = 192.4 Hz, $^2J_{CF}$ = 31.7 Hz, $^2J_{CF}$ = 19.2 Hz, 1C, C7), 81.2 (ddq, $^1J_{CF}$ = 185.5 Hz, $^2J_{CF}$ = 24.9 Hz, $^3J_{CF}$ = 1.4 Hz 1C, C9), 56.6 (1C, C10).

^{19}F NMR (564 MHz, $CDCl_3$): δ [ppm] = -73.8 (s, 3F, F-C11), -77.9 (dddd, $^4J_{FF}$ = 8.0 Hz, $^3J_{FF}$ = 7.2 Hz, $^4J_{HF}$ = 1.2 Hz, $^4J_{HF}$ = 0.8 Hz , 3F, F-C6), -177.7 (dddq, $^3J_{HF}$ = 21.9 Hz, $^3J_{HF}$ = 18.8 Hz, $^3J_{FF}$ = 12.4 Hz, $^3J_{FF}$ = 7.2 Hz, 1F, F-C5), -234.5 (dddq, $^2J_{HF}$ = 46.8 Hz, $^2J_{HF}$ = 46.2 Hz, $^3J_{FF}$ = 13.6 Hz, $^4J_{FF}$ = 8.0 Hz, 1F, C-C7).

$^{19}F\ \{^1H\}$ NMR (376 MHz, $CDCl_3$): δ [ppm] = -73.8 (s, 3F, F-C11), -77.9 (dd, $^4J_{FF}$ = 8.0 Hz, $^3J_{FF}$ = 7.2 Hz, 3F, F-C6), -177.7 (dq, $^3J_{FF}$ = 12.4 Hz, $^3J_{FF}$ = 7.2 Hz, 1F, F-C5), -234.5 (dq, $^3J_{FF}$ = 13.6 Hz, $^4J_{FF}$ = 8.0 Hz,1F, F-C7).

ESI-MS: (*m/z*) requires: $[(C_{11}H_{18}O_4F_8S)Na]^+$ = 410.99078, (*m/z*) found: $[(C_{11}H_{18}O_4F_8S)Na]^+$ = 410.99067.

2.5. Optimisation of the Enantioselective Reaction

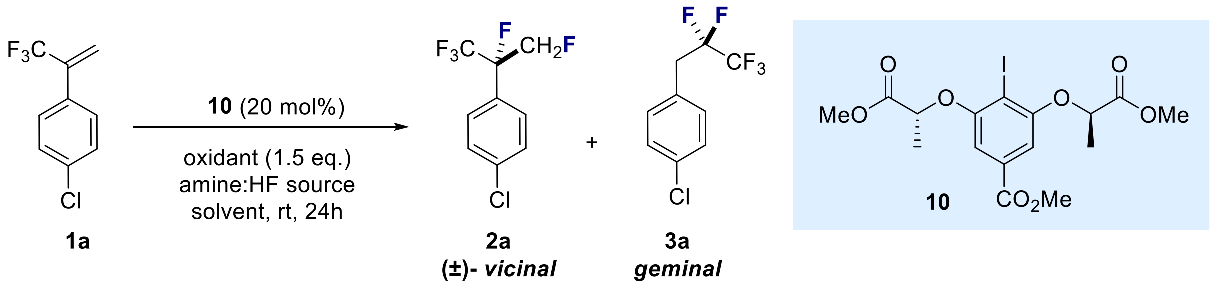

Table S3. Conditions Screen[a]

Entry	Solvent	Oxidant	Amine : HF ratio	Conversion [%][b]	Yield [%][b] (*vic*:*gem*)	*e.r.* (*vic*)
1	$CHCl_3$	Selectfluor	1:7.5	>95	88 (2.4:1)	86:14
2	CH_2Cl_2	Selectfluor	1:7.5	>95	84 (2.1:1)	86:14
3	DCE	Selectfluor	1:7.5	>95	77 (2.2:1)	84:16
4	HFIP	Selectfluor	1:7.5	>95	78 (1.7:1)	86:14
5	toluene	Selectfluor	1:7.5	14	>5	-
6	$CHCl_3$	*m*CPBA	1:7.5	>95	75 (2.3:1)	86:14
7	$CHCl_3$	*t*BuOOH	1:7.5	19	<5	-
8	$CHCl_3$	Oxone	1:7.5	43	<5	-
9	$CHCl_3$	Selectfluor	1:5	24	<5	-
10	$CHCl_3$	Selectfluor	1:5.5	24	<5	-
11	$CHCl_3$	Selectfluor	1:6	33	9 (3.5:1)	86:14
12	$CHCl_3$	Selectfluor	1:6.5	77	63 (2.9:1)	86:14
13	$CHCl_3$	Selectfluor	1:7	90	70 (2.7:1)	86:14
14	$CHCl_3$	Selectfluor	1:9.23	>95	64 (1.7:1)	79:21
15	$CHCl_3$	Selectfluor	DMPU:HF	>95	47 (0.9:1)	74:26
16[c]	$CHCl_3$	Selectfluor	1:7.5	69	58 (2.6:1)	86:14
17[d]	$CHCl_3$	Selectfluor	1:7.5	7%	<5	-
18[e]	$CHCl_3$	Selectfluor	1:7.5	9%	<5	-
19[f]	$CHCl_3$	Selectfluor	1:7.5	13%	<5	-
20	$CHCl_3$	-	1:7.5	31	<5	-
21	$CHCl_3$	Selectfluor	-	19	<5	-
22[g]	$CHCl_3$	Selectfluor	1:7.5	22	<5	-

[a] Standard reaction conditions: 1-chloro-4-(3,3,3-trifluoroprop-1-en-2-yl)benzene (0.2 mmol), 10 (20 mol%), oxidant (1.5 equiv.), amine:HF source (0.5 mL), solvent (0.5 mL) 24 h, ambient temperature. [b] *Vicinal* and *geminal* product combined, determined by ^{19}F NMR using α,α,α- trifluorotoluene as an internal standard. [c] at 10 °C for 72 h. [d] at 0 °C for 72 h. [e] at -10 °C for 7 days. [f] with 10 mol% HFIP at 0 °C for 72 h. [g] without catalyst.

Correlating amine:HF ratio to regioselectivity (Entry 9-14)

During reaction optimisation a correlation between the Brønsted acidity (amine:HF ratio) and regioselectivity was observed. Ratios with higher Brønsted acidity had an undesirable effect favouring the geminal byproduct.[1] However, typically lowering the acidity was detrimental to overall conversion. At this outcome is highly substrate dependent three methods varying amine:HF ratios (1:4:5, 1:6, 1:7.5) were developed and tested on each substrate.

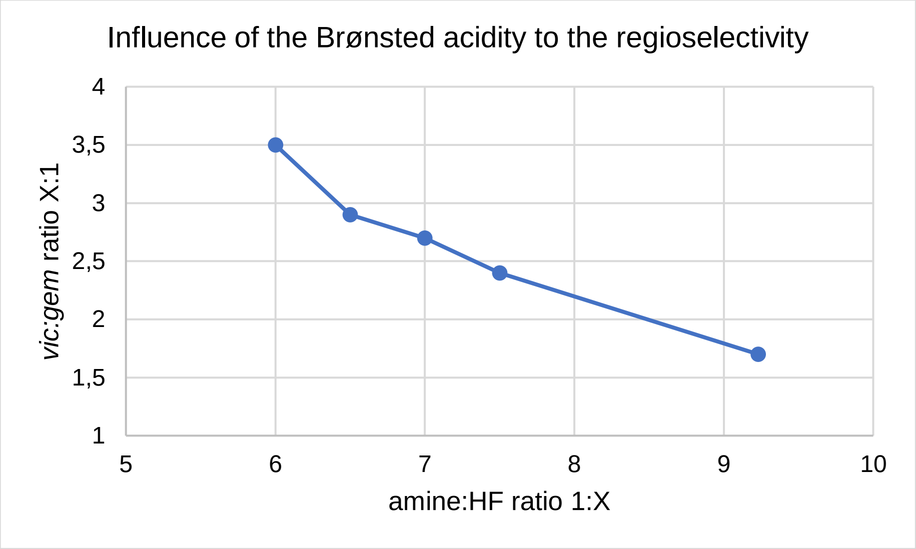

Figure S5. Influence of Brønsted acidity versus regioselectivity.

2.6. Synthesis of Chiral Pentafluoroisopropyl Surrogates

General procedure E for the asymmetric 1,2-difluorination

Unless stated otherwise, a Teflon® vial was equipped with a 1 cm stirring bar followed by the addition of styrene (0.5 mmol, 1.0 eq.), catalyst **10** (47 mg, 0.1 mmol, 20 mol%) and $CHCl_3$ (1.25 mL). The mixture was stirred and the stated amine:HF mixture was added (1.25 mL) via syringe. After stirring for 1 min, Selectfluor® (266 mg, 0.75 mmol, 1.5 eq.) was added in one portion. The reaction vessel was then sealed with a Teflon® screw cap. After stirring (350 rpm) at ambient temperature for 24 h, DCM (2 mL) was added to dilute the reaction and a saturated aqueous solution of $NaHCO_3$ (2 mL) was added via a long glass pipette to prequench the reaction. The mixture was poured in an Erlenmeyer flask charged with 250 mL of a saturated aqueous solution of $NaHCO_3$ (CAUTION, strong generation of CO_2!). The Teflon® vial was rinsed with DCM and dropped into another flask with saturated aqueous solution of $NaHCO_3$ to guarantee the removal of excess HF. The organics were extracted with DCM (3x 75 mL) and the combined organic layers were dried over $MgSO_4$ and solvent was carefully removed under reduced pressure. An internal standard (α-, α-, α-trifluorotoluene) was added to the crude residue and the NMR yield and regioselectivity ratio (*vicinal:geminal*) was analysed by ^{19}F NMR spectroscopy against the

internal standard. The NMR sample was recombined with the crude residue and purification by column chromatography or preparative thin layer chromatography yielded the desired product.

1-Chloro-4-(1,1,1,2,3-pentafluoropropan-2-yl)benzene (2a)

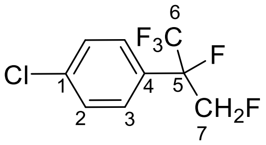

Compound **2a** was prepared according to the General Procedure **E** with an amine:HF ratio of 1:7.5 using 1-chloro-4-(3,3,3-trifluoroprop-1-en-2-yl)benzene (**1a**) (103 mg, 0.5 mmol, 1.0 eq). After workup, the crude mixture was analysed by ^{19}F NMR (>95% NMR yield, 2.2:1 *vic:gem*). Purification by column chromatography (*n*-pentane) yielded the title compound as a colourless oil (55 mg, 0.22 mmol, 45%).

The enantiomeric ratio was determined by chiral HPLC with a chiral OM column, *n*-hexane : isopropanol 99.9:0.1, 0.5 mL/min, t_R major = 13.1 min, t_R minor = 14.5 min, *e.r.* = 86:14.

ORD ($CHCl_3$, c 1.00) $[\alpha]_D^{25}$ = +5.246

HPLC trace ±**2a**

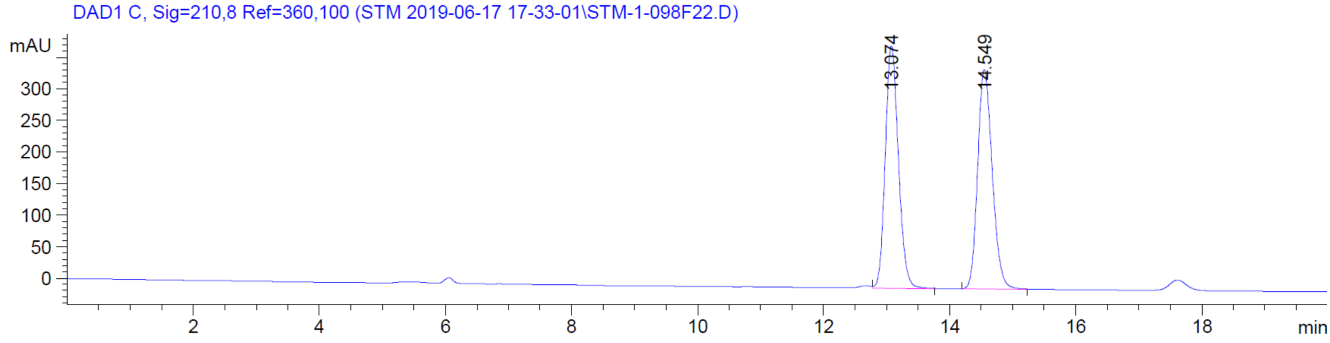

Signal 2: DAD1 C, Sig=210,8 Ref=360,100

Peak #	RetTime [min]	Type	Width [min]	Area [mAU*s]	Height [mAU]	Area %
1	13.074	VB	0.2226	5547.54883	383.94913	49.8109
2	14.549	BB	0.2496	5589.67285	347.65656	50.1891
Totals :				1.11372e4	731.60568	

HPLC trace (*S*)-**2a**

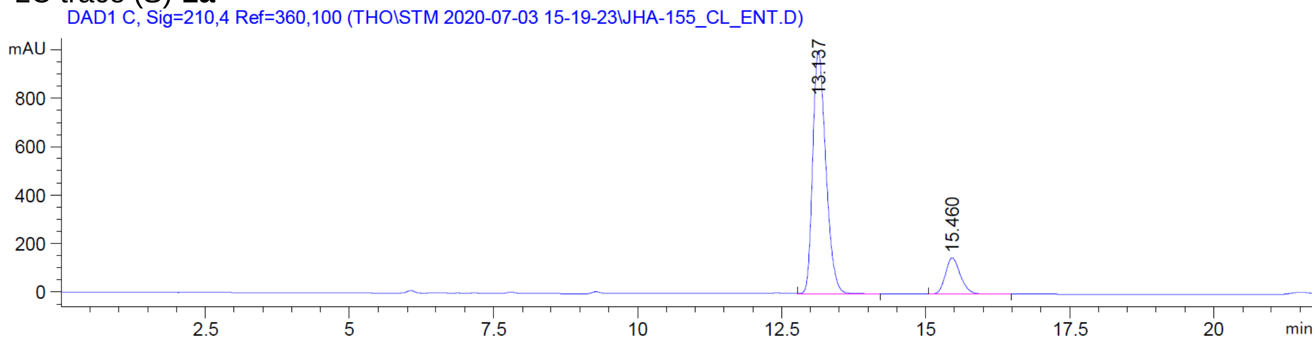

Signal 3: DAD1 C, Sig=210,4 Ref=360,100

Peak #	RetTime [min]	Type	Width [min]	Area [mAU*s]	Height [mAU]	Area %
1	13.137	VB	0.2442	1.58600e4	1004.37390	85.5297
2	15.460	BB	0.2776	2683.27197	149.32204	14.4703
Totals :				1.85433e4	1153.69594	

1-Methyl-4-(1,1,1,2,3-pentafluoropropan-2-yl)benzene (2e)

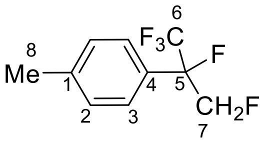

Compound **2e** was prepared according to General Procedure **E** with an amine:HF ratio of 1:6 using 1-methyl-4-(3,3,3-trifluoroprop-1-en-2-yl)benzene (**1e**) (93 mg, 0.5 mmol, 1.0 eq.). After workup, the crude mixture was analysed by ^{19}F NMR (>95% NMR yield, 17:1 *vic:gem*). Purification by column chromatography (*n*-pentane) yielded the title compound as colourless oil (75 mg, 0.34 mmol, 67%).

The enantiomeric ratio was determined by chiral HPLC with a chiral OM column, *n*-hexane : isopropanol 99.9:0.1, 0.5 mL/min, t_R major = 11.9 min, t_R minor = 13.2 min, *e.r.* = 83:17.

ORD ($CHCl_3$, c 1.00) $[\alpha]_D^{25}$ = +5.582

HPLC trace ±**2e**

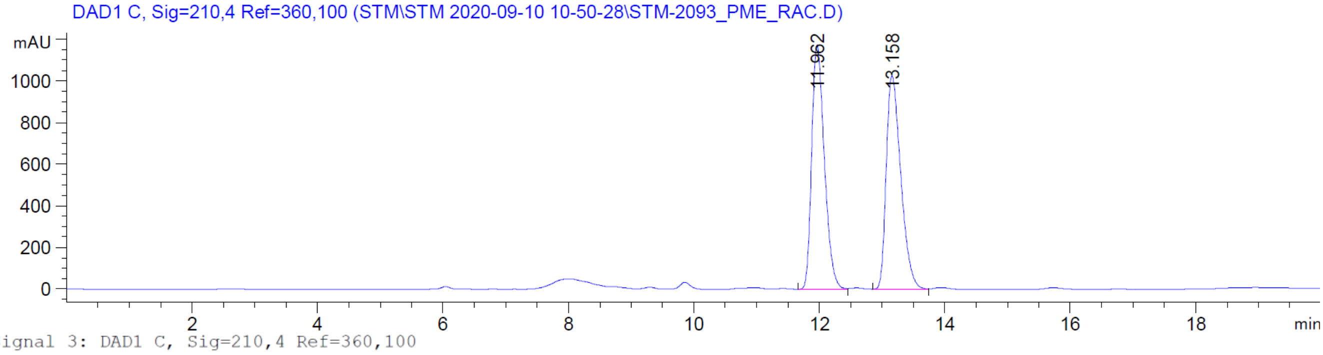

Signal 3: DAD1 C, Sig=210,4 Ref=360,100

Peak #	RetTime [min]	Type	Width [min]	Area [mAU*s]	Height [mAU]	Area %
1	11.962	BV	0.2156	1.64662e4	1174.31641	49.8999
2	13.158	BV	0.2479	1.65322e4	1026.65674	50.1001
Totals :				3.29984e4	2200.97314	

HPLC trace (*S*)-**2e**

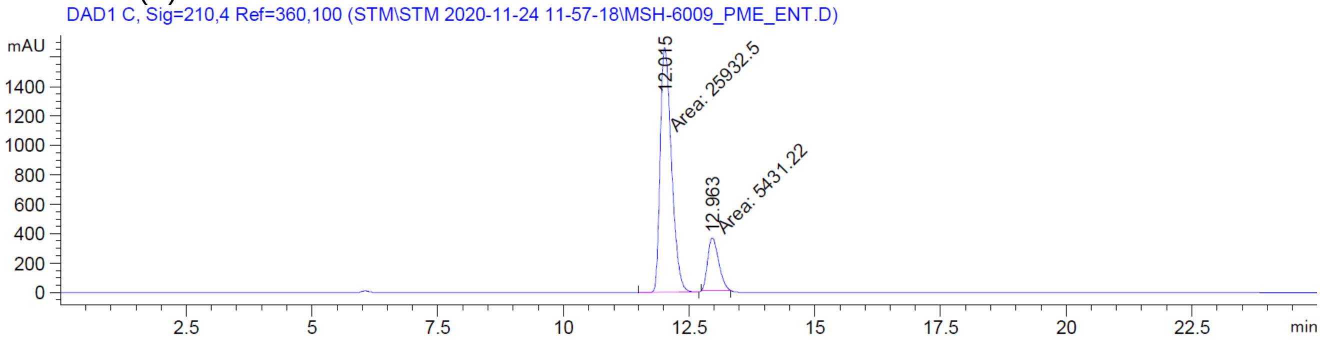

Signal 3: DAD1 C, Sig=210,4 Ref=360,100

Peak #	RetTime [min]	Type	Width [min]	Area [mAU*s]	Height [mAU]	Area %
1	12.015	MM	0.2599	2.59325e4	1663.02844	82.6831
2	12.963	MM	0.2529	5431.22119	357.96863	17.3169
Totals :				3.13637e4	2020.99707	

4-Fluoro-4'-(1,1,1,2,3-pentafluoropropan-2-yl)-1,1'-biphenyl (2j)

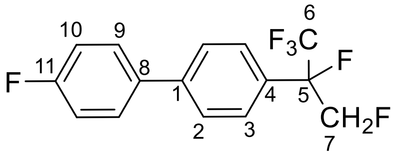

Compound **2j** was prepared according to the General Procedure **D** with an amine:HF ratio of 1:6 using 4-fluoro-4'-(3,3,3-trifluoroprop-1-en-2-yl)-1,1'-biphenyl (**1j**) (133 mg, 0.5 mmol, 1.0 eq.). After workup, the crude mixture was analysed by ^{19}F NMR (77% NMR yield, 7.3:1 *vic:gem*). Purification by column chromatography (10% DCM in *n*-pentane) yielded the title compound as a white solid (84 mg, 0.28 mmol, 55%).

Single crystals suitable for X-ray crystallographic analysis were obtained *via* slow evaporation of a solution of *n*-pentane / methyl *tert*-butyl ether.

The enantiomeric ratio was determined by chiral HPLC with a chiral OM column, *n*-hexane : isopropanol 97.0:3.0, 0.5 mL/min, t_R major = 9.8 min, t_R minor = 10.4 min, *e.r.* = 85:15.

ORD ($CHCl_3$, c 1.00) $[\alpha]_D^{25}$ = +5.995

HPLC trace ±**2j**

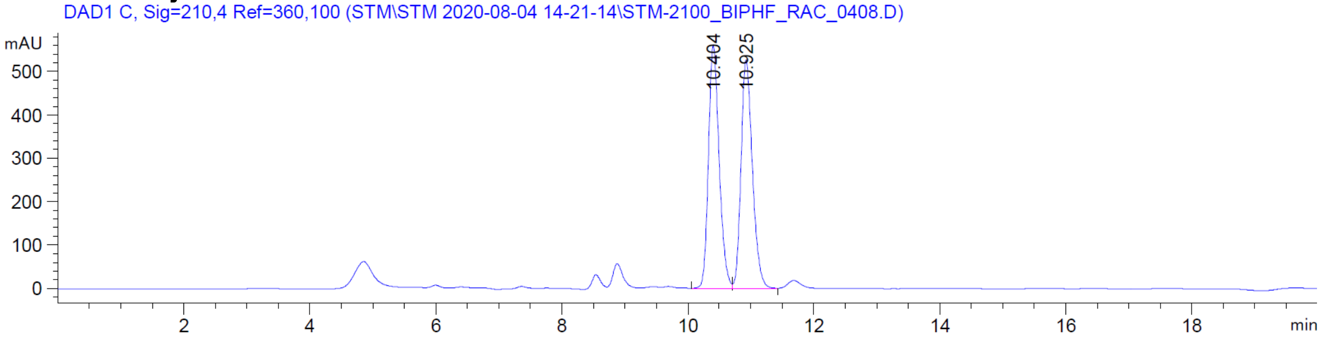

Signal 3: DAD1 C, Sig=210,4 Ref=360,100

Peak #	RetTime [min]	Type	Width [min]	Area [mAU*s]	Height [mAU]	Area %
1	10.404	BV	0.1795	6597.81836	559.67993	49.9910
2	10.925	VV	0.1921	6600.20410	527.18762	50.0090
Totals :				1.31980e4	1086.86755	

HPLC trace (*S*)-**2j**

DAD1 C, Sig=210,4 Ref=360,100 (STM\STM 2020-09-11 13-14-03\STM-2130_PBPHF_ENT.D)

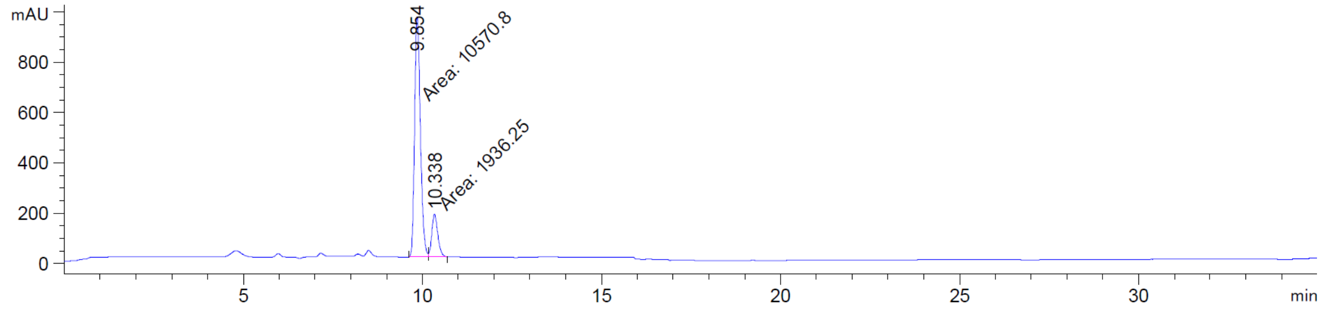

Signal 3: DAD1 C, Sig=210,4 Ref=360,100

Peak #	RetTime [min]	Type	Width [min]	Area [mAU*s]	Height [mAU]	Area %
1	9.854	MF	0.1847	1.05708e4	953.71619	84.5187
2	10.338	FM	0.1913	1936.24792	168.70277	15.4813

Totals : 1.25070e4 1122.41896

HPLC trace (*S*)-**2j** after recrystallisation

DAD1 C, Sig=210,4 Ref=360,100 (STM\STM 2020-11-17 17-31-44\STM-2130_CRYSTAL_5.D)

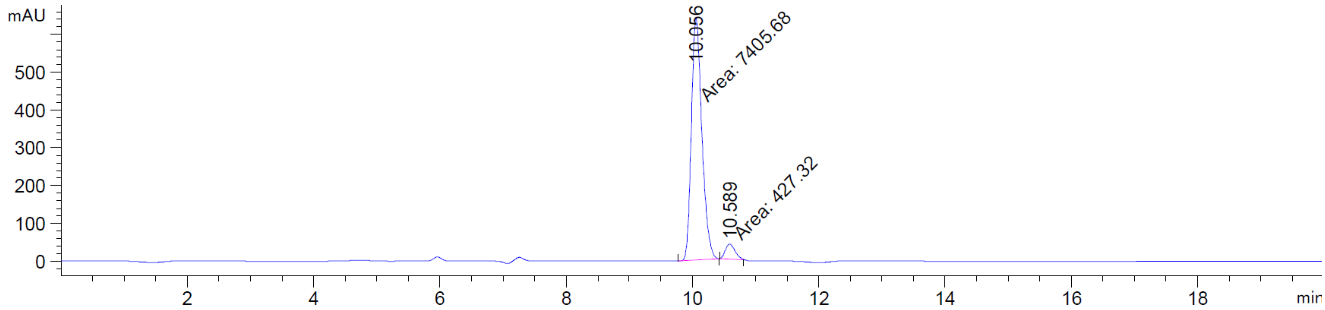

Signal 3: DAD1 C, Sig=210,4 Ref=360,100

Peak #	RetTime [min]	Type	Width [min]	Area [mAU*s]	Height [mAU]	Area %
1	10.056	MM	0.1921	7405.68066	642.67285	94.5446
2	10.589	MM	0.1777	427.31979	40.07500	5.4554

Totals : 7833.00046 682.74785

1-Bromo-4-(1,1,1,2,3-pentafluoropropan-2-yl)benzene (2l)

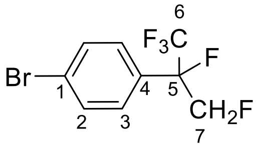

Compound **2l** was prepared according to the General Procedure **E** with an amine:HF ratio of 1:7.5 using 1-bromo-4-(3,3,3-trifluoroprop-1-en-2-yl)benzene (**1l**) (126 mg, 0.5 mmol, 1.0 eq.). After workup, the crude mixture was analysed by ^{19}F NMR (>95% NMR yield, 2.0:1 *vic:gem*). Purification by column chromatography (*n*-pentane) yielded the title compound as a colourless oil (72 mg, 0.25 mmol, 50%). The enantiomeric ratio was determined by chiral HPLC with a chiral OM column, *n*-hexane : isopropanol 99.9:0.1, 0.25 mL/min, t_R major = 35.2 min, t_R minor = 40.8 min, *e.r.* = 87:13.

ORD ($CHCl_3$, c 1.00) $[\alpha]_D^{25}$ = +6.603

HPLC trace ±**2l**

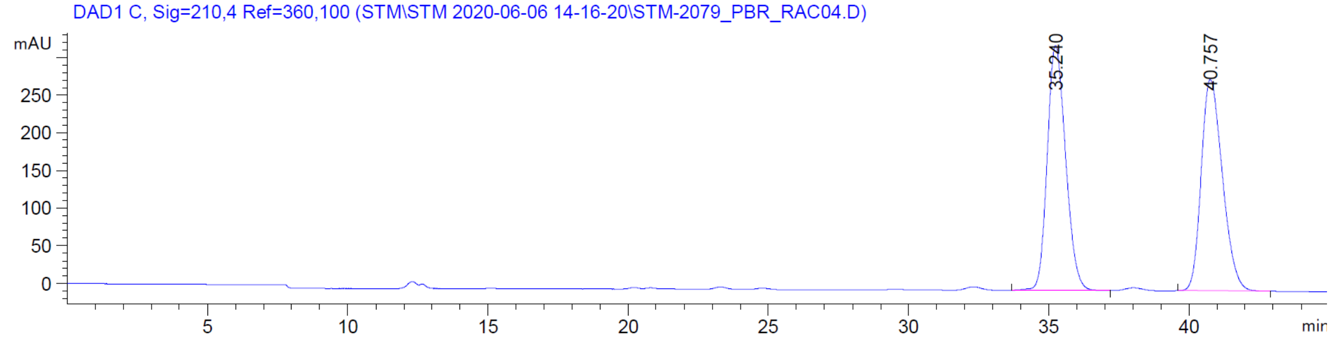

Signal 2: DAD1 C, Sig=210,4 Ref=360,100

Peak #	RetTime [min]	Type	Width [min]	Area [mAU*s]	Height [mAU]	Area %
1	35.240	BB	0.6883	1.48413e4	325.26230	50.1145
2	40.757	BB	0.8063	1.47735e4	280.57080	49.8855
Totals :				2.96148e4	605.83310	

HPLC trace (*S*)-**2l**

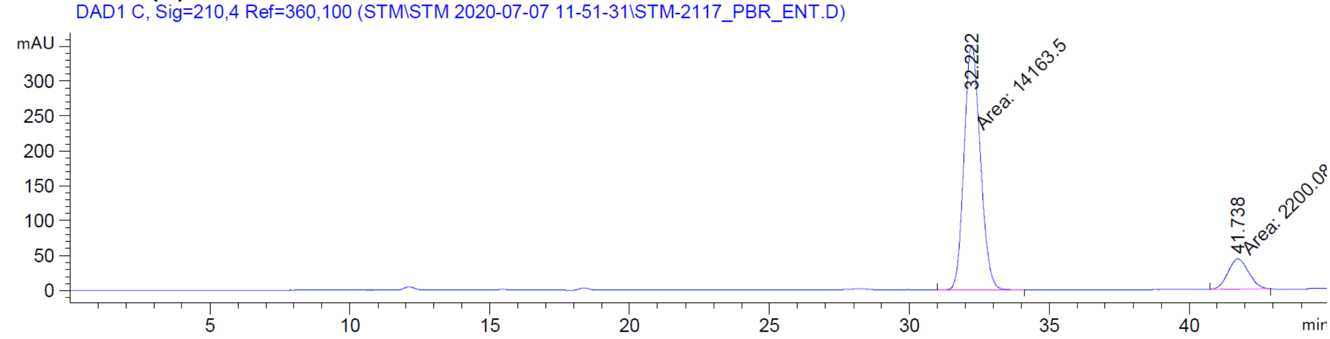

Signal 3: DAD1 C, Sig=210,4 Ref=360,100

Peak #	RetTime [min]	Type	Width [min]	Area [mAU*s]	Height [mAU]	Area %
1	32.222	MM	0.6732	1.41635e4	350.64313	86.5550
2	41.738	MM	0.8503	2200.08447	43.12563	13.4450
Totals :				1.63635e4	393.76875	

1-(Methoxymethyl)-4-(1,1,1,2,3-pentafluoropropan-2-yl)benzene (2m)

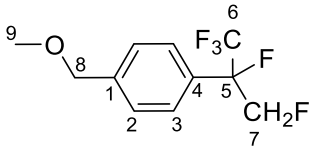

Compound **2m** was prepared according to the General Procedure **E** with an amine:HF ratio of 1:7.5 using 1-(methoxymethyl)-4-(3,3,3-trifluoroprop-1-en-2-yl)benzene (**1m**) (108 mg, 0.5 mmol, 1.0 eq.). After workup, the crude mixture was analysed by ^{19}F NMR (95% NMR yield, 3.1:1 *vic:gem*). Purification by preparative thin layer chromatography (5% Et_2O in *n*-pentane) yielded the title compound as a colourless oil (77 mg, 0.30 mmol, 61%).

The enantiomeric ratio was determined by chiral HPLC with a chiral AS-H column, *n*-hexane : isopropanol 99.9:0.1, 0.5 mL/min, t_R major = 17.7 min, t_R minor = 20.1 min, *e.r.* = 86:14.

ORD ($CHCl_3$, c 1.00) $[\alpha]_D^{25}$ = +5.205.

HPLC trace ±**2m**

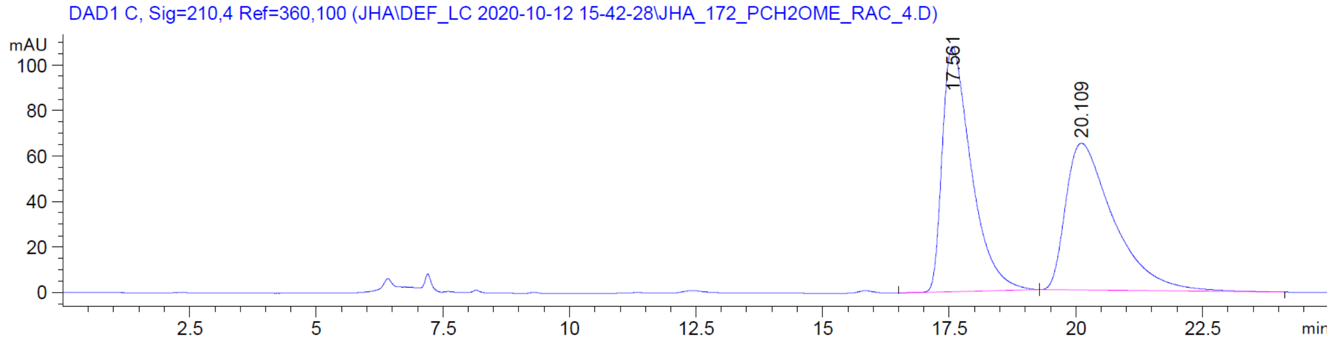

Signal 3: DAD1 C, Sig=210,4 Ref=360,100

Peak #	RetTime [min]	Type	Width [min]	Area [mAU*s]	Height [mAU]	Area %
1	17.561	BB	0.5988	4296.59375	107.84573	50.2473
2	20.109	BBA	0.9705	4254.30615	64.58159	49.7527

Totals : 8550.89990 172.42731

HPLC trace (*S*)-**2m**

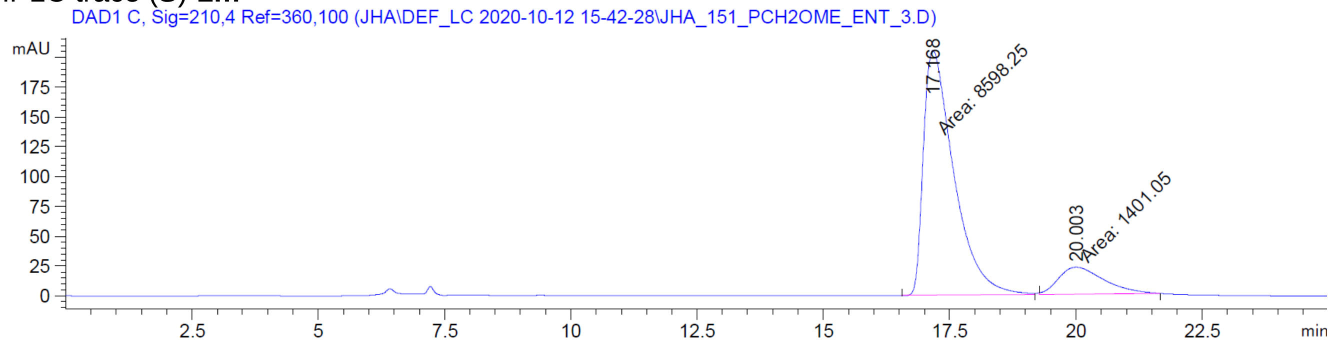

Signal 3: DAD1 C, Sig=210,4 Ref=360,100

Peak #	RetTime [min]	Type	Width [min]	Area [mAU*s]	Height [mAU]	Area %
1	17.168	MM	0.6974	8598.24512	205.48106	85.9885
2	20.003	MM	1.0348	1401.05103	22.56500	14.0115

Totals : 9999.29614 228.04607

2-(4-(1,1,1,2,3-pentafluoropropan-2-yl)phenyl)isoindoline-1,3-dione (2p)

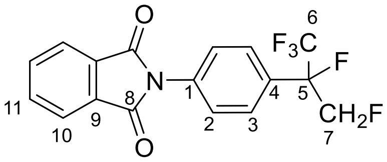

Compound **2p** was prepared according to the General Procedure **E** with an amine:HF ratio of 1:6 and an extended reaction time of 48 h using 2-(4-(3,3,3)-trifluoroprop-1-en-2-yl)phenyl)isoindoline-1,3-dione (**1p**) (159 mg, 0.5 mmol, 1.0 eq.). After workup, the crude mixture was analysed by ^{19}F NMR (90% NMR yield, 2.6:1 *vic:gem*). Purification by column chromatography (25% Et_2O in *n*-pentane) yielded the title compound as a white solid (100 mg, 0.28 mmol, 56%).

Single crystals suitable for X-ray crystallographic analysis were obtained *via* slow evaporation of a solution of *n*-pentane / EtOAc.

The enantiomeric ratio was determined by chiral HPLC with a chiral OJ-H column, *n*-hexane : isopropanol 85.0:15.0, 0.5 mL/min, t_R major = 54.2 min, t_R minor = 63.5 min, *e.r.* = 87:13.

ORD ($CHCl_3$, c 1.00) $[\alpha]_D^{25}$ = +4.766.

HPLC trace racemic sample **2p**

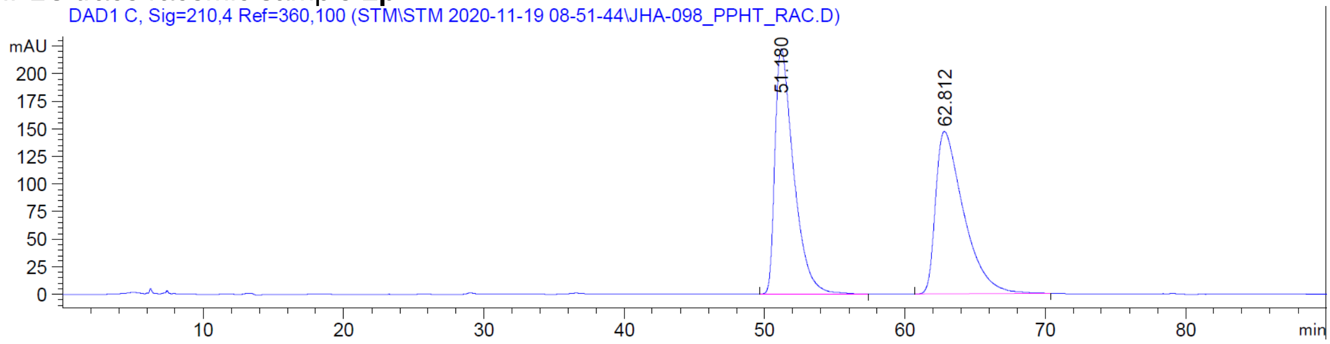

Signal 3: DAD1 C, Sig=210,4 Ref=360,100

Peak #	RetTime [min]	Type	Width [min]	Area [mAU*s]	Height [mAU]	Area %
1	51.180	BB	1.3731	2.06359e4	222.28726	50.1626
2	62.812	BB	1.9872	2.05021e4	147.17236	49.8374

Totals : 4.11379e4 369.45963

HPLC trace chiral sample **2p**

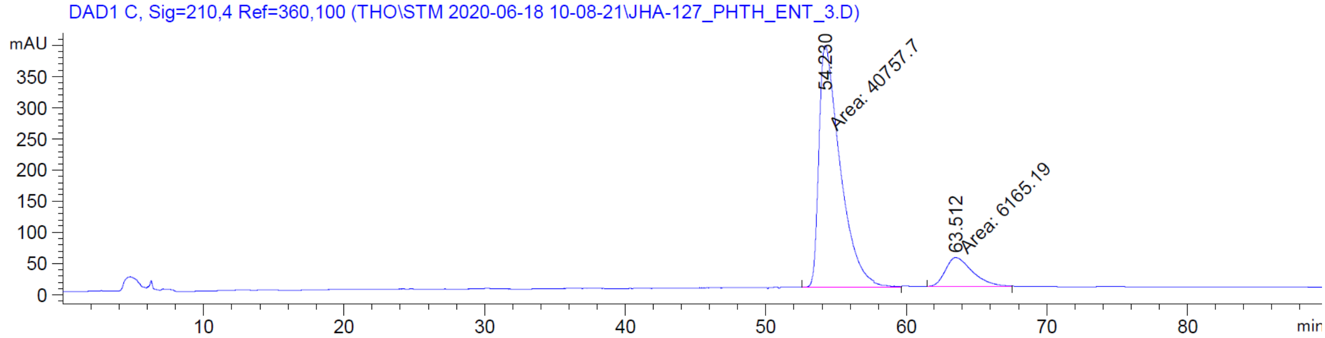

Signal 3: DAD1 C, Sig=210,4 Ref=360,100

Peak #	RetTime [min]	Type	Width [min]	Area [mAU*s]	Height [mAU]	Area %
1	54.230	MM	1.7532	4.07577e4	387.46094	86.8610
2	63.512	MM	2.2305	6165.18506	46.06723	13.1390
Totals :				4.69229e4	433.52816	

HPLC trace chiral sample **2p** after recrystallisation

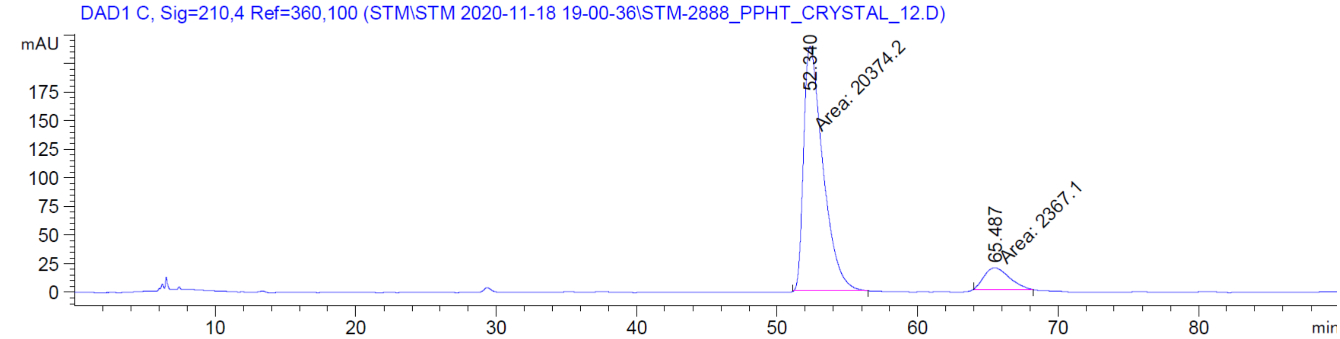

Signal 3: DAD1 C, Sig=210,4 Ref=360,100

Peak #	RetTime [min]	Type	Width [min]	Area [mAU*s]	Height [mAU]	Area %
1	52.340	MM	1.5889	2.03742e4	213.70976	89.5912
2	65.487	MM	2.0805	2367.10278	18.96295	10.4088
Totals :				2.27413e4	232.67271	

2.7. Synthesis of the Chiral Analogue of a TRPA1 Antagonist

2-(1,3-Dimethyl-2,6-dioxo-1,2,3,6-tetrahydro-7H-purin-7-yl)-N-(4-(1,1,1,2,3-pentafluoropropan-2-yl)phenyl)acetamide (2r)

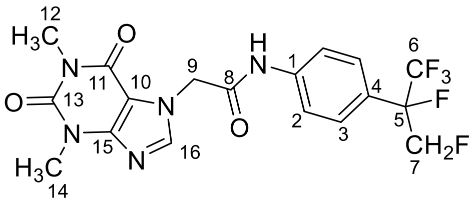

To a stirred solution of **2p** (71.1 mg, 0.2 mmol, 1 eq.) in Et_2O (4 mL, 0.05 M), hydrazine monohydrate (64% N_2H_4, 23 µL, 0.3 mmol, 1.5 eq.) was added. The reaction mixture was stirred for 2 h at ambient temperature forming a white precipitate. After completion, the crude mixture was filtered through a pad of silica, eluting the aniline intermediate with Et_2O/*n*-pentane (1:1, 100 mL). Solvent was removed under reduced pressure before the addition of theophylline-7-acetic acid (47.6 mg, 0.2 mmol, 1 eq.) and DCM (666 µL, 0.3 M). The reaction mixture was cooled to 0 °C before the addition of DCC (43.3 mg, 0.21 mmol, 1.05 eq.) and DMAP (1.2 mg, 0.01 mmol, 5 mol%). After stirring at 0 °C for 1 h, the reaction was allowed to warm to ambient temperature and was stirred for 3 h. After completion, the reaction mixture was filtered, washing the filter cake with DCM. The filtrate was concentrated under reduced pressure and the crude

residue was purified by column chromatography (0-3% MeOH in DCM) to afford the desired product as a white solid (17.4 mg, 20% over 2 steps).

ORD ($CHCl_3$, c 0.50) $[\alpha]_D^{25}$ = +4.177

R_f = 0.31 (5% MeOH in DCM)

M.p.: 260 °C – 262 °C (decomposition).

FT-IR ($\tilde{\nu}$ = cm^{-1}): 2928 (w), 1704 (m), 1658 (s), 1609 (w), 1550 (m), 1474 (w), 1197 (m), 763 (m).

^{1}H NMR (599 MHz, $CDCl_3$): δ [ppm] = 9.84 (s, 1H, H-N), 7.77 (s, 1H, H-C16), 7.64 (m, 2H, H-C3), 7.44 (m, 2H, H-C2), 4.97 (s, 2H, H-C9), 4.95 – 4.86 (m, 1H, H^a-C7), 4.85 – 4.74 (m, 1H, H^b-C7), 3.61 (s, 3H, H-C14), 3.47 (d, J = 1.4 Hz, 3H, H-C12).

^{13}C{^{1}H} NMR (151 MHz, $CDCl_3$): δ [ppm] = 163.6 (C8), 156.8 (C11), 151.3 (C13), 149.9 (C15), 142.6 (C16), 139.2 (C1), 126.8 (d, $^3J_{CF}$ = 9.7 Hz, C3), 126.5 (dd, $^2J_{CF}$ = 21.6 Hz, $^3J_{CF}$ = 2.9 Hz, C4), 122.4 (d, $^1J_{CF}$ = 285.5 Hz, C6), 119.9 (d, $^4J_{CF}$ = 2.0 Hz, C2), 106.8 (C10), 92.8 (d, $^2J_{CF}$ = 31.0 Hz, C5), 81.5 (dd, $^1J_{CF}$ = 185.7 Hz, $^2J_{CF}$ = 23.7 Hz, C7), 52.1 (C9), 30.2 (C14), 28.5 (C12).

^{19}F NMR (470 MHz, $CDCl_3$): δ [ppm] = 77.5 (m, 3F, F-C6), -179.1 (m, 1F, F-C5), -234.0 (m, 1F, F-C7).

^{19}F{^{1}H} NMR (470 MHz, $CDCl_3$): δ [ppm] = 77.5 (dd, $^4J_{FF}$ = 8.3, $^3J_{FF}$ = 7.1 Hz, 3F, F-C6), -179.1 (dq, $^3J_{FF}$ = 14.1 Hz, $^3J_{FF}$ = 7.1 Hz, 1F, F-C5), -234.0 (dq, $^3J_{FF}$ = 13.4 Hz, $^4J_{FF}$ = 8.3 Hz, 1F, F-C6).

ESI-MS: (*m/z*) requires: $[(C_{18}H_{16}N5O_3F_5)Na]^+$ = 468.10655, (*m/z*) found: $[(C_{18}H_{16}N5O_3F_5)Na]^+$ = 468.10631.

3. X-ray Crystallographic Analysis

X-Ray diffraction: Data sets for compounds **2j**, **2p** and **10** were collected with a Bruker D8 Venture CMOS diffractometer. Programs used: data collection: APEX3 V2016.1-0 (Bruker AXS Inc., **2016**); cell refinement: SAINT V8.37A (Bruker AXS Inc., **2015**); data reduction: SAINT V8.37A (Bruker AXS Inc., **2015**); absorption correction, SADABS V2014/7 (Bruker AXS Inc., **2014**); structure solution *SHELXT-2015* (Sheldrick, G. M. *Acta Cryst.,* **2015**, *A71*, 3-8); structure refinement *SHELXL-2015* (Sheldrick, G. M. *Acta Cryst.,* **2015**, *C71* (1), 3-8) and graphics, *XP* (Version 5.1, Bruker AXS Inc., Madison, Wisconsin, USA, **1998**). *R*-values are given for observed reflections, and wR^2 values are given for all reflections.
Exceptions and special features: For compound **2p** four independent molecules with chirality center *S* at C2 atom were found in the asymmetric unit. For molecule numbered with suffix "B" a disorder over two positions of the substituents at C2 atom was found. Due to this disorder, a mixture of enantiomers *S* and *R* in ration 75:25 was observed. For others three independent molecules only the *S* enantiomer configuration was found.

X-ray crystal structure analysis of 2j (gil9953): A colorless needle-like specimen of $C_{15}H_{10}F_6$, approximate dimensions 0.040 mm x 0.120 mm x 0.255 mm, was used for the X-ray crystallographic analysis. The X-ray intensity data were measured on a Bruker D8 Venture PHOTON III Diffractometer system equipped with a micro focus tube Cu Ims (CuK_α, λ = 1.54178 Å) and a MX mirror monochromator. A total of 844 frames were collected. The total exposure time was 15.39 hours. The frames were integrated with the Bruker SAINT software package using a wide-frame algorithm. The integration of the data using an orthorhombic unit cell yielded a total of 7710 reflections to a maximum θ angle of 65.76° (0.85 Å resolution), of which 2167 were independent (average redundancy 3.558, completeness = 99.9%, R_{int} = 10.78%, R_{sig} = 8.68%) and 1278 (58.98%) were greater than $2\sigma(F^2)$. The final cell constants of a = 5.6627(3) Å, b = 7.4327(4) Å, c = 29.6883(18) Å, volume = 1249.56(12) $Å^3$, are based upon the refinement of the XYZ-centroids of 1769 reflections above 20 σ(I) with 5.953° < 2θ < 126.4°. Data were corrected for absorption effects using the Multi-Scan method (SADABS). The ratio of minimum to maximum apparent transmission was 0.735. The calculated minimum and maximum transmission coefficients (based on crystal size) are 0.7180 and 0.9460. The structure was solved and refined using the Bruker SHELXTL Software Package, using the space group $P2_12_12_1$, with Z = 4 for the formula unit, $C_{15}H_{10}F_6$. The final anisotropic full-matrix least-squares refinement on F^2 with 190 variables converged at R1 = 6.35%, for the observed data and wR2 = 19.07% for all data. The goodness-of-fit was 0.994. The largest peak in the final difference electron density synthesis was 0.354 $e^-/Å^3$ and the largest hole was -0.231 $e^-/Å^3$ with an RMS deviation of 0.062 $e^-/Å^3$. On the basis of the final model, the calculated density was 1.617 g/cm^3 and F(000), 616 e^-. Flack parameter was refined to 0.0(3). CCDC number: 2044630.

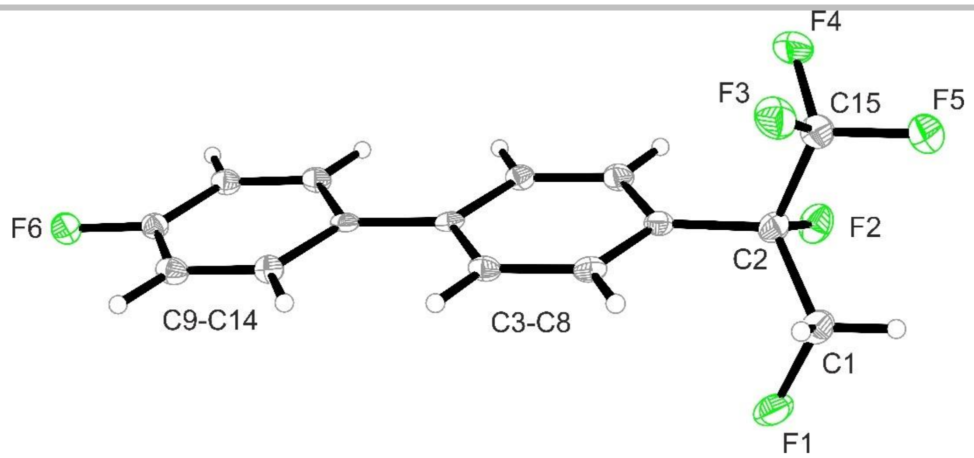

Figure S1: Crystal structure of compound **2j**.
Thermal ellipsoids are shown at 15% probability.

X-ray crystal structure analysis of 2p (gil9947): A colorless plate-like specimen of $C_{17}H_{10}F_5NO_2$, approximate dimensions 0.045 mm x 0.071 mm x 0.151 mm, was used for the X-ray crystallographic analysis. The X-ray intensity data were measured on a Bruker D8 Venture PHOTON III Diffractometer system equipped with a micro focus tube Cu Ims (CuK_α, λ = 1.54178 Å) and a MX mirror monochromator. A total of 2304 frames were collected. The total exposure time was 28.26 hours. The frames were integrated with the Bruker SAINT software package using a wide-frame algorithm. The integration of the data using a triclinic unit cell yielded a total of 49707 reflections to a maximum θ angle of 66.66° (0.84 Å resolution), of which 10244 were independent (average redundancy 4.852, completeness = 99.6%, R_{int} = 5.18%, R_{sig} = 3.73%) and 8996 (87.82%) were greater than 2σ(F^2). The final cell constants of a = 10.7563(2) Å, b = 10.9611(2) Å, c = 13.7042(2) Å, α = 101.2210(10)°, β = 103.5050(10)°, γ = 100.9730(10)°, volume = 1493.19(5) $Å^3$, are based upon the refinement of the XYZ-centroids of 9536 reflections above 20 σ(I) with 6.851° < 2θ < 133.1°. Data were corrected for absorption effects using the Multi-Scan method (SADABS). The ratio of minimum to maximum apparent transmission was 0.863. The calculated minimum and maximum transmission coefficients (based on crystal size) are 0.8290 and 0.9440. The structure was solved and refined using the Bruker SHELXTL Software Package, using the space group *P*1, with Z = 4 for the formula unit, $C_{17}H_{10}F_5NO_2$. The final anisotropic full-matrix least-squares refinement on F^2 with 974 variables converged at R1 = 3.89%, for the observed data and wR2 = 10.08% for all data. The goodness-of-fit was 1.038. The largest peak in the final difference electron density synthesis was 0.453 $e^-/Å^3$ and the largest hole was -0.220 $e^-/Å^3$ with an RMS deviation of 0.039 $e^-/Å^3$. On the basis of the final model, the calculated density was 1.580 g/cm^3 and F(000), 720 e^-. Flack parameter was refined to 0.03(7). CCDC number: 2044631.

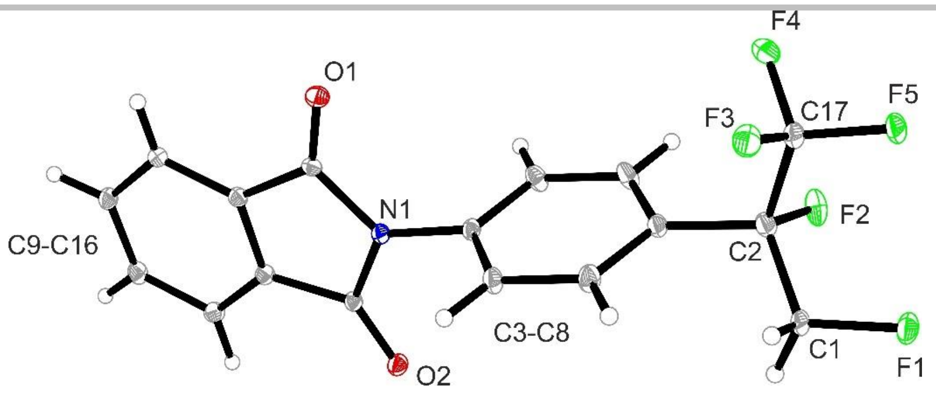

Figure S2: Crystal structure of compound **2p**. Only one molecule (molecule "A") of four found in the asymmetric unit is shown. Thermal ellipsoids are shown at 15% probability.

X-ray crystal structure analysis of 10 (gil9505): A colorless needle-like specimen of $C_{16}H_{19}IO_8$, approximate dimensions 0.064 mm x 0.114 mm x 0.238 mm, was used for the X-ray crystallographic analysis. The X-ray intensity data were measured. A total of 704 frames were collected. The total exposure time was 8.04 hours. The frames were integrated with the Bruker SAINT software package using a wide-frame algorithm. The integration of the data using an orthorhombic unit cell yielded a total of 16319 reflections to a maximum θ angle of 70.16° (0.82 Å resolution), of which 3376 were independent (average redundancy 4.834, completeness = 97.5%, R_{int} = 9.00%, R_{sig} = 6.70%) and 3348 (99.17%) were greater than 2σ(F^2). The final cell constants of a = 17.9429(5) Å, b = 9.6346(3) Å, c = 10.5858(3) Å, volume = 1830.00(9) $Å^3$, are based upon the refinement of the XYZ-centroids of 9982 reflections above 20 σ(I) with 9.179° < 2θ < 140.3°. Data were corrected for absorption effects using the multi-scan method (SADABS). The ratio of minimum to maximum apparent transmission was 0.663. The calculated minimum and maximum transmission coefficients (based on crystal size) are 0.1340 and 0.4650. The structure was solved and refined using the Bruker SHELXTL Software Package, using the space group $P2_12_12$, with Z = 4 for the formula unit, $C_{16}H_{19}IO_8$. The final anisotropic full-matrix least-squares refinement on F^2 with 231 variables converged at R1 = 8.21%, for the observed data and wR2 = 20.58% for all data. The goodness-of-fit was 1.078. The largest peak in the final difference electron density synthesis was 1.889 $e^-/Å^3$ and the largest hole was -2.587 $e^-/Å^3$ with an RMS deviation of 0.237 $e^-/Å^3$. On the basis of the final model, the calculated density was 1.692 g/cm^3 and F(000), 928 e^-. Flack parameter was refined to 0.01(3). CCDC number: 2047146.

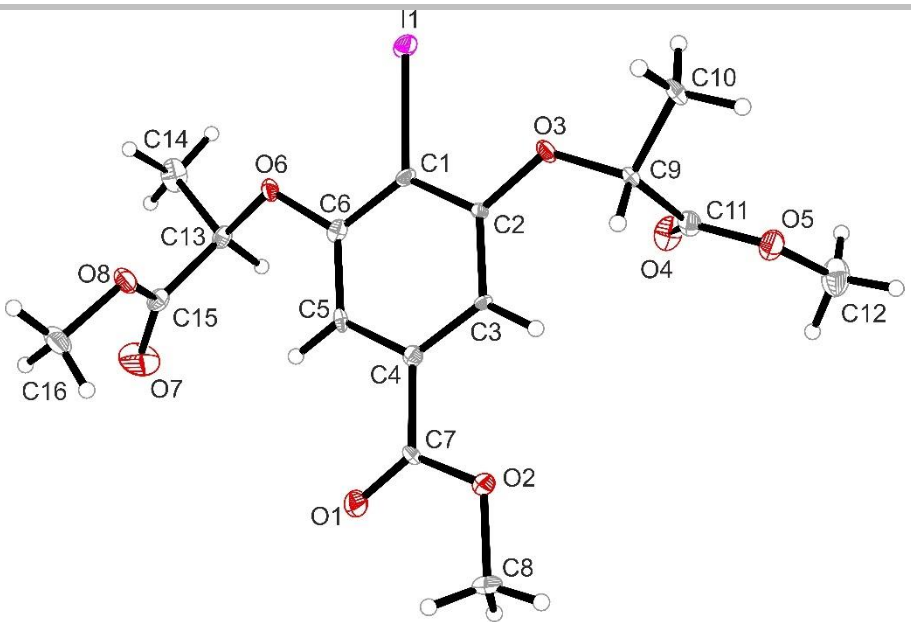

Figure S3: Crystal structure of compound **10**.

Thermal ellipsoids are shown at 30% probability.

1. *APEX3* (**2016**), *SAINT* (**2015**) and *SADABS* (**2015**), Bruker AXS Inc., Madison, Wisconsin, USA.
2. Sheldrick, G. M., *SHELXT – Integrated space-group and crystal-structure determination*, *Acta Cryst.,* **2015**, *A71*, 3-8.
3. Sheldrick, G.M., *Crystal structure refinement with SHELXL*, *Acta Cryst.*, **2015**, *C71 (1)*, 3-8.
4. *XP – Interactive molecular graphics, Version 5.1,* Bruker AXS Inc., Madison, Wisconsin, USA, **1998**.

4. NMR-Spectra of Key Compounds

NMR of Starting Material

2-Methoxy-1,3-dimethyl-5-(3,3,3-trifluoroprop-1-en-2-yl)benzene (1c)

^{1}H NMR (599 MHz, $CDCl_3$)

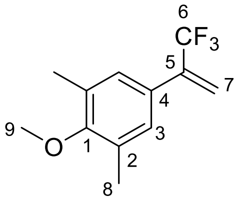

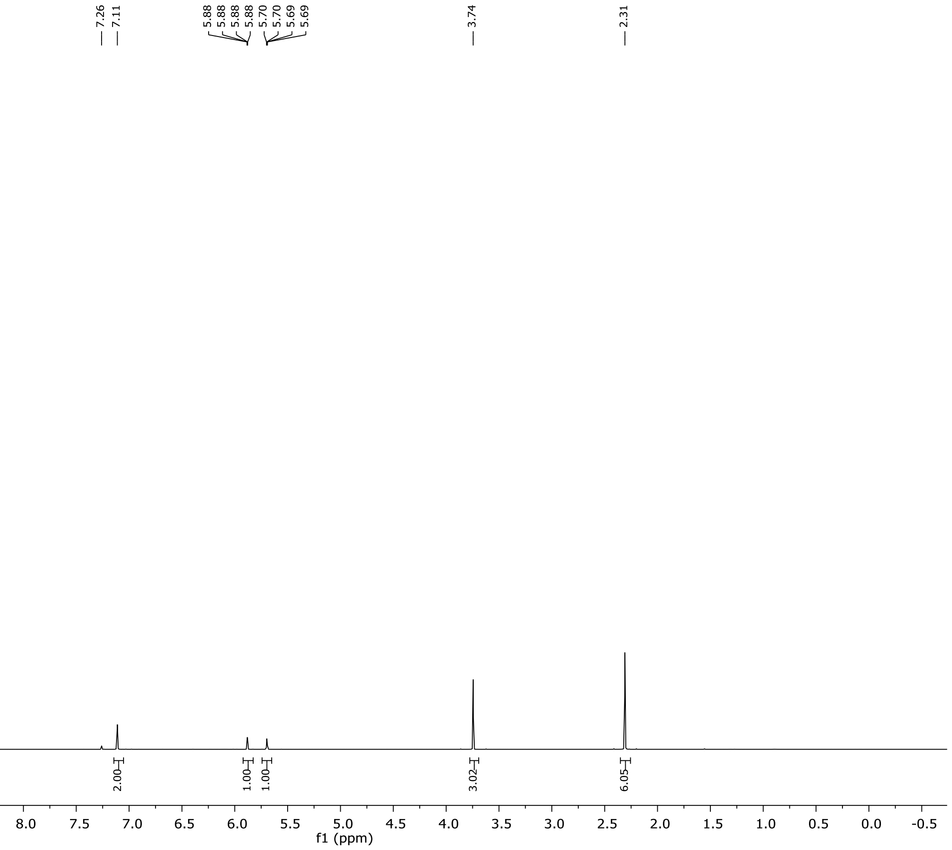

^{13}C NMR (151 MHz, $CDCl_3$)

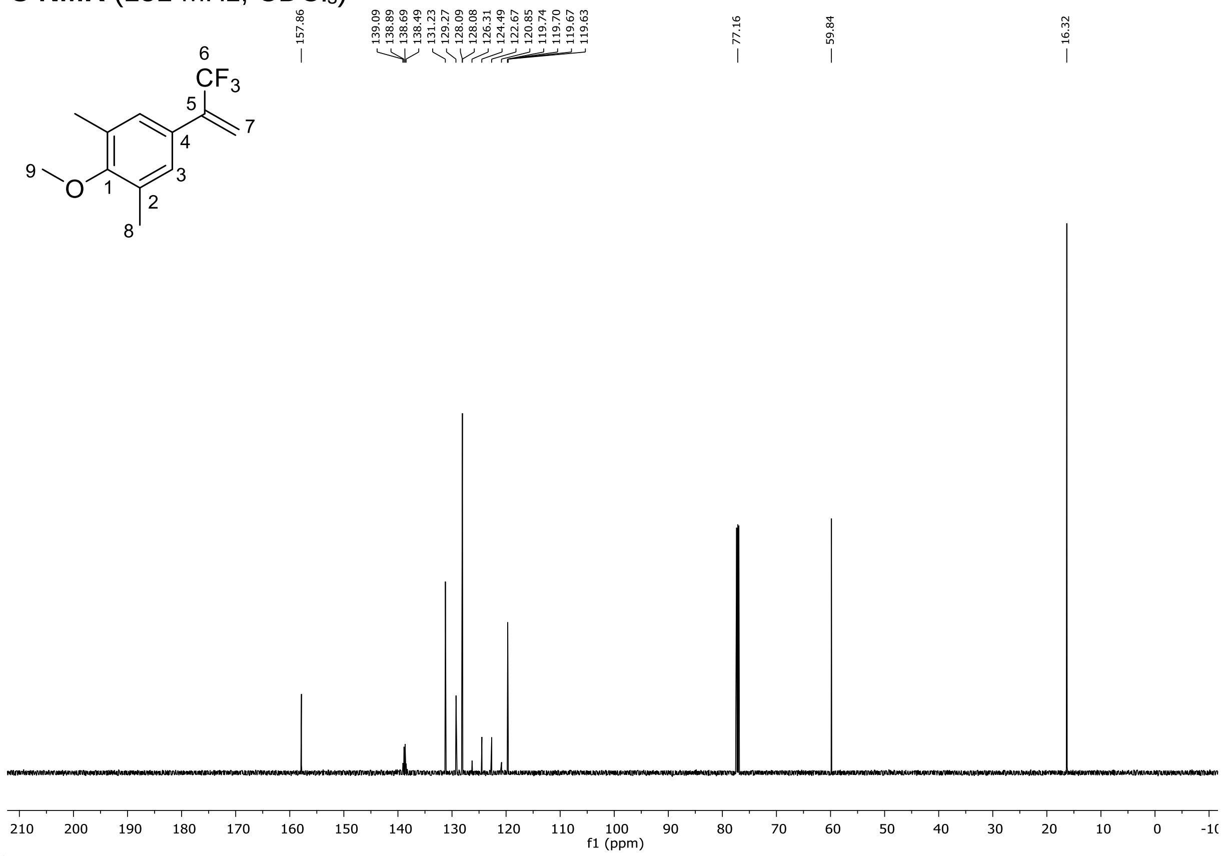

^{19}F{^{1}H} NMR (376 MHz, $CDCl_3$)

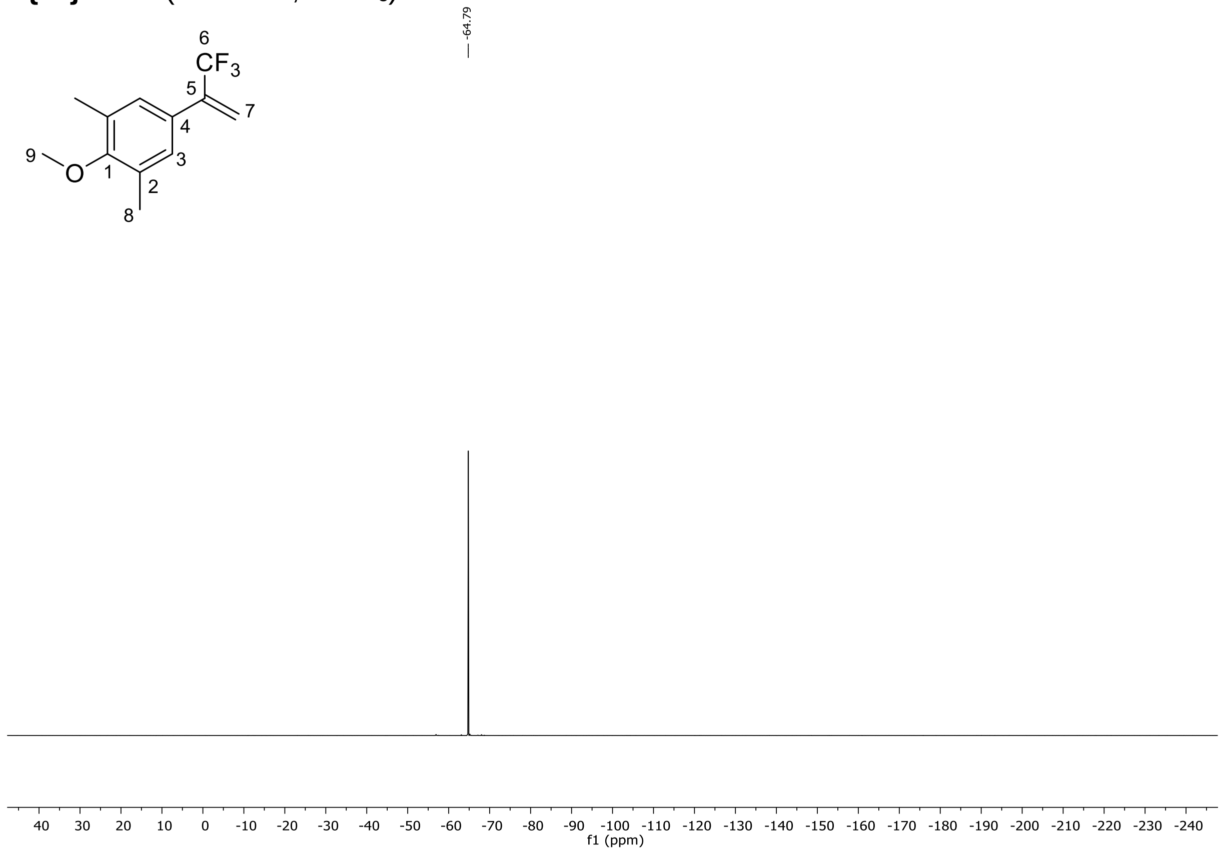

1-Bromo-4-((4-(3,3,3-trifluoroprop-1-en-2-yl)phenoxy)methyl)benzene (1d)

^{1}H NMR (599 MHz, $CDCl_3$)

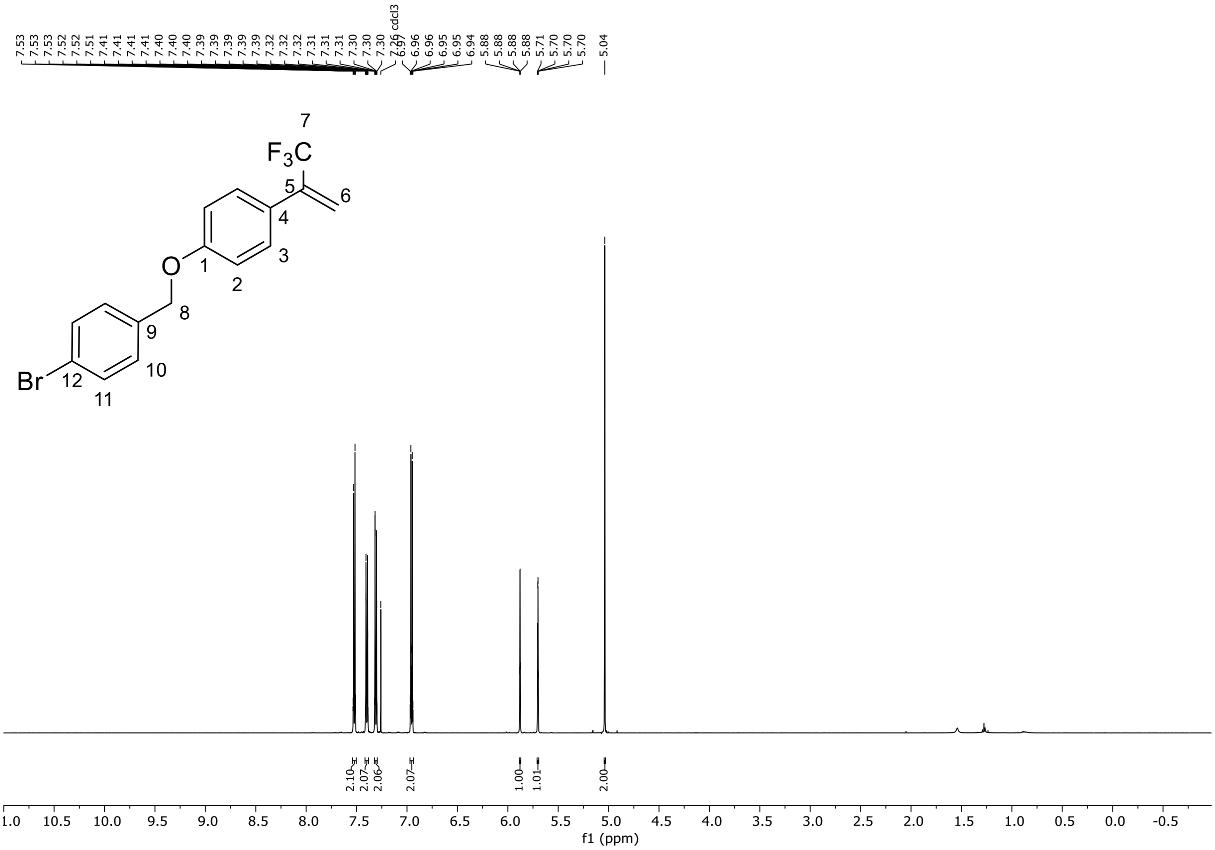

^{13}C NMR (126 MHz, $CDCl_3$)

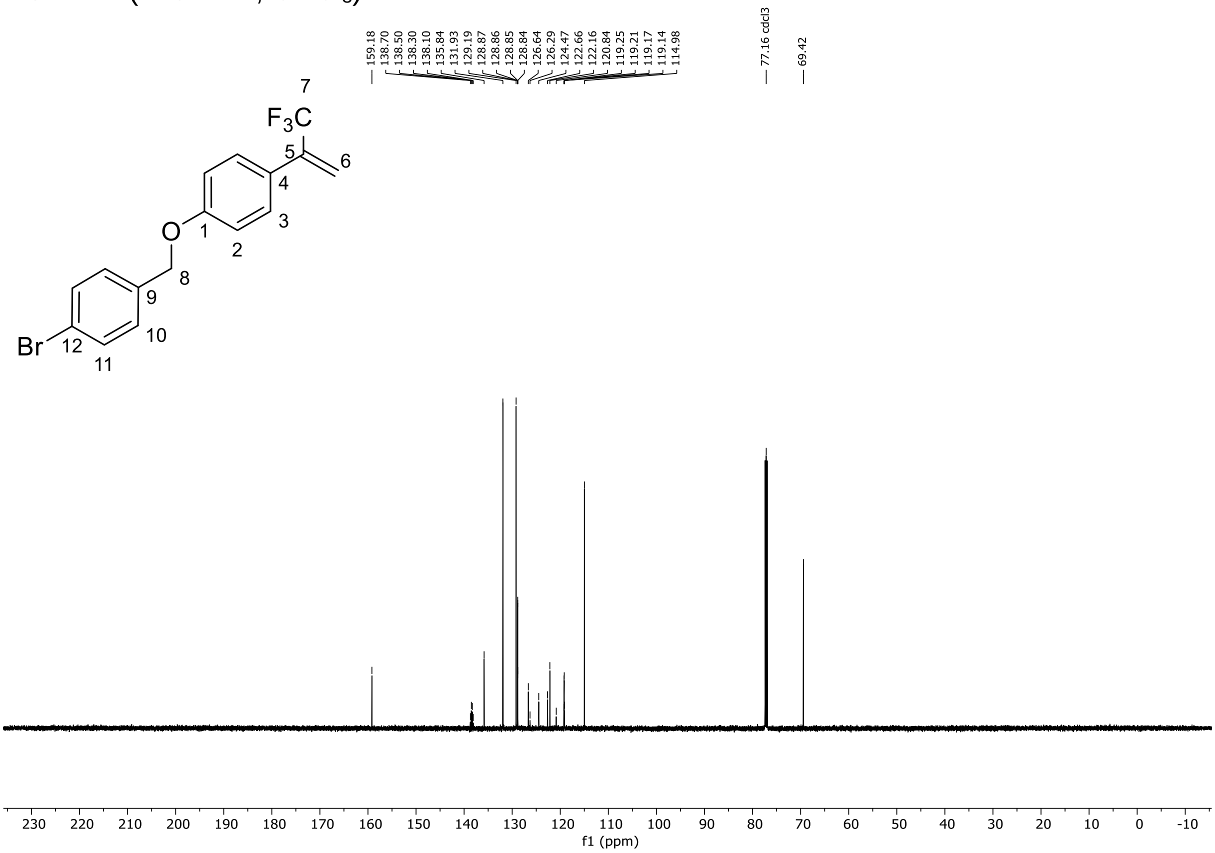

$^{19}F\{^{1}H\}$ NMR (376 MHz, $CDCl_3$)

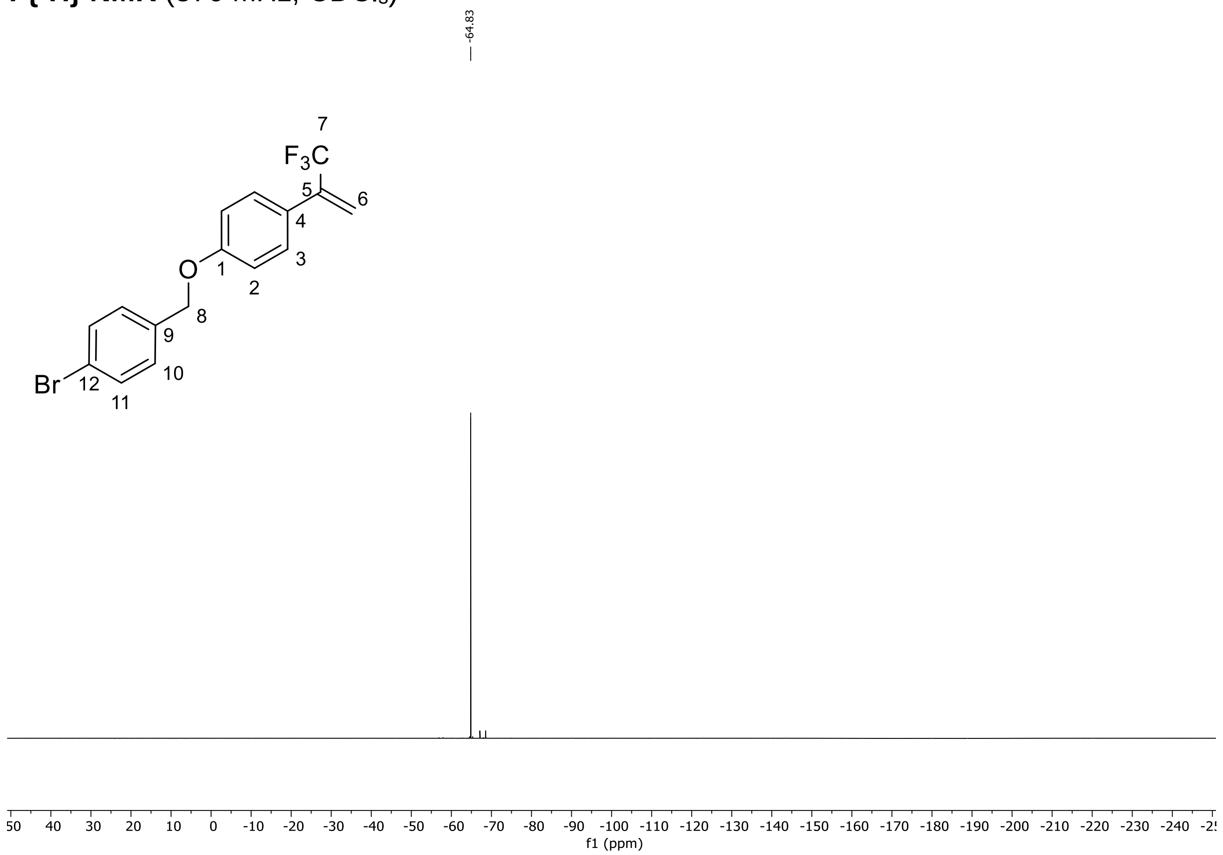

1-Cyclopropyl-4-(3,3,3-trifluoroprop-1-en-2-yl)benzene (1h)

^{1}H NMR (599 MHz, $CDCl_3$)

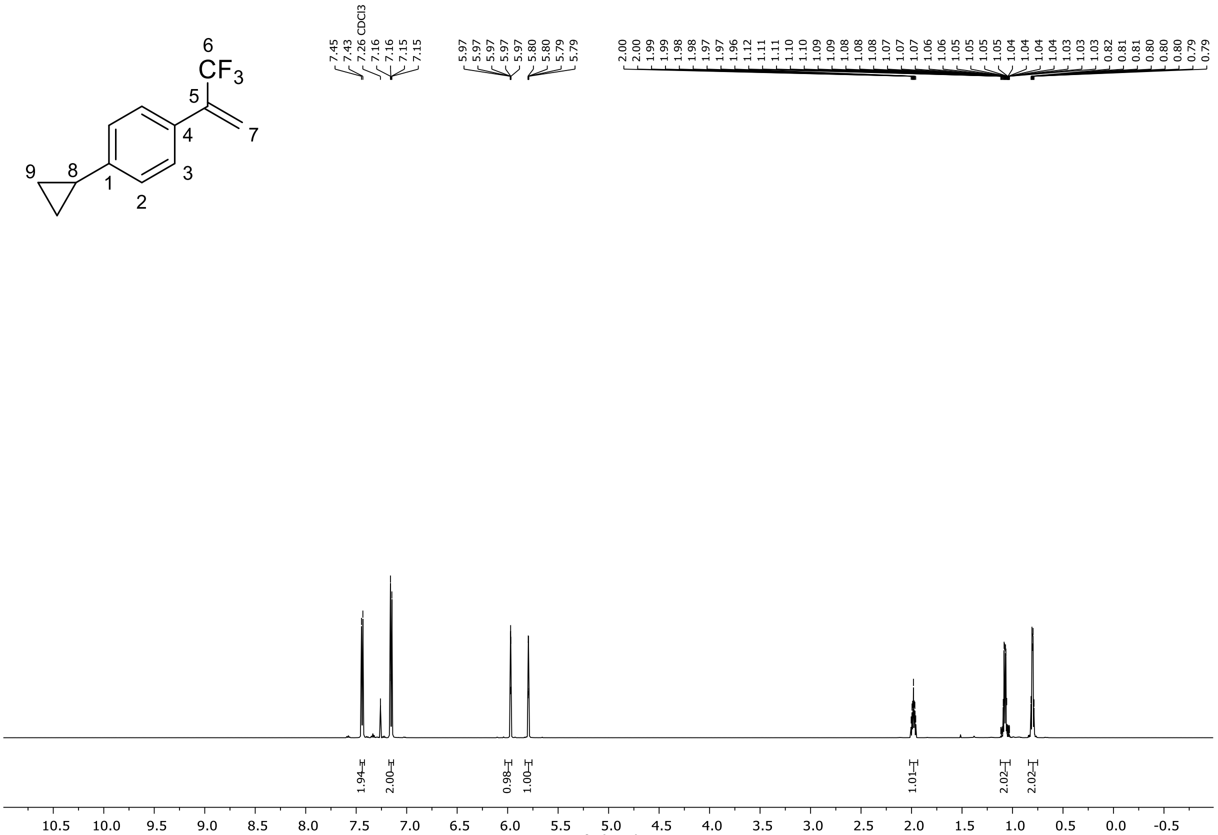

SUPPORTING INFORMATION

^{13}C NMR (151 MHz, $CDCl_3$)

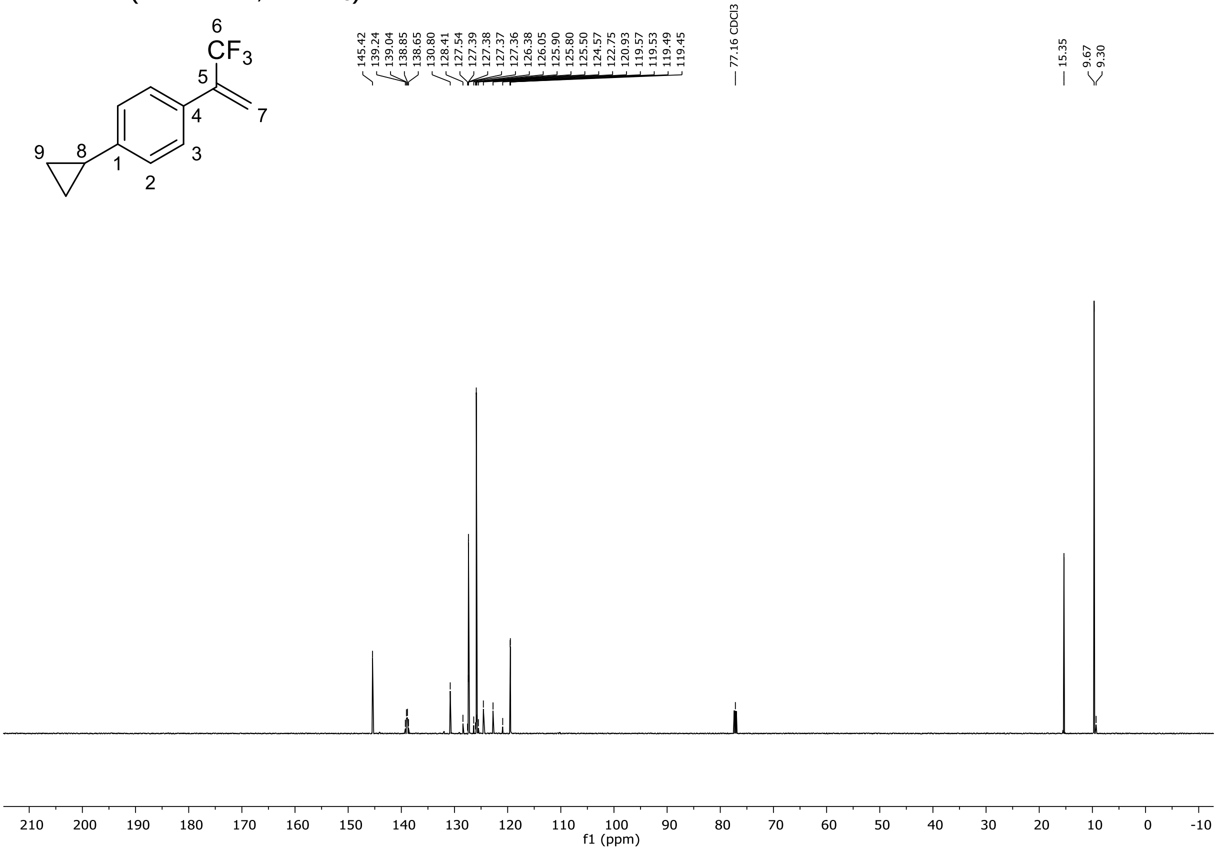

^{19}F{^{1}H} NMR (376 MHz, $CDCl_3$)

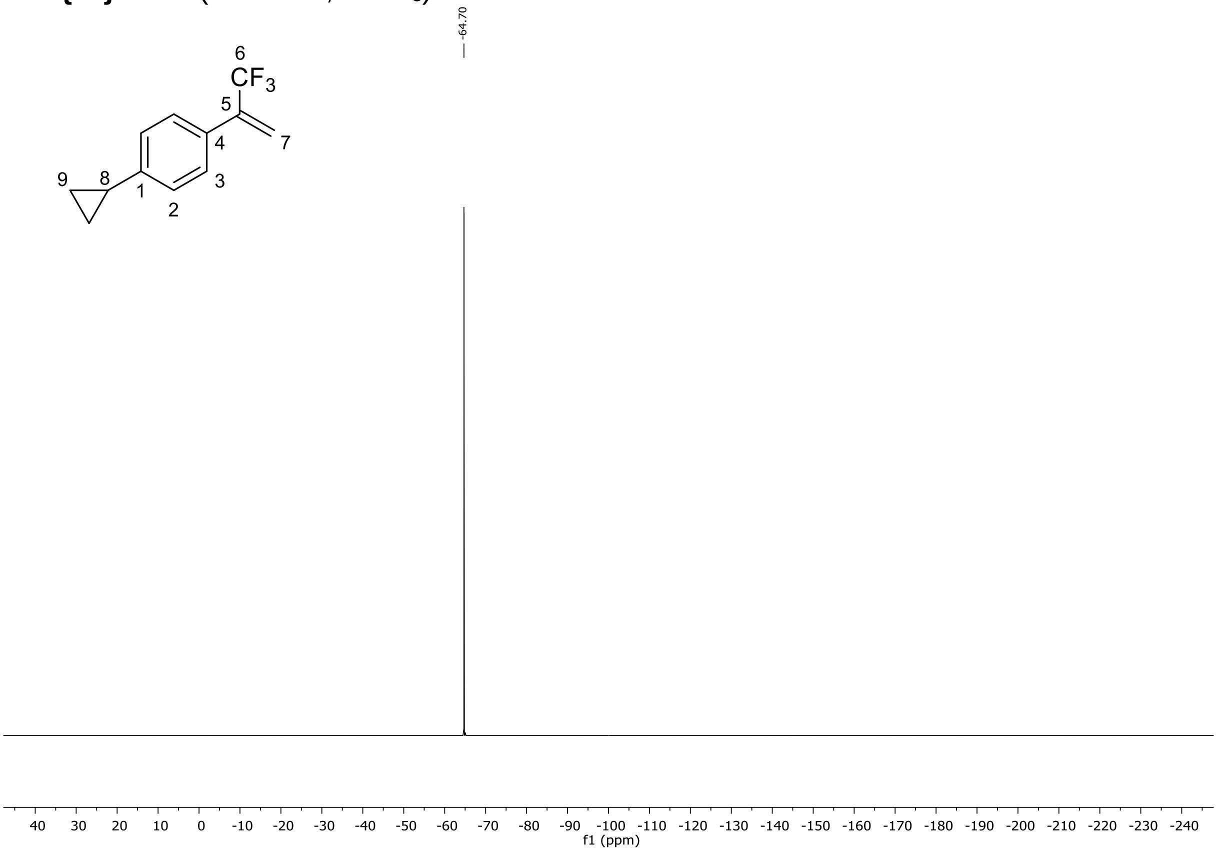

4-Methyl-*N*-(4-(3,3,3-trifluoroprop-1-en-2-yl)phenyl)benzenesulfonamide (S21)

^{1}H NMR (599 MHz, $CDCl_3$)

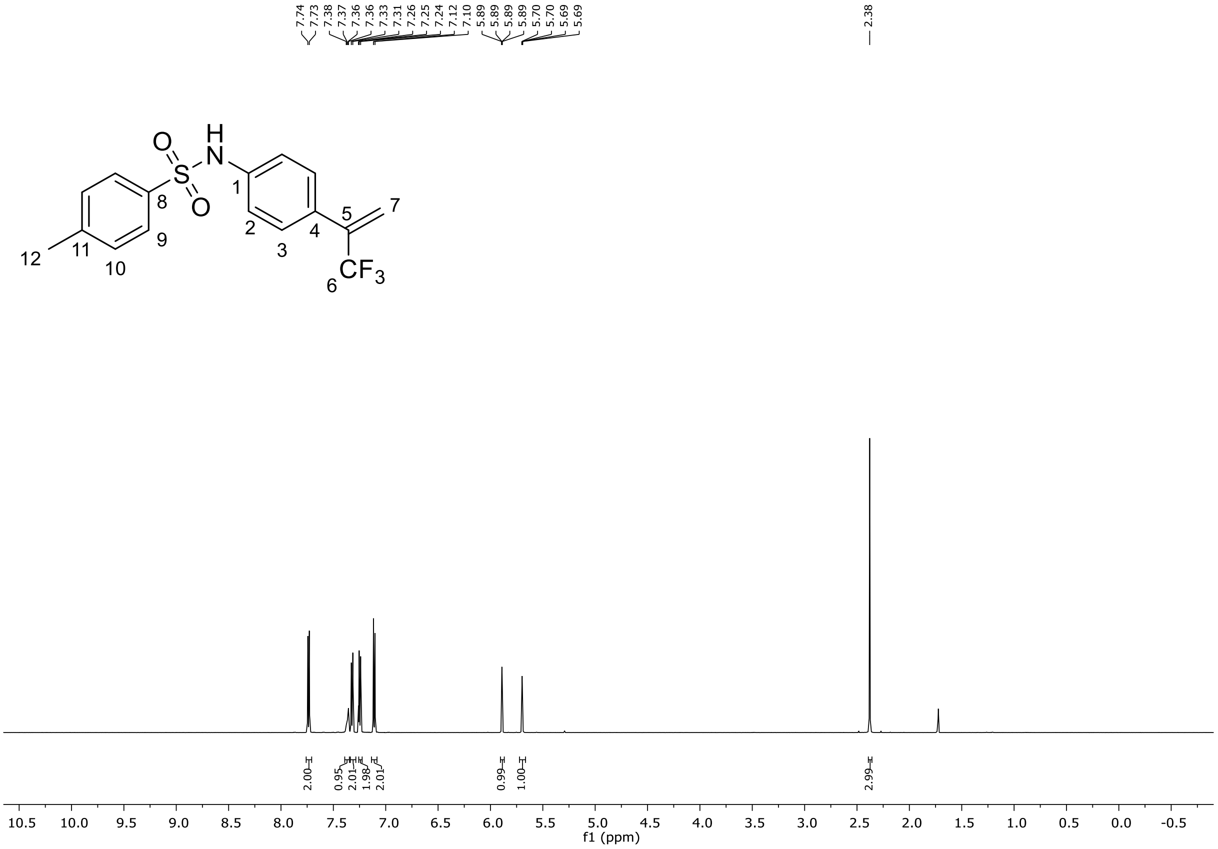

^{13}C NMR (151 MHz, $CDCl_3$)

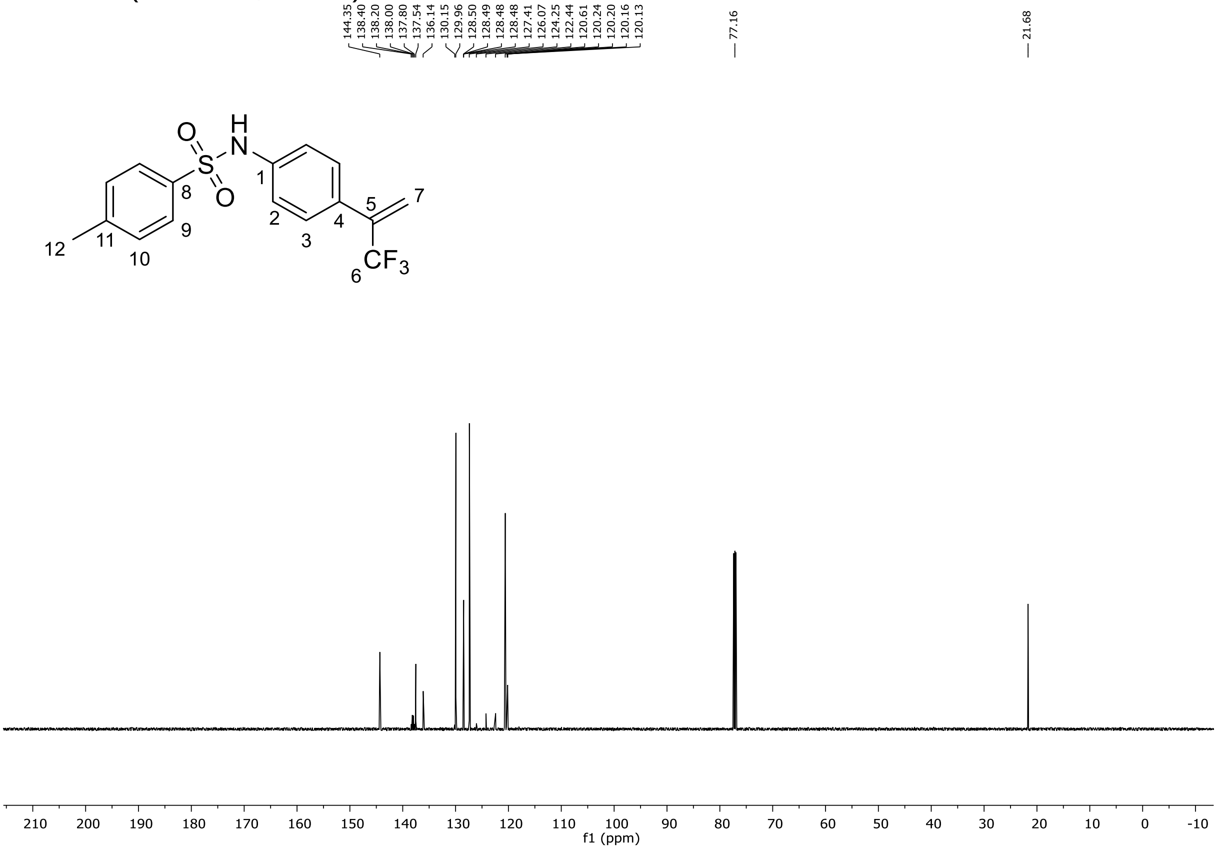

$^{19}F\{^1H\}$ NMR (376 MHz, $CDCl_3$)

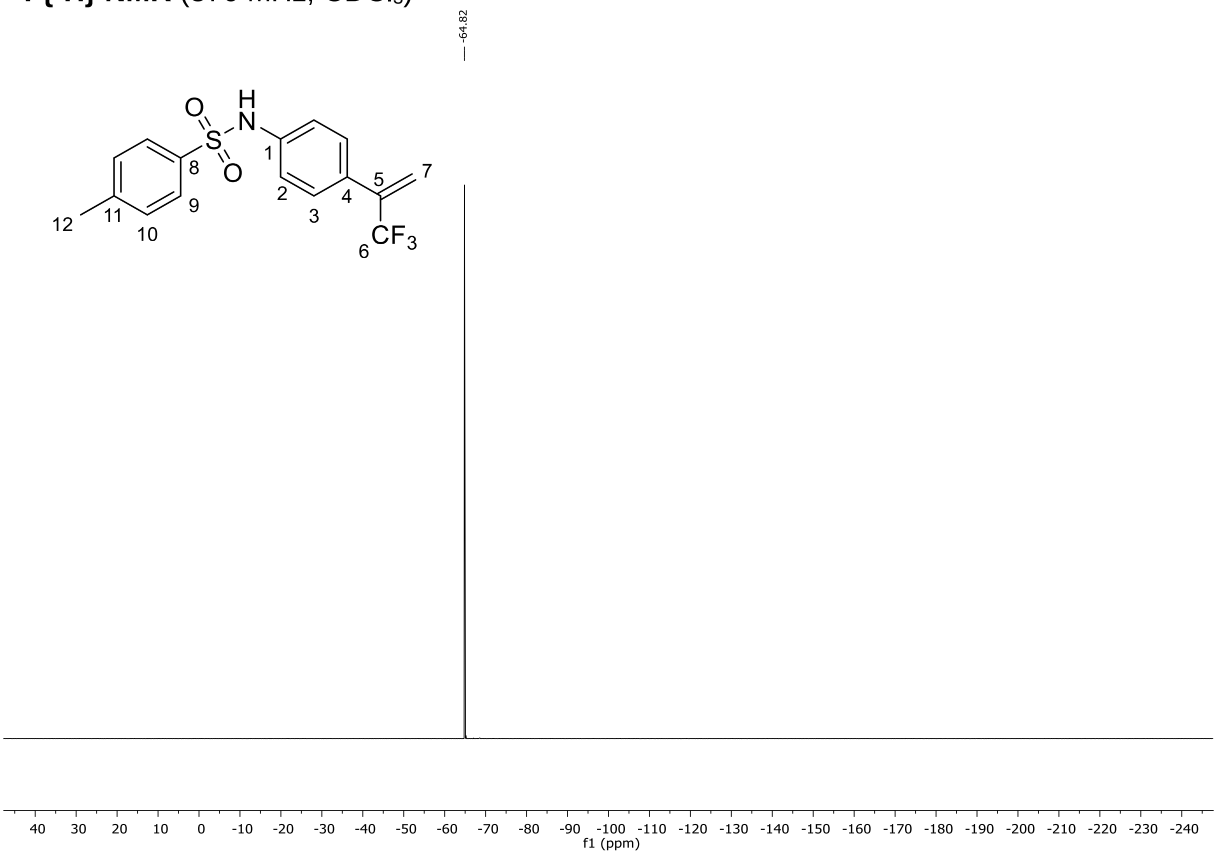

***N*,4-Dimethyl-*N*-(4-(3,3,3-trifluoroprop-1-en-2-yl)phenyl)benzenesulfonamide (1i)**

^1H NMR (599 MHz, $CDCl_3$)

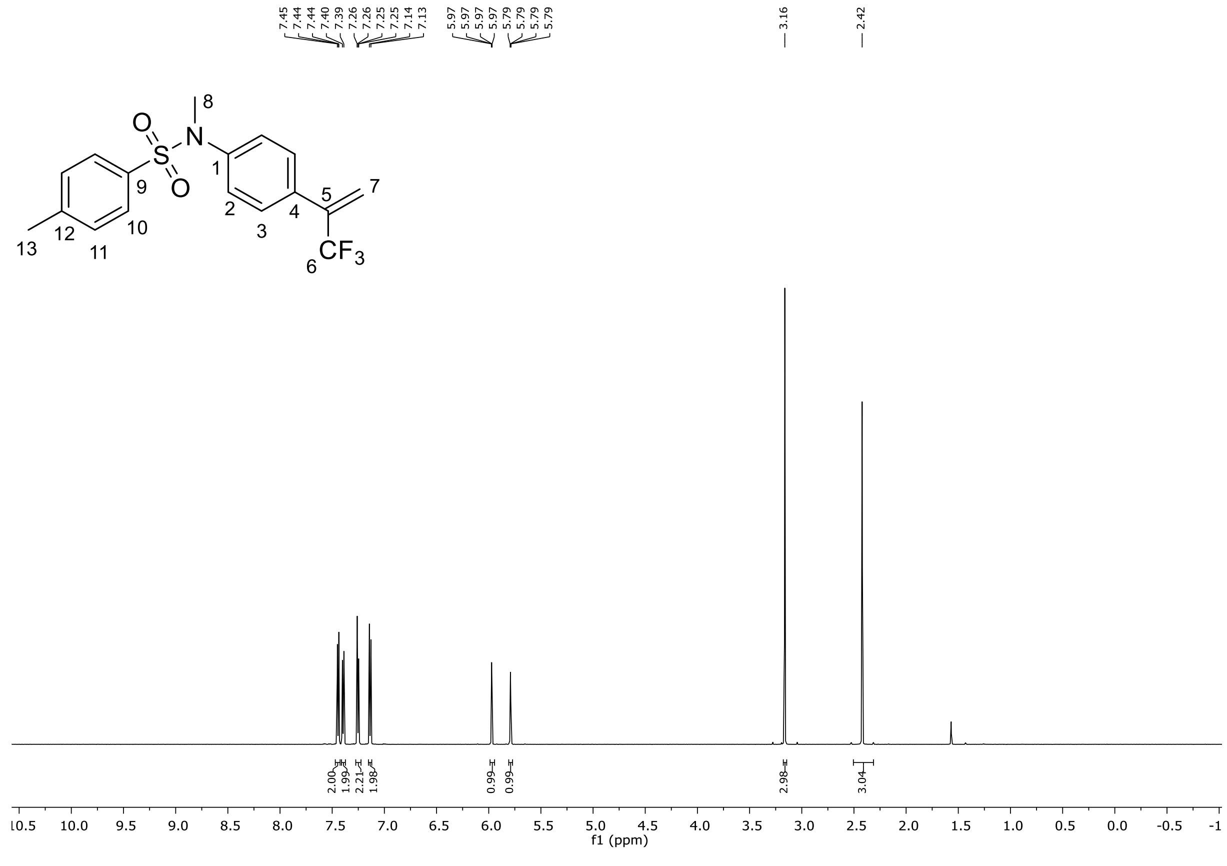

^{13}C NMR (151 MHz, $CDCl_3$)

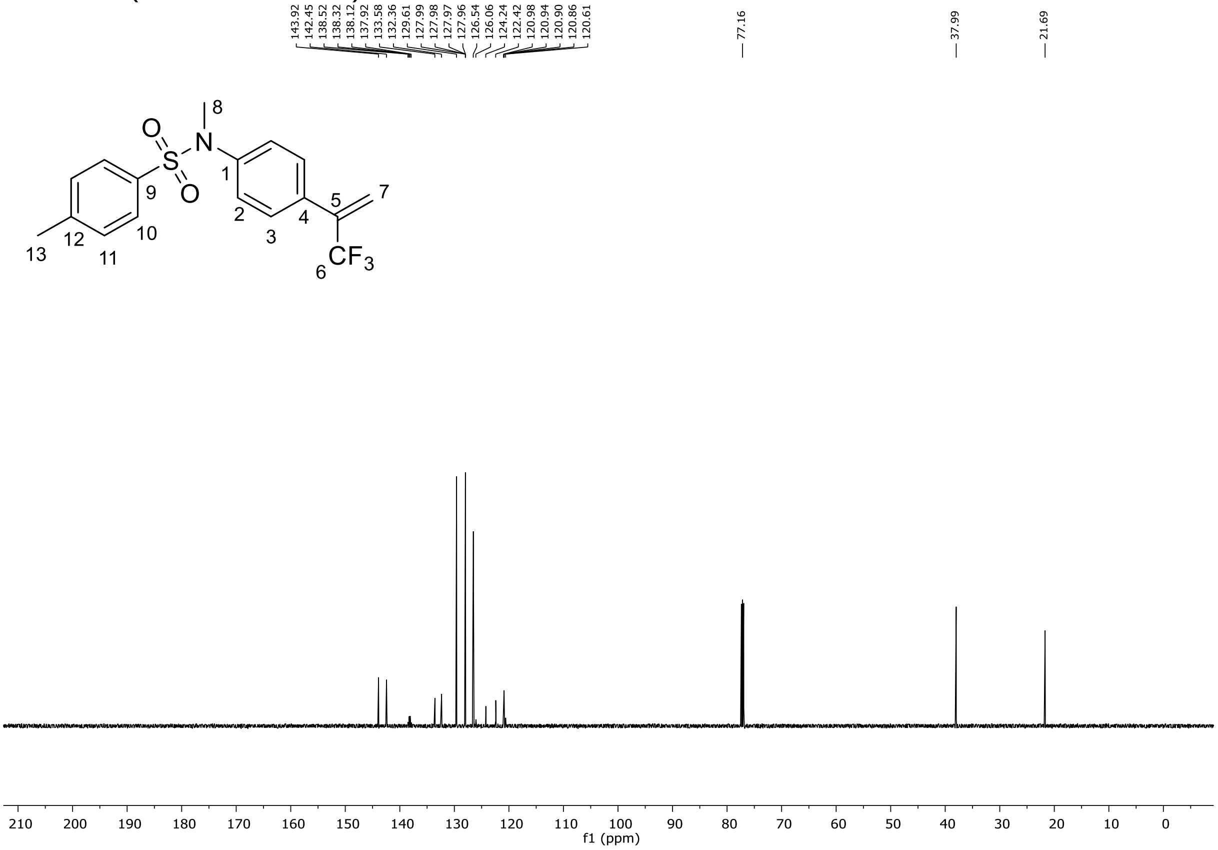

^{19}F{^{1}H} NMR (376 MHz, $CDCl_3$)

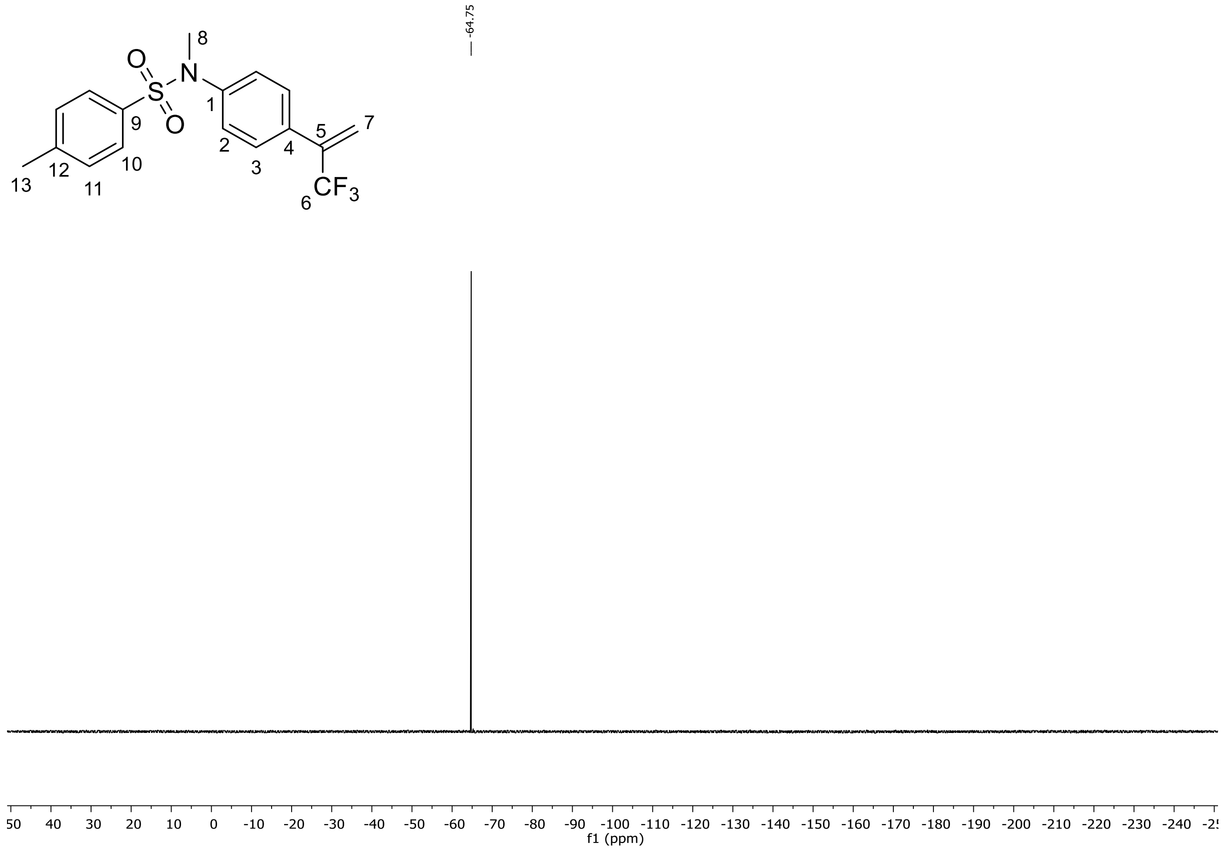

4-Fluoro-4'-(3,3,3-trifluoroprop-1-en-2-yl)-1,1'-biphenyl (1j)

^{1}H NMR (599 MHz, $CDCl_3$)

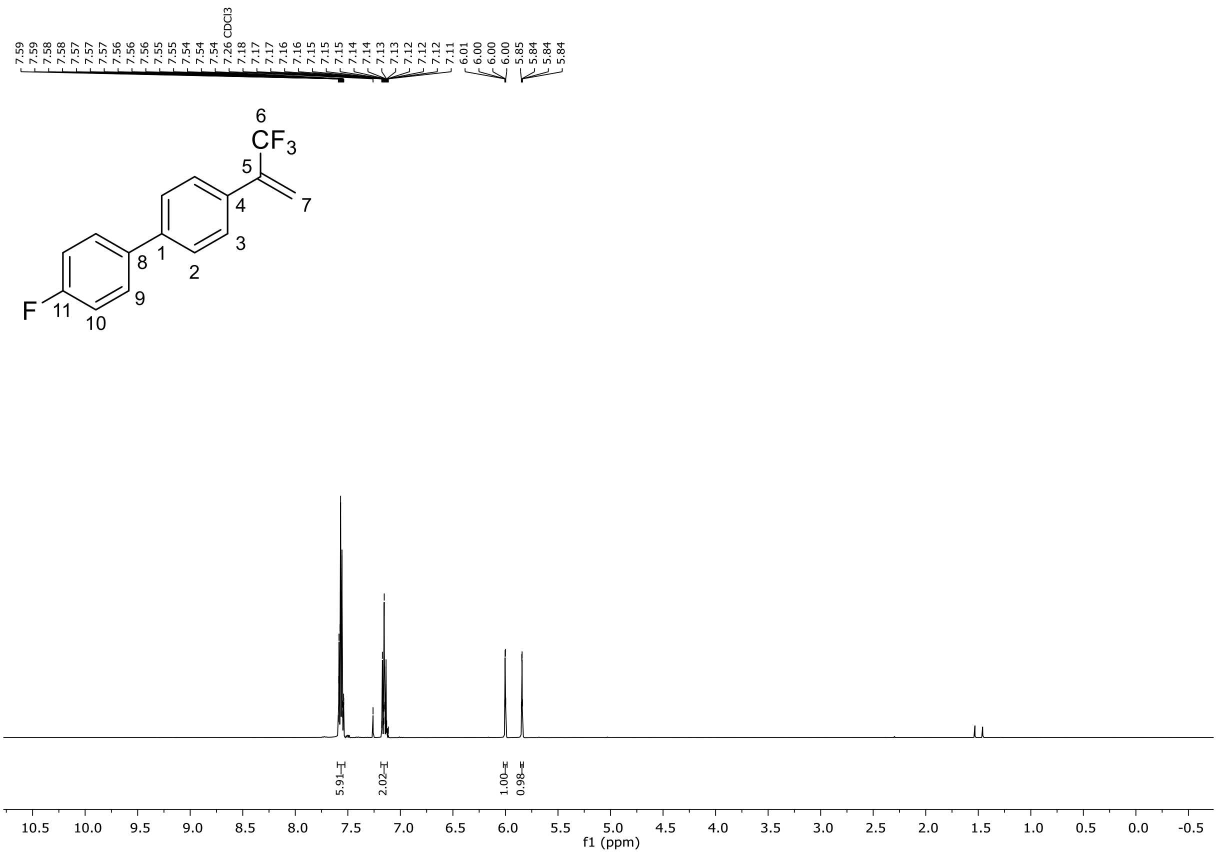

^{13}C NMR (151 MHz, $CDCl_3$)

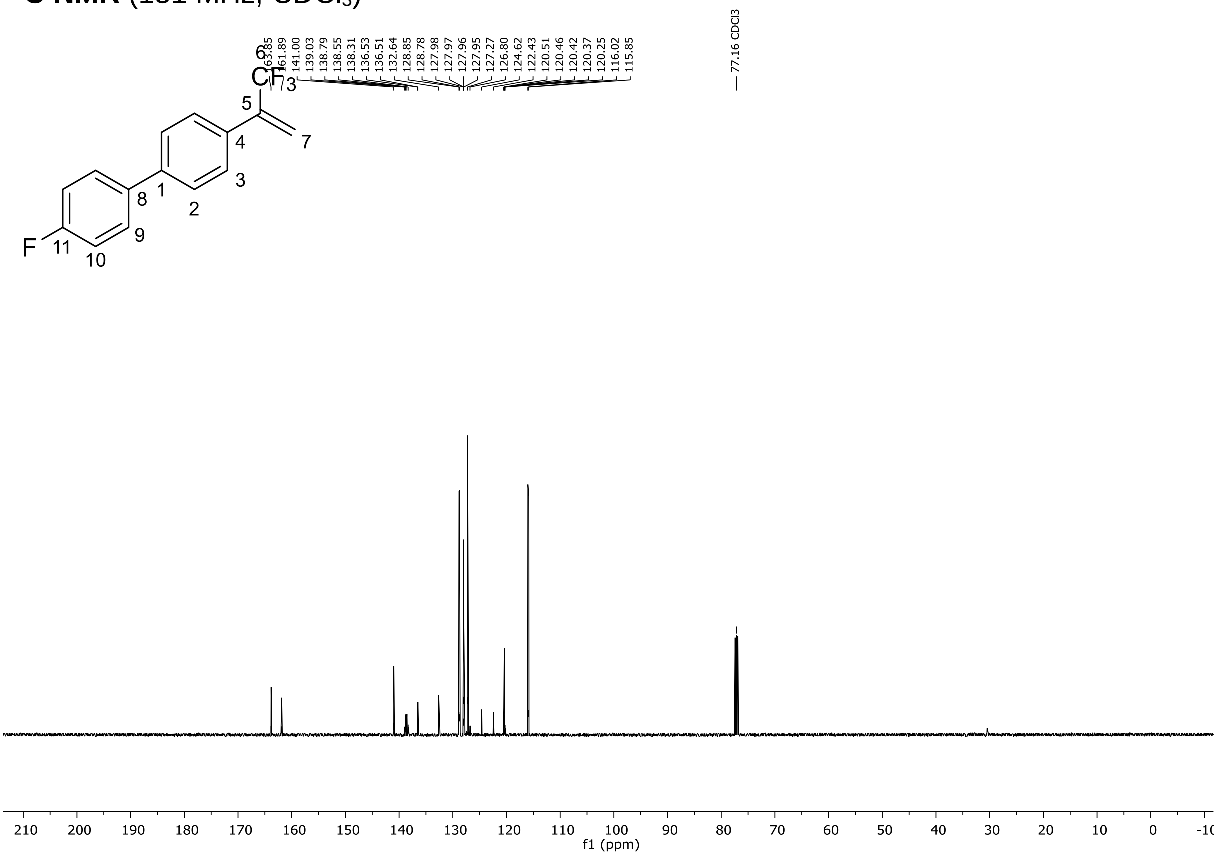

$^{19}F\{^{1}H\}$ NMR (376 MHz, $CDCl_3$)

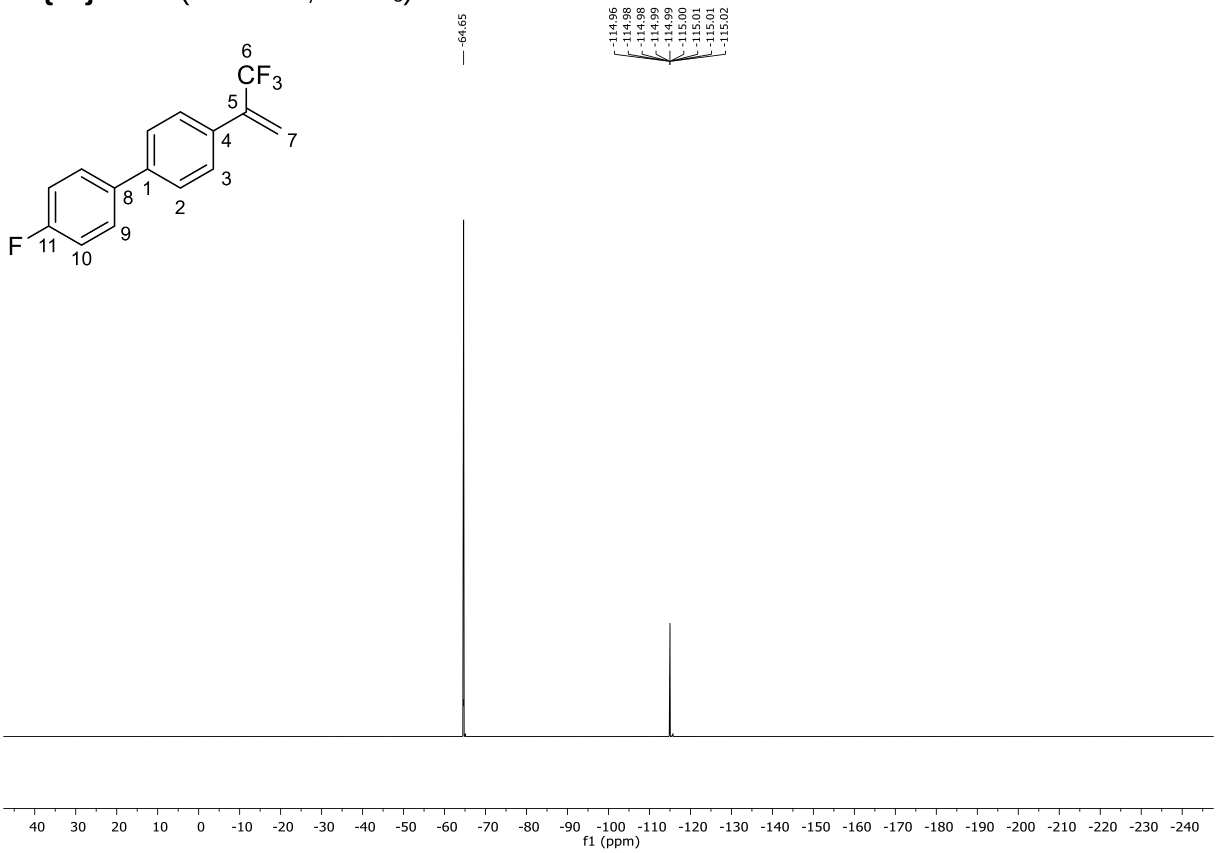

2-Methoxy-5-(3,3,3-trifluoroprop-1-en-2-yl)benzonitrile (**1k**)

^{1}H NMR (599 MHz, $CDCl_3$)

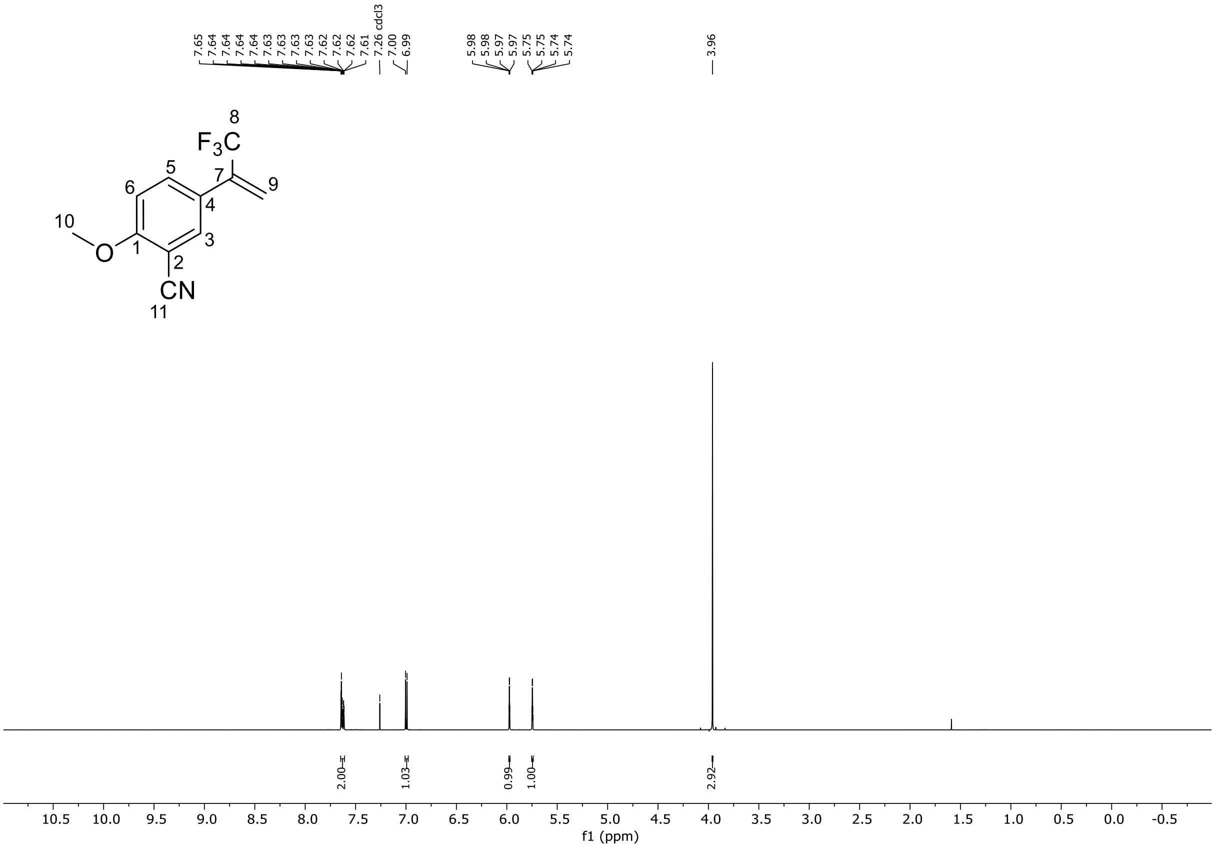

^{13}C NMR (151 MHz, $CDCl_3$)

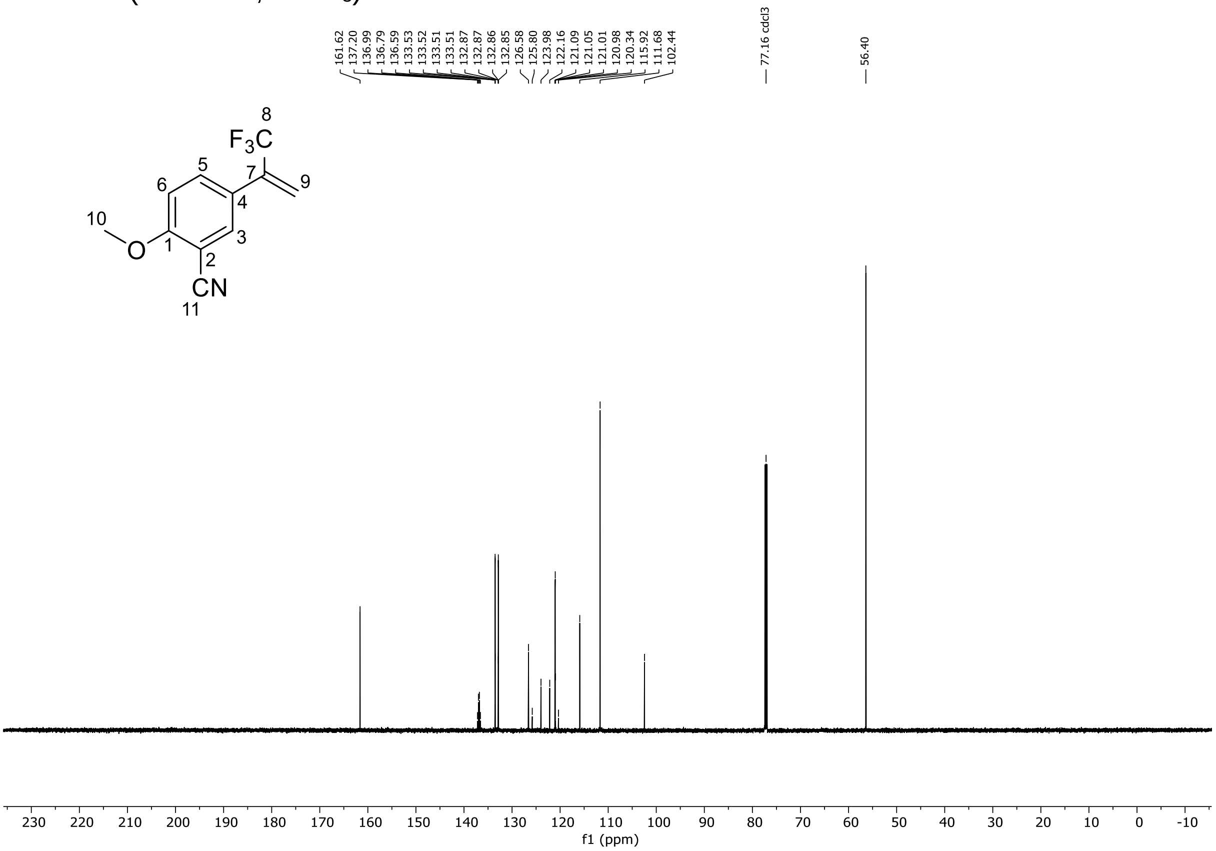

^{19}F{^{1}H} NMR (376 MHz, $CDCl_3$)

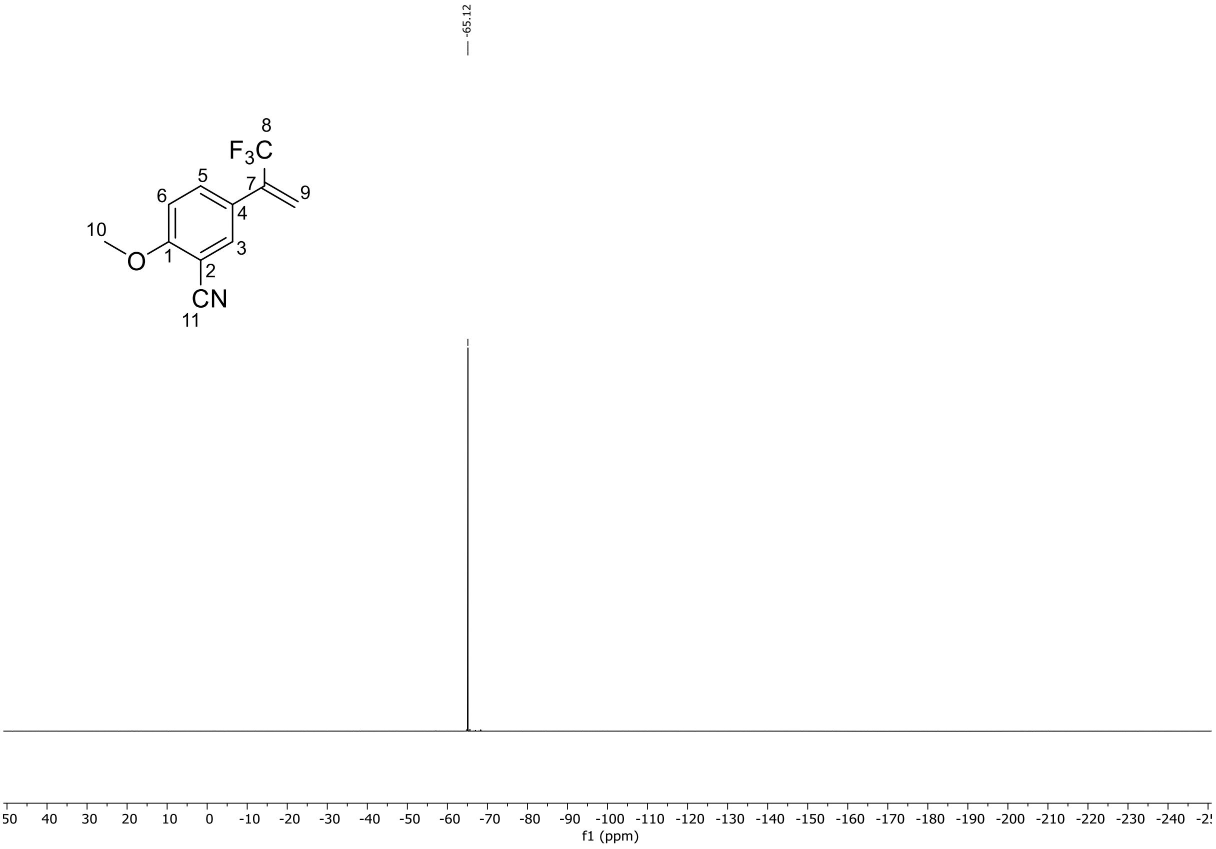

1-(Methoxymethyl)-4-(3,3,3-trifluoroprop-1-en-2-yl)benzene (1m)

^{1}H NMR (599 MHz, $CDCl_3$)

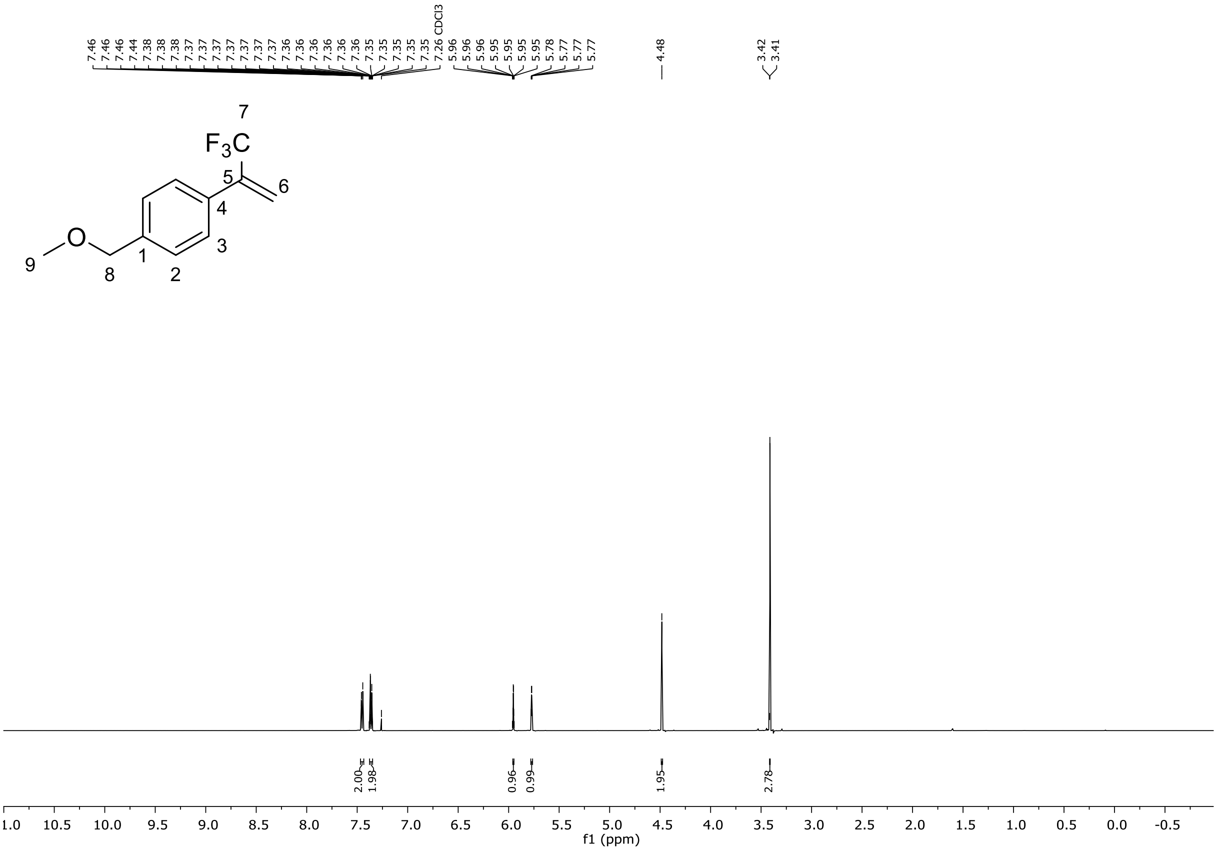

^{13}C NMR (151 MHz, $CDCl_3$)

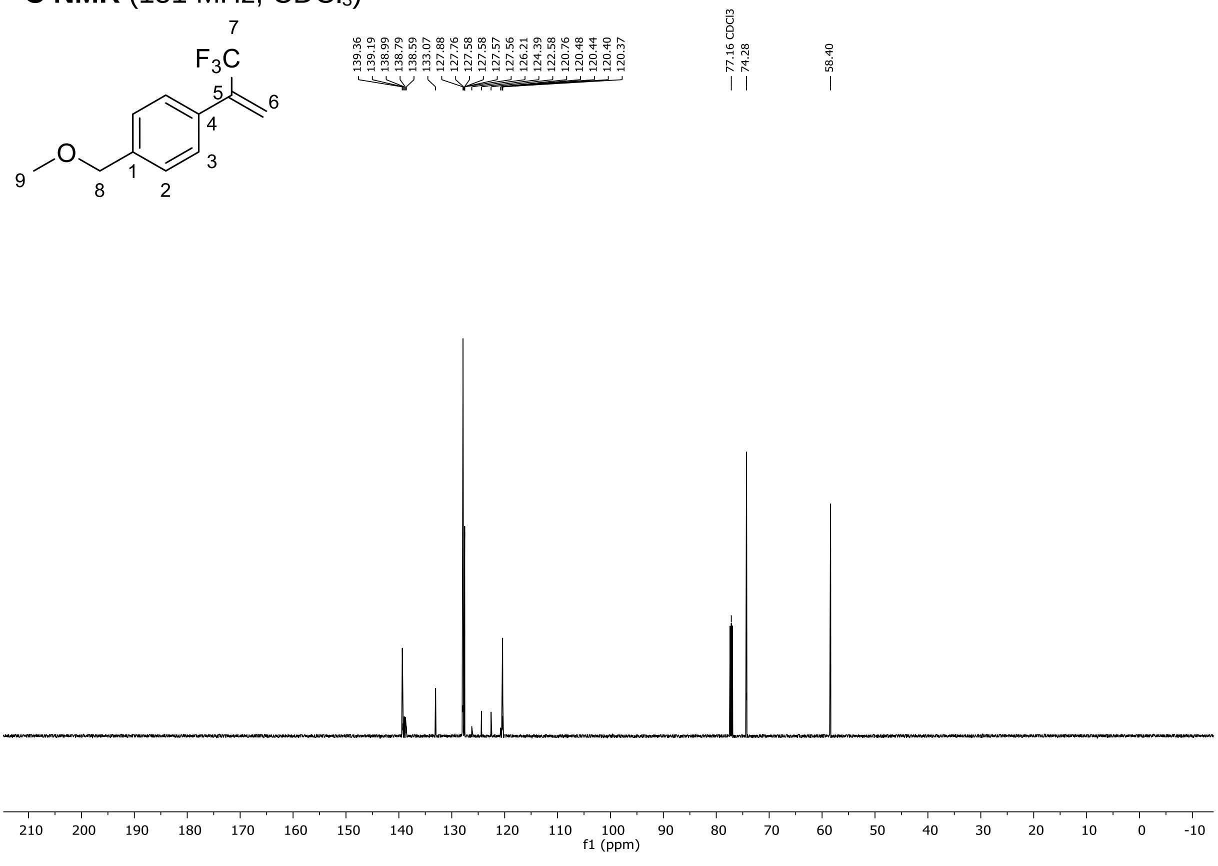

$^{19}F\{^{1}H\}$ NMR (376 MHz, $CDCl_3$)

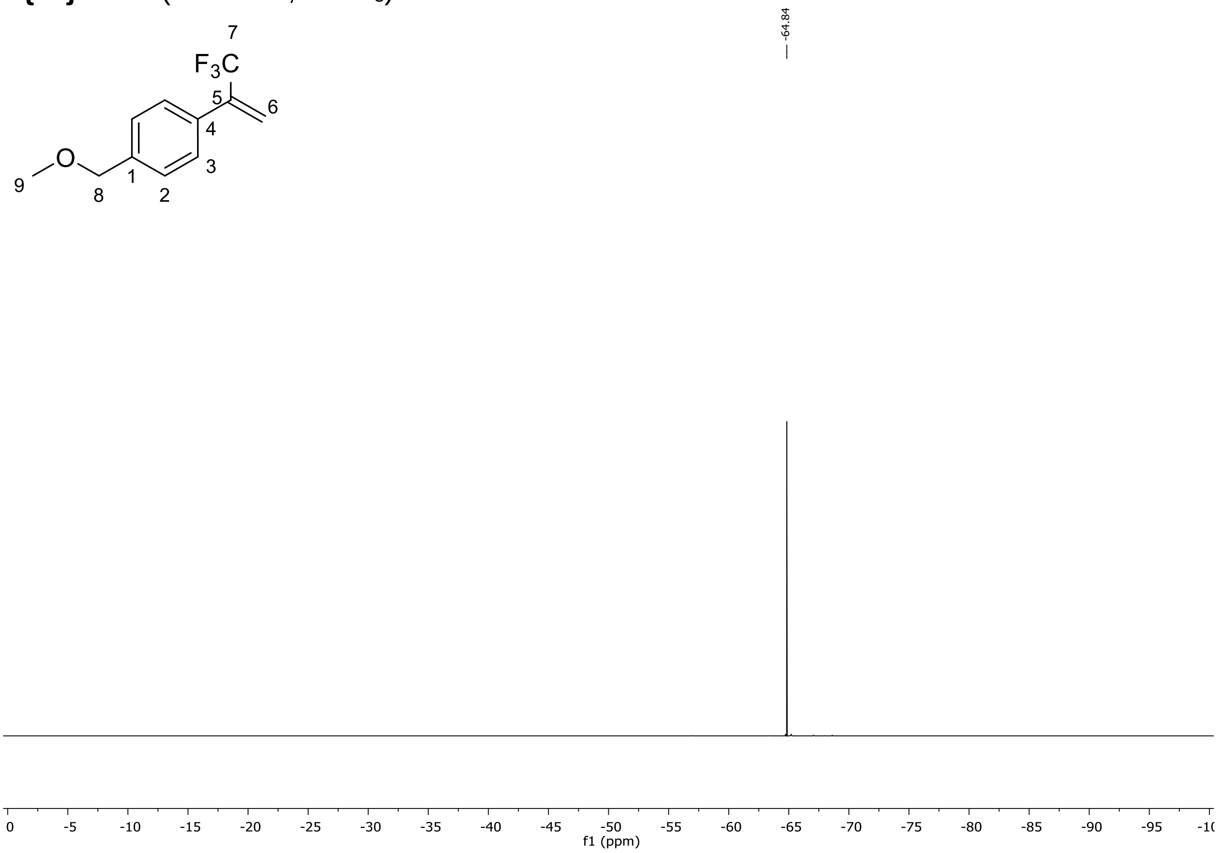

4-nitro-4'-(3,3,3-trifluoroprop-1-en-2-yl)-1,1'-biphenyl (1o)

^{1}H NMR (500 MHz, $CDCl_3$)

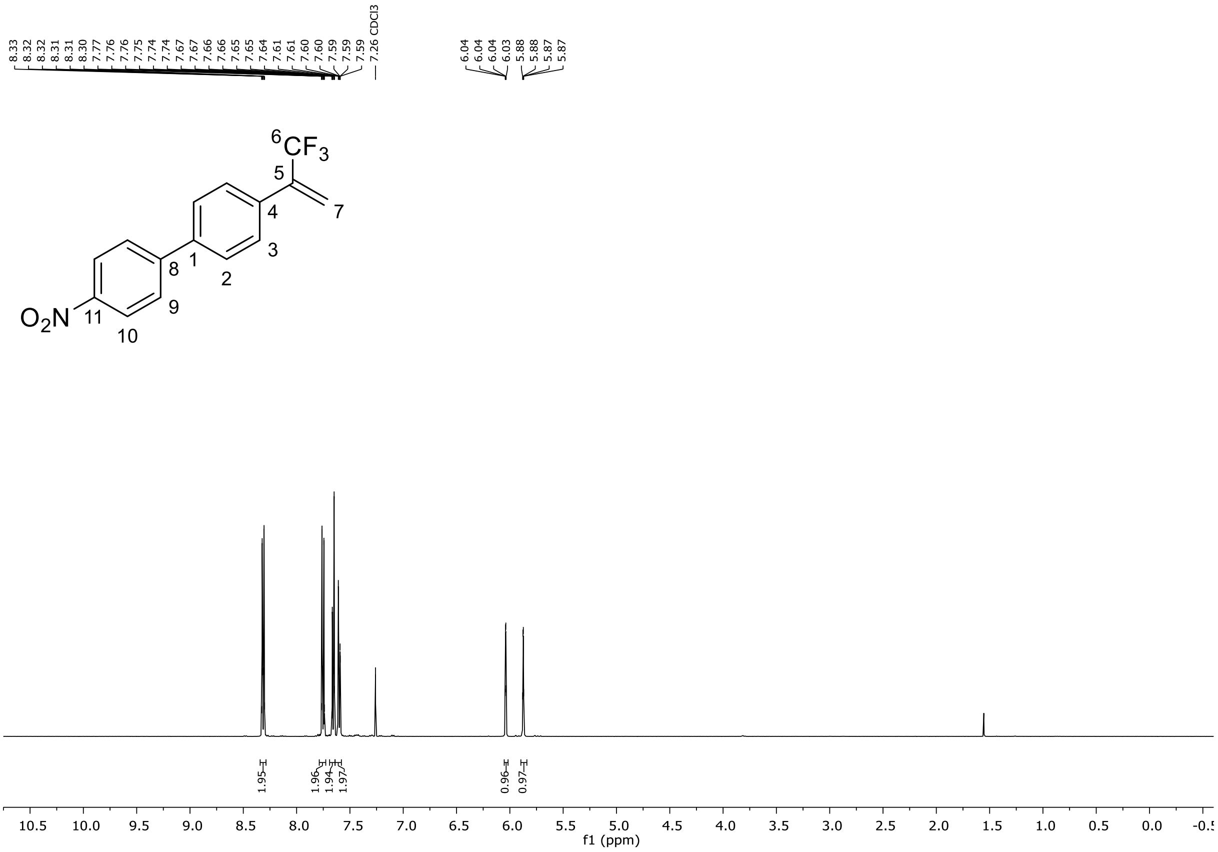

^{13}C NMR (126 MHz, $CDCl_3$)

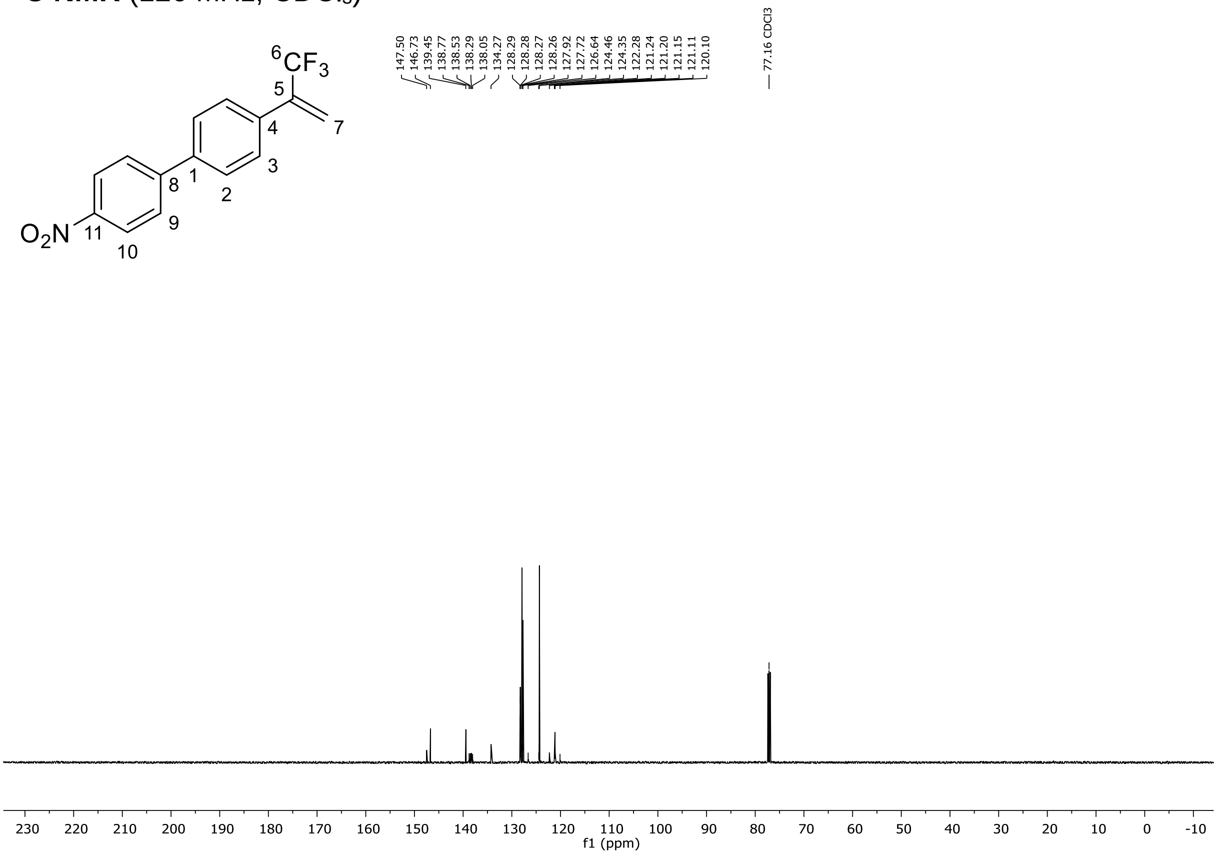

^{19}F{^{1}H} NMR (376 MHz, $CDCl_3$)

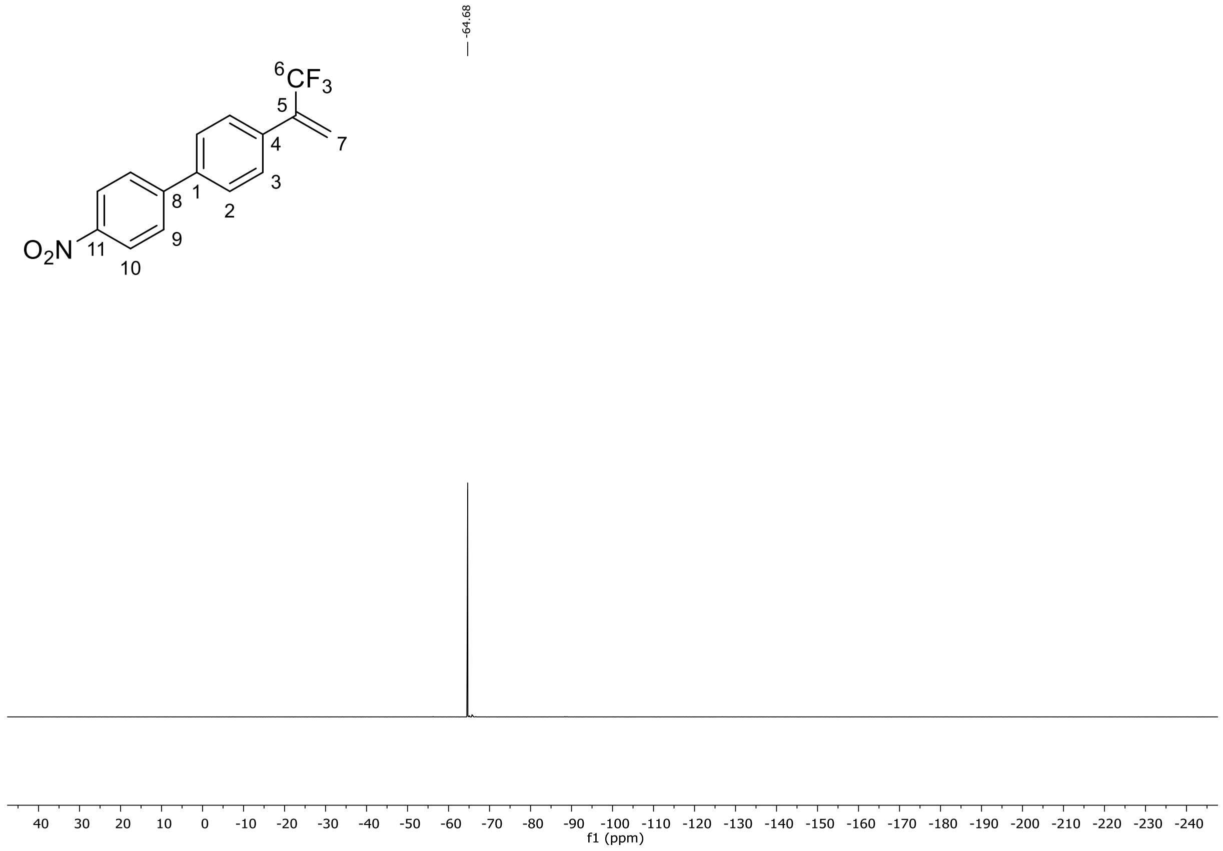

4-(3,3,3-Trifluoroprop-1-en-2-yl)aniline (S23)

^{1}H NMR (500 MHz, $CDCl_3$)

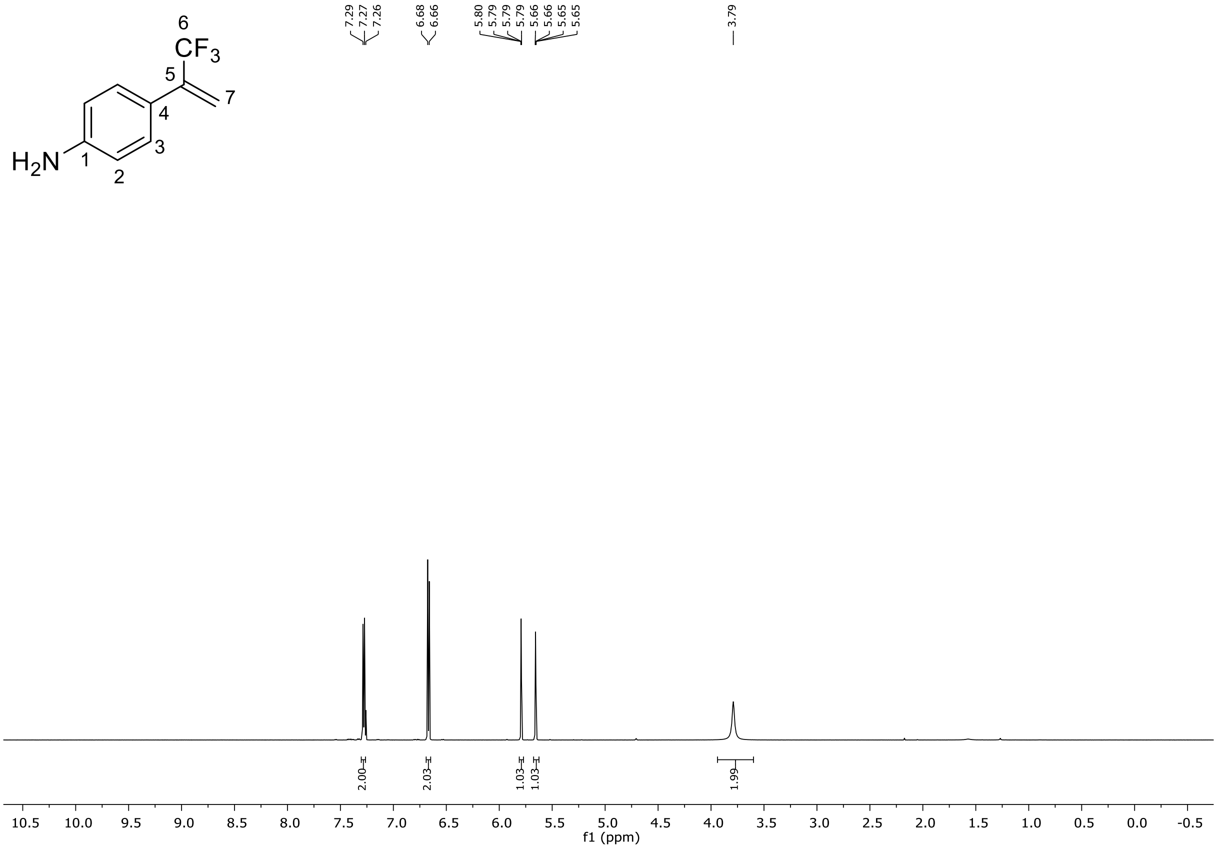

^{13}C NMR (126 MHz, $CDCl_3$)

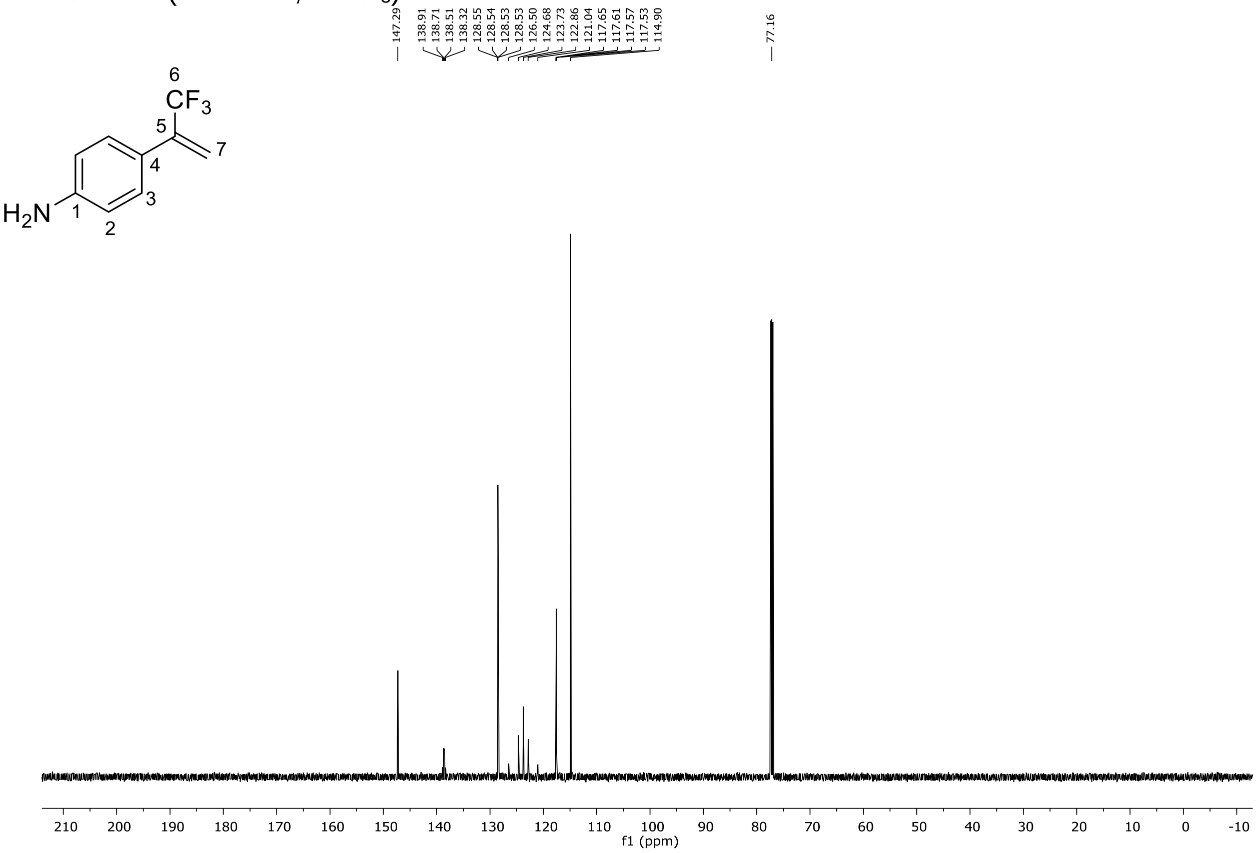

$^{19}F\{^{1}H\}$ NMR (376 MHz, $CDCl_3$)

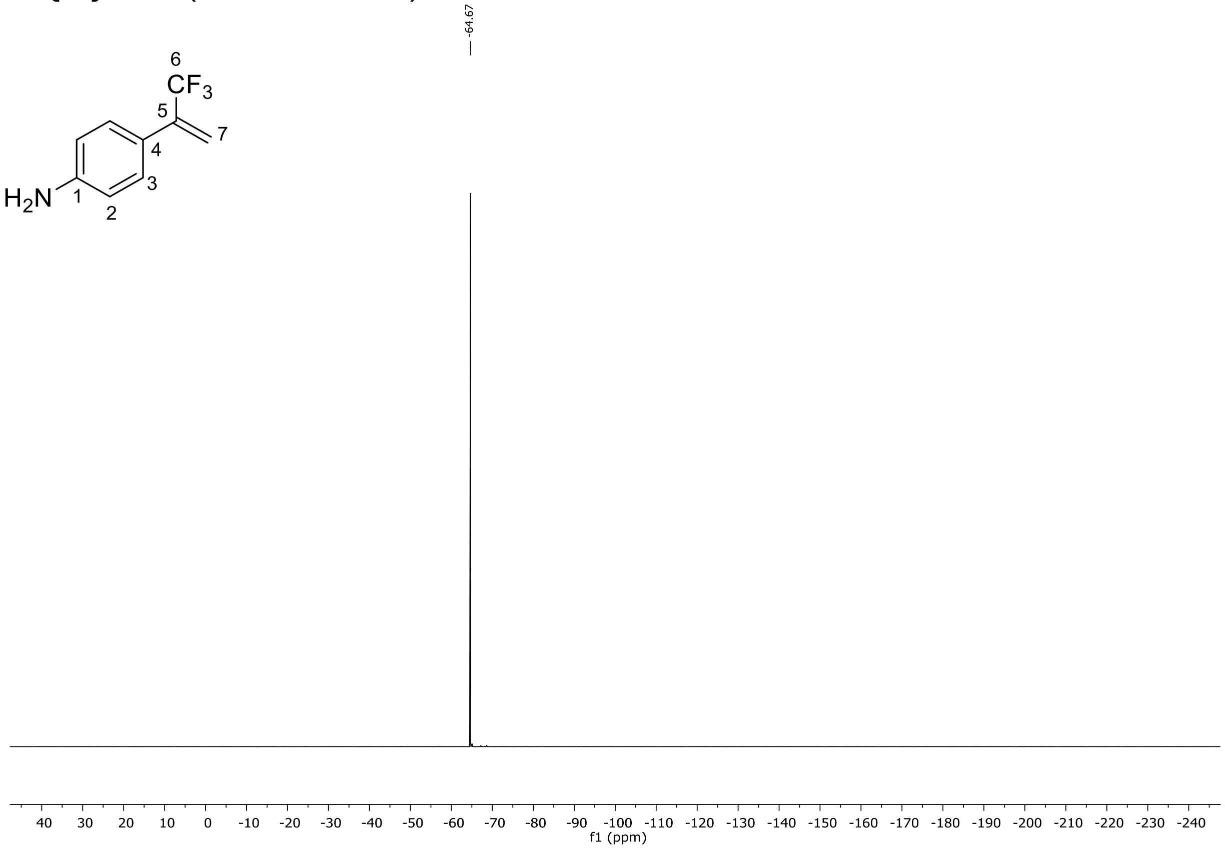

2-(4-(3,3,3-Trifluoroprop-1-en-2-yl)phenyl)isoindoline-1,3-dione (1p)

^{1}H NMR (500 MHz, $CDCl_3$)

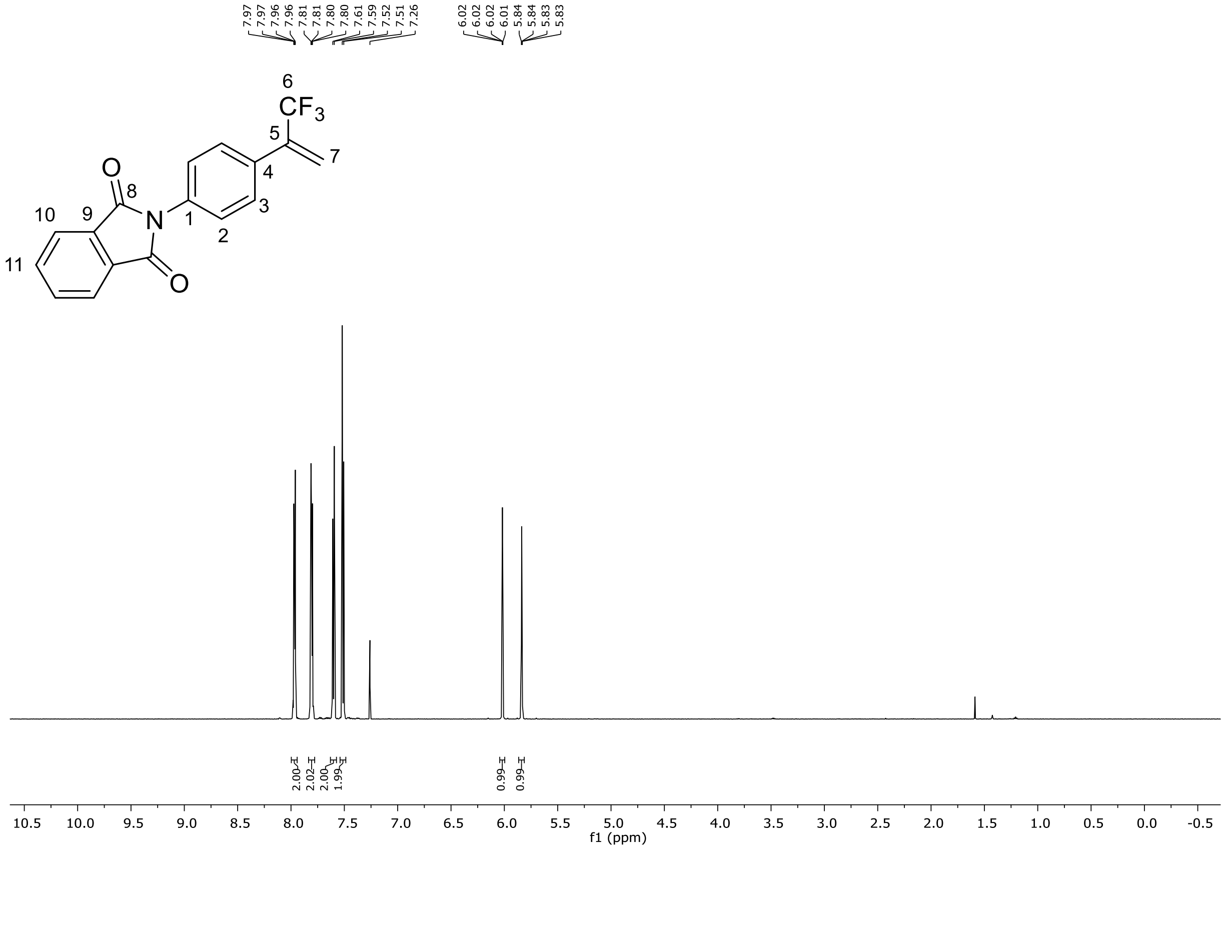

^{13}C NMR (126 MHz, $CDCl_3$)

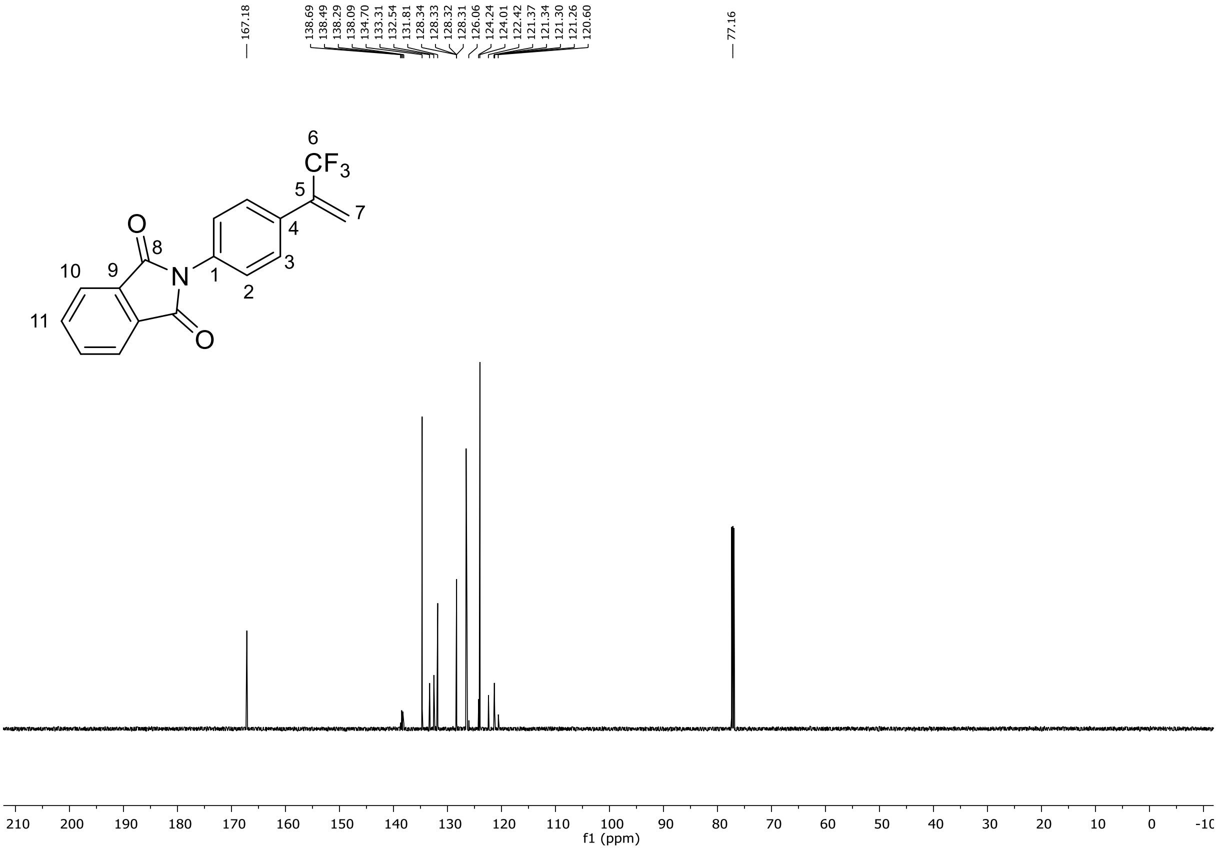

^{19}F{^{1}H} NMR (376 MHz, $CDCl_3$)

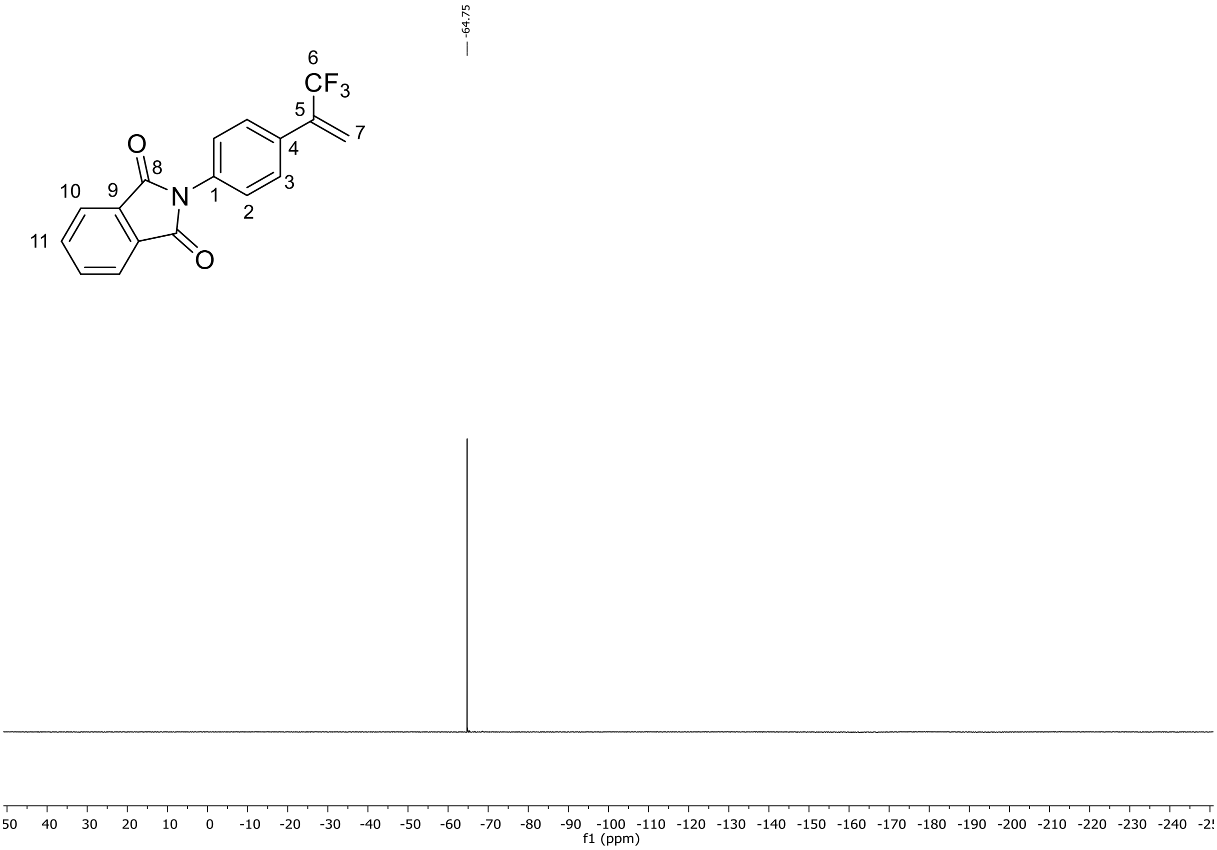

2-Methoxy-5-(3,3,3-trifluoroprop-1-en-2-yl)phenol (S24)

^{1}H NMR (500 MHz, $CDCl_3$)

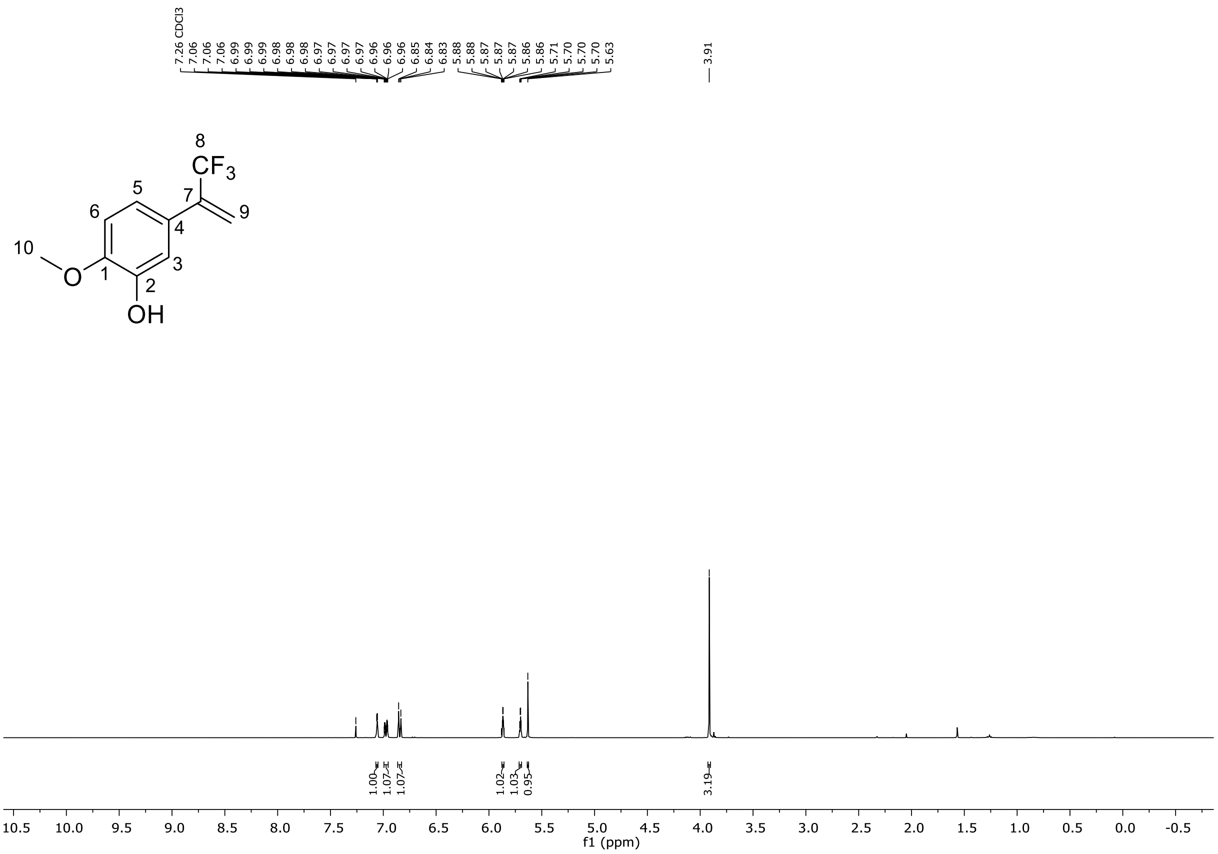

^{13}C NMR (126 MHz, $CDCl_3$)

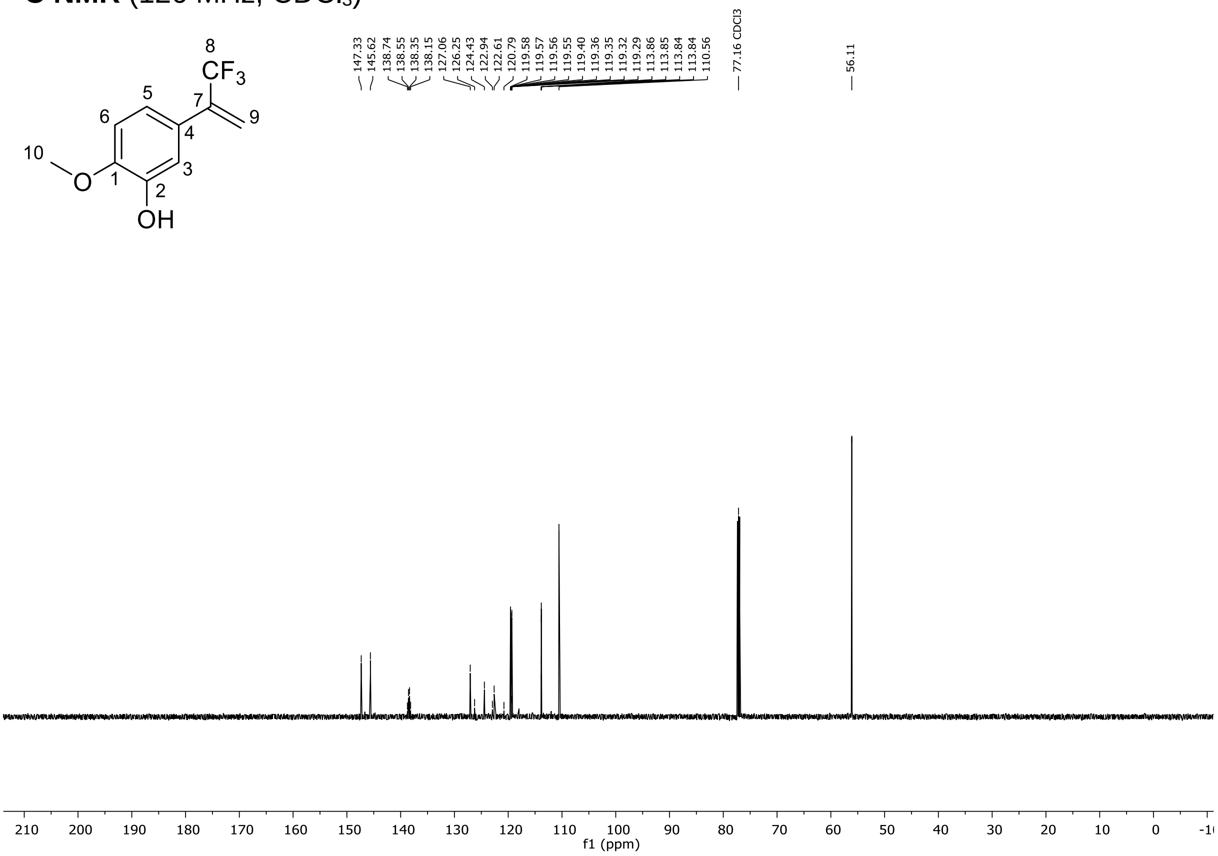

$^{19}F\{^{1}H\}$ NMR (376 MHz, $CDCl_3$)

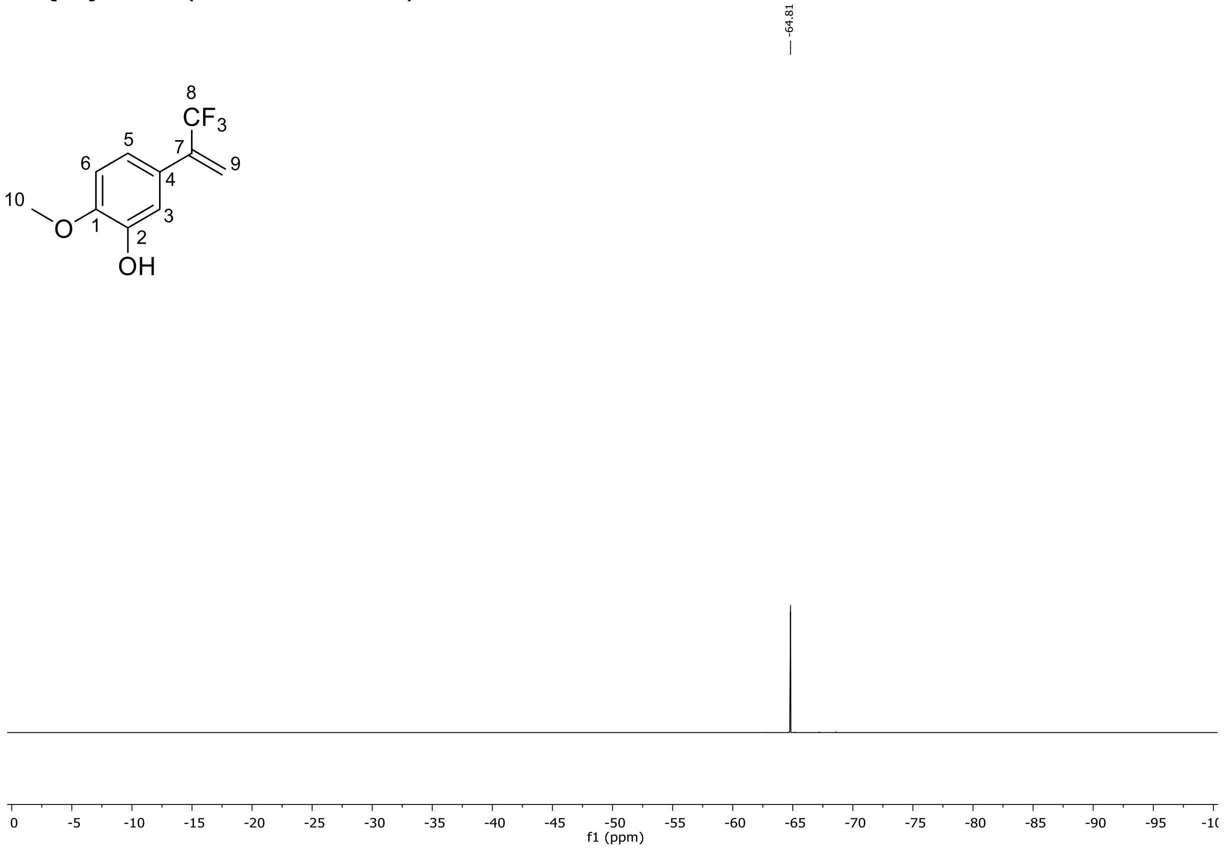

2-Methoxy-5-(3,3,3-trifluoroprop-1-en-2-yl)phenyl trifluoromethanesulfonate (1q)

^{1}H NMR (500 MHz, $CDCl_3$)

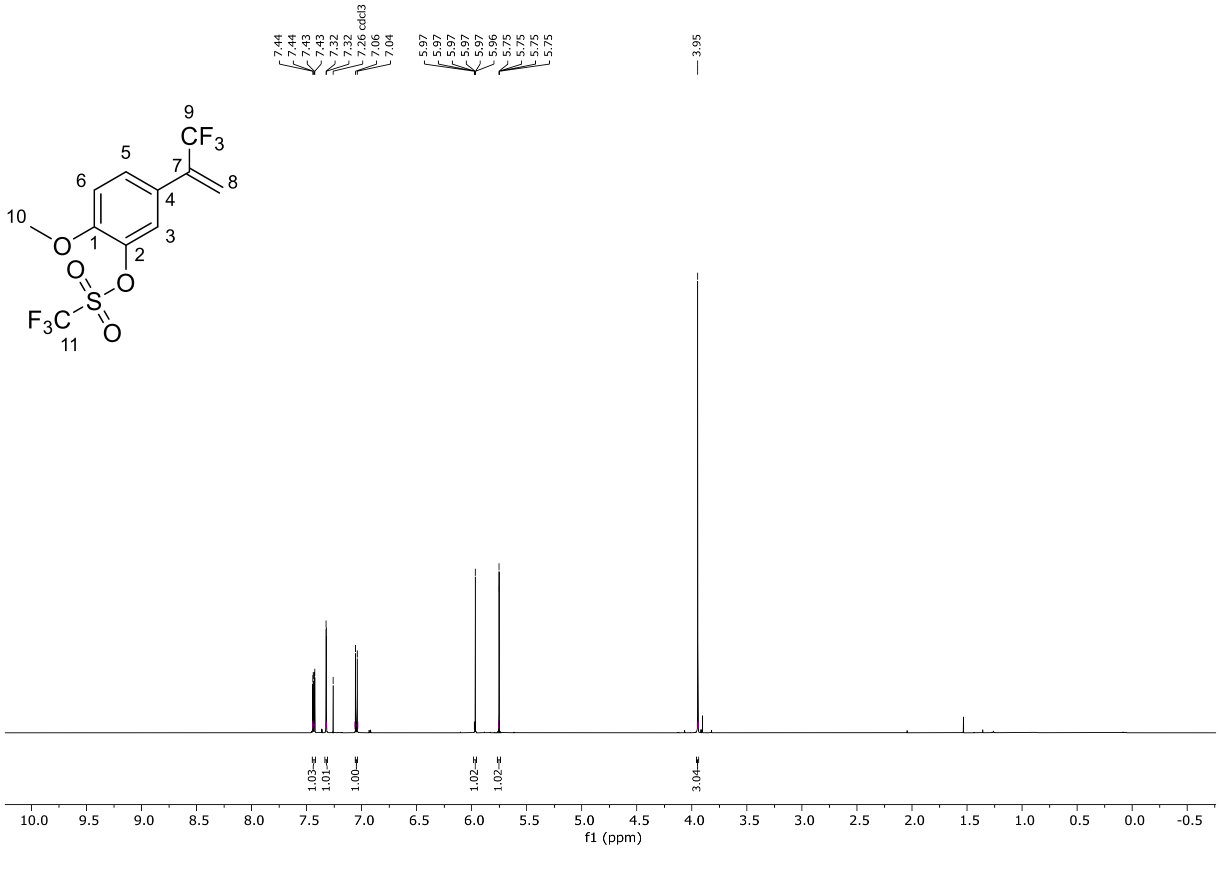

^{13}C NMR (126 MHz, $CDCl_3$)

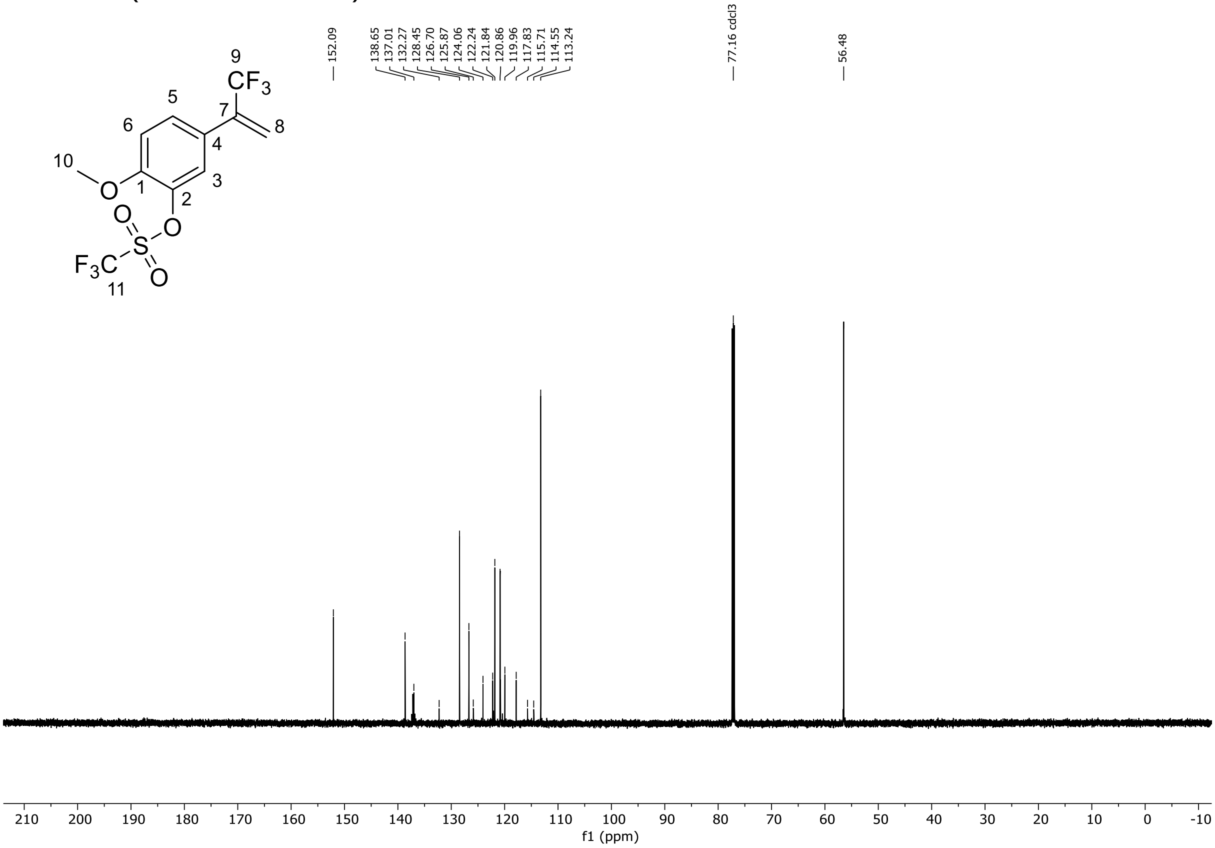

^{19}F{^{1}H} NMR (376 MHz, $CDCl_3$)

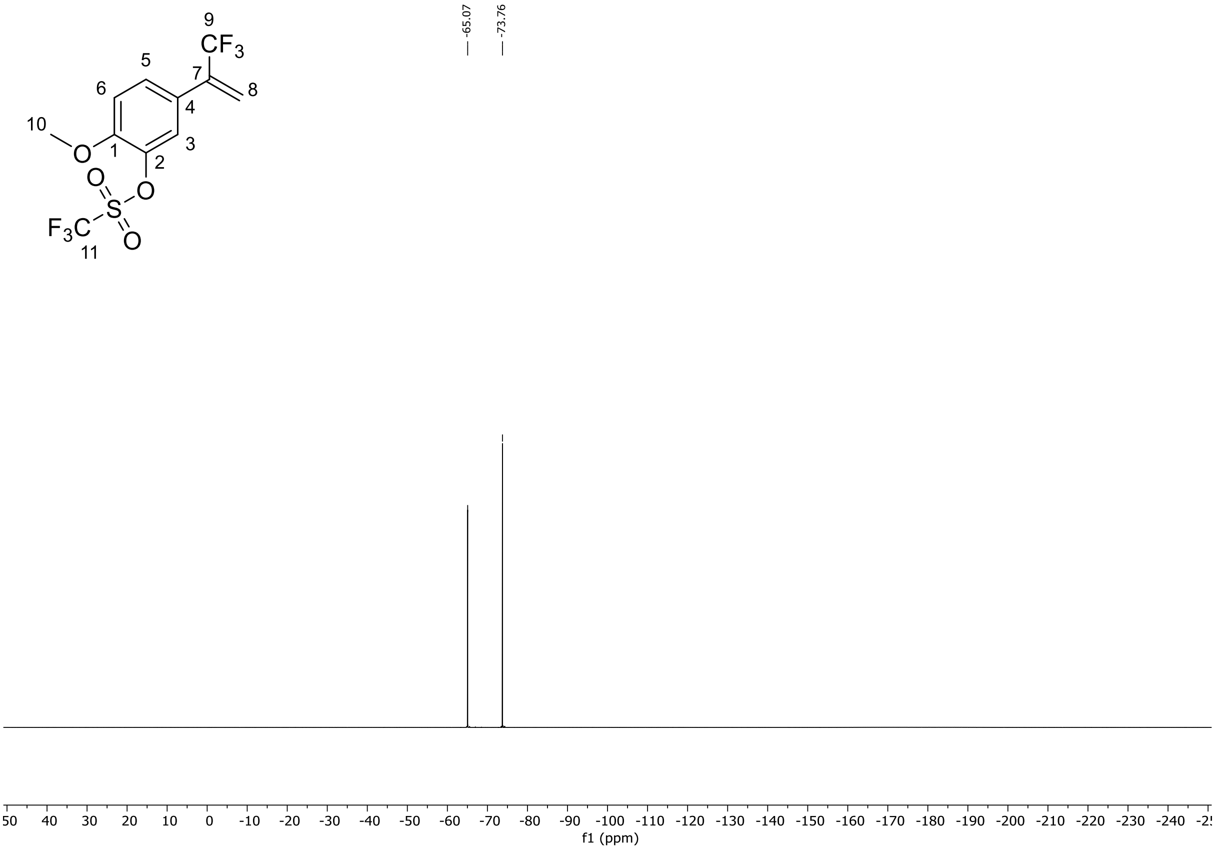

NMR of Catalysts

Dimethyl 2,2'-((2-iodo-5-methyl-1,3-phenylene)bis(oxy))diacetate (8)

^{1}H NMR (500 MHz, $CDCl_3$)

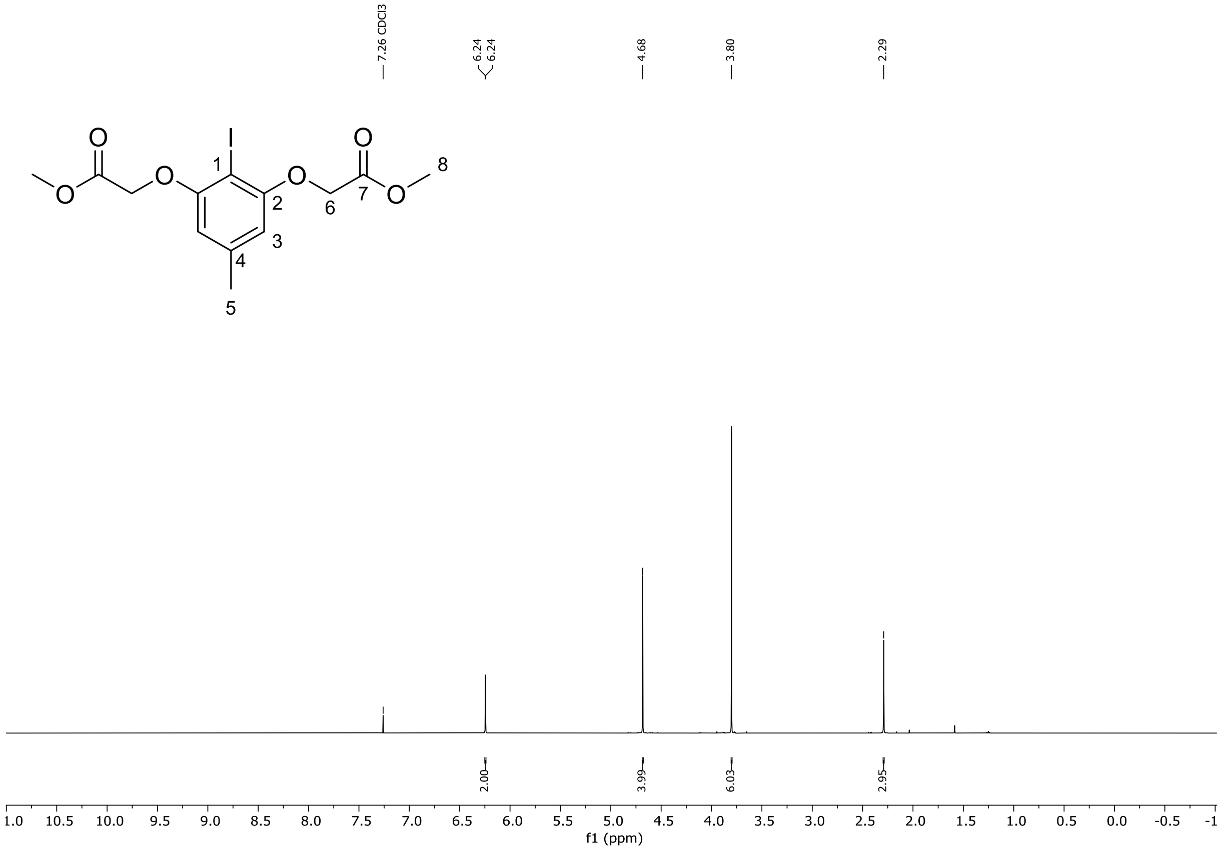

^{13}C NMR (126 MHz, $CDCl_3$)

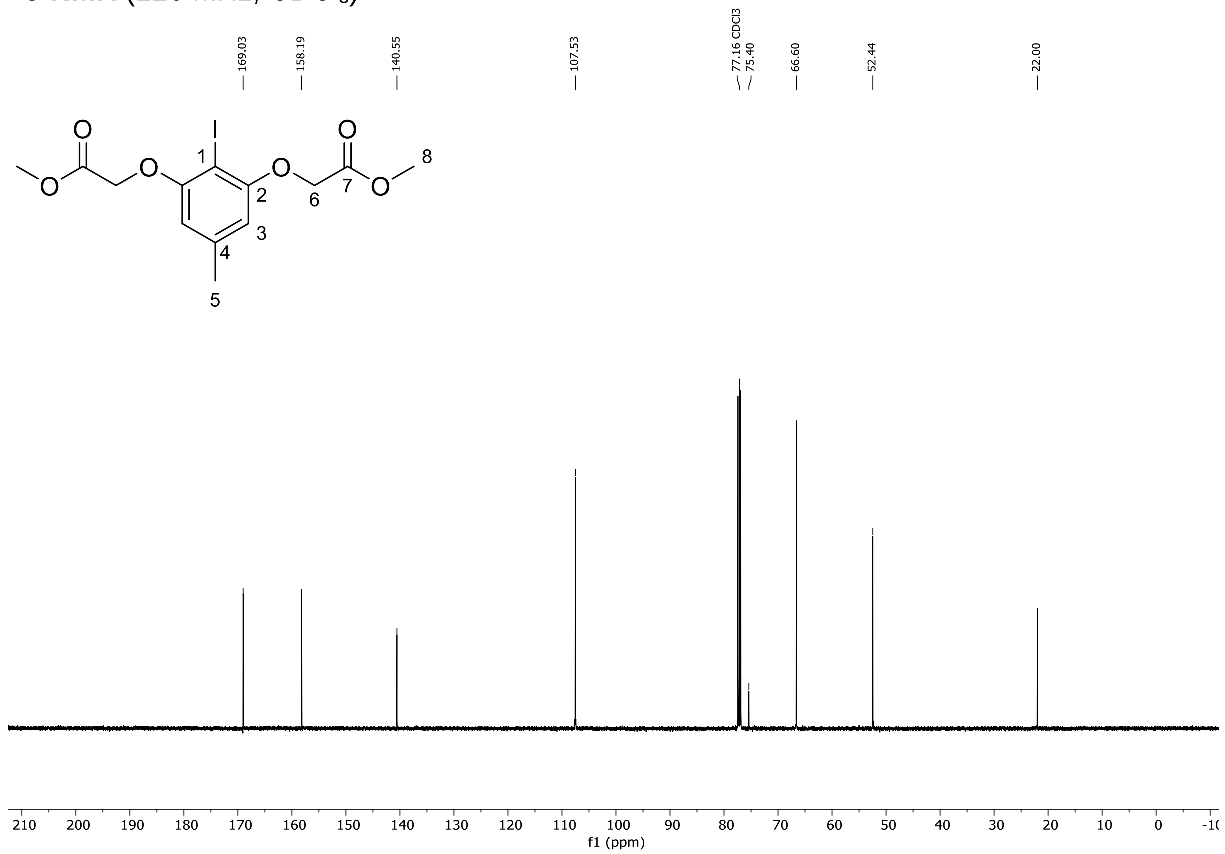

Dimethyl 2,2'-((2-iodo-5-(methoxycarbonyl)-1,3-phenylene)bis(oxy))(*2R,2'R*)-dipropionate (10)

^{1}H NMR (300 MHz, $CDCl_3$)

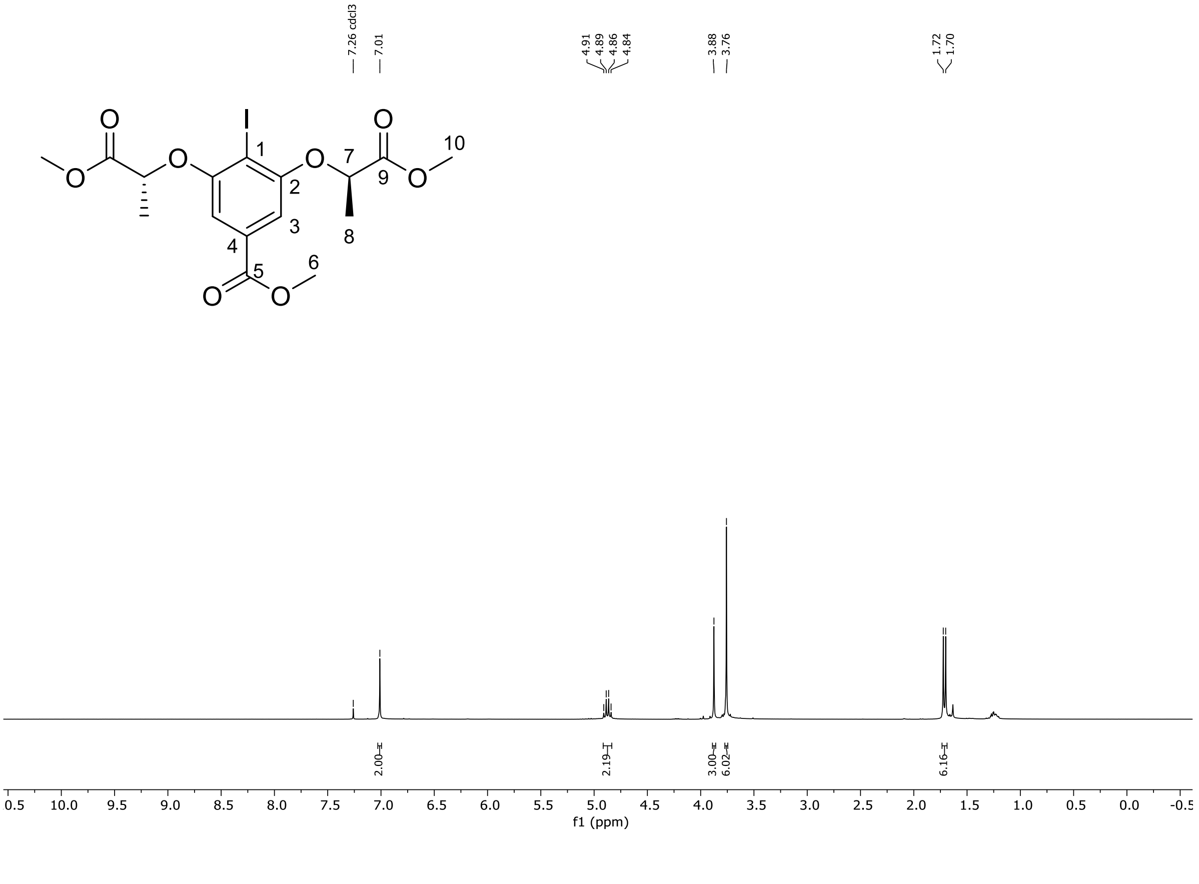

^{13}C NMR (75 MHz, $CDCl_3$)

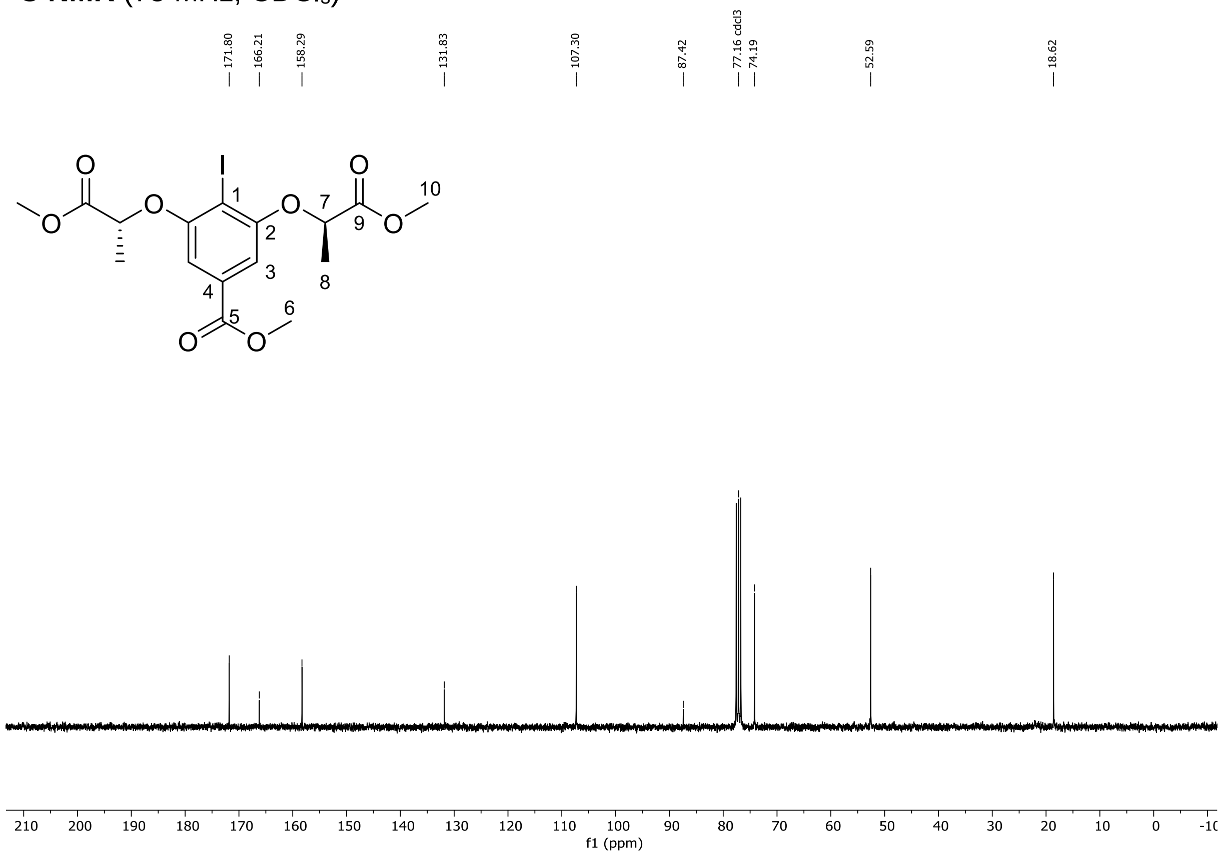

Dimethyl 2,2'-((2-iodo-5-(methoxycarbonyl)-1,3-phenylene)bis(oxy))(*2R,2'R*)-dipropionate (11)

^{1}H NMR (500 MHz, $CDCl_3$)

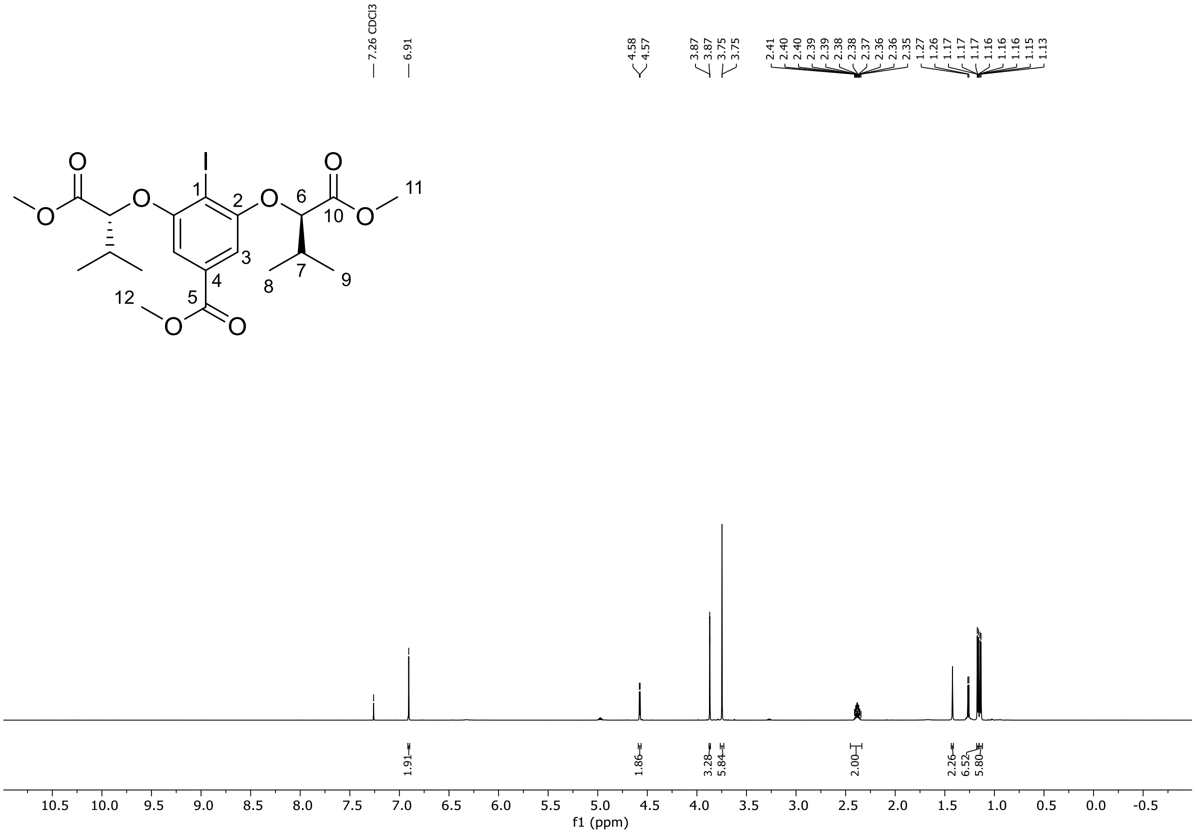

^{13}C NMR (126 MHz, $CDCl_3$)

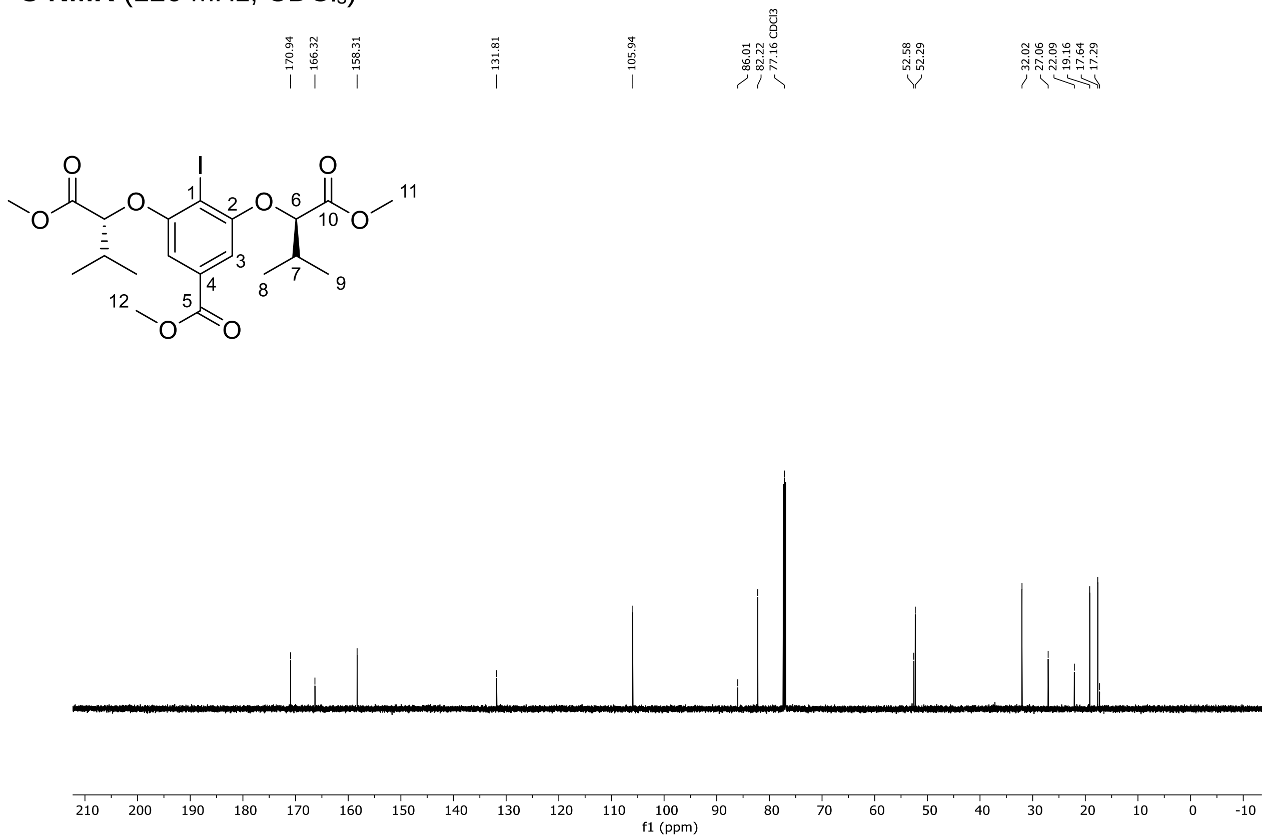

Dimethyl 2,2'-((2-iodo-5-methyl-1,3-phenylene)bis(oxy))(*2R,2'R*)-bis(3-phenylpropanoate) (13)

^{1}H NMR (500 MHz, $CDCl_3$)

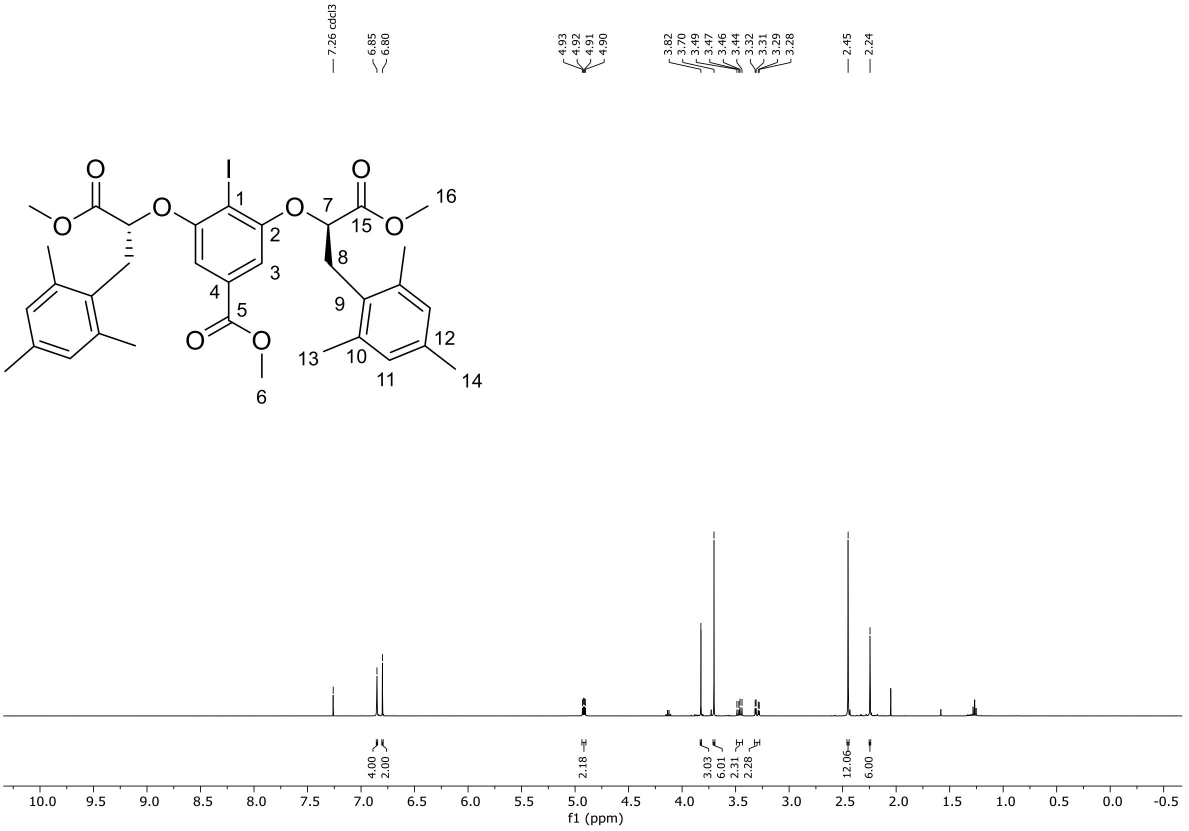

^{13}C NMR (126 MHz, $CDCl_3$)

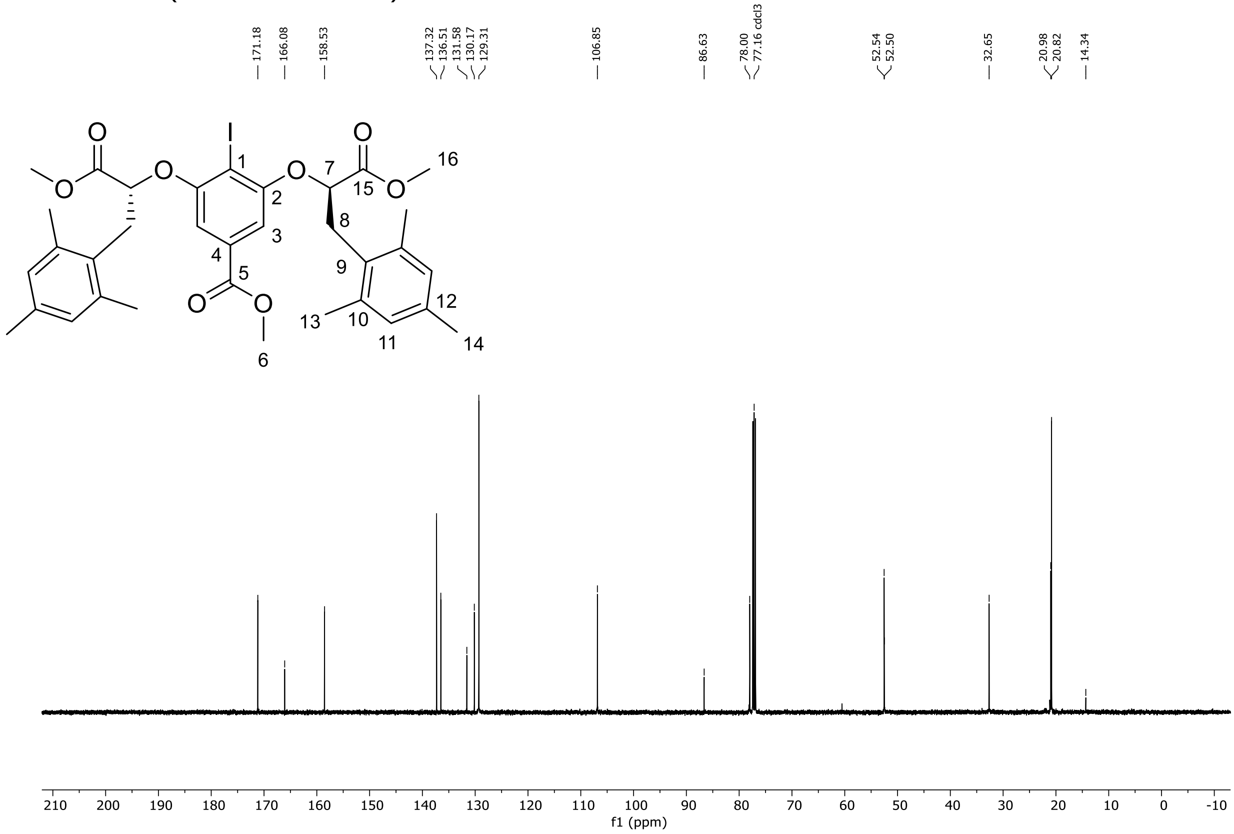

Dibenzyl 2,2'-((2-Iodo-5-(methoxycarbonyl)-1,3-phenylene)bis(oxy))(*2R,2'R*)-dipropionate (14)

^{1}H NMR (599 MHz, $CDCl_3$)

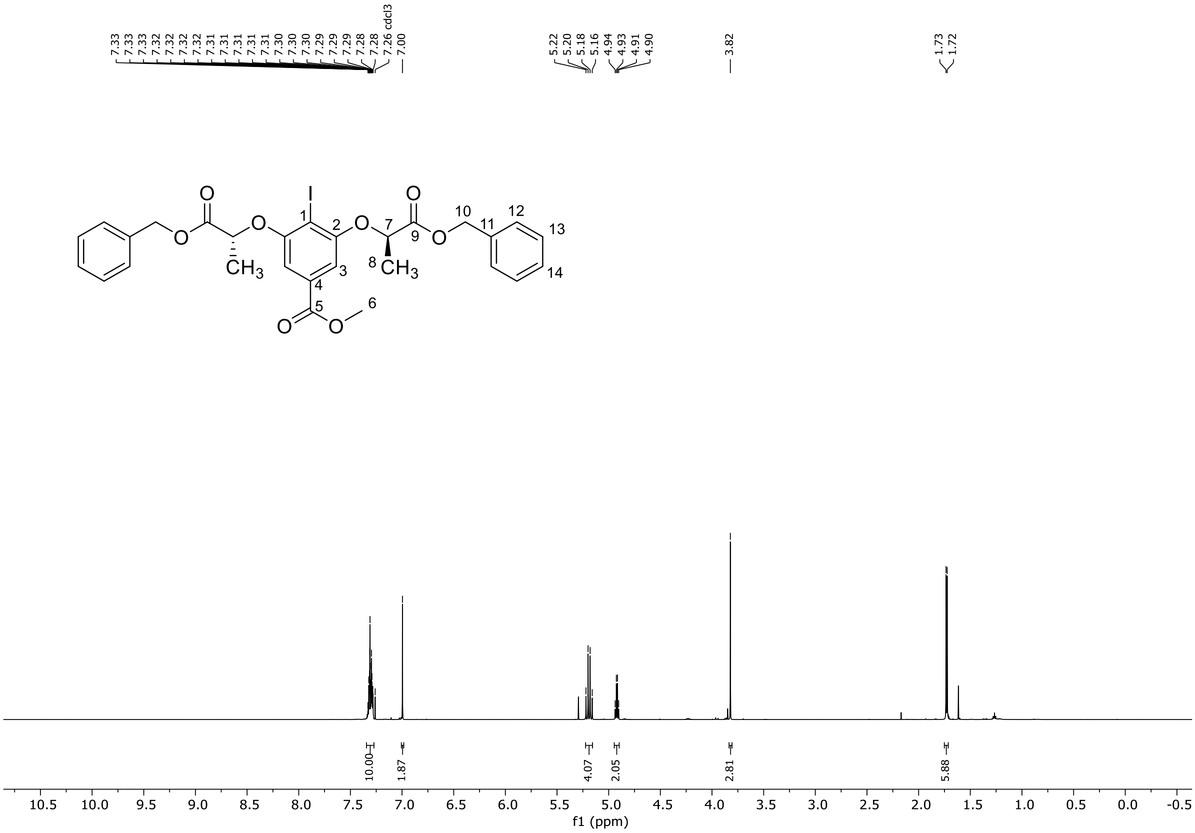

^{13}C NMR (151 MHz, $CDCl_3$)

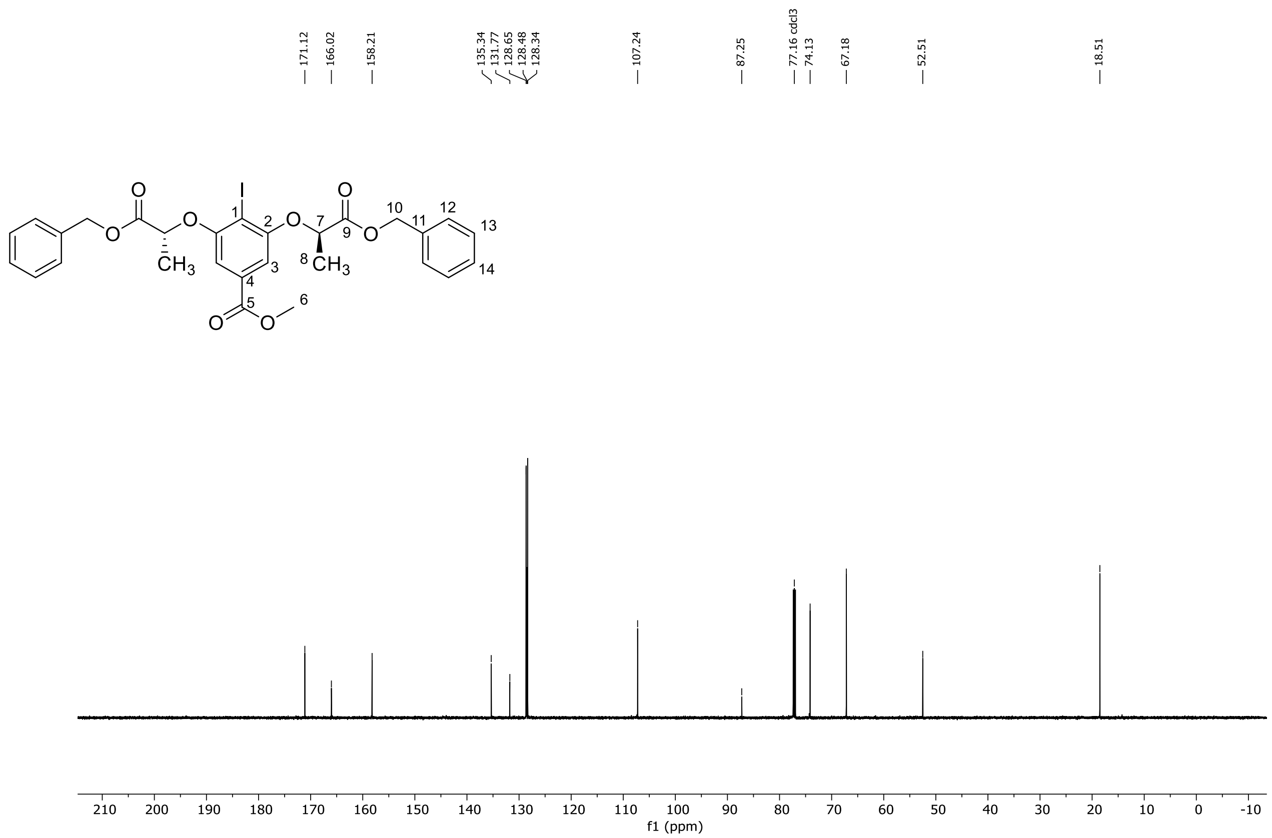

Methyl 3,5-bis(((R)-1-(dimethylamino)-1-oxopropan-2-yl)oxy)-4-iodobenzoate (15)

^{1}H NMR (599 MHz, $CDCl_3$)

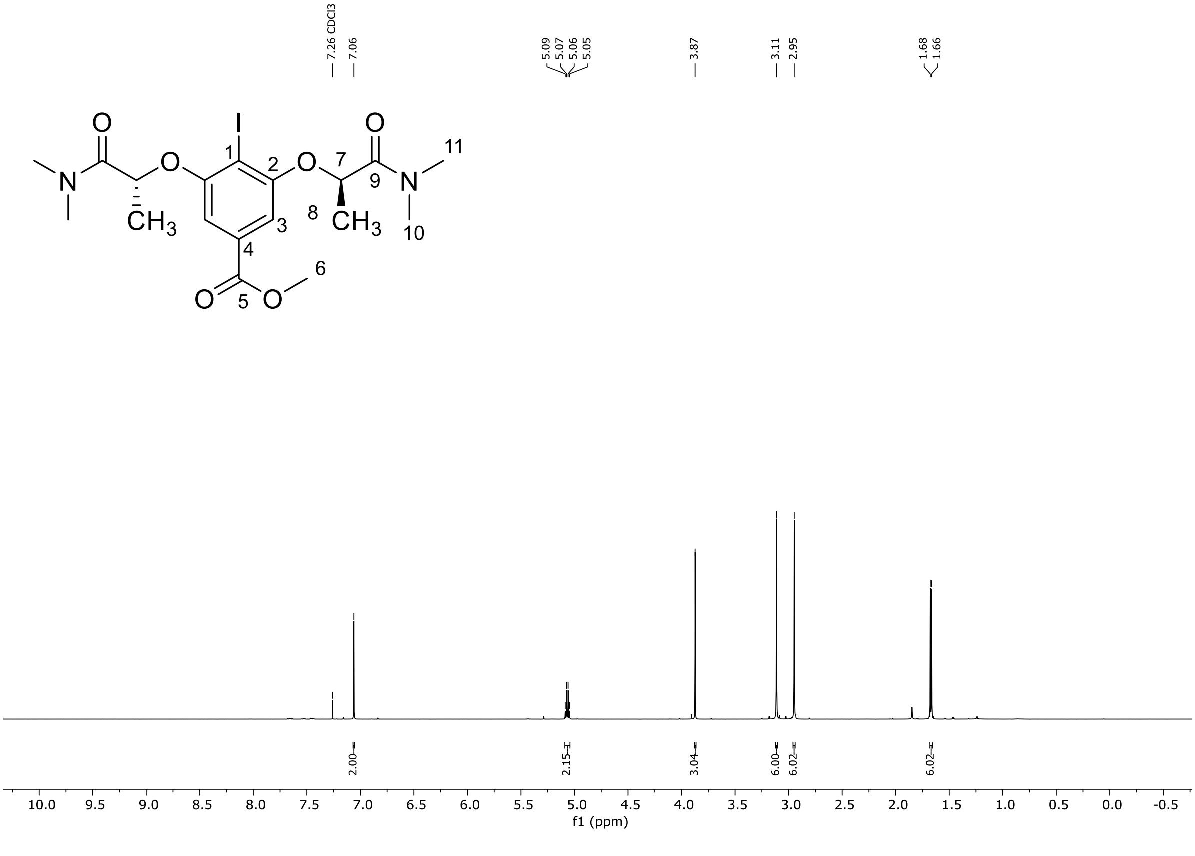

^{13}C NMR (151 MHz, $CDCl_3$)

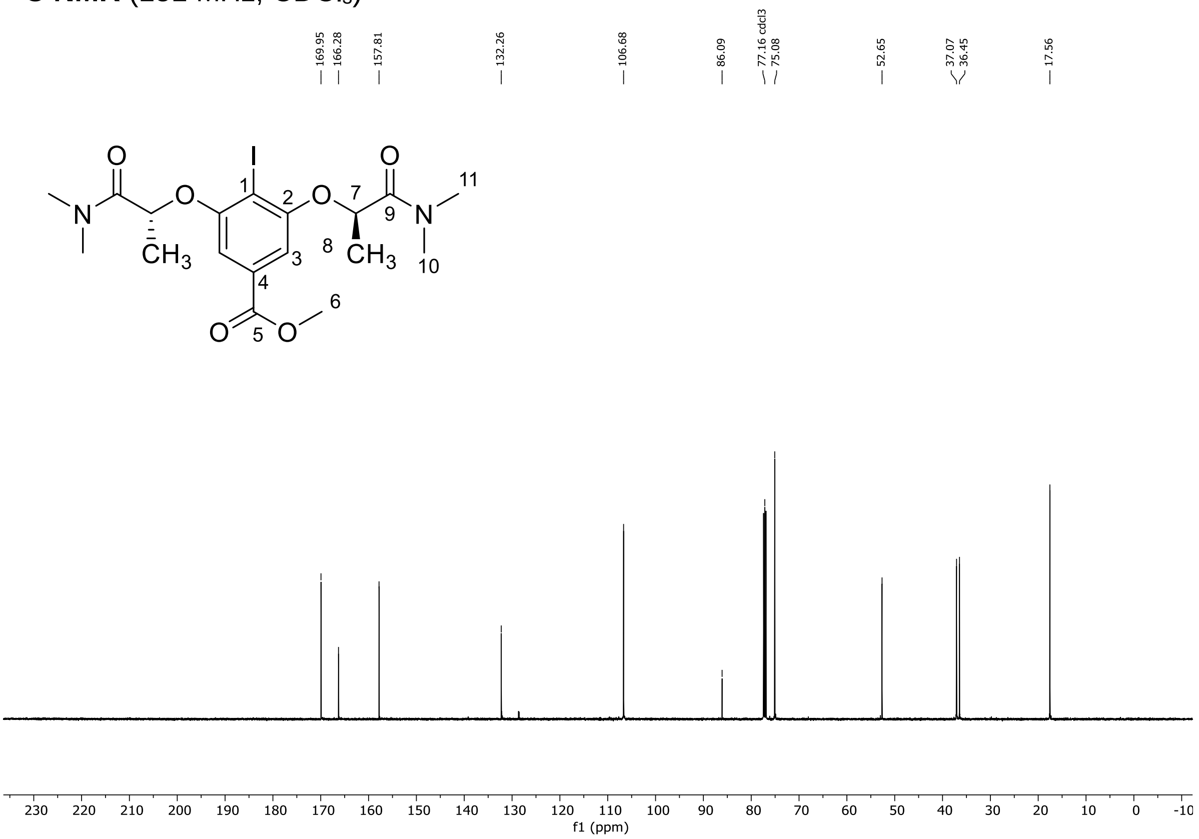

Methyl 4-iodo-3,5-bis(((*R*)-1-(methylamino)-1-oxopropan-2-yl)oxy)benzoate (16)

^{1}H NMR (599 MHz, $CDCl_3$)

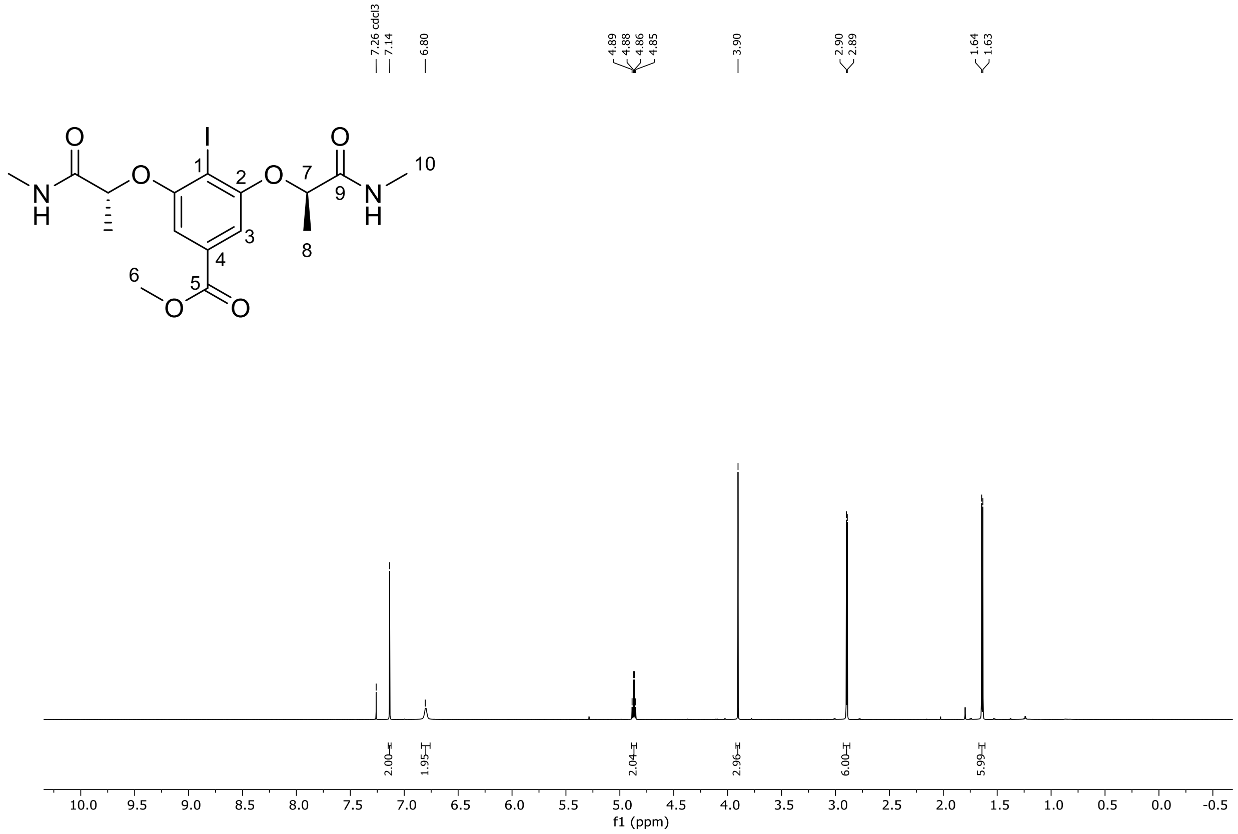

^{13}C NMR (151 MHz, $CDCl_3$)

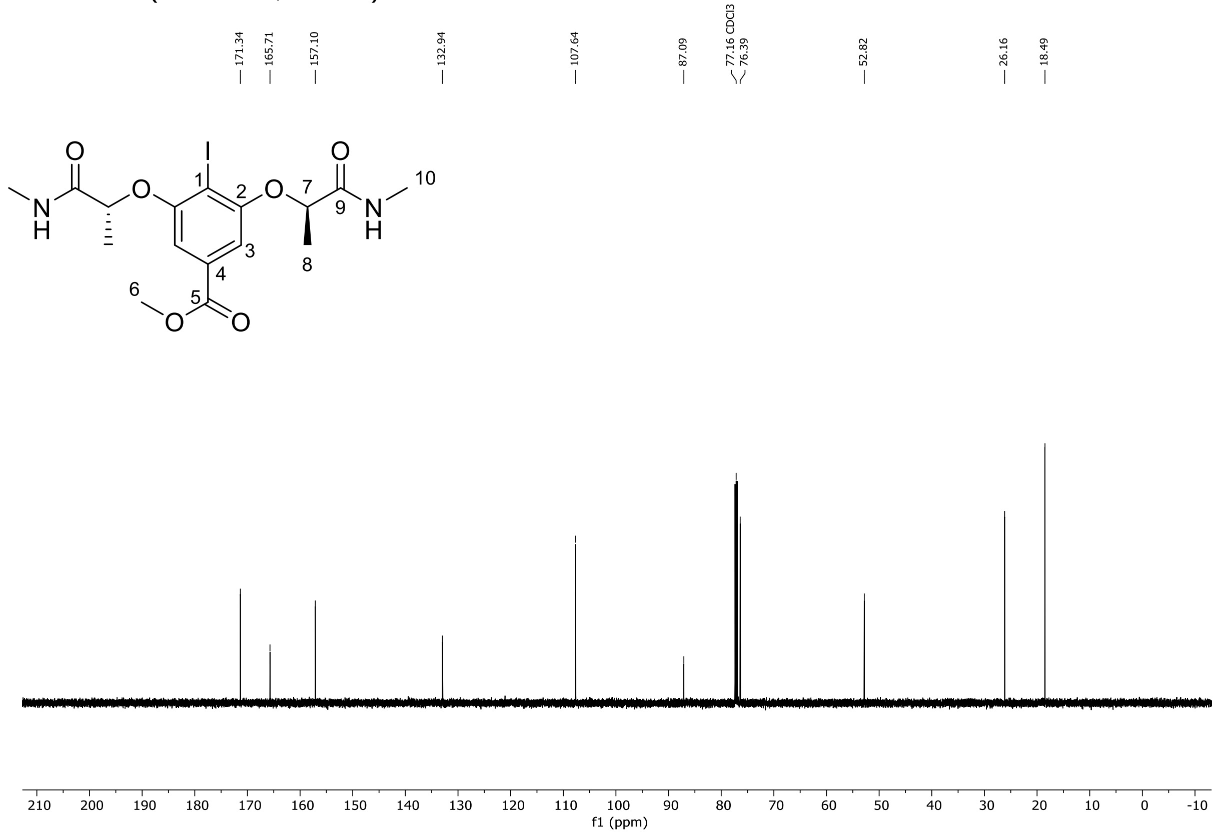

Methyl (*R*)-4-iodo-3-((1-methoxy-1-oxopropan-2-yl)oxy)benzoate (17, STM-2221)

^{1}H NMR (599 MHz, $CDCl_3$)

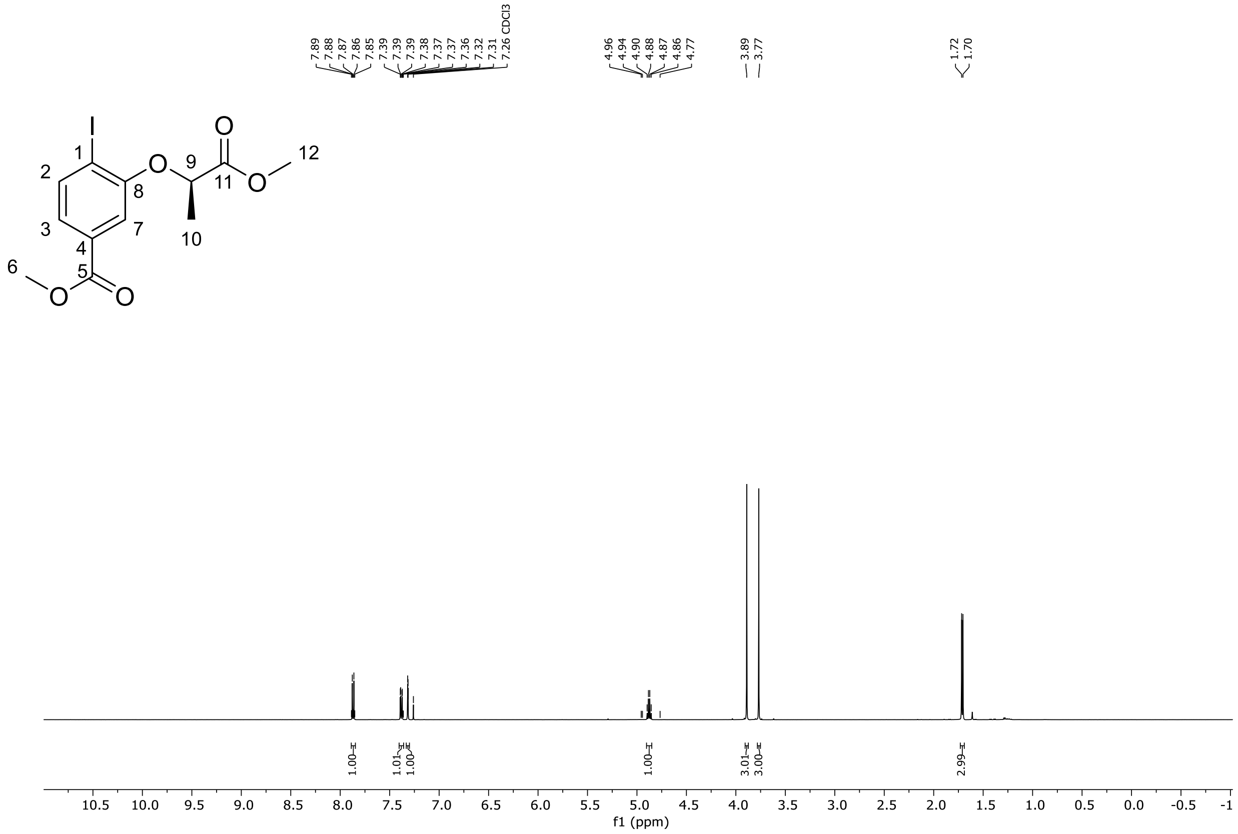

^{13}C NMR (151 MHz, $CDCl_3$)

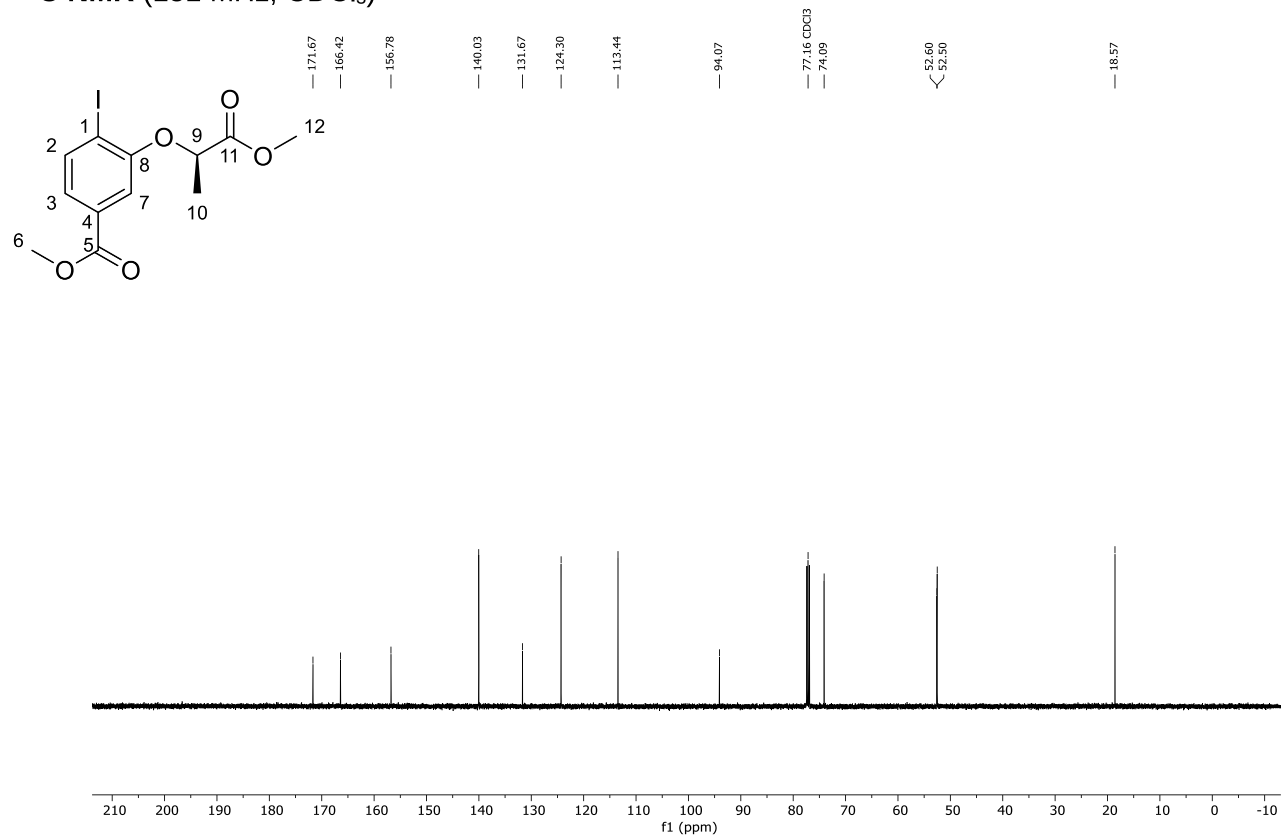

Methyl (*R*)-3-((1-(benzyloxy)-1-oxopropan-2-yl)oxy)-4-iodobenzoate (18)

^{1}H NMR (599 MHz, $CDCl_3$)

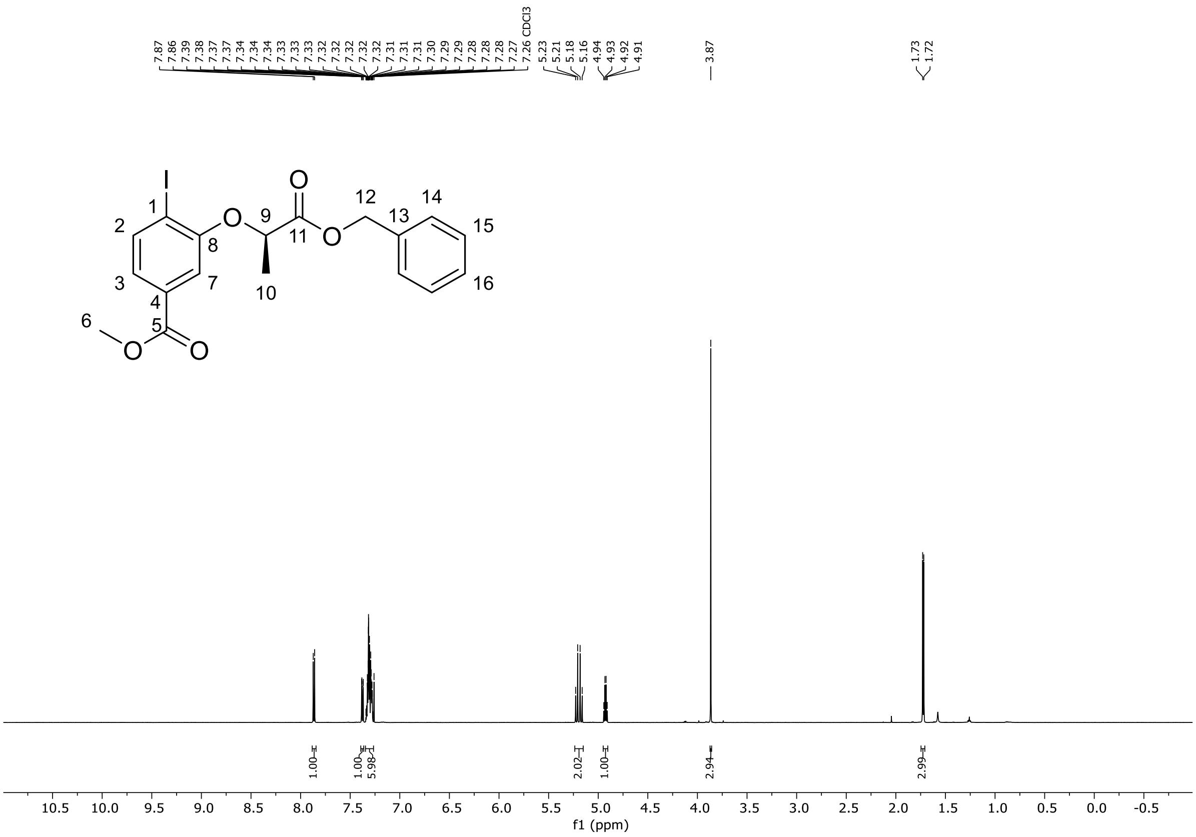

^{13}C NMR (151 MHz, $CDCl_3$)

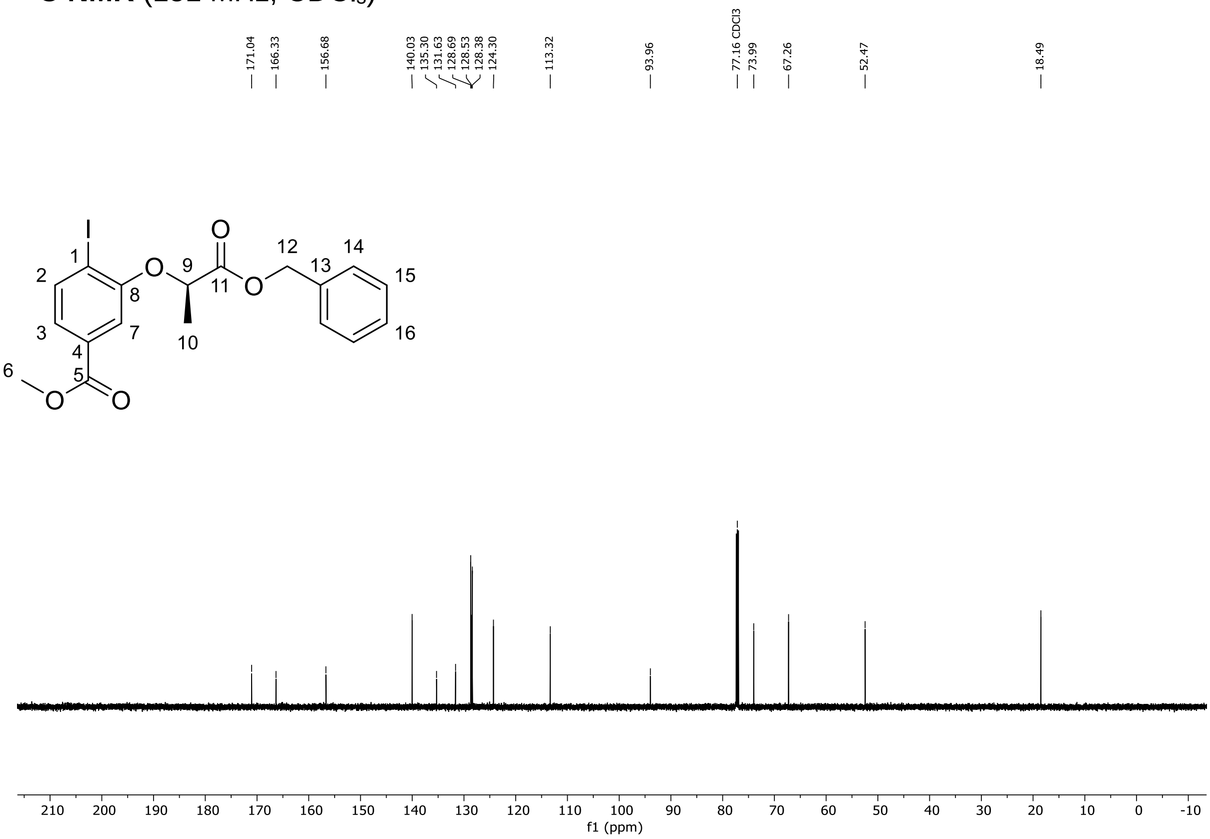

Methyl (*R*)-4-iodo-3-((1-methoxy-1-oxo-3-phenylpropan-2-yl)oxy)benzoate (19)

^{1}H NMR (599 MHz, $CDCl_3$)

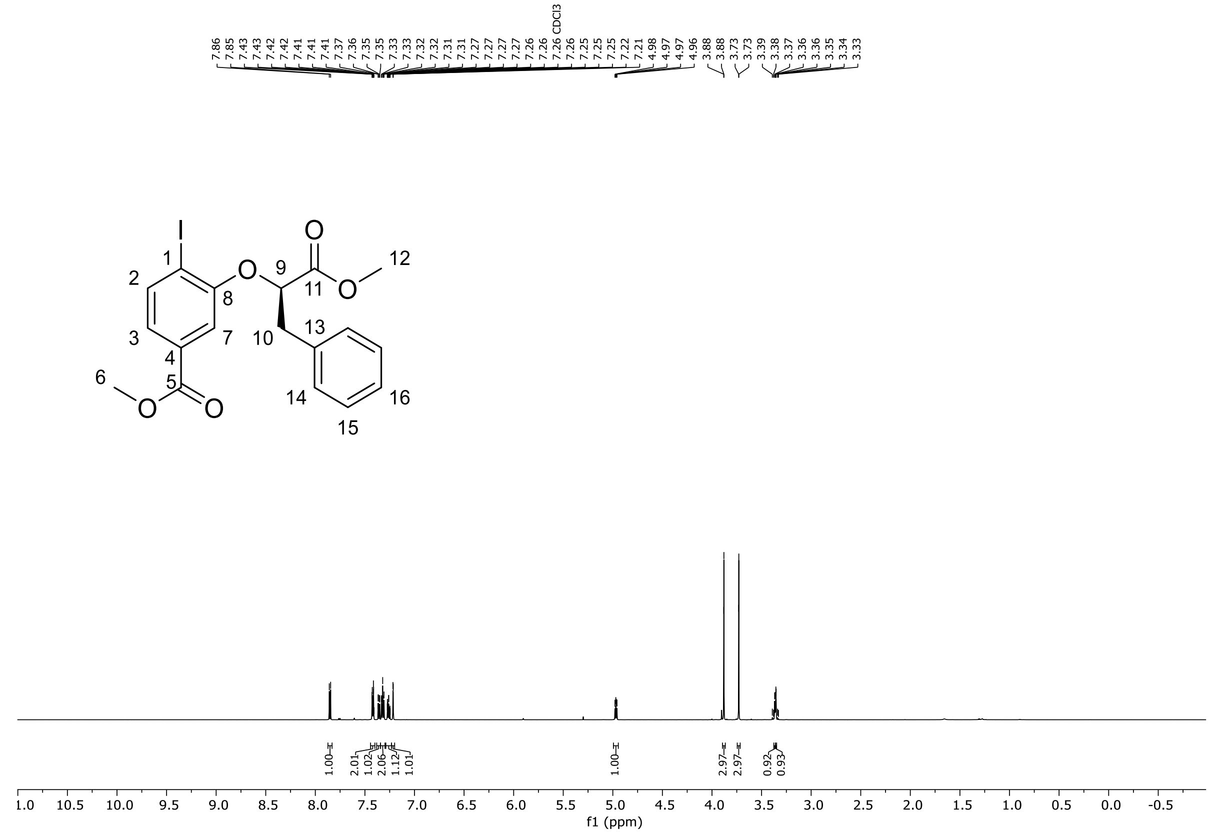

^{13}C NMR (151 MHz, $CDCl_3$)

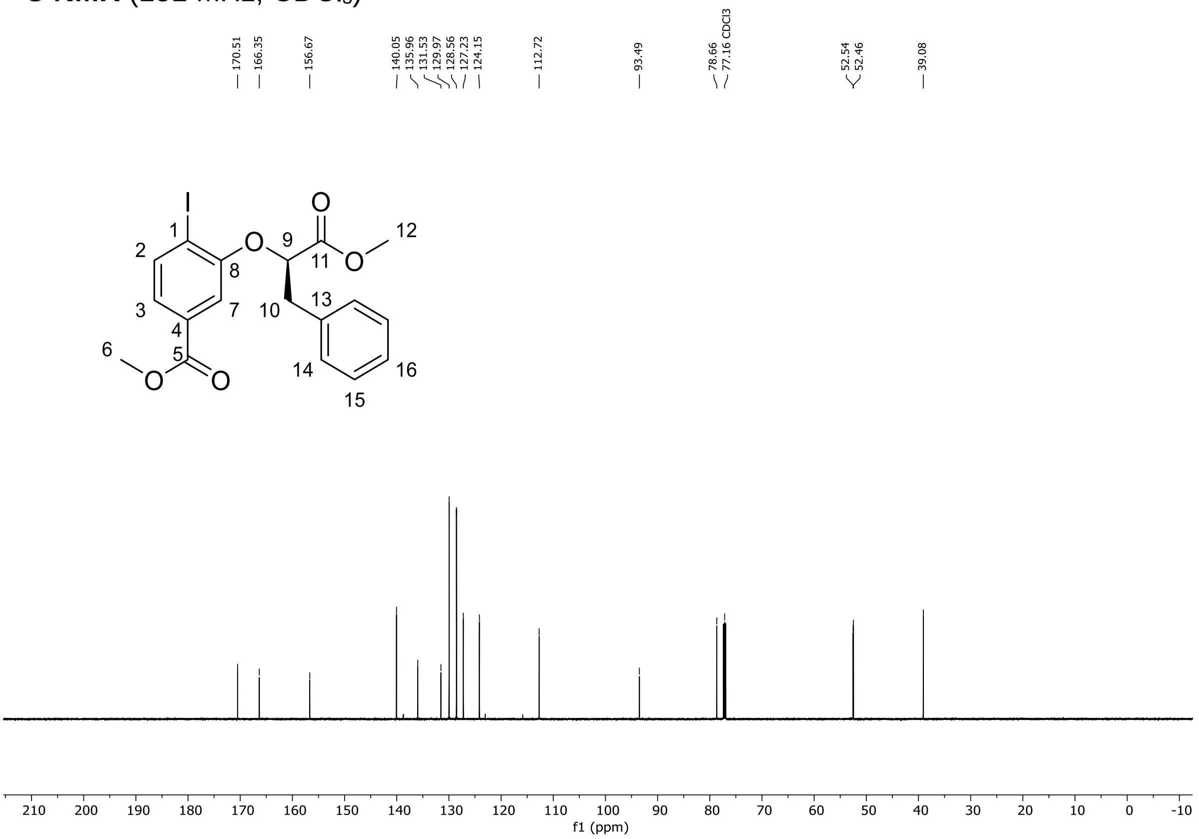

NMR of Fluorinated Products

1-Chloro-4-(2,2,3,3,3-pentafluoropropyl)benzene (3a)
^{1}H NMR (599 MHz, $CDCl_3$)

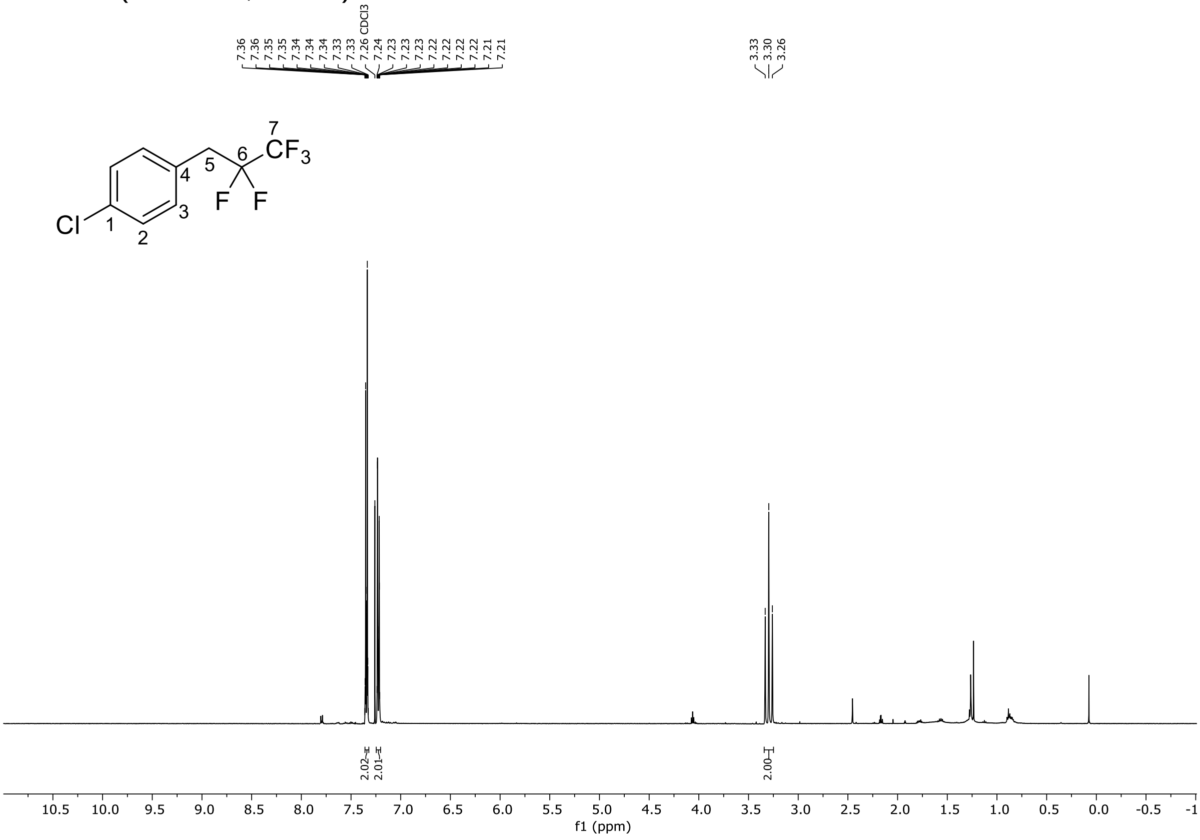

^{13}C NMR (151 MHz, $CDCl_3$)

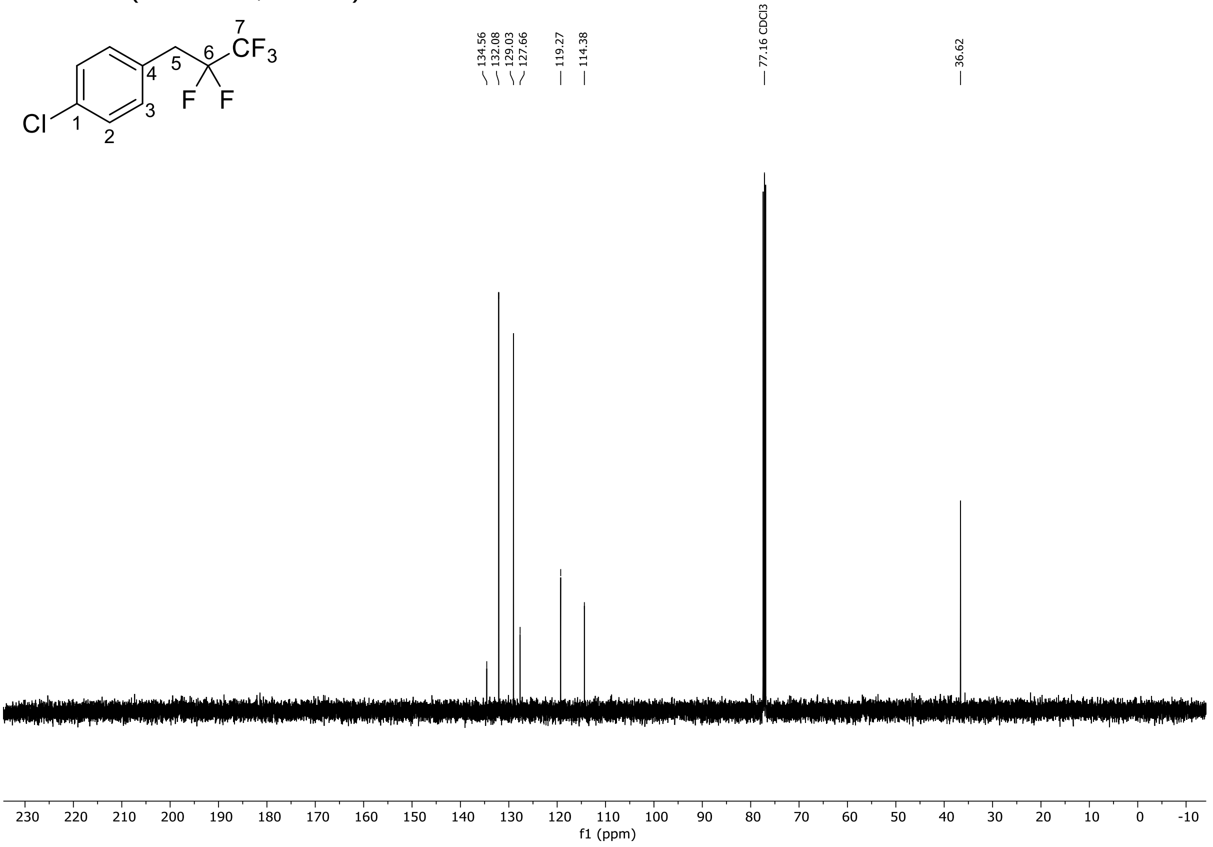

^{19}F NMR (376 MHz, $CDCl_3$)

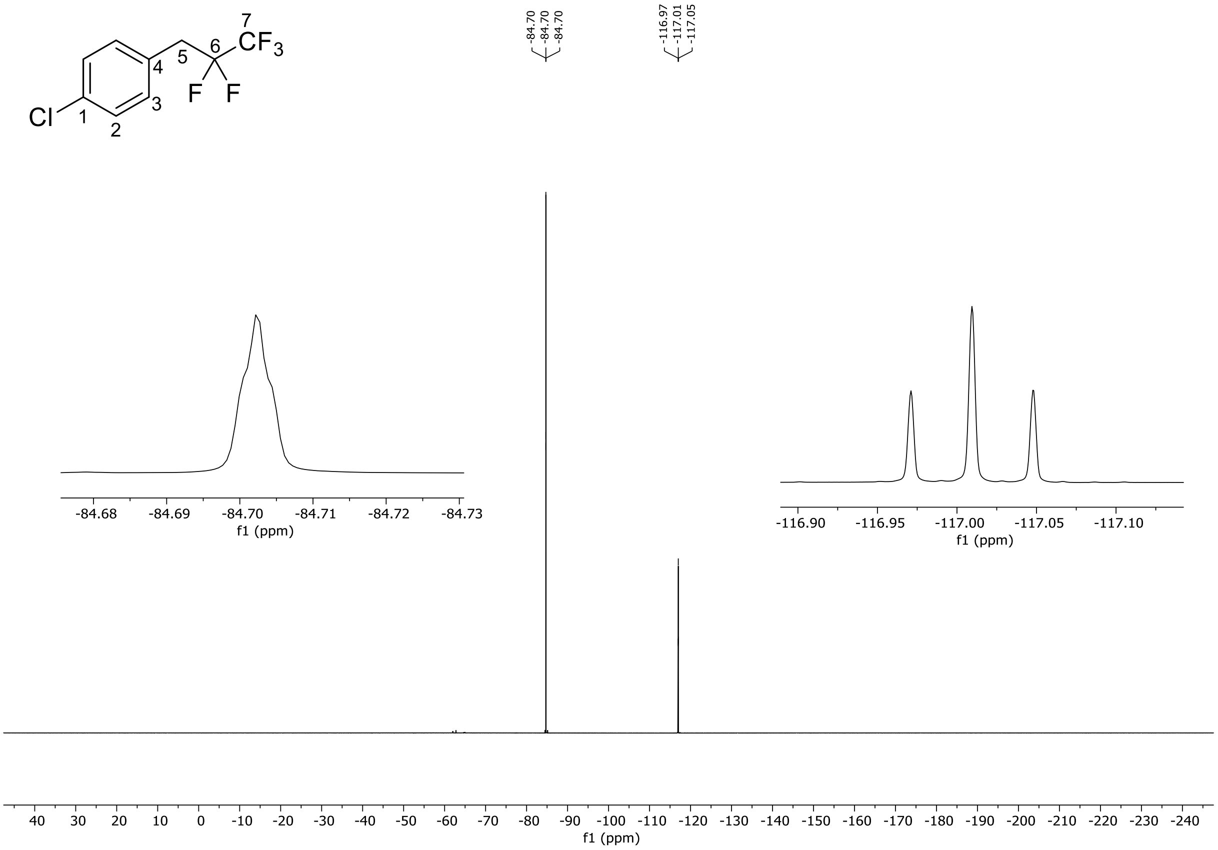

$^{19}F\{^1H\}$ NMR (376 MHz, $CDCl_3$)

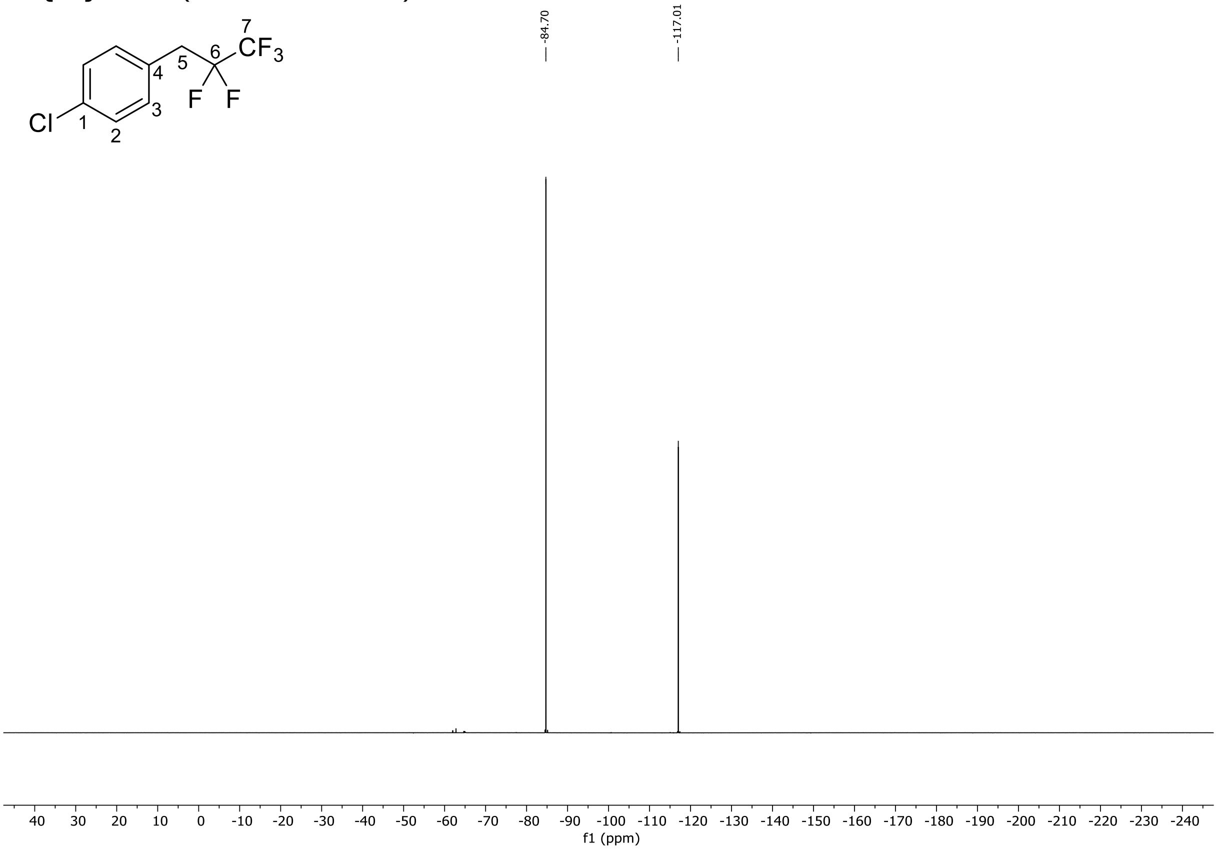

1-Chloro-4-(1,1,1,2,3-pentafluoropropan-2-yl)benzene (2a)

^1H NMR (5996MHz, $CDCl_3$)

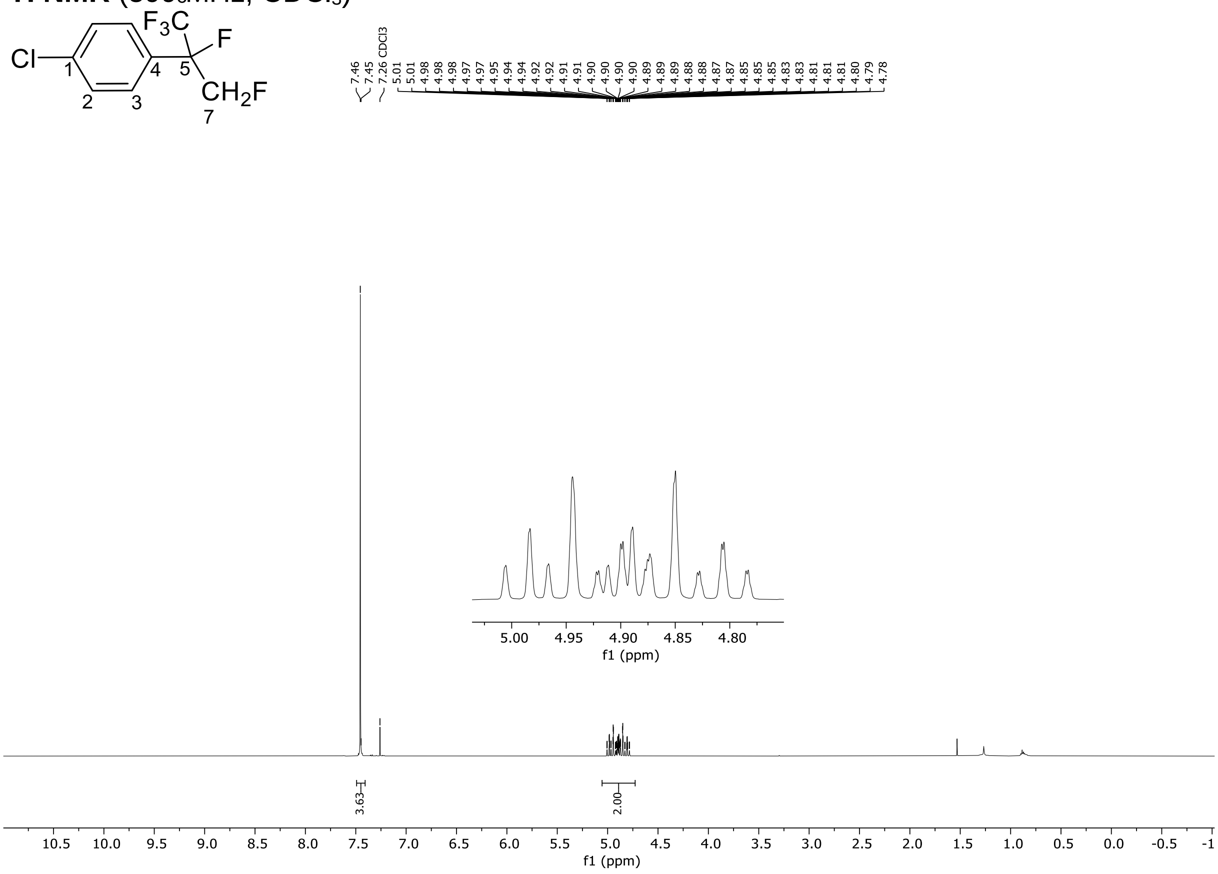

^{13}C NMR (151 MHz, $CDCl_3$)

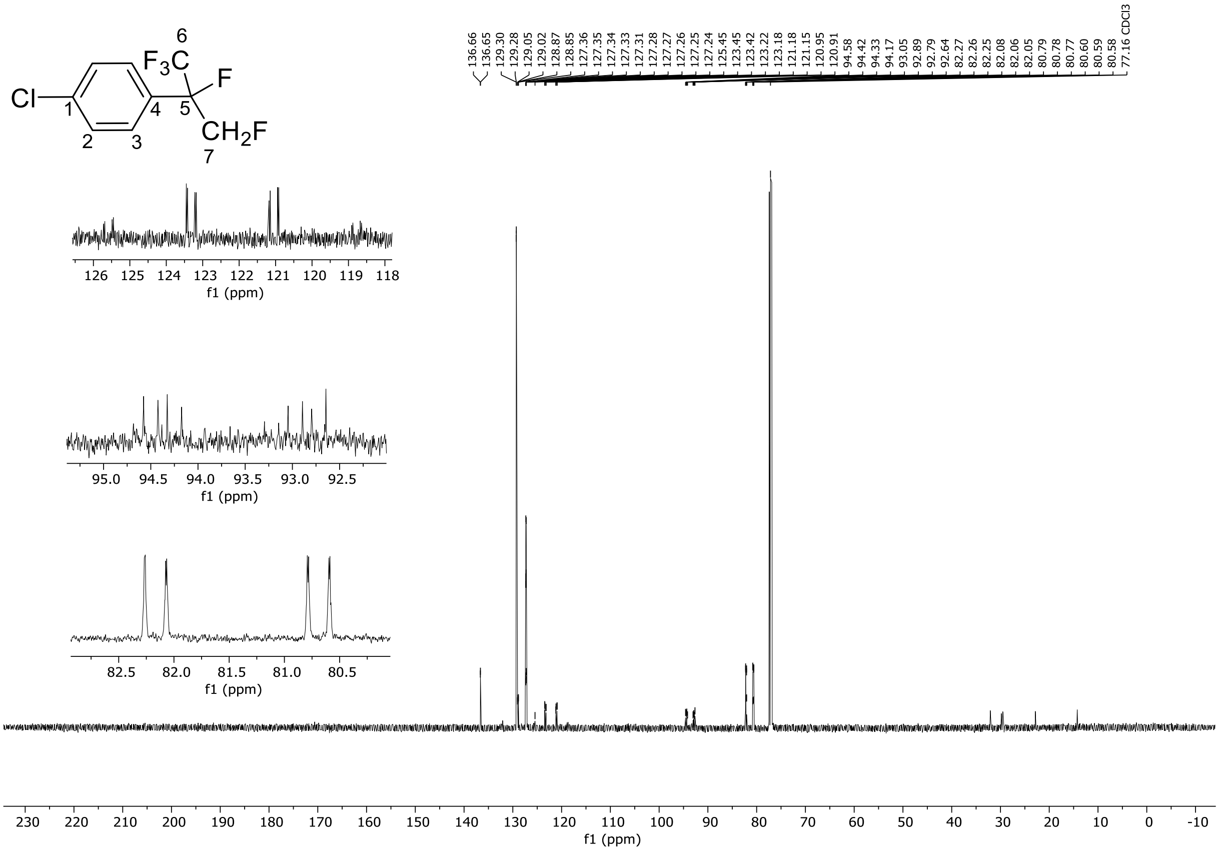

^{19}F NMR (376 MHz, $CDCl_3$)

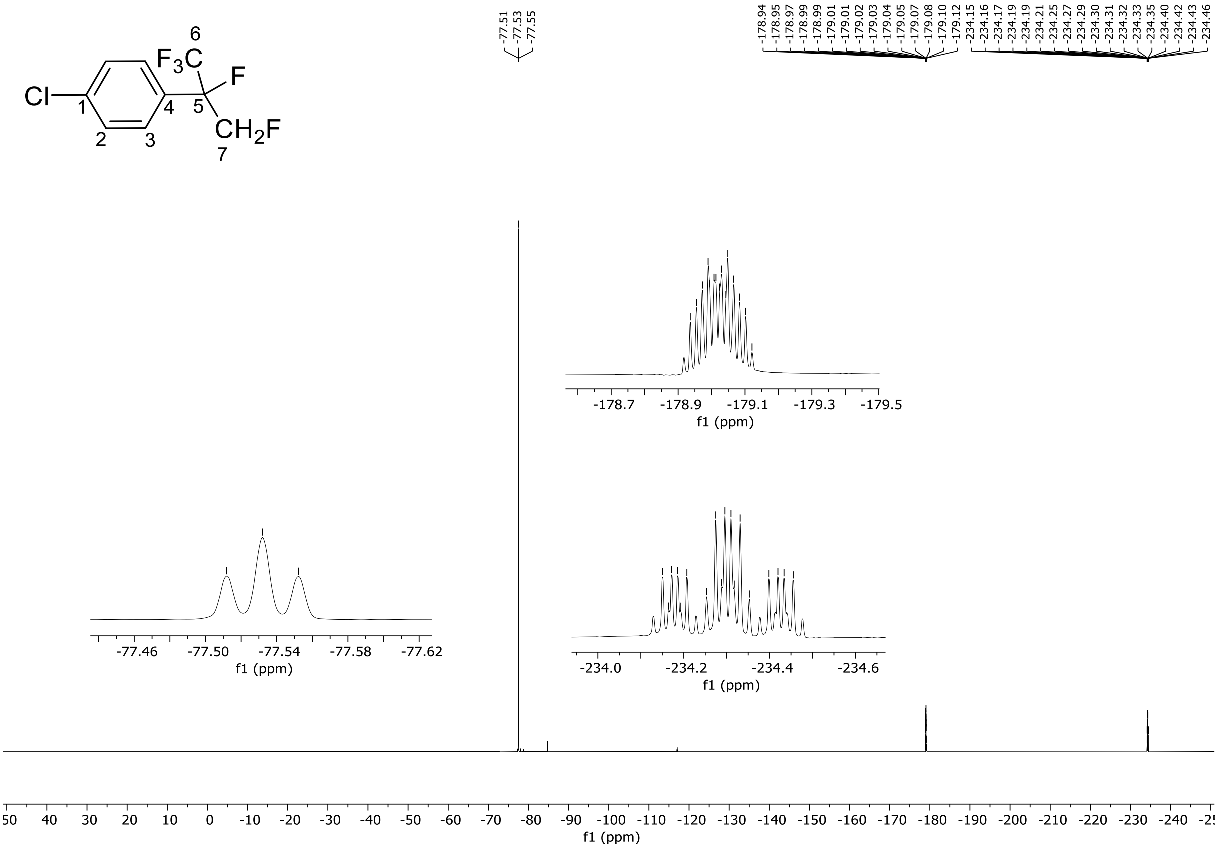

$^{19}F\{^{1}H\}$ NMR (376 MHz, $CDCl_3$)

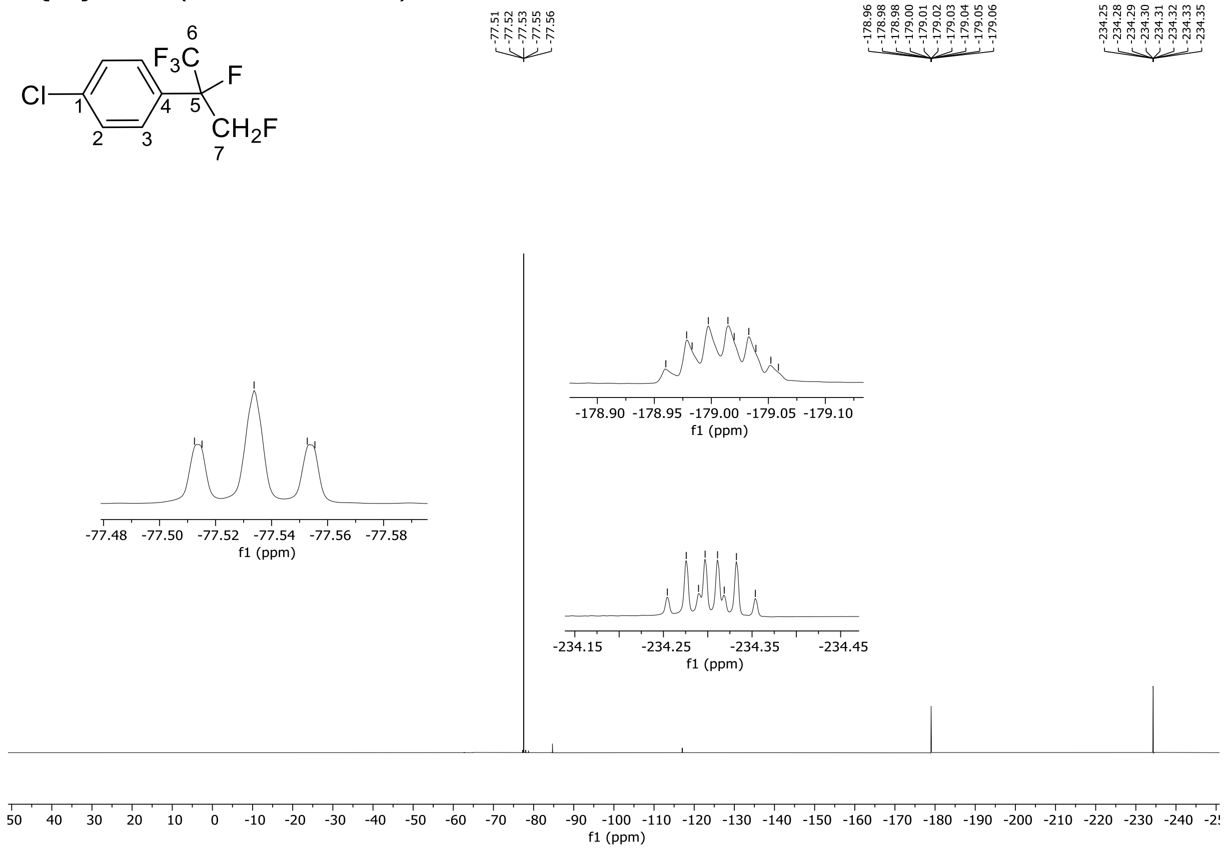

1-Methoxy-4-(1,1,1,2,3-pentafluoropropan-2-yl)benzene (2b)

^{1}H NMR (500 MHz, $CDCl_3$)

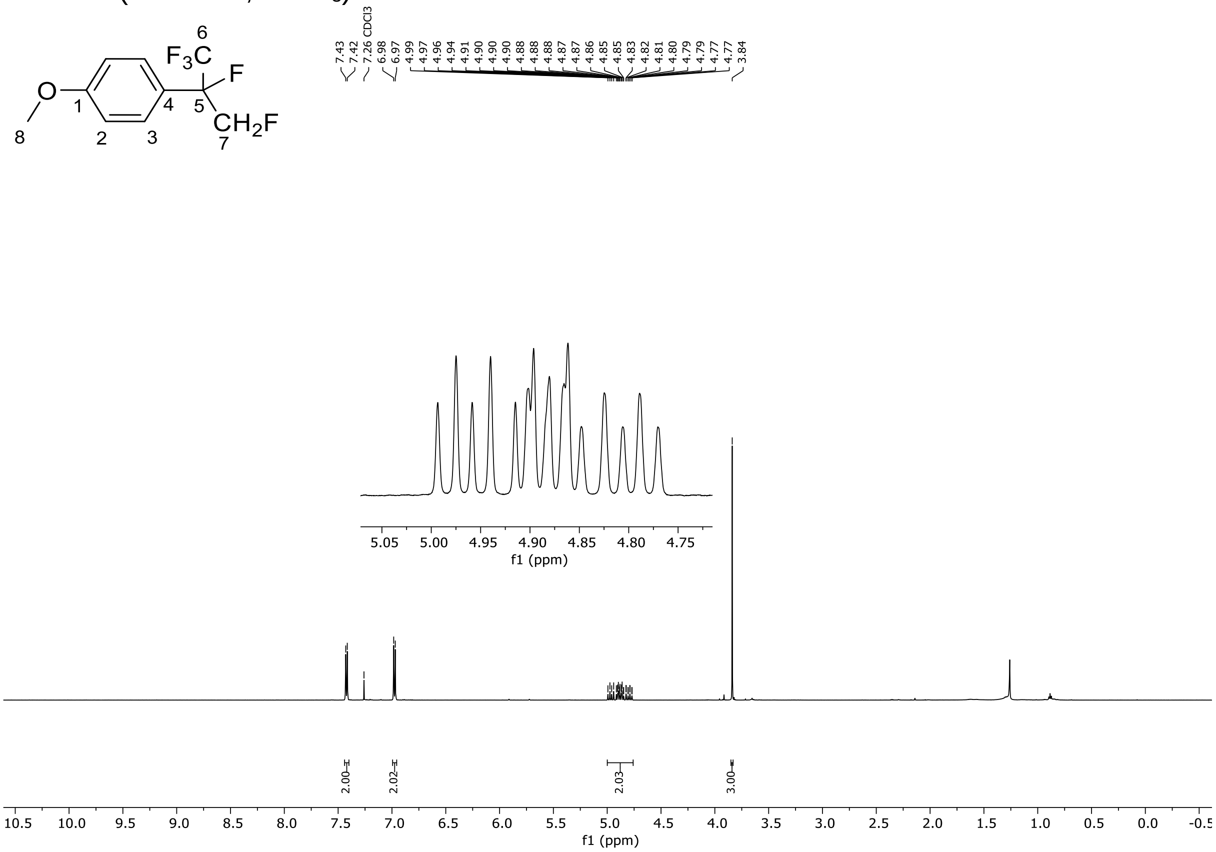

^{13}C NMR (151 MHz, $CDCl_3$)

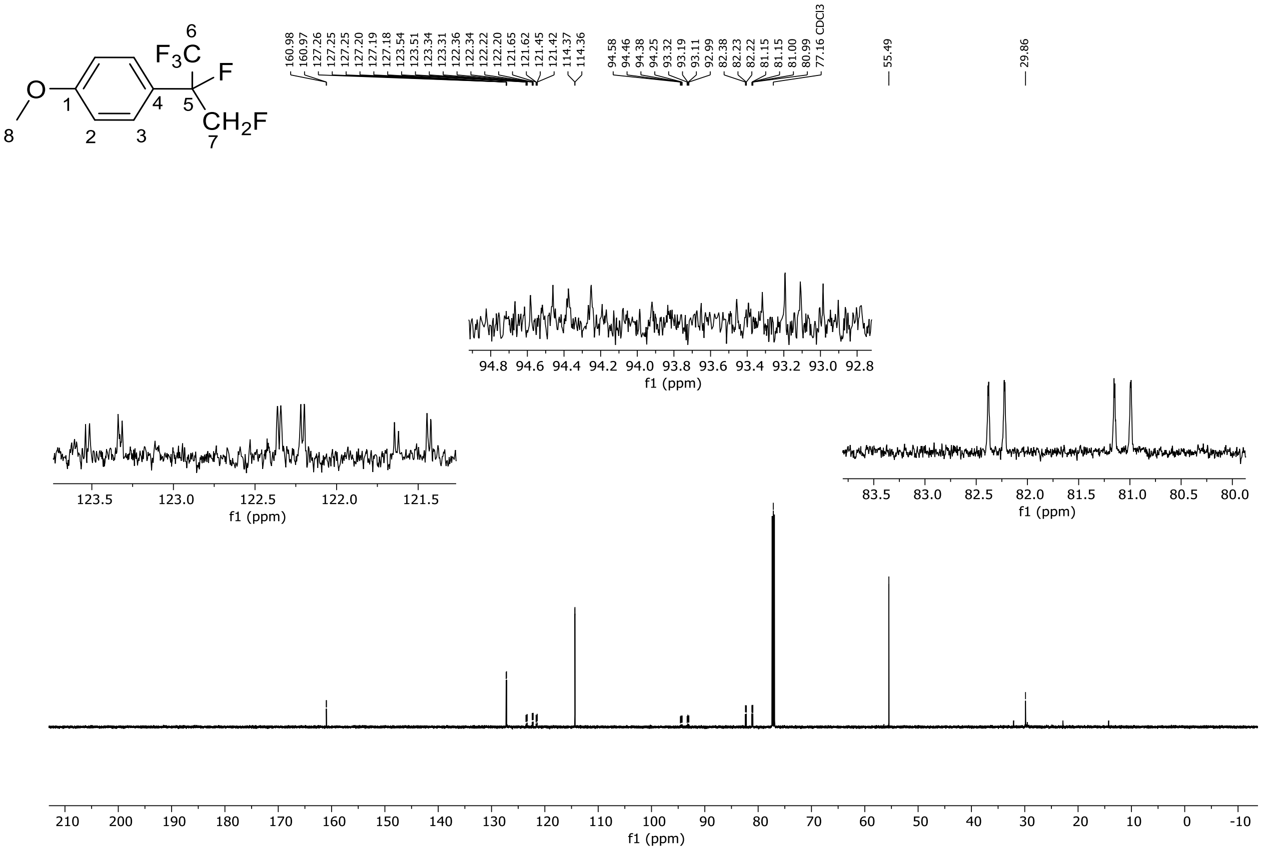

^{19}F NMR (564 MHz, $CDCl_3$)

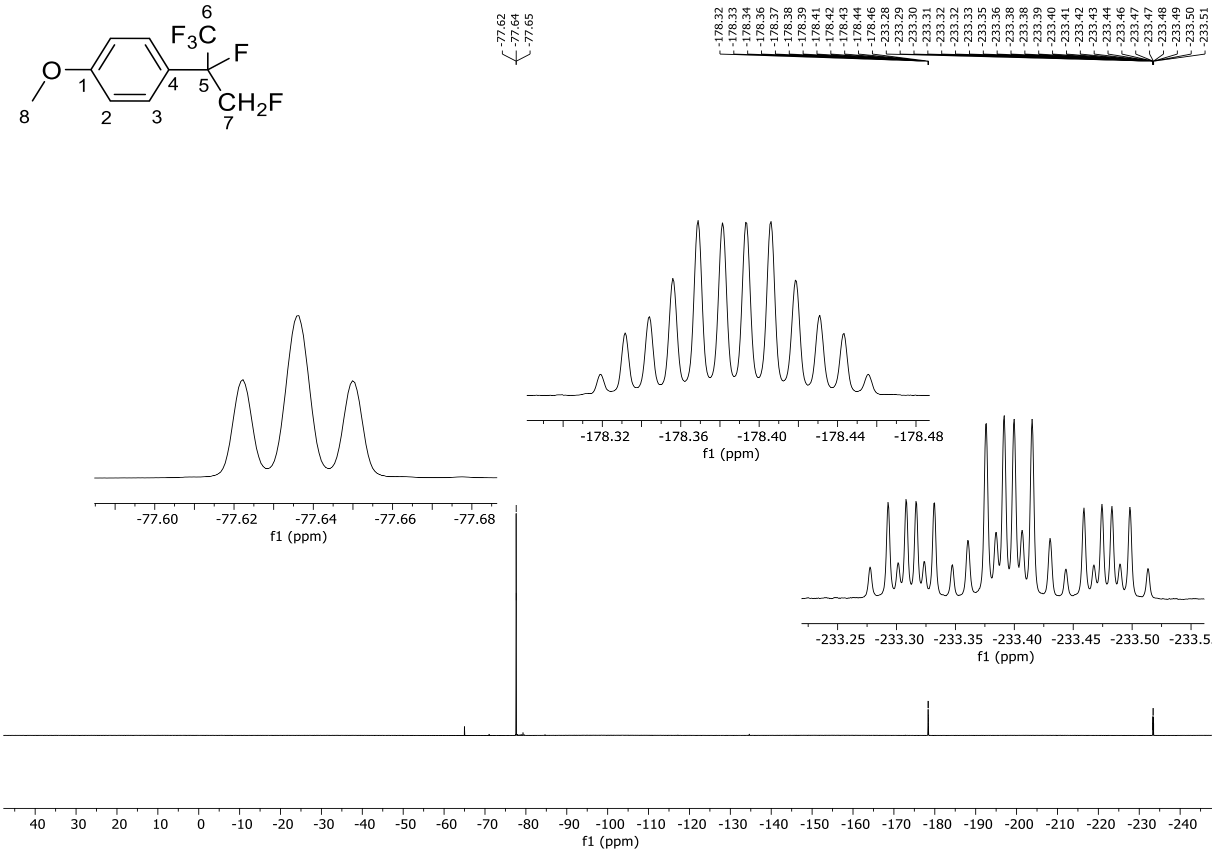

$^{19}F\{^{1}H\}$ NMR (564 MHz, $CDCl_3$)

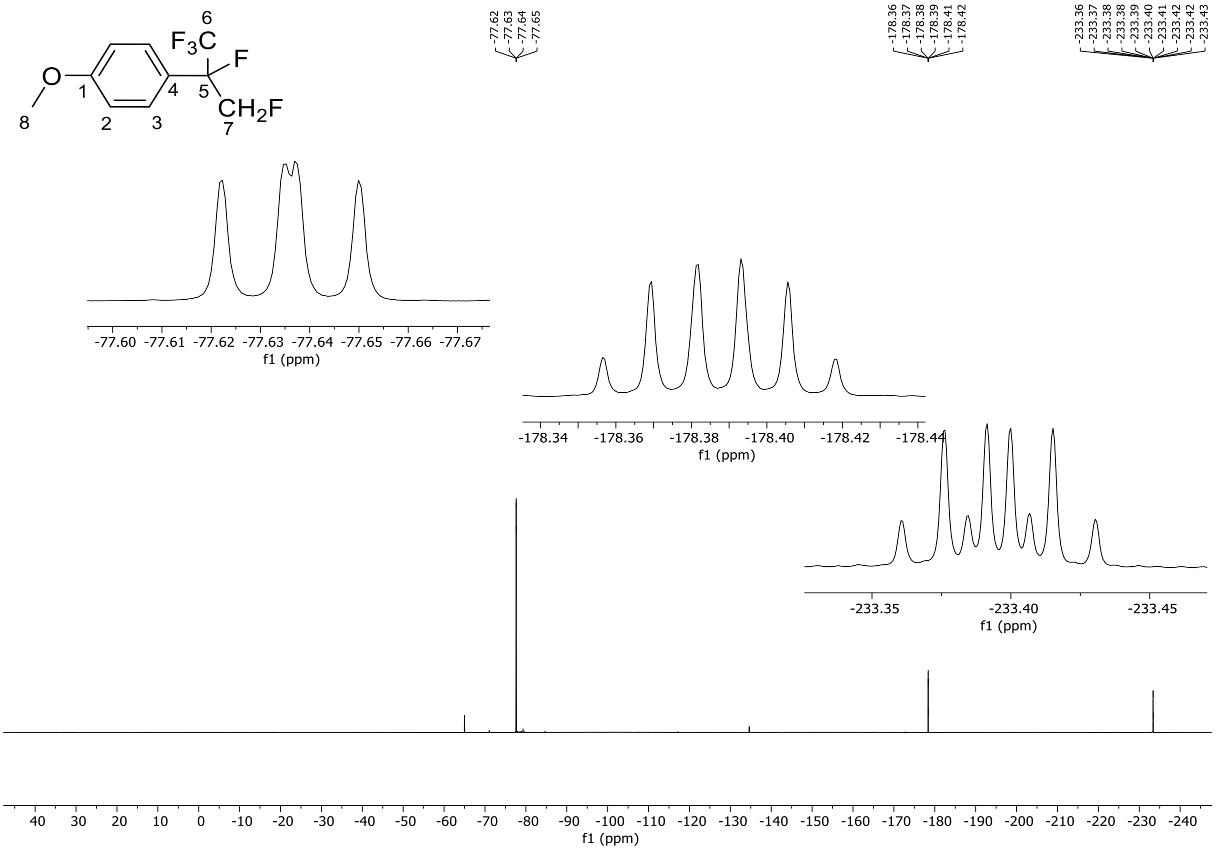

2-Methoxy-1,3-dimethyl-5-(1,1,1,2,3-pentafluoropropan-2-yl)benzene (2c)

^{1}H NMR (599 MHz, $CDCl_3$)

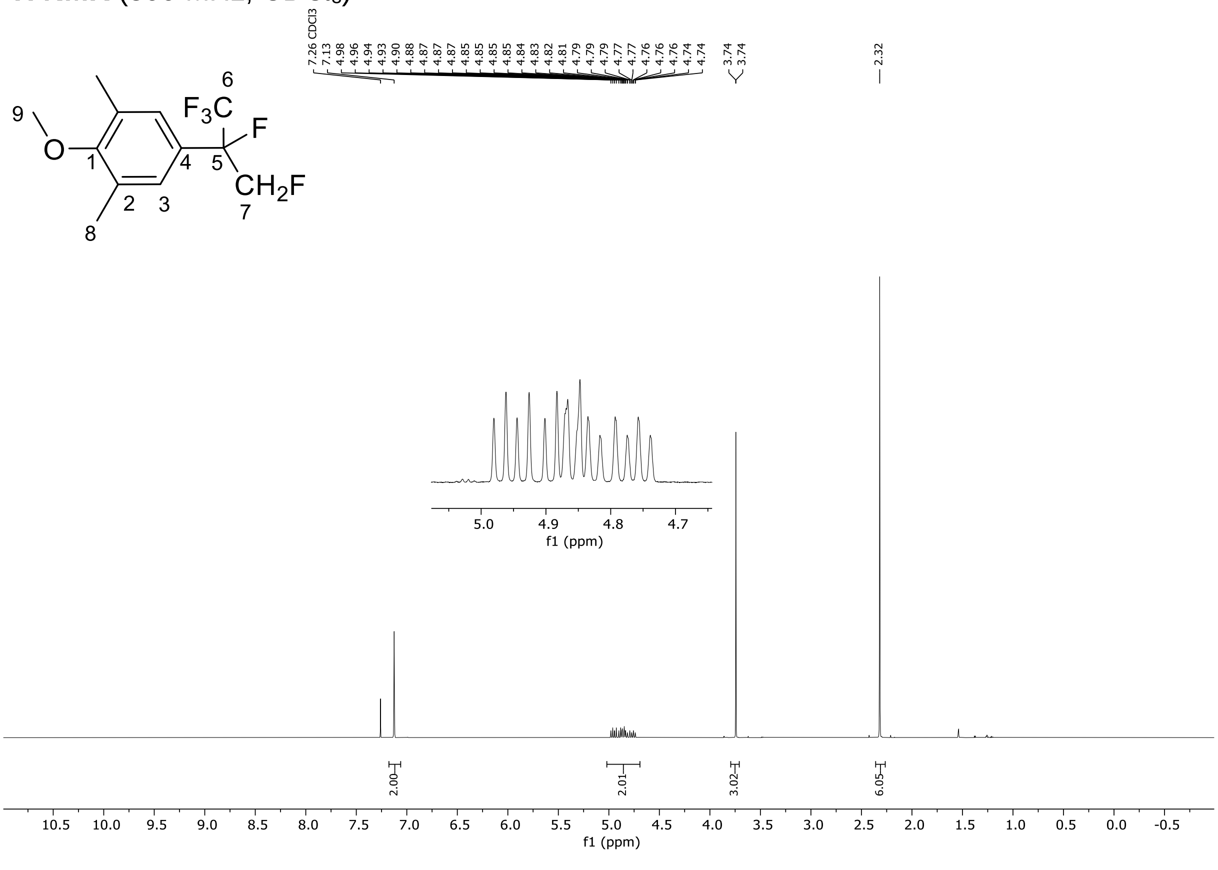

^{13}C NMR (126 MHz, $CDCl_3$)

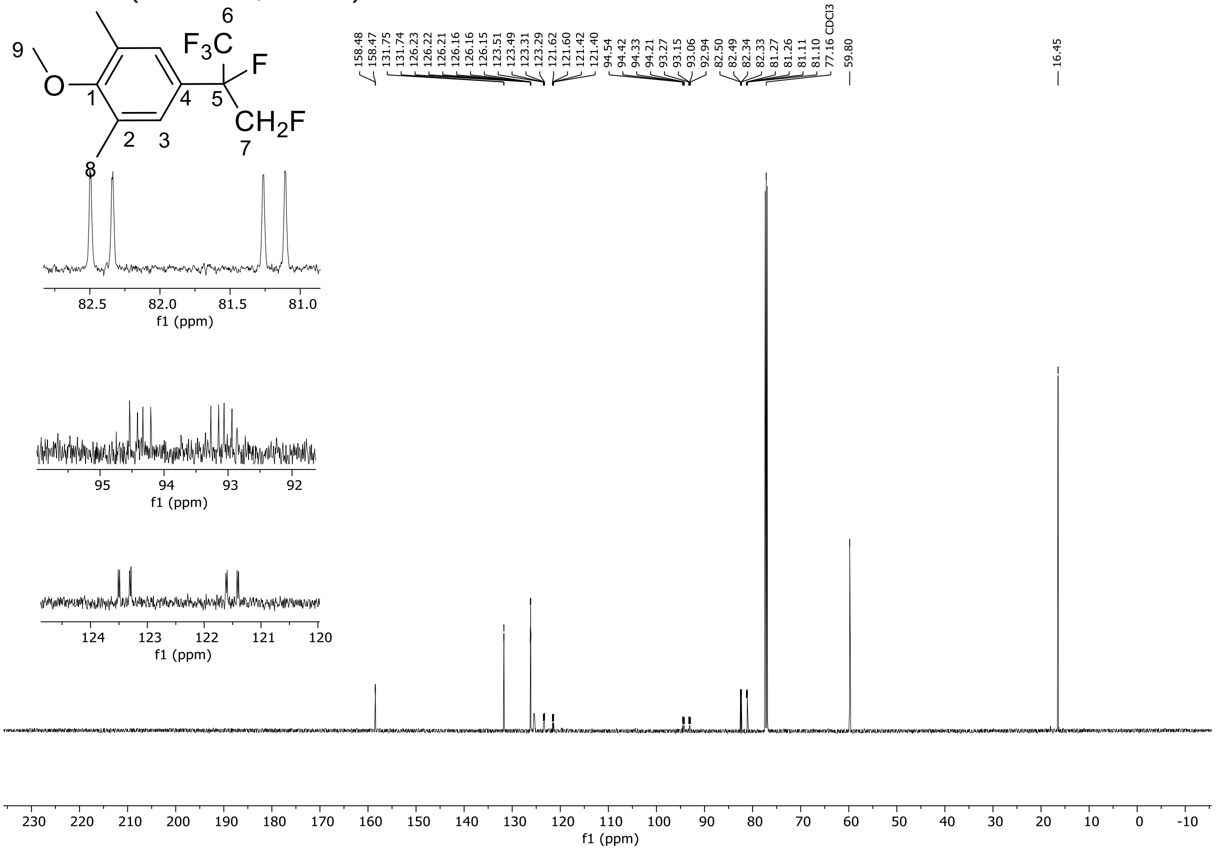

^{19}F NMR (376 MHz, $CDCl_3$)

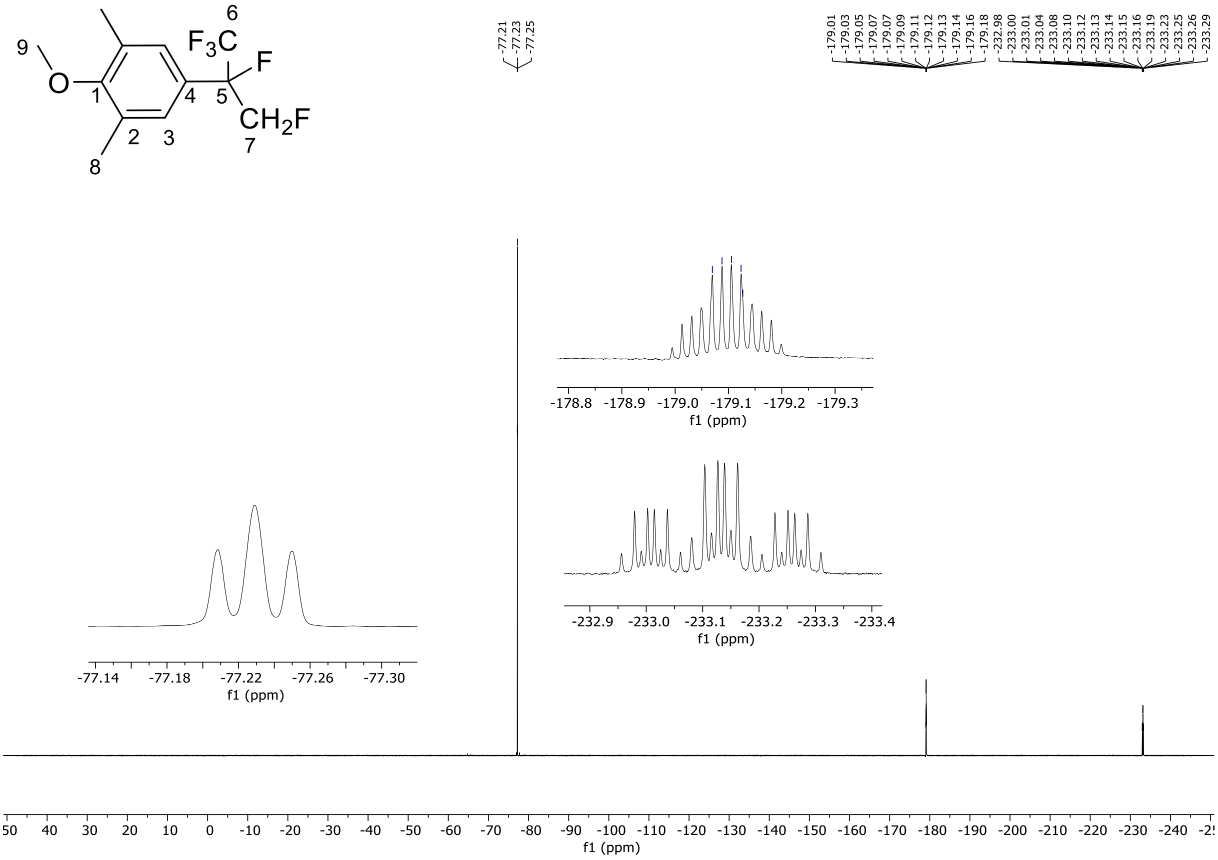

$^{19}F\{^{1}H\}$ NMR (376 MHz, $CDCl_3$)

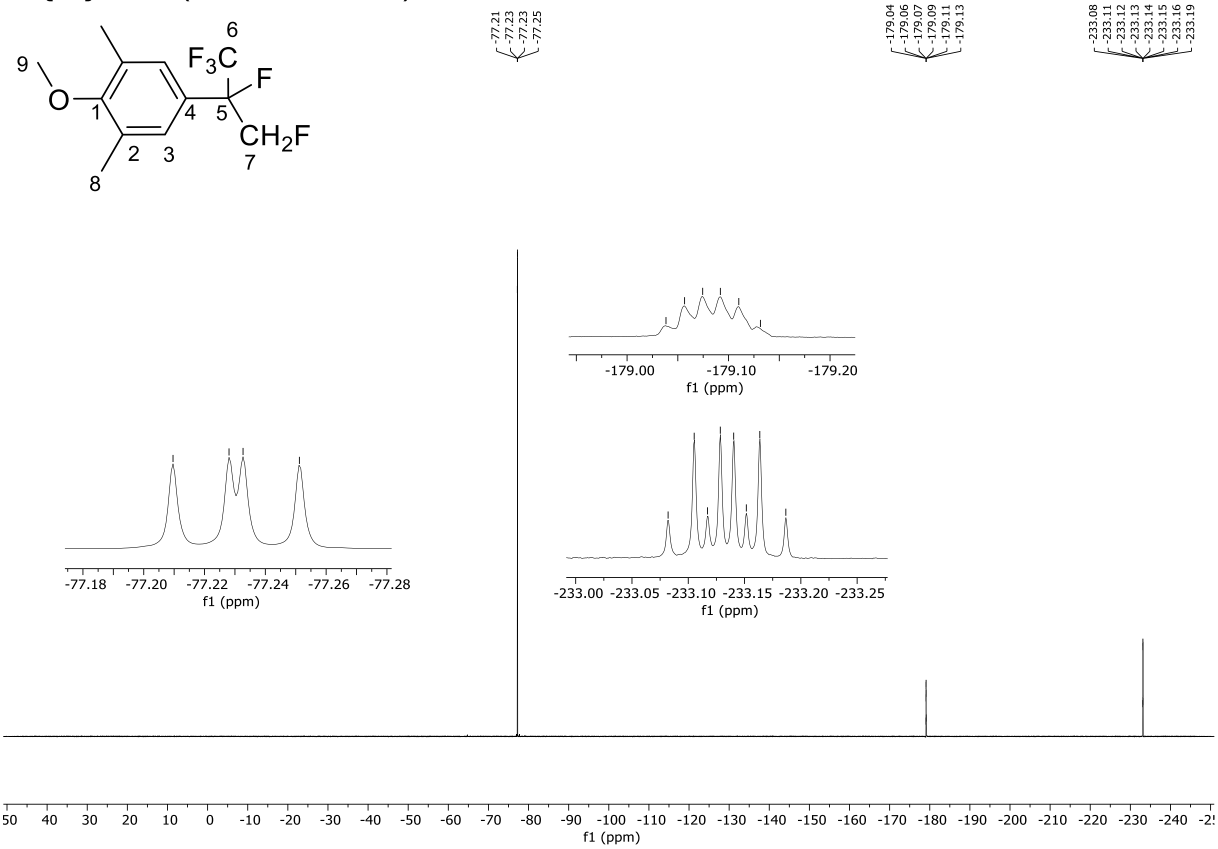

1-Bromo-4-((4-(1,1,1,2,3-pentafluoropropan-2-yl)phenoxy)methyl)benzene (2d)

^{1}H NMR (599 MHz, $CDCl_3$)

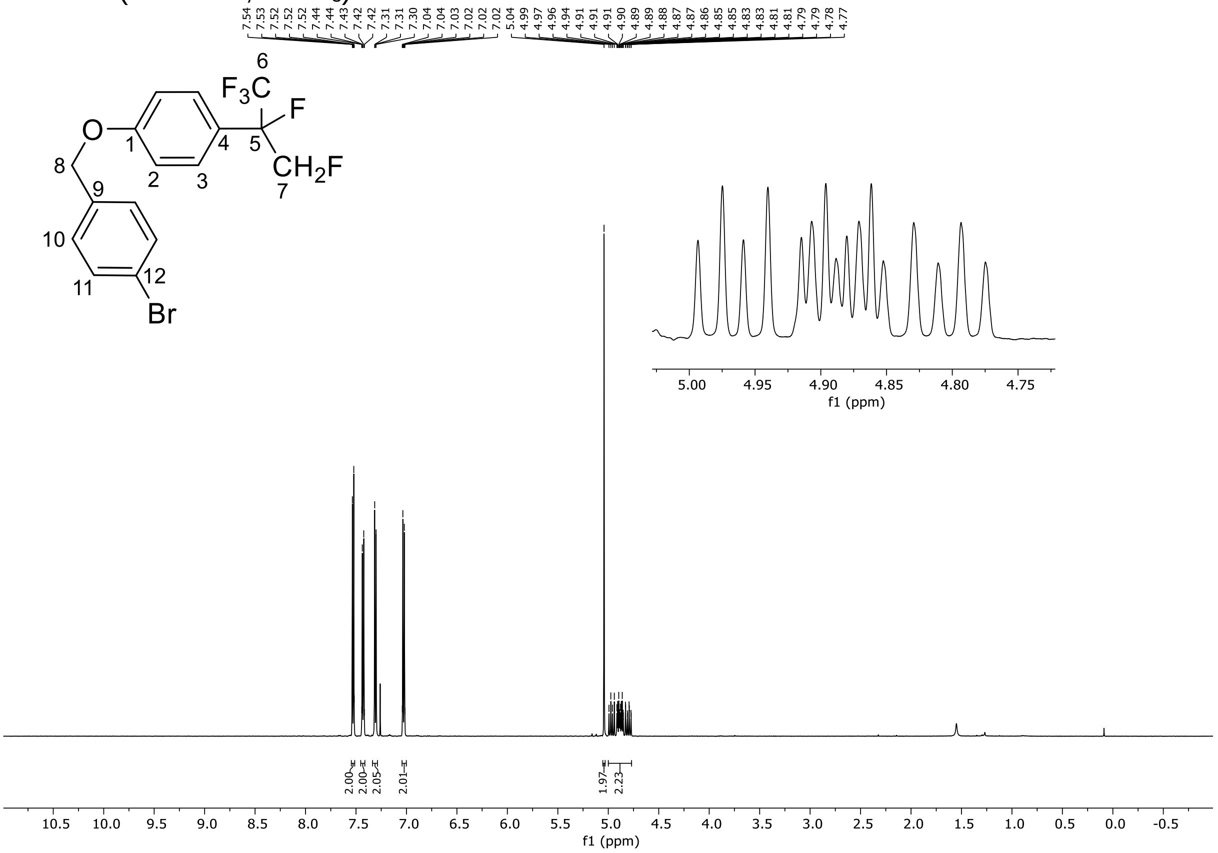

^{13}C NMR (151 MHz, $CDCl_3$)

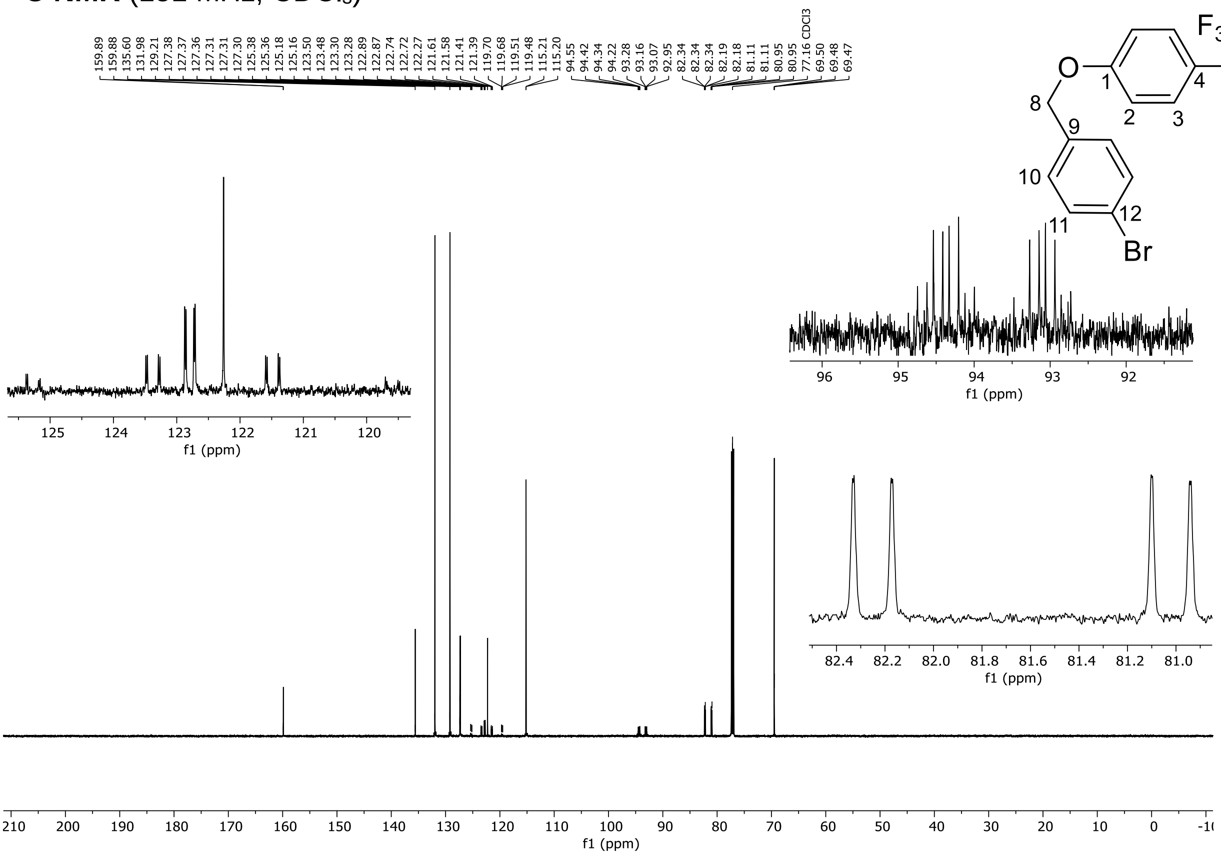

^{19}F NMR (564 MHz, $CDCl_3$)

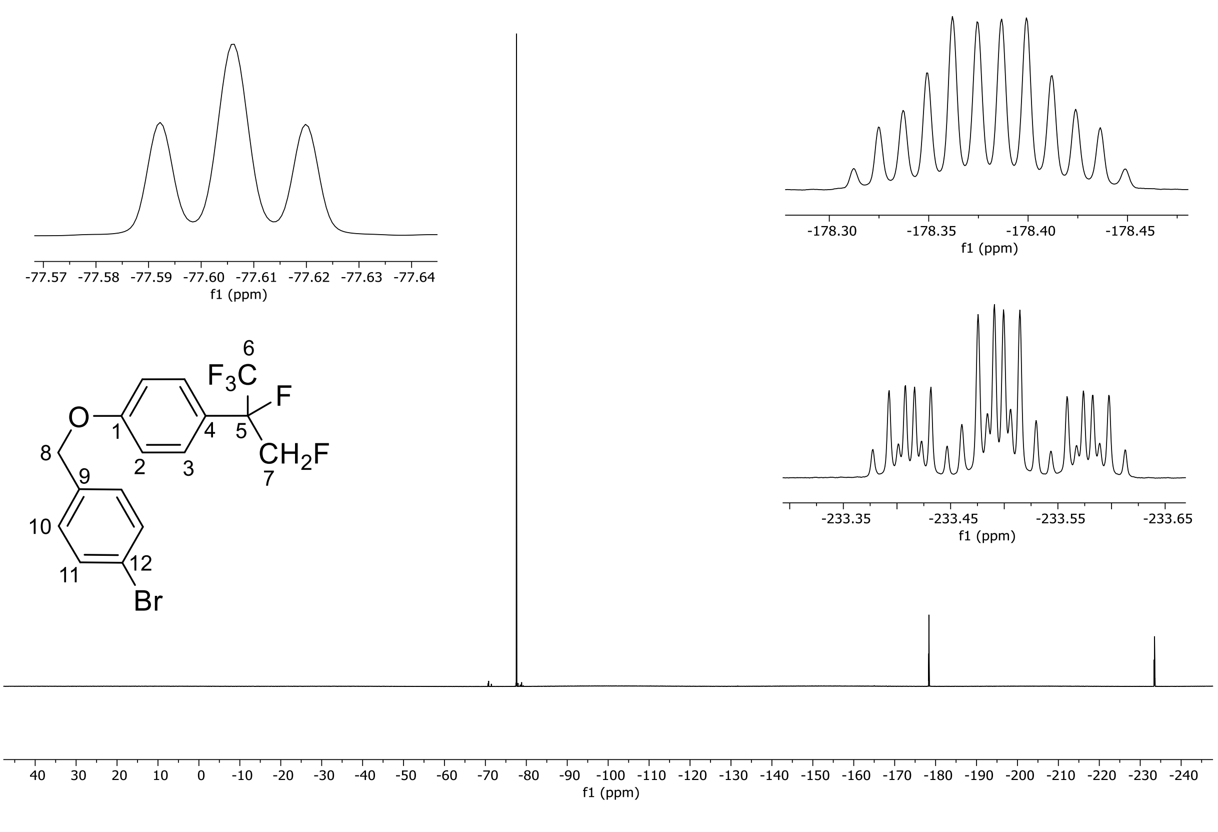

^{19}F{^{1}H} NMR (564 MHz, $CDCl_3$)

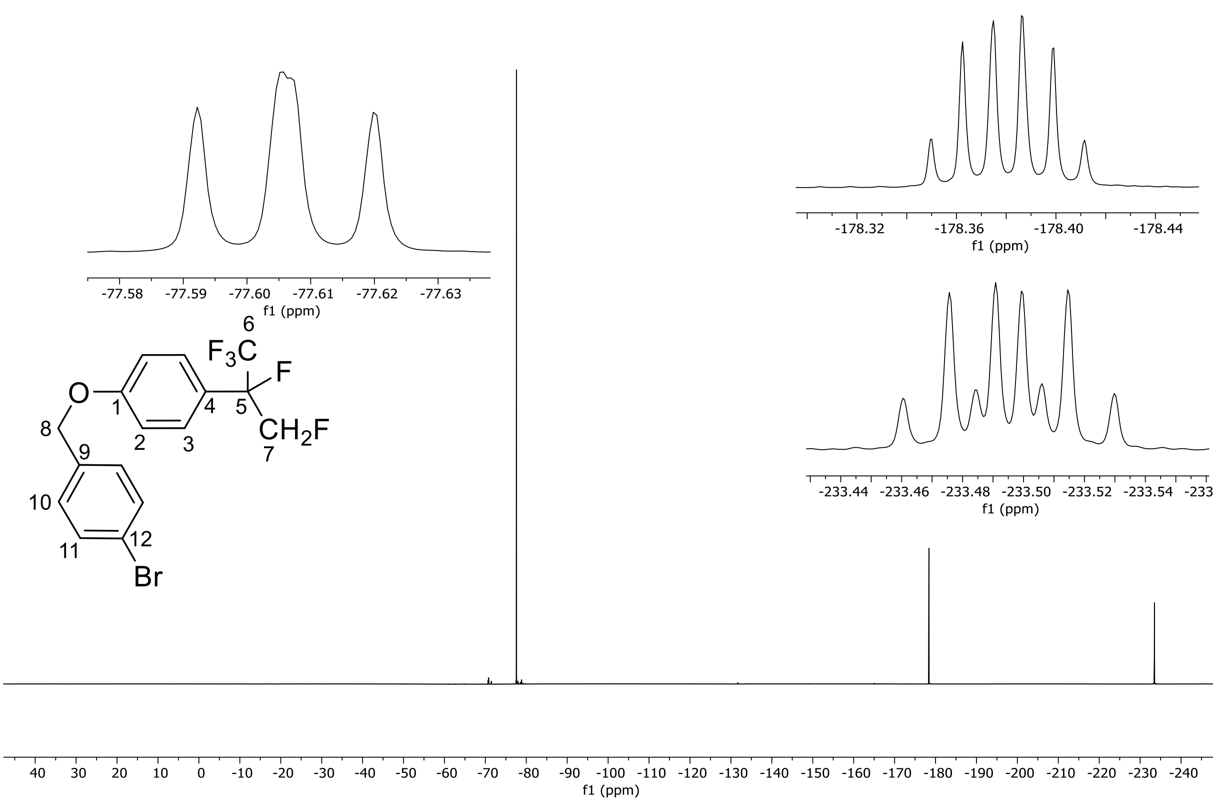

1-Methyl-4-(1,1,1,2,3-pentafluoropropan-2-yl)benzene (2e)

^{1}H NMR (599 MHz, $CDCl_3$)

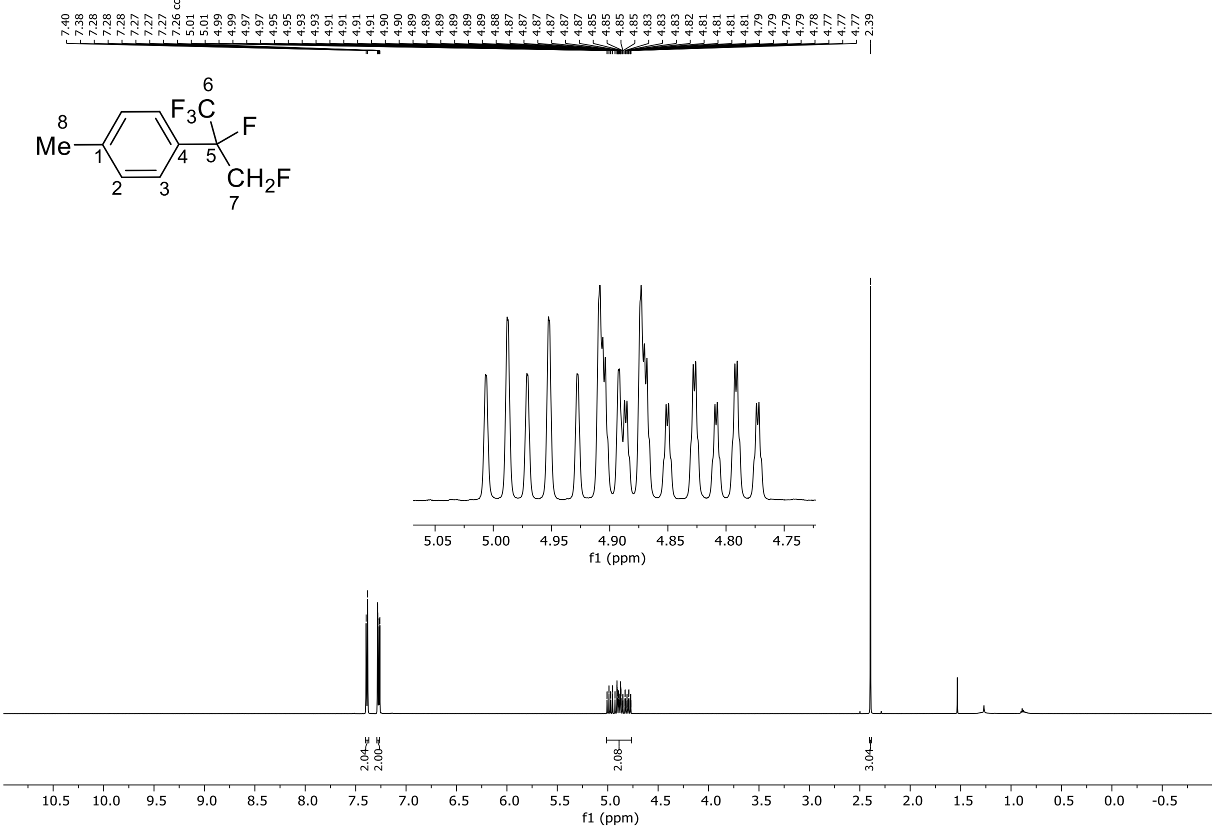

^{13}C NMR (151 MHz, $CDCl_3$)

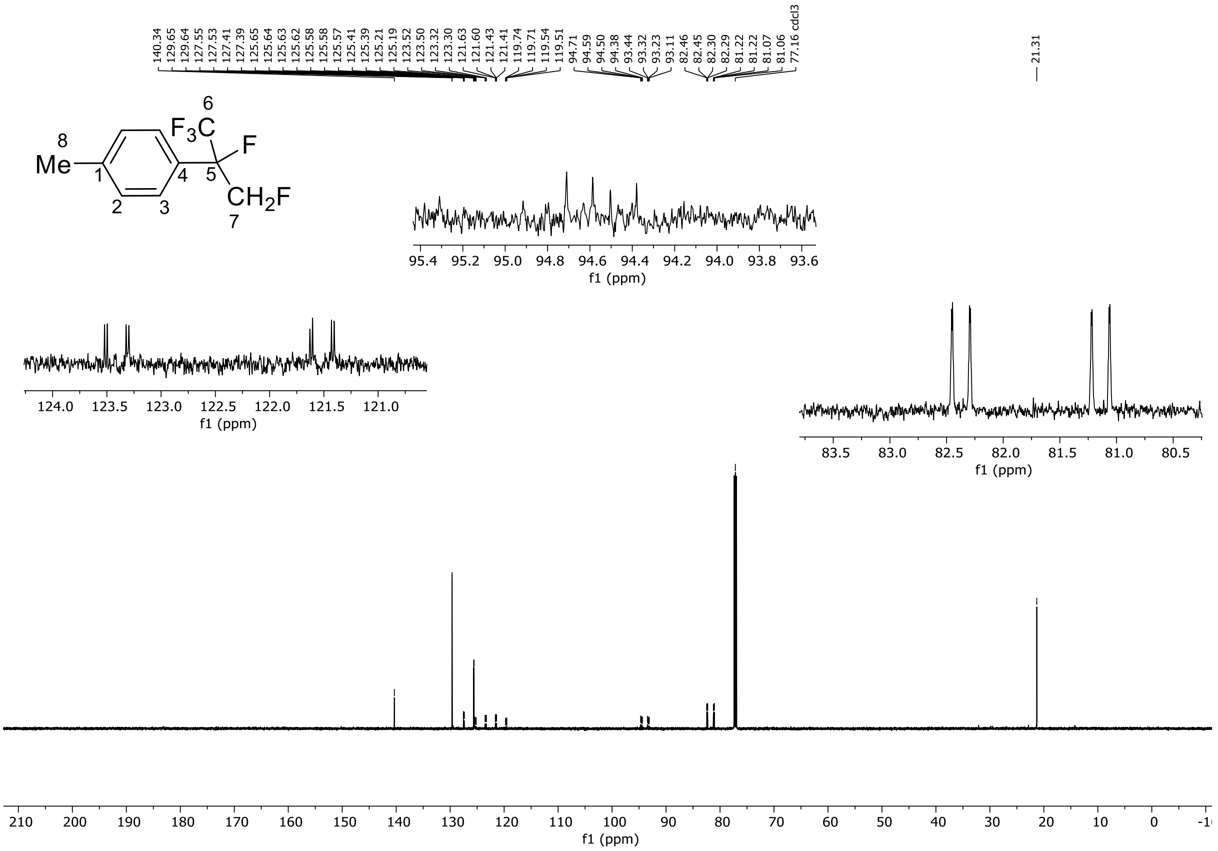

^{19}F NMR (564 MHz, $CDCl_3$)

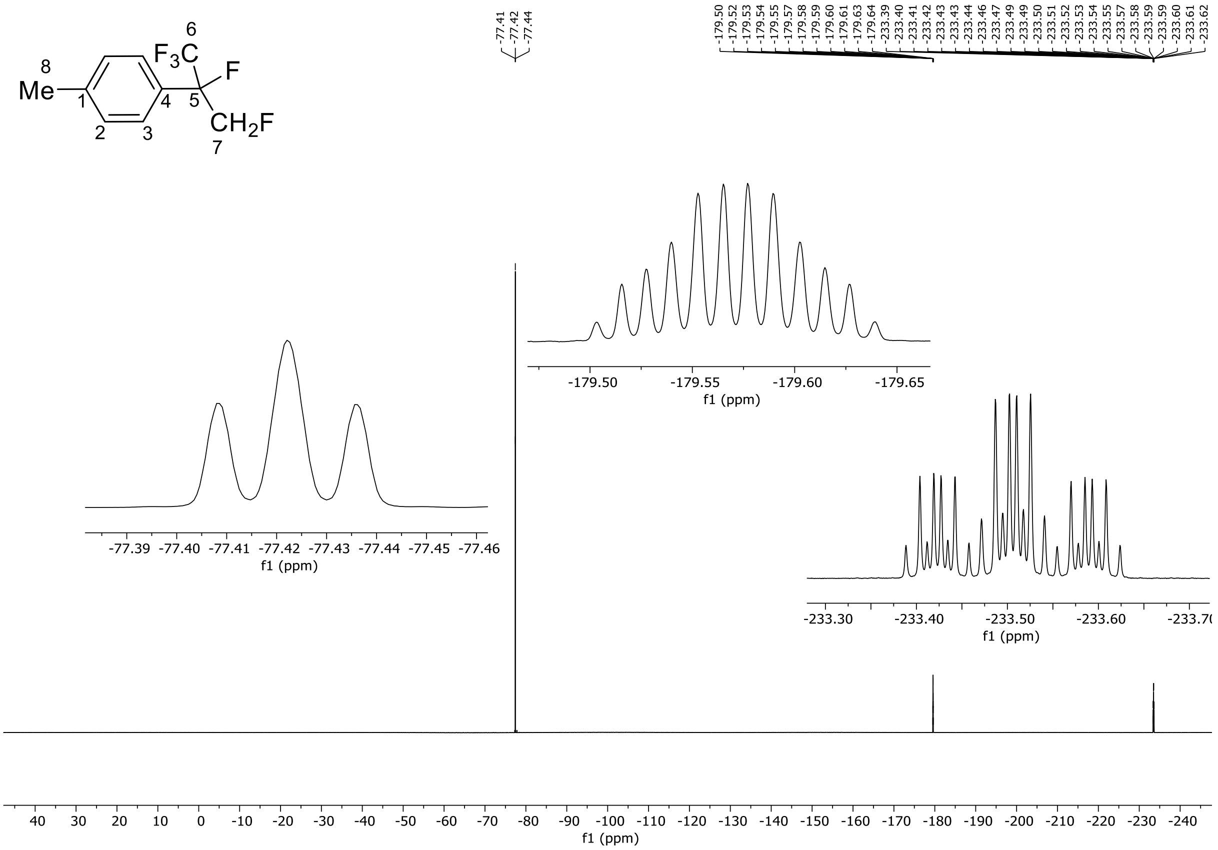

^{19}F{^{1}H} NMR (564 MHz, $CDCl_3$)

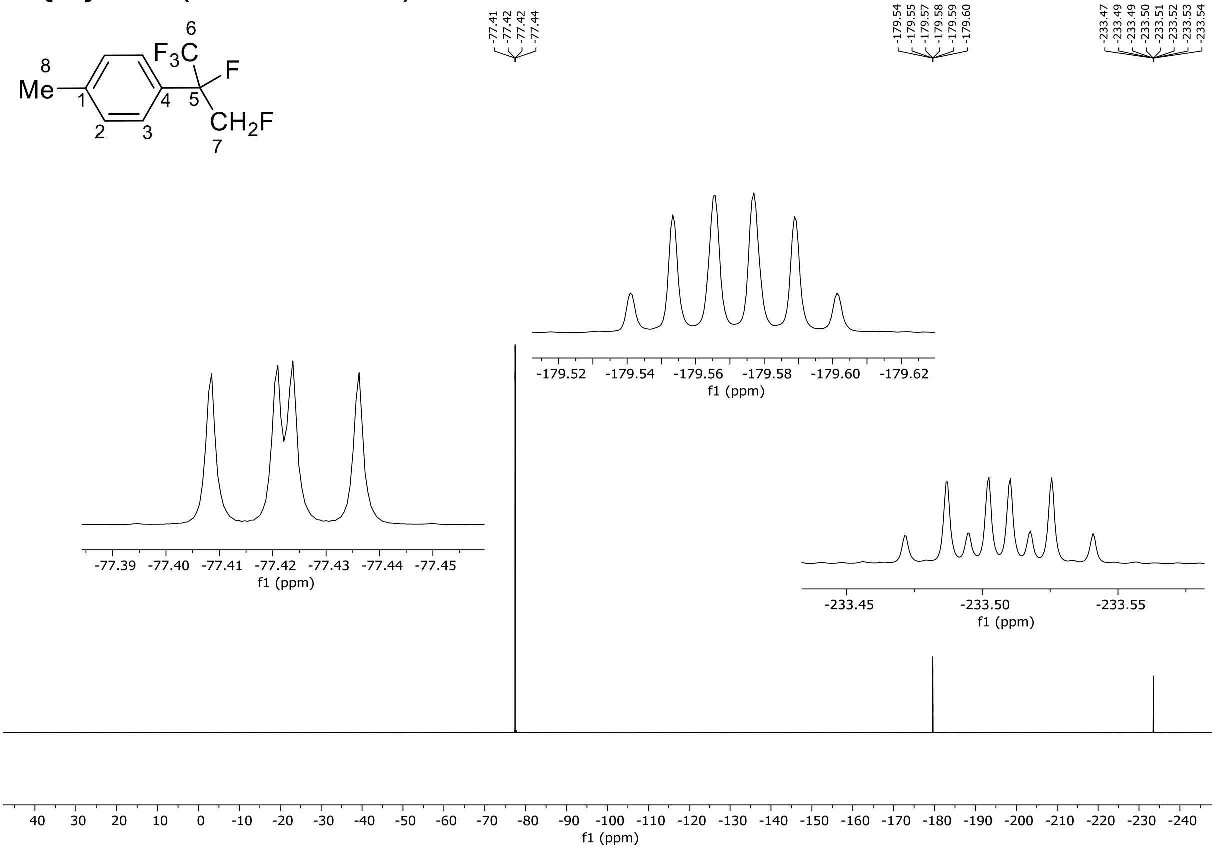

1-Isopropyl-4-(1,1,1,2,3-pentafluoropropan-2-yl)benzene (2f)

^{1}H NMR (500 MHz, $CDCl_3$)

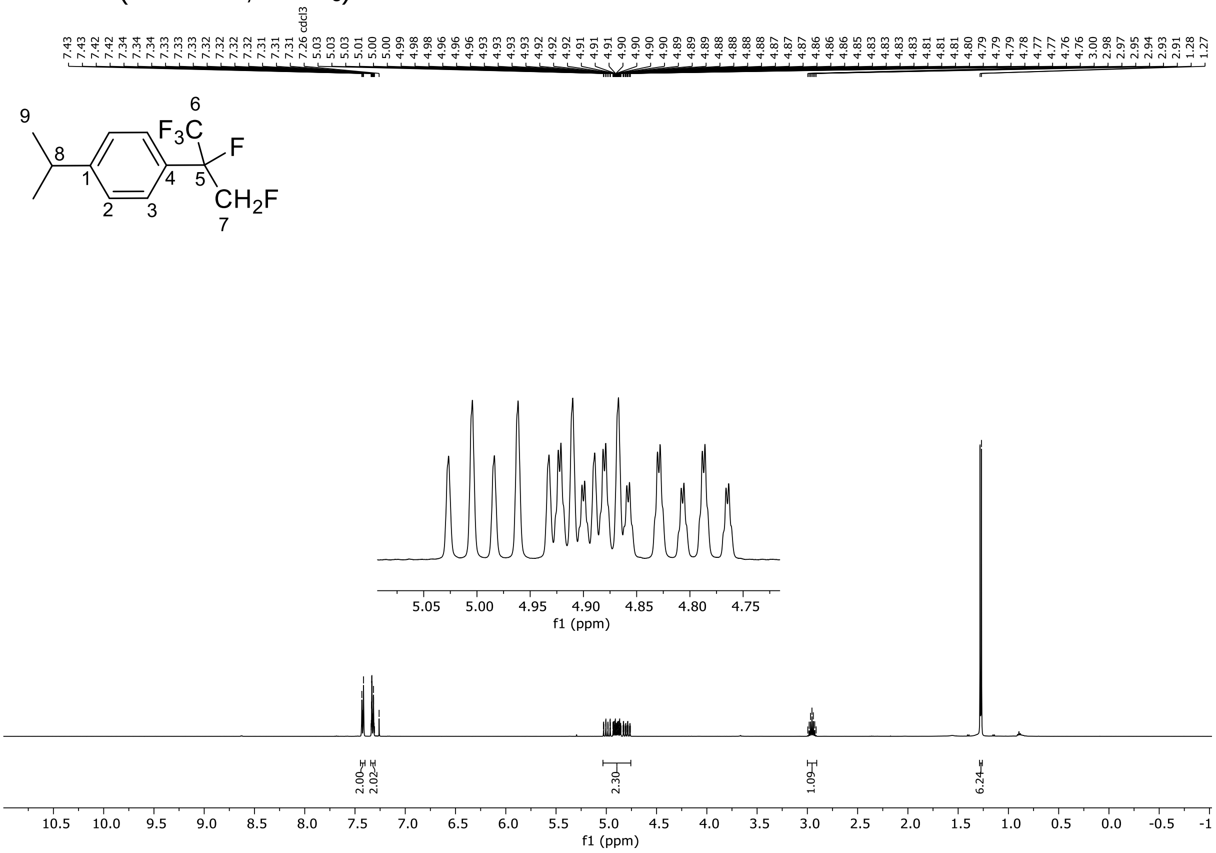

^{13}C NMR (126 MHz, $CDCl_3$)

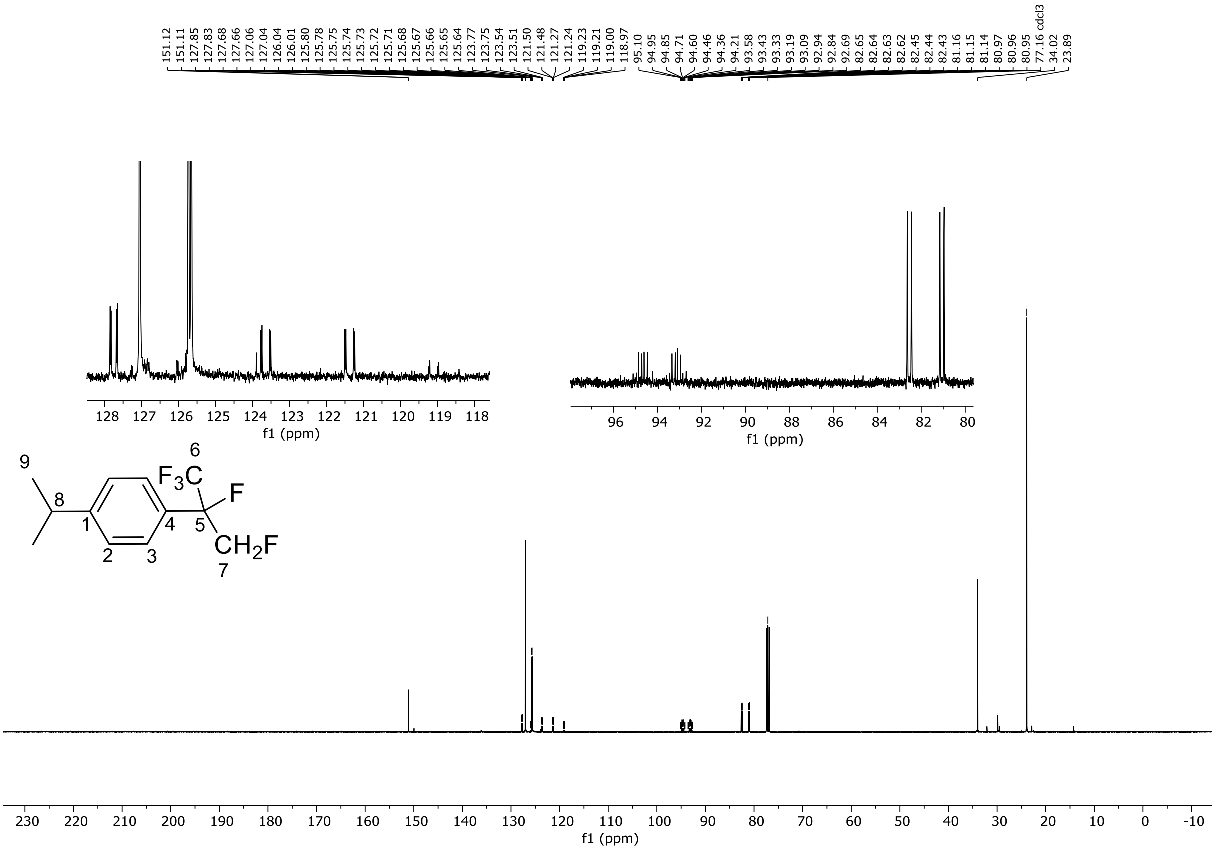

^{19}F NMR (126 MHz, $CDCl_3$)

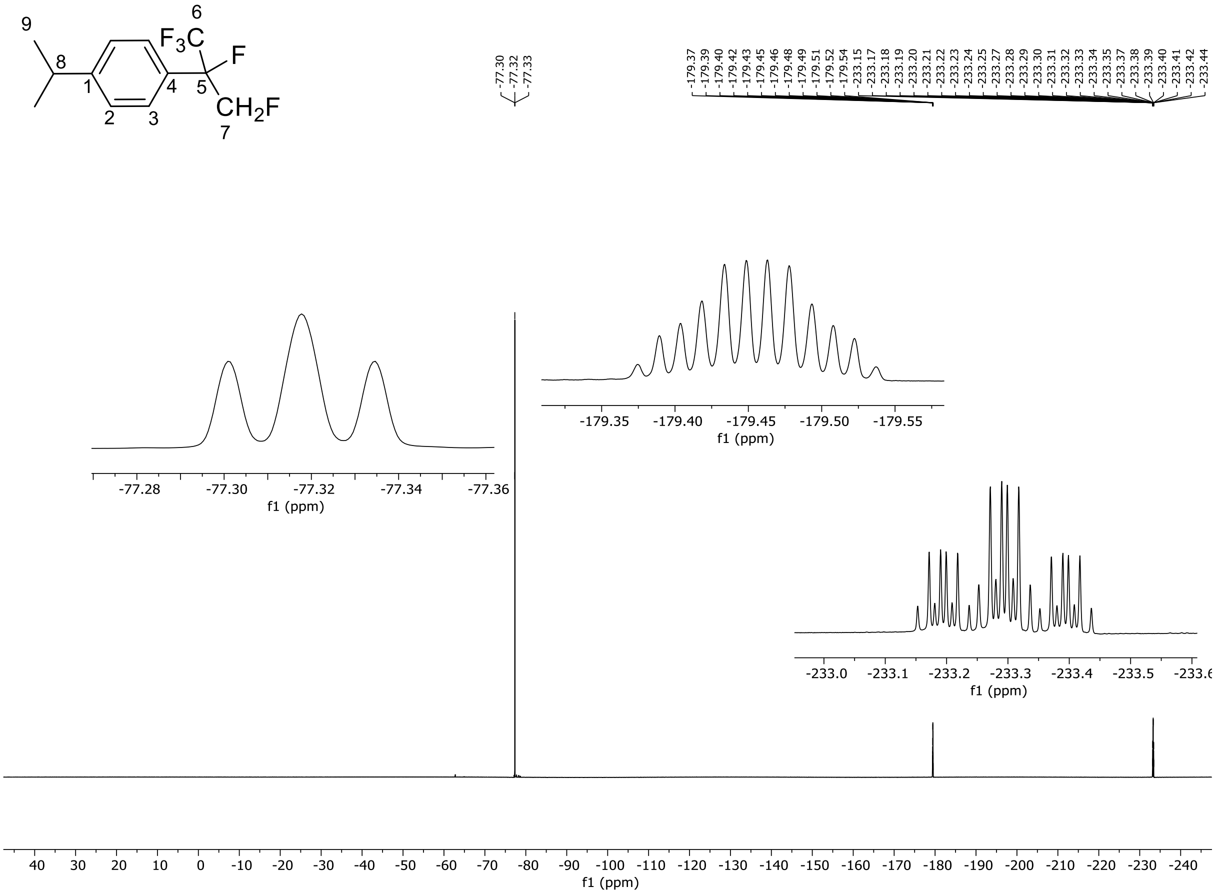

$^{19}F\{^{1}H\}$ NMR (126 MHz, $CDCl_3$)

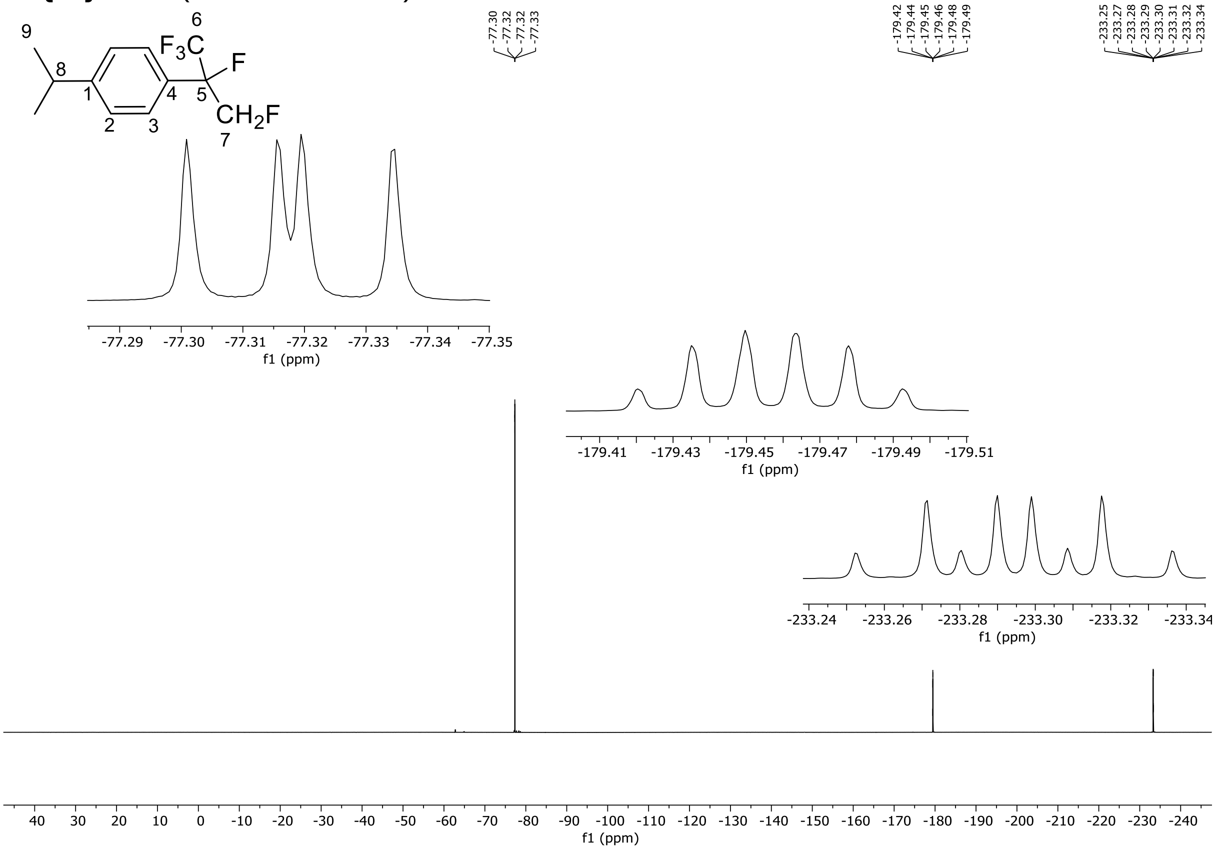

1-(Tert-butyl)-4-(1,1,1,2,3-pentafluoropropan-2-yl)benzene (2g)

^{1}H NMR (500 MHz, $CDCl_3$)

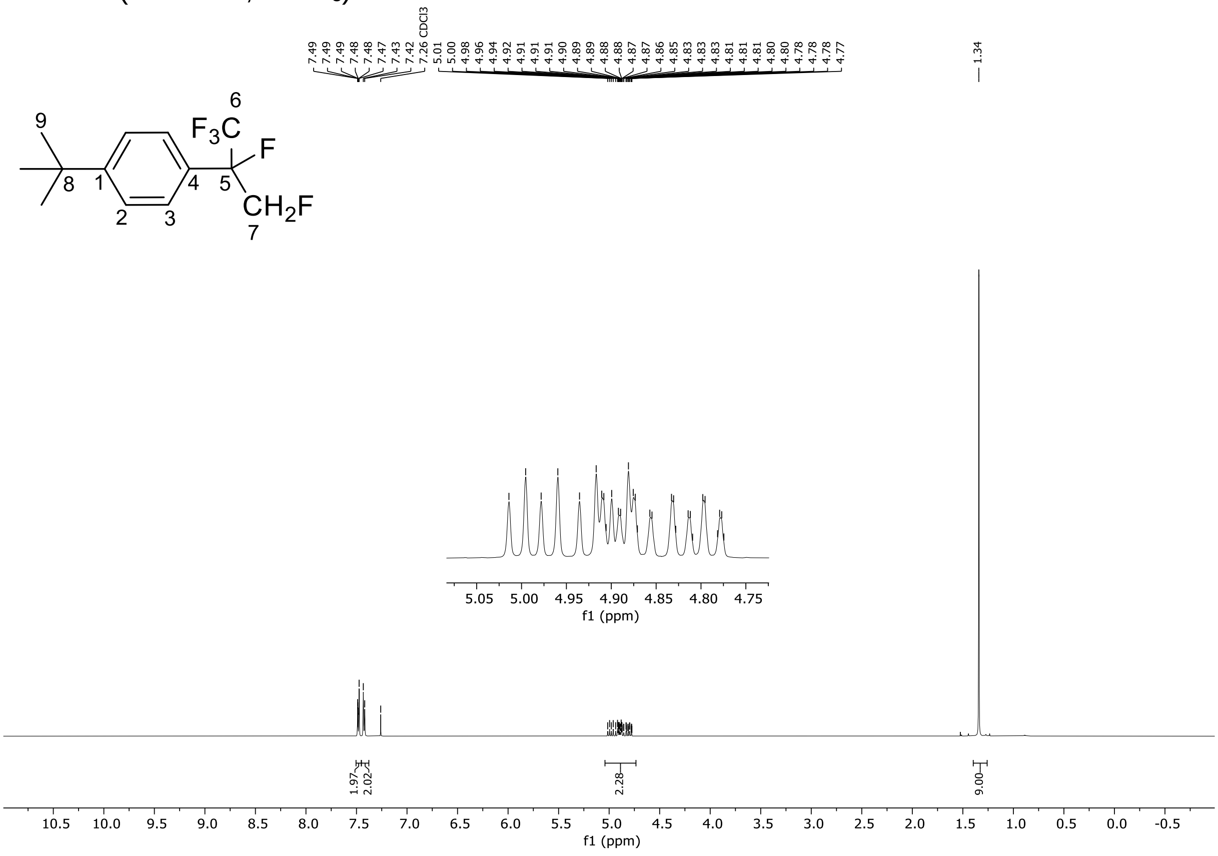

^{13}C NMR (126 MHz, $CDCl_3$)

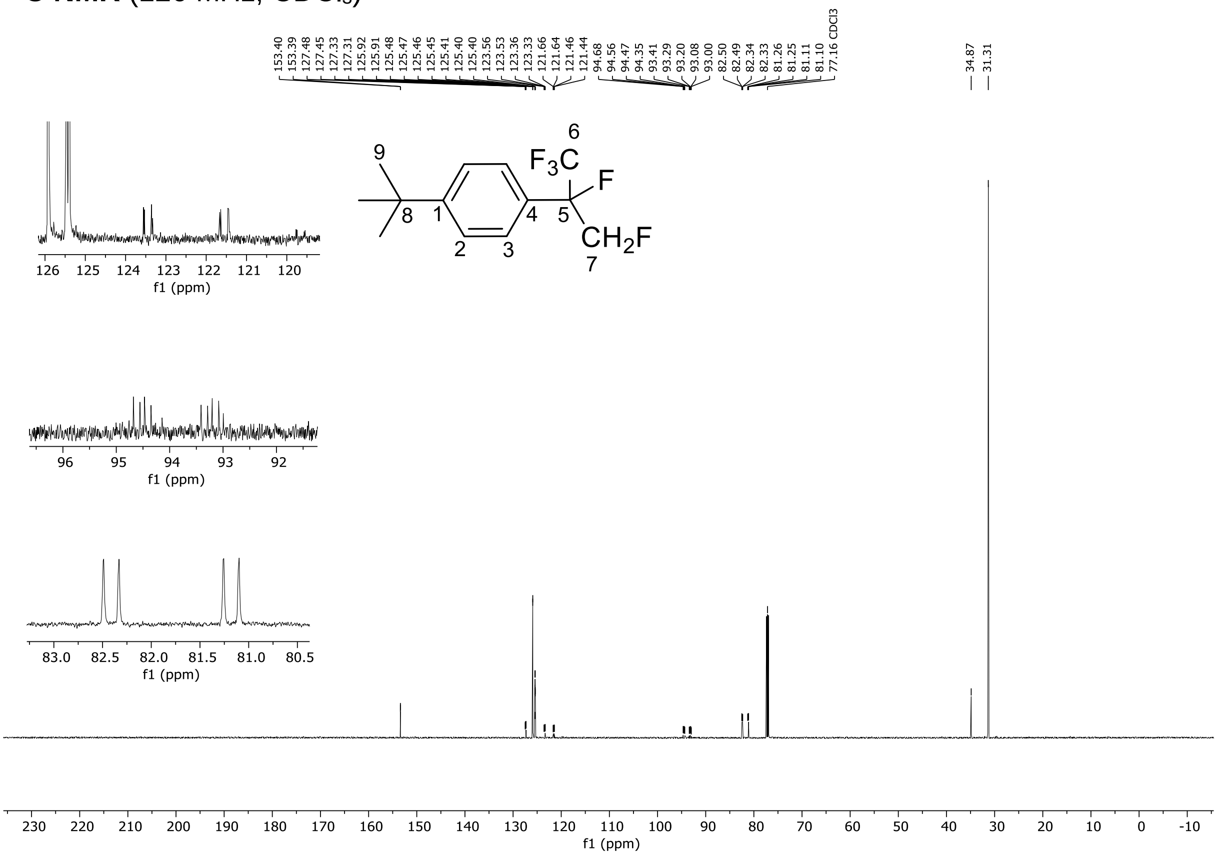

^{19}F NMR (376 MHz, $CDCl_3$)

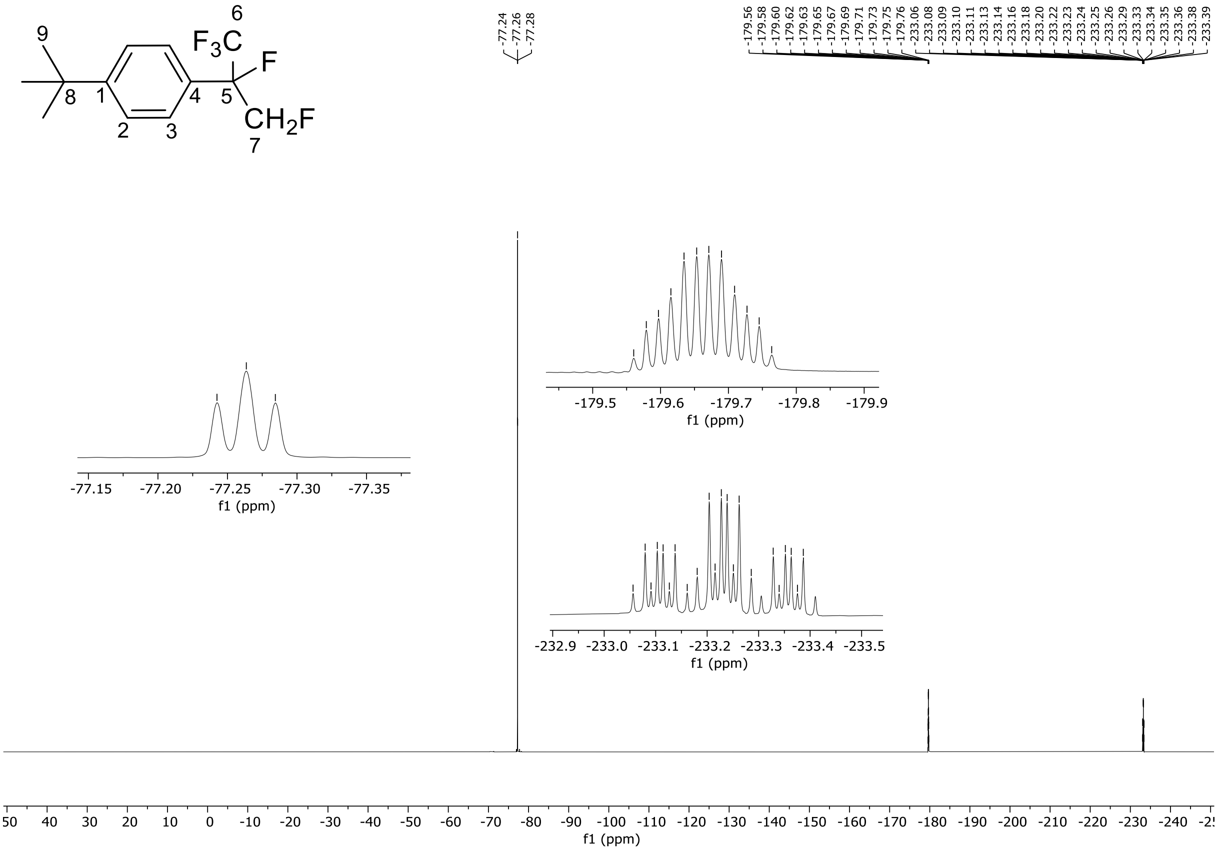

$^{19}F\{^1H\}$ NMR (126 MHz, $CDCl_3$)

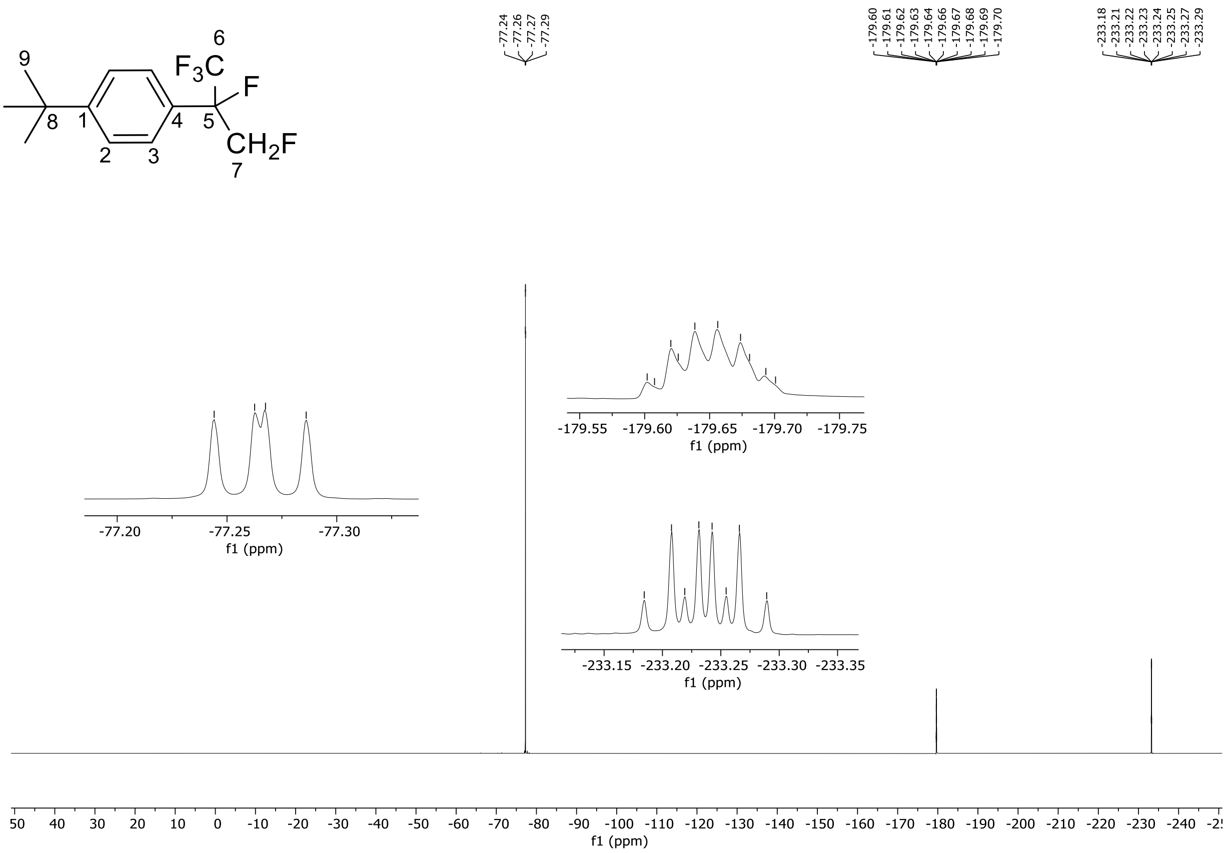

(1-(4-Cyclopropylphenyl)-1,2,2,2-tetrafluoroethyl)(λ^3-methyl)-λ^2-fluorane (2h)

^1H NMR (599 MHz, $CDCl_3$)

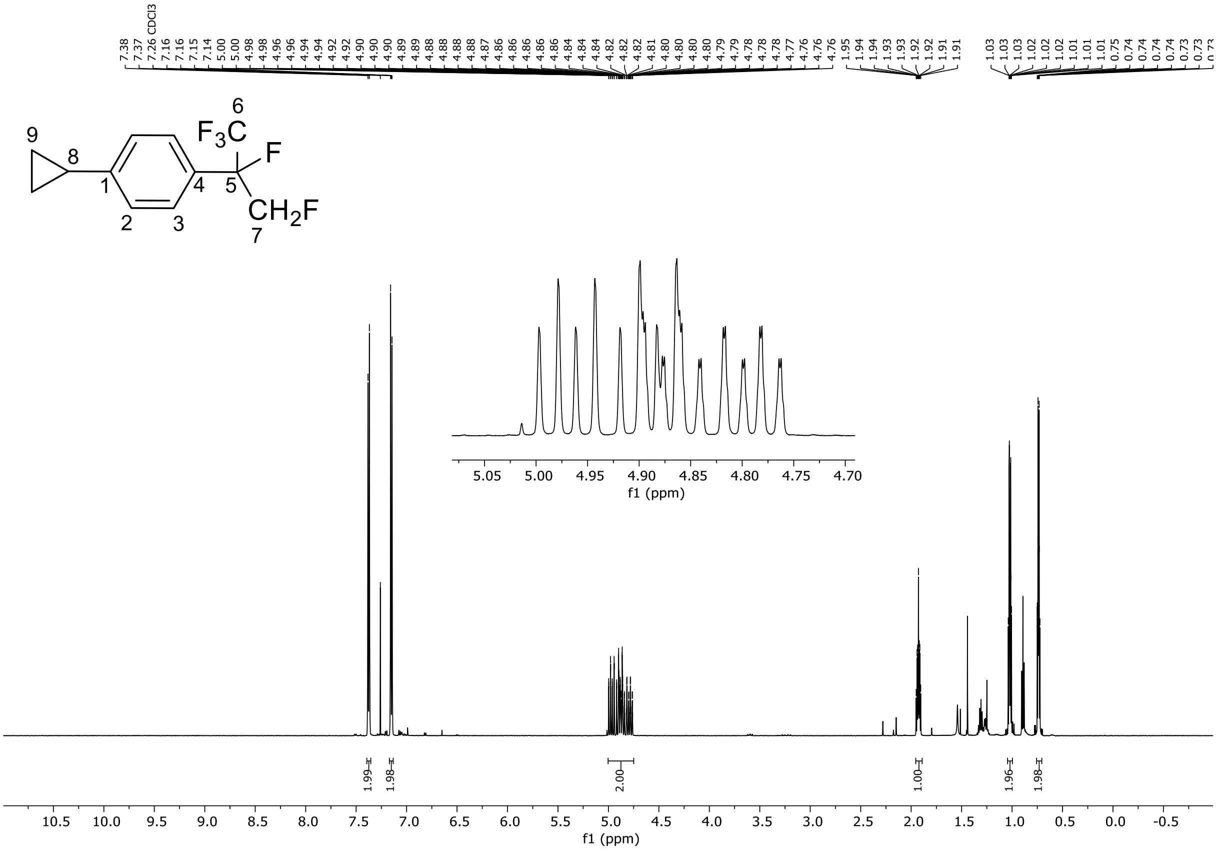

^{13}C NMR (151 MHz, $CDCl_3$)

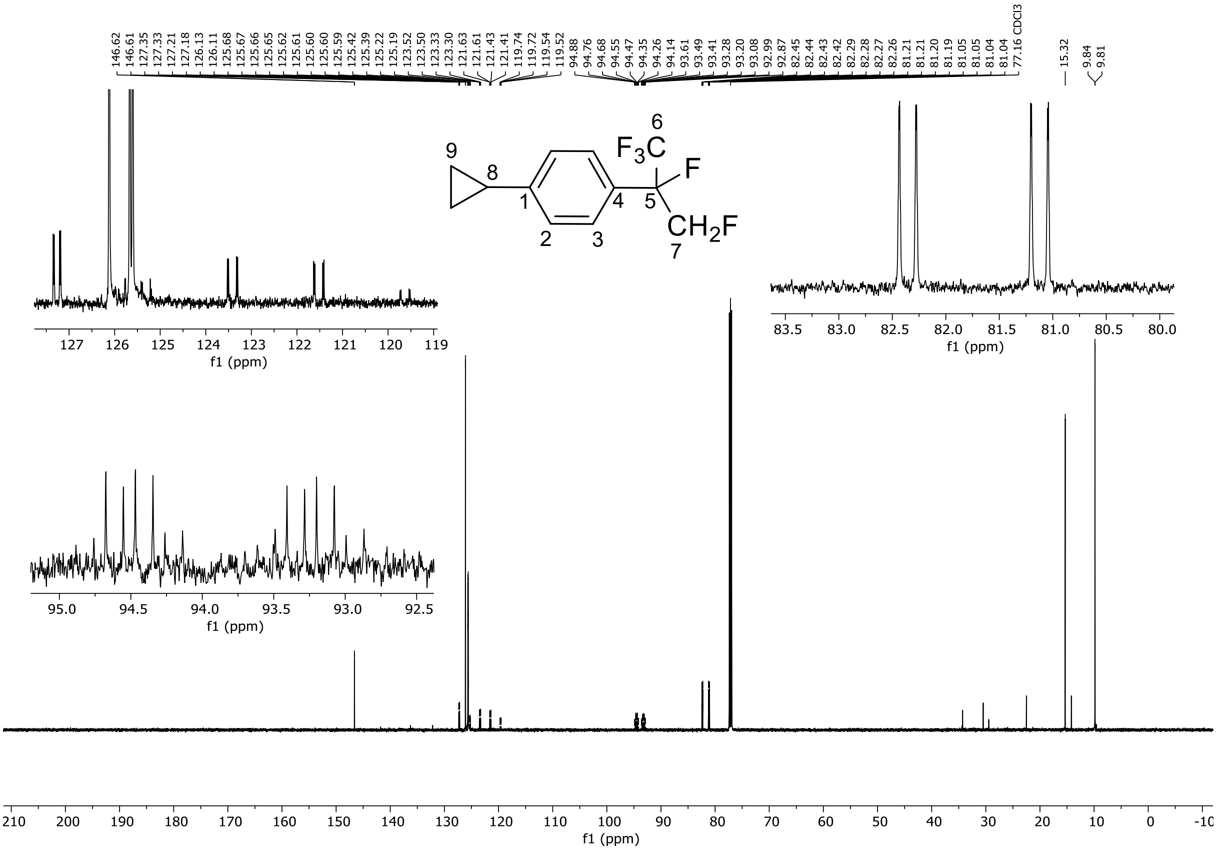

^{19}F NMR (564 MHz, $CDCl_3$)

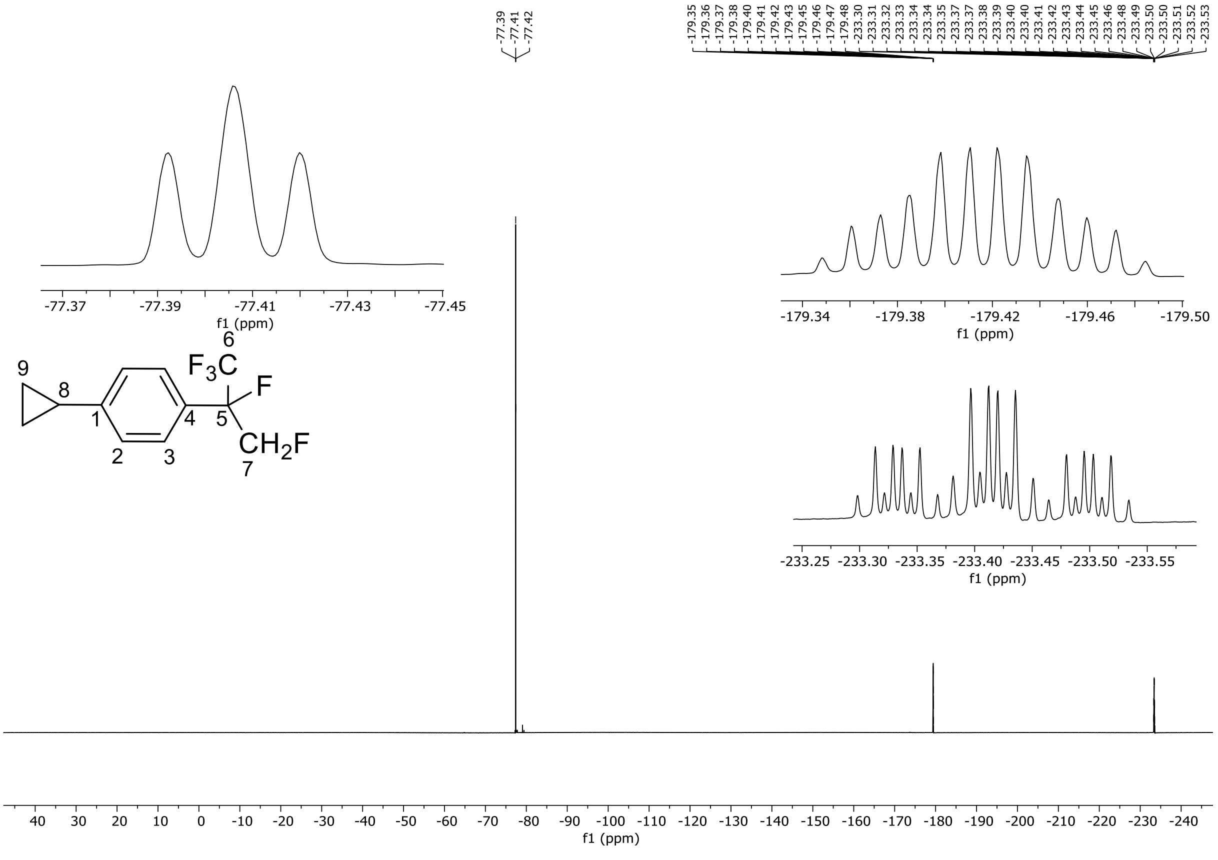

$^{19}F\{^1H\}$ NMR (564 MHz, $CDCl_3$)

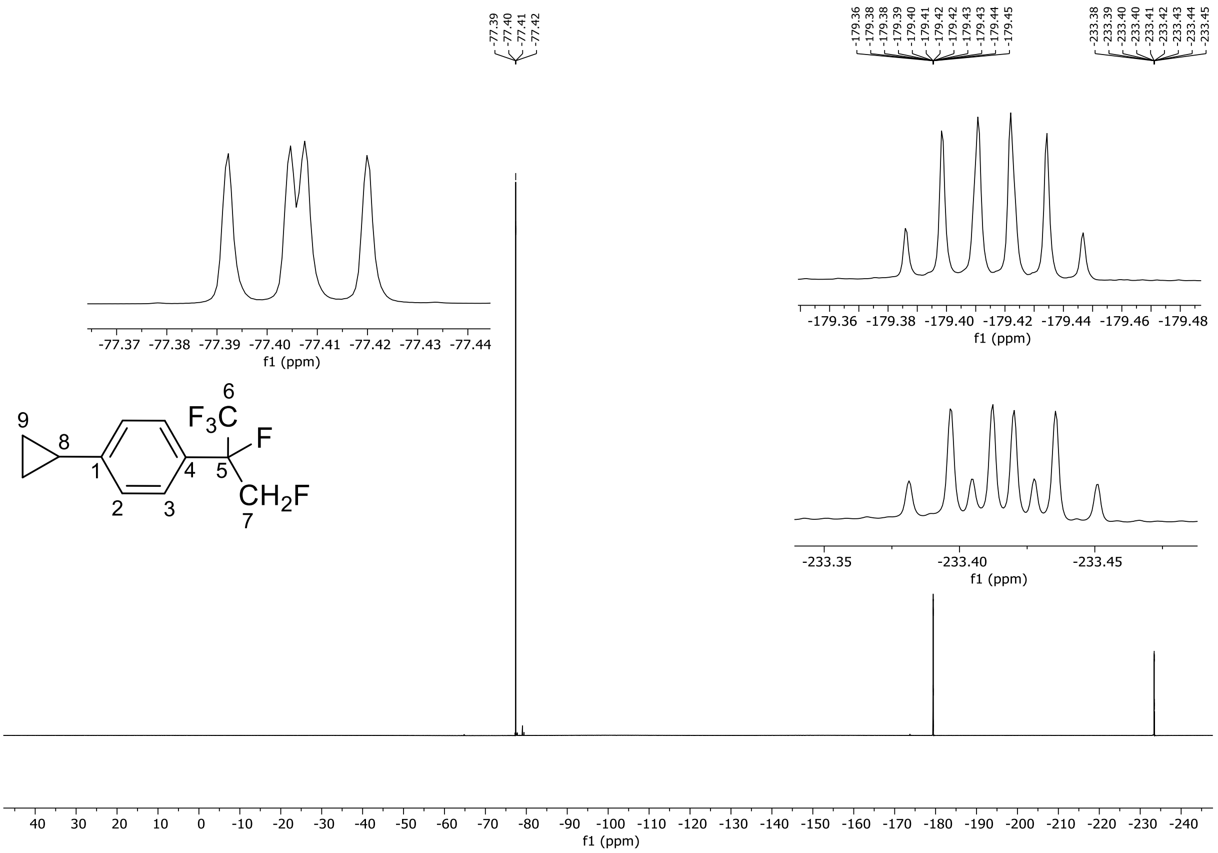

N,4-Dimethyl-N-(4-(1,1,1,2,3-pentafluoropropan-2-yl)phenyl)benzenesulfonamide (2i)

^1H NMR (500 MHz, $CDCl_3$)

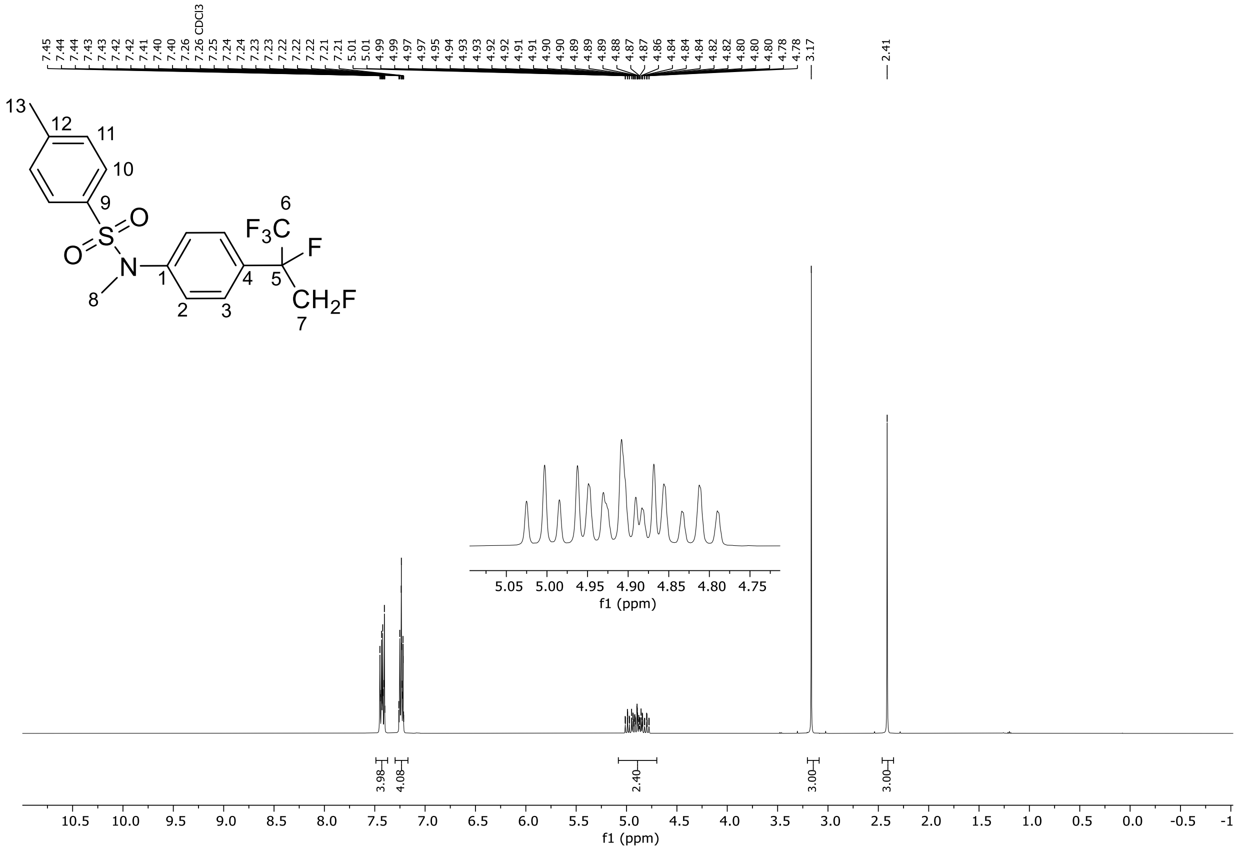

^{13}C NMR (126 MHz, $CDCl_3$)

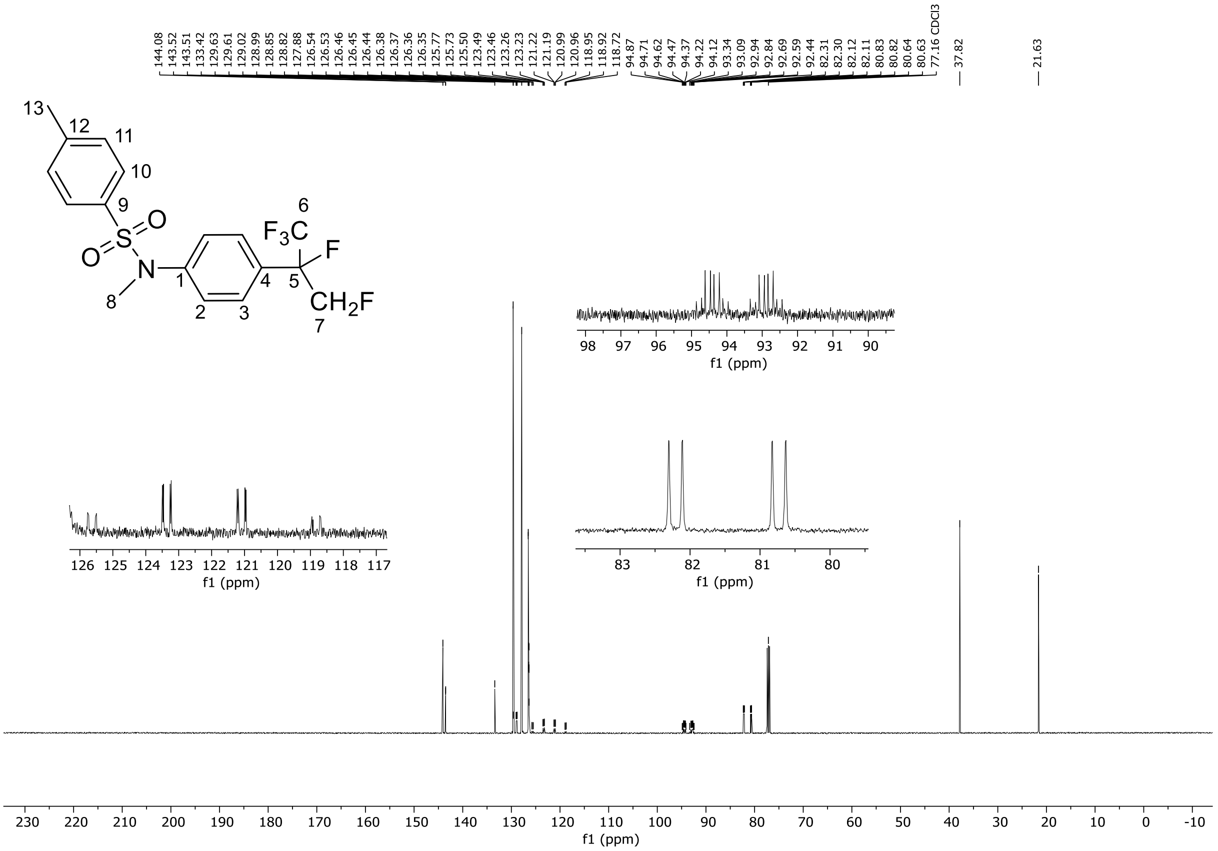

^{19}F NMR (470 MHz, $CDCl_3$)

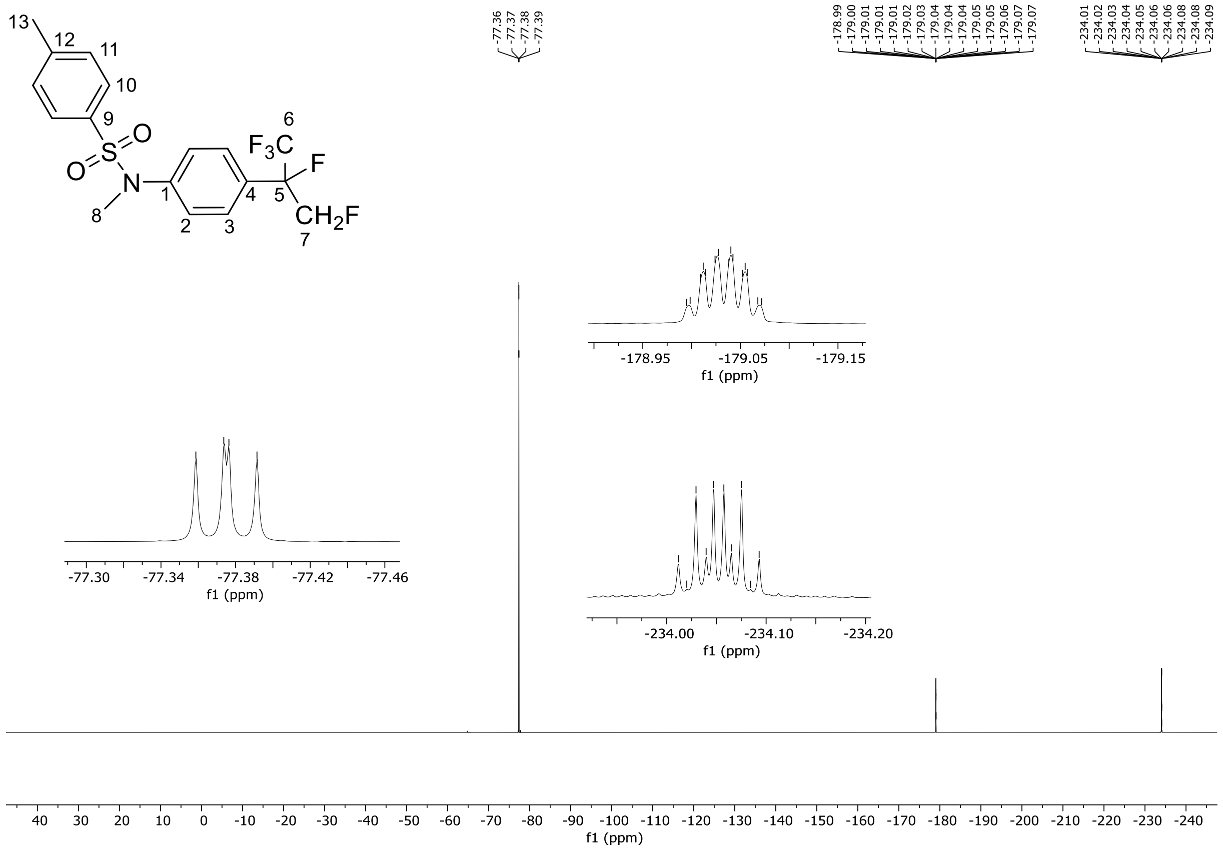

$^{19}F\{^{1}H\}$ NMR (470 MHz, $CDCl_3$)

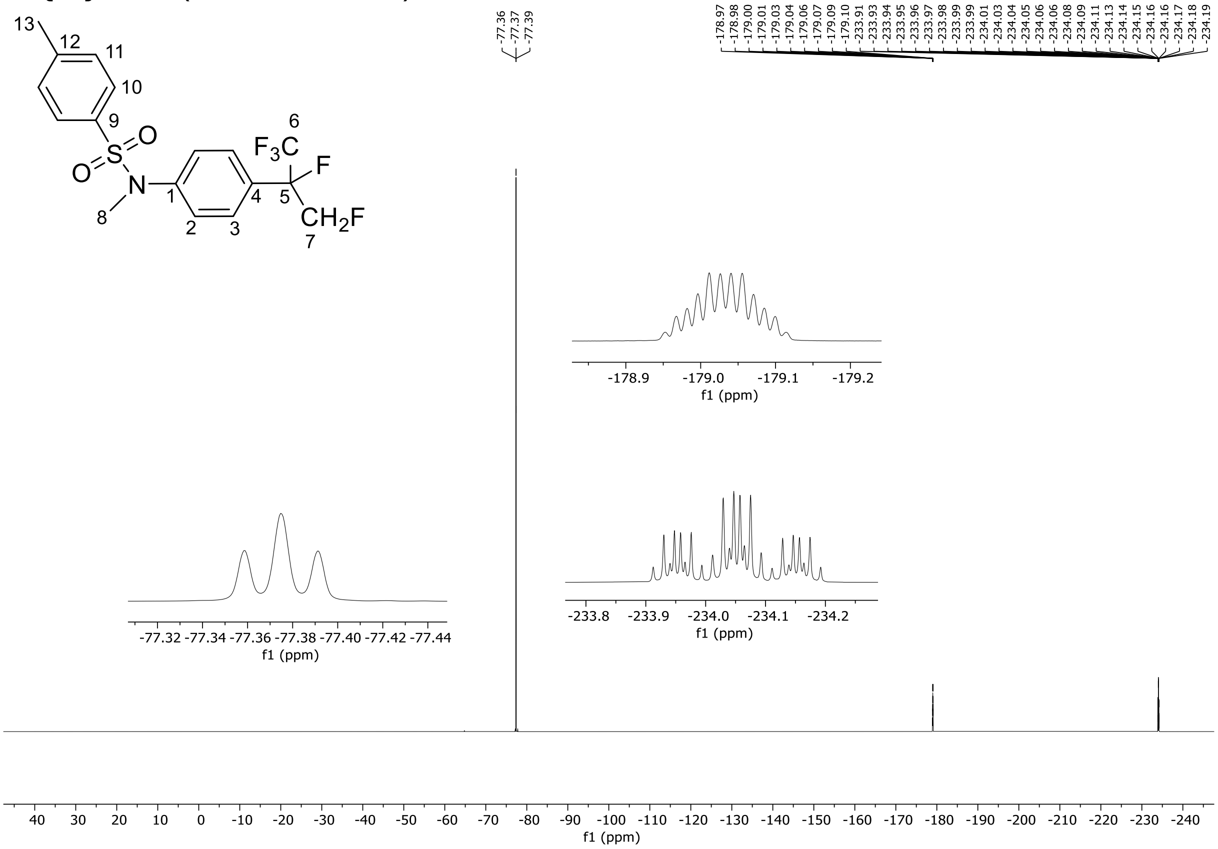

4-Fluoro-4'-(1,1,1,2,3-pentafluoropropan-2-yl)-1,1'-biphenyl (2j)

^{1}H NMR (500 MHz, $CDCl_3$)

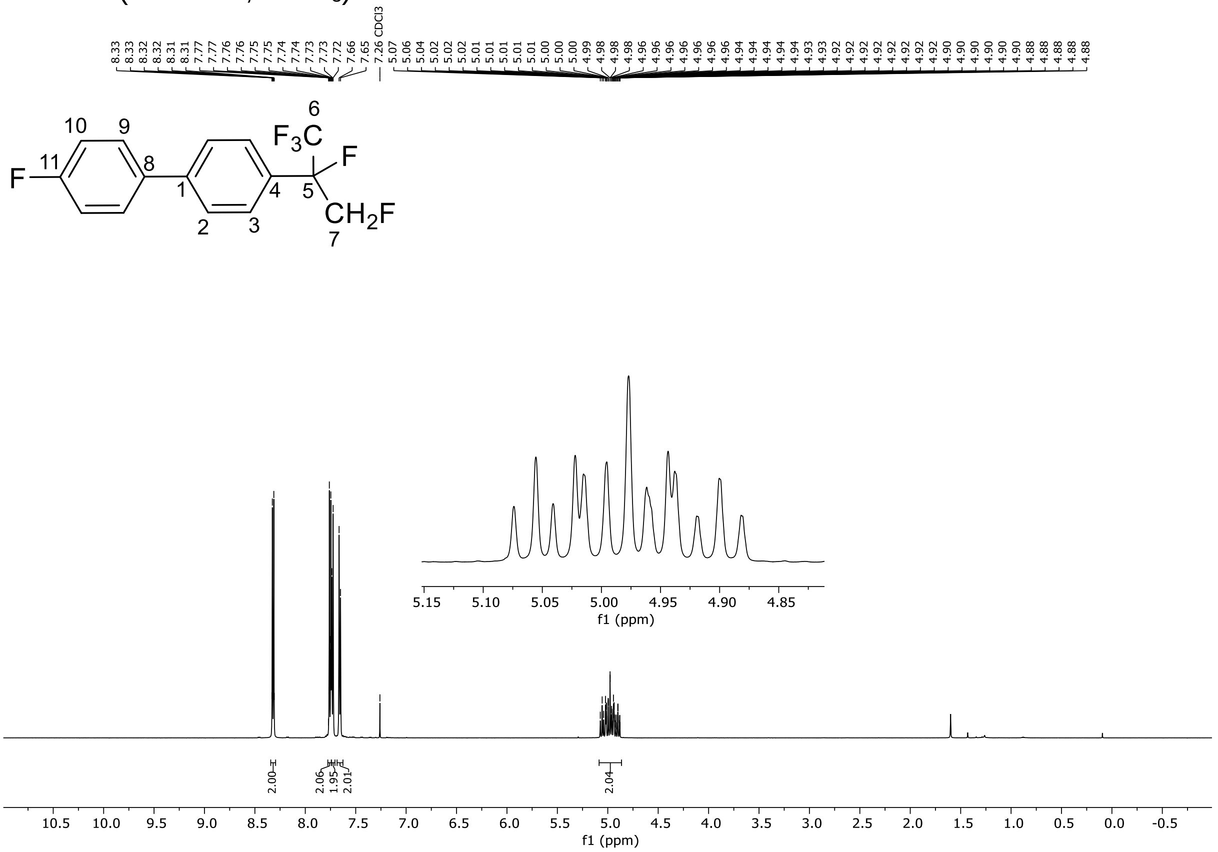

^{13}C NMR (126 MHz, $CDCl_3$)

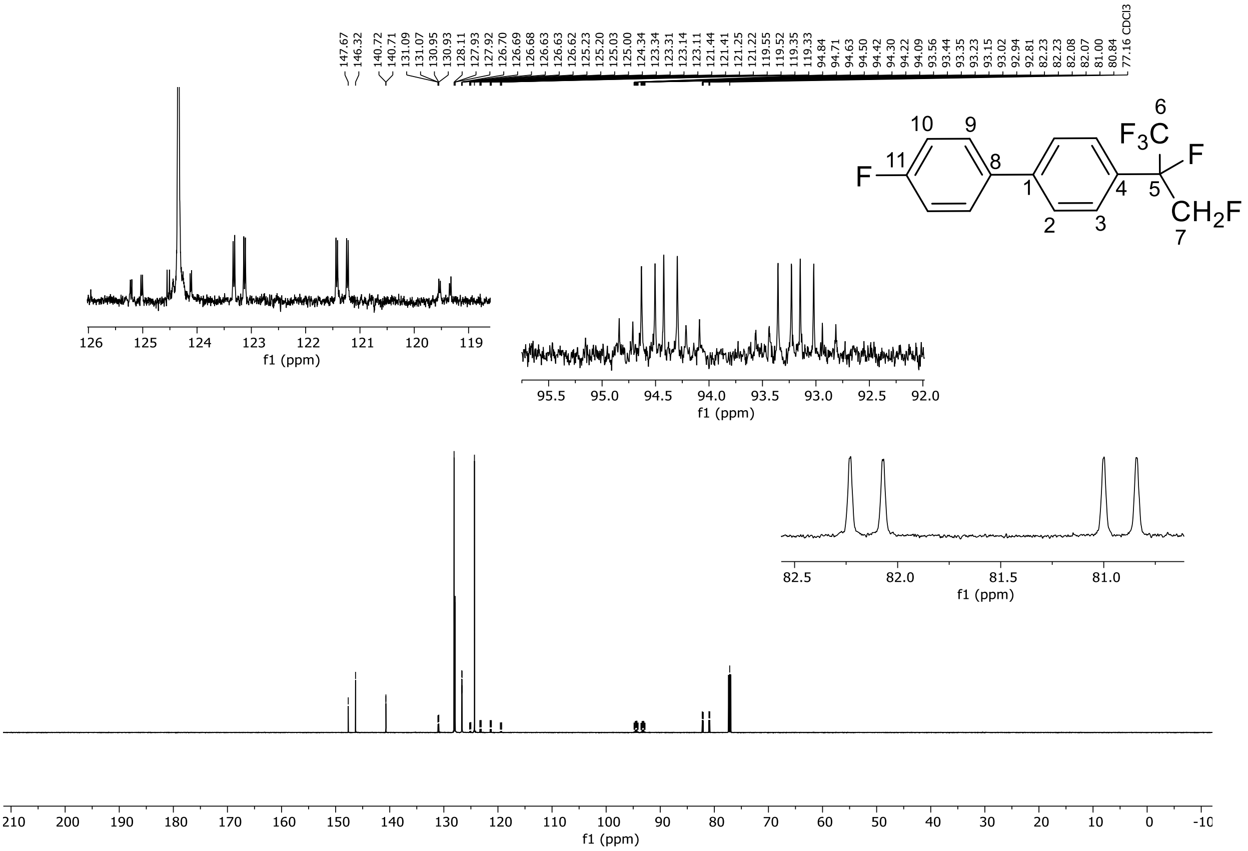

^{19}F NMR (126 MHz, $CDCl_3$)

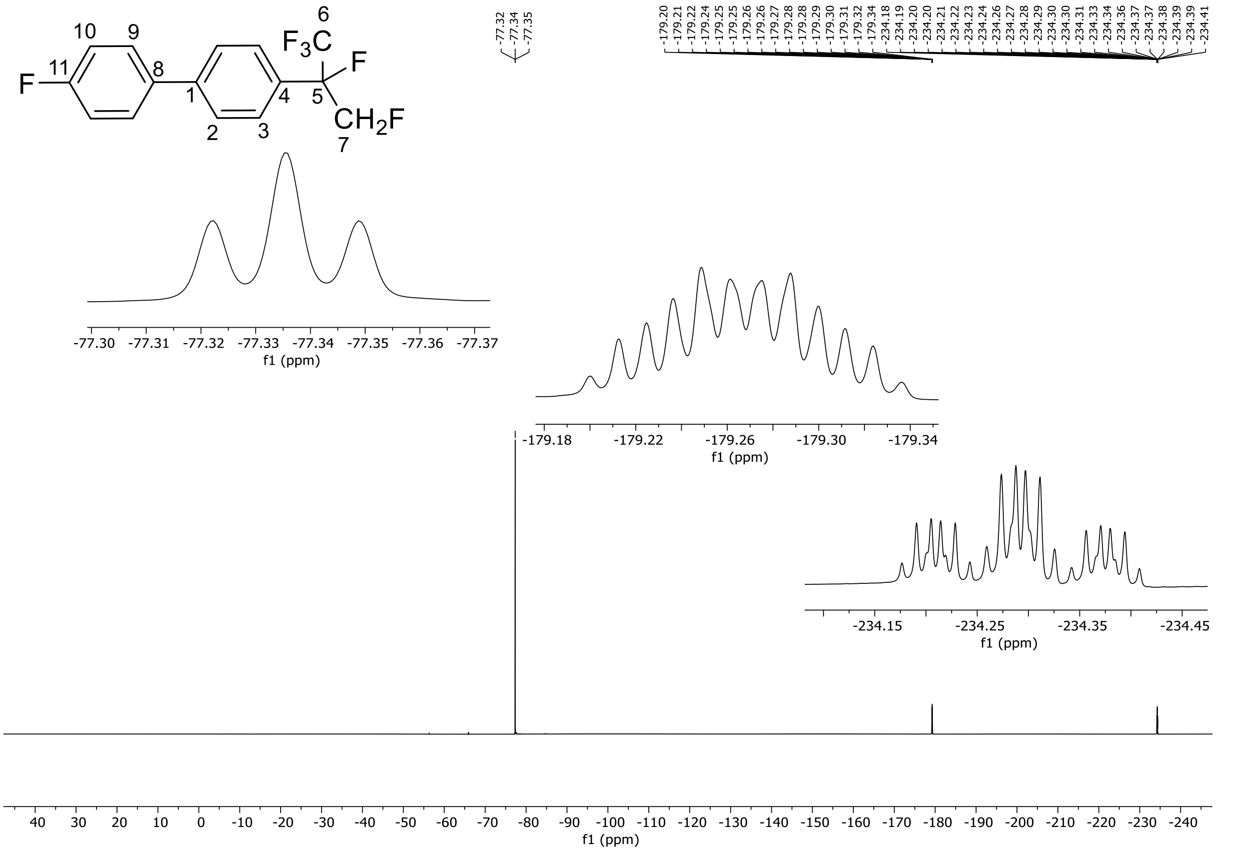

$^{19}F\{^{1}H\}$ NMR (126 MHz, $CDCl_3$)

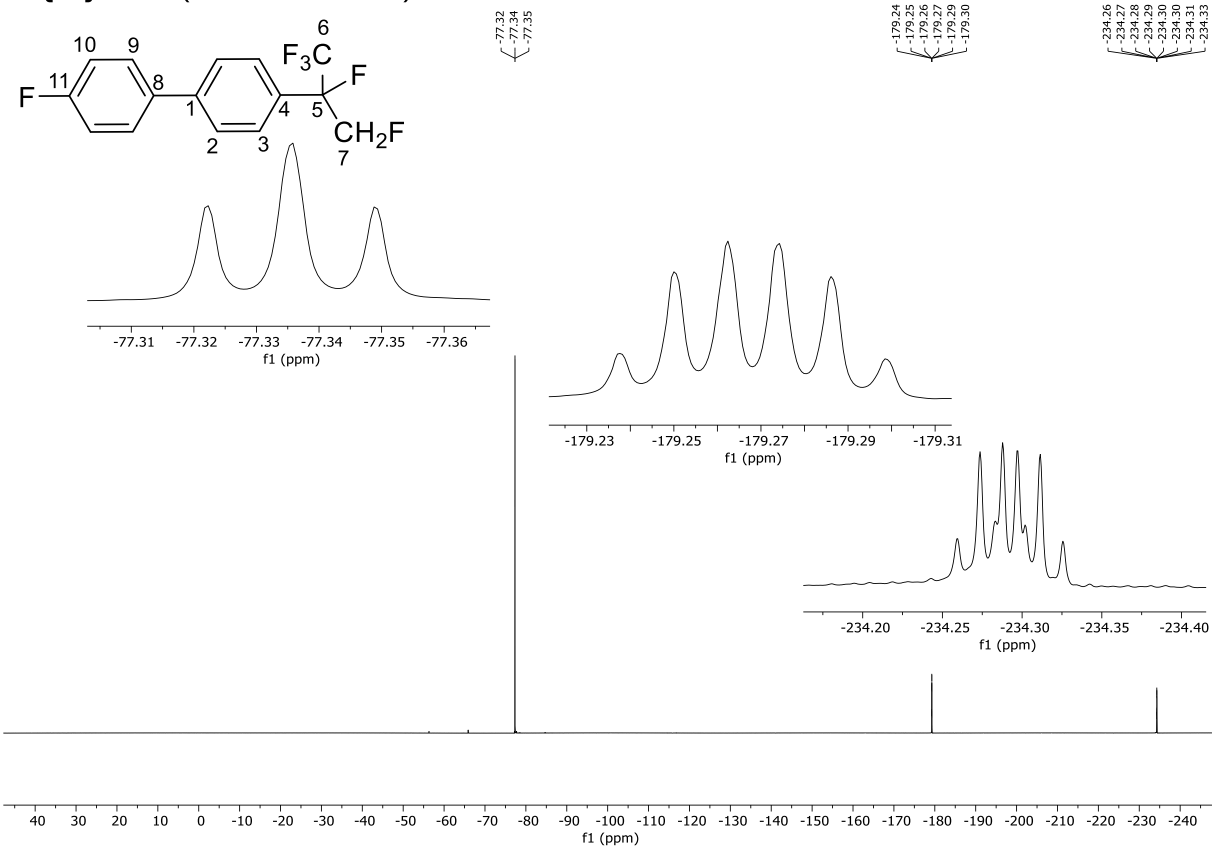

2-Methoxy-5-(1,1,1,2,3-pentafluoropropan-2-yl)benzonitrile (2k)
^{1}H NMR (599 MHz, $CDCl_3$)

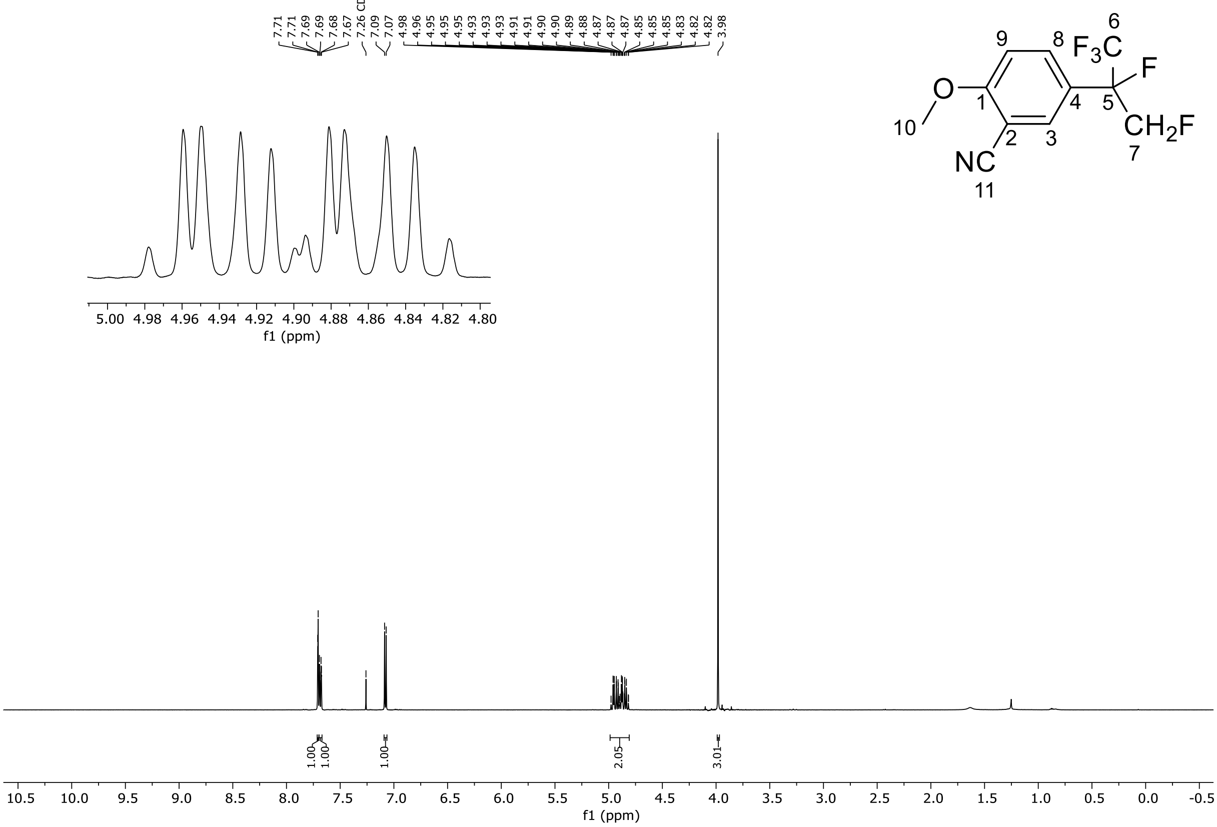

^{13}C NMR (151 MHz, $CDCl_3$)

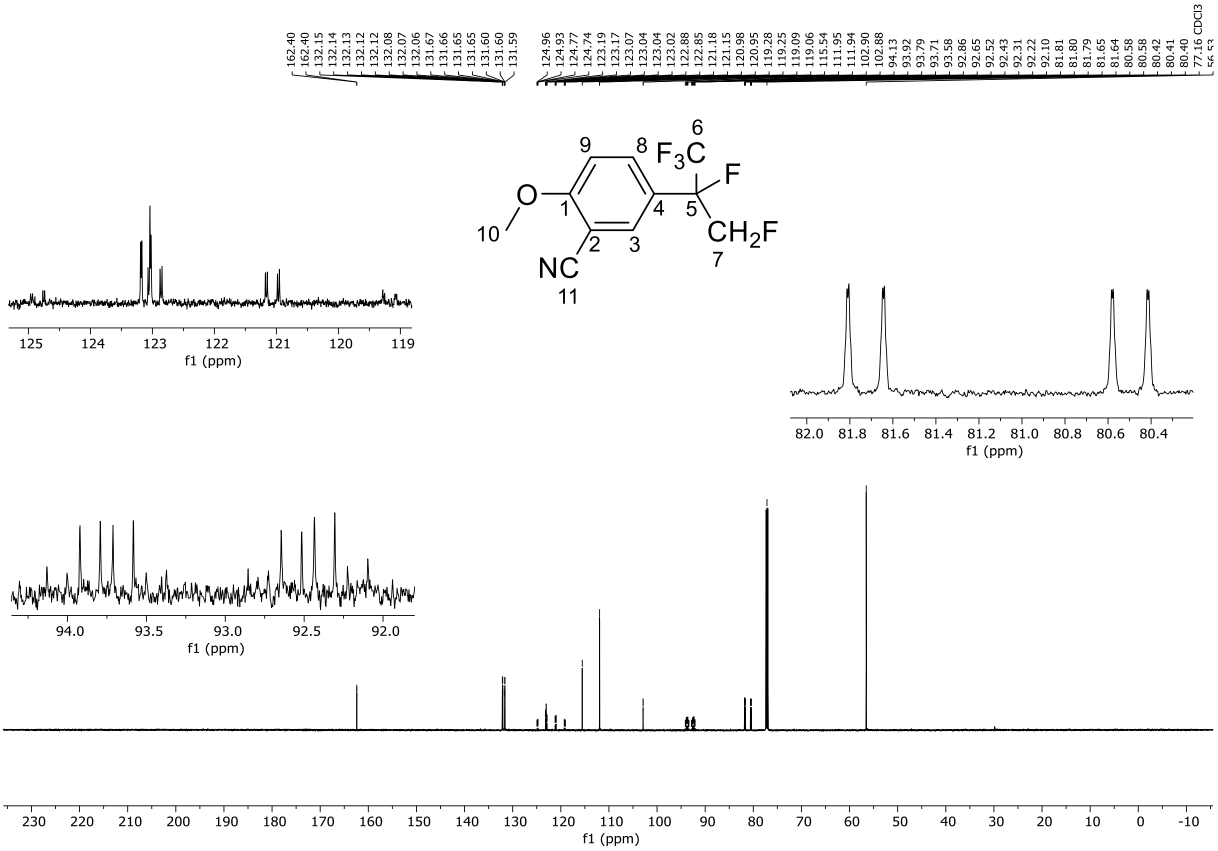

^{19}F NMR (564 MHz, $CDCl_3$)

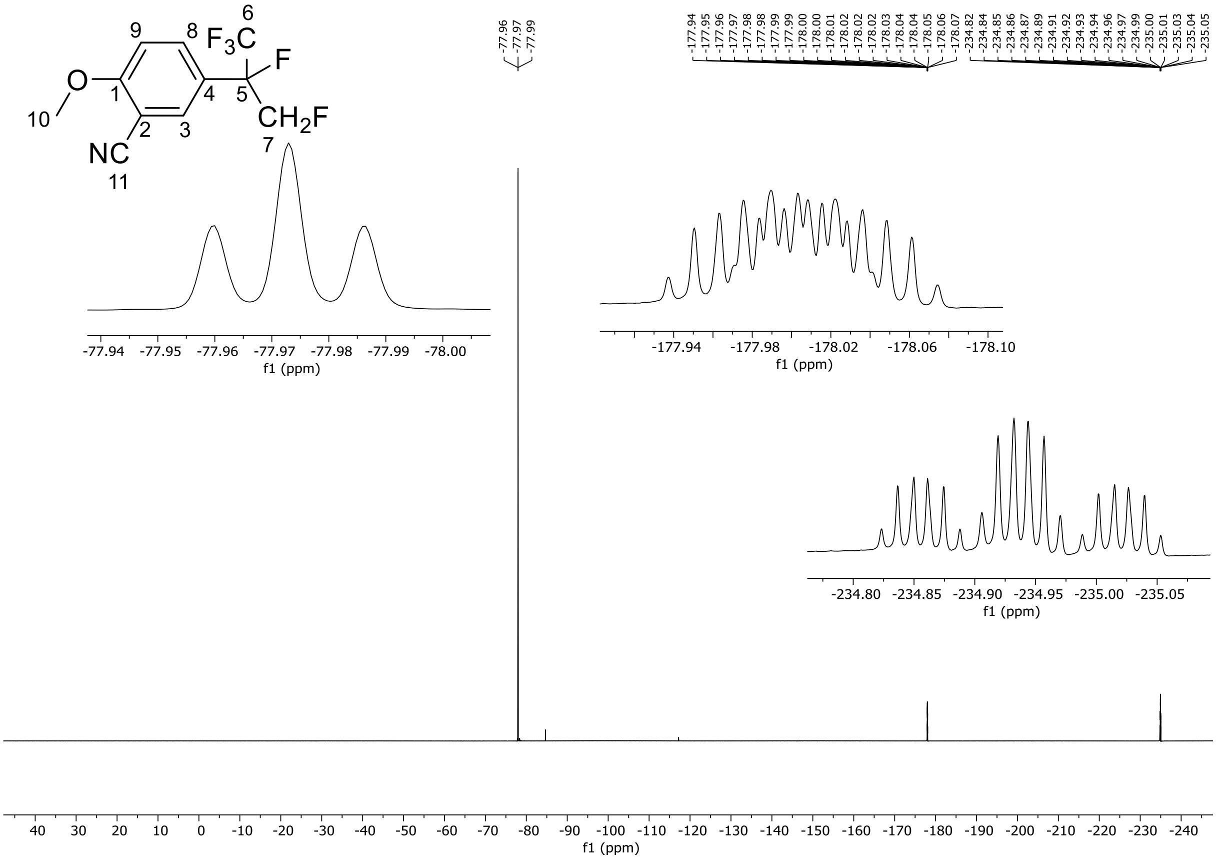

$^{19}F\{^{1}H\}$ **NMR** (564 MHz, $CDCl_3$)

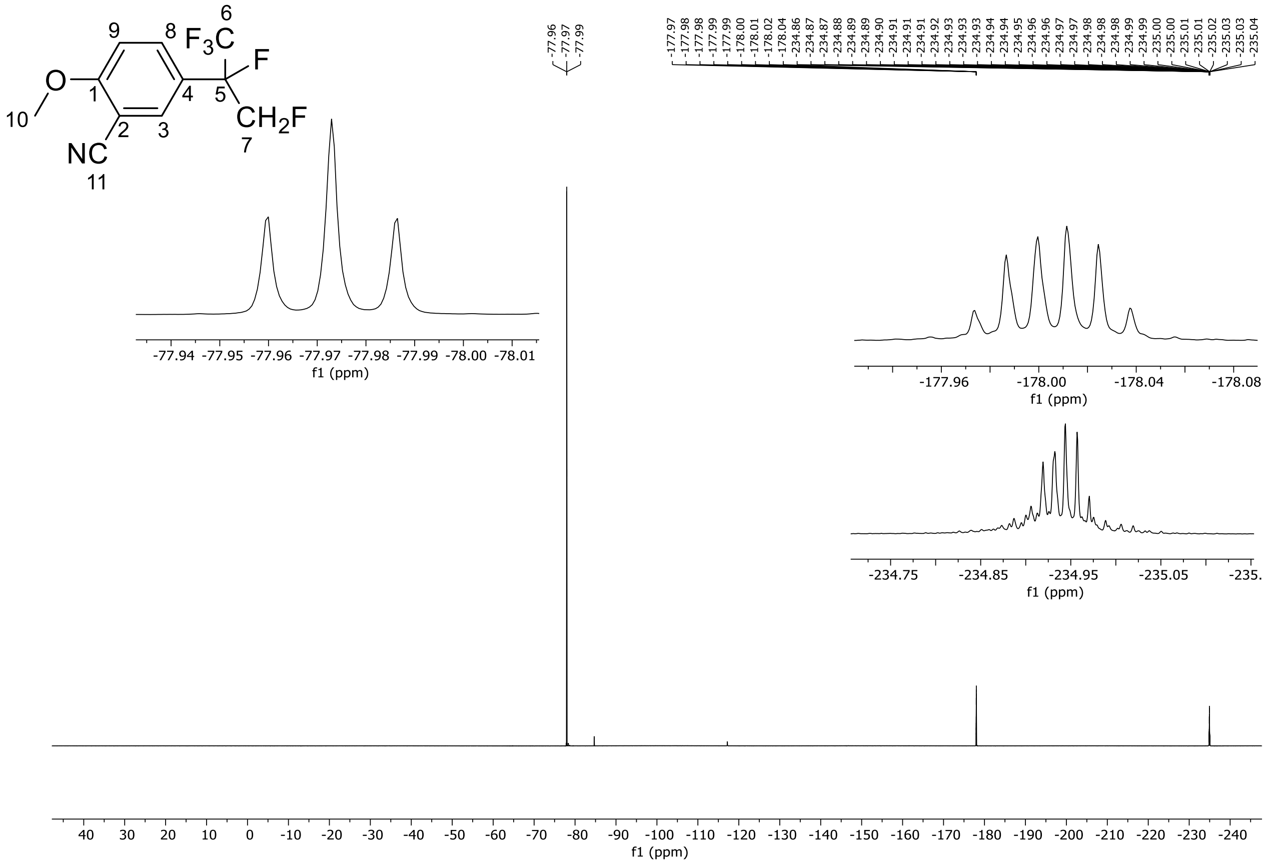

1-Bromo-4-(1,1,1,2,3-pentafluoropropan-2-yl)benzene (2l)

^{1}H **NMR** (500 MHz, $CDCl_3$)

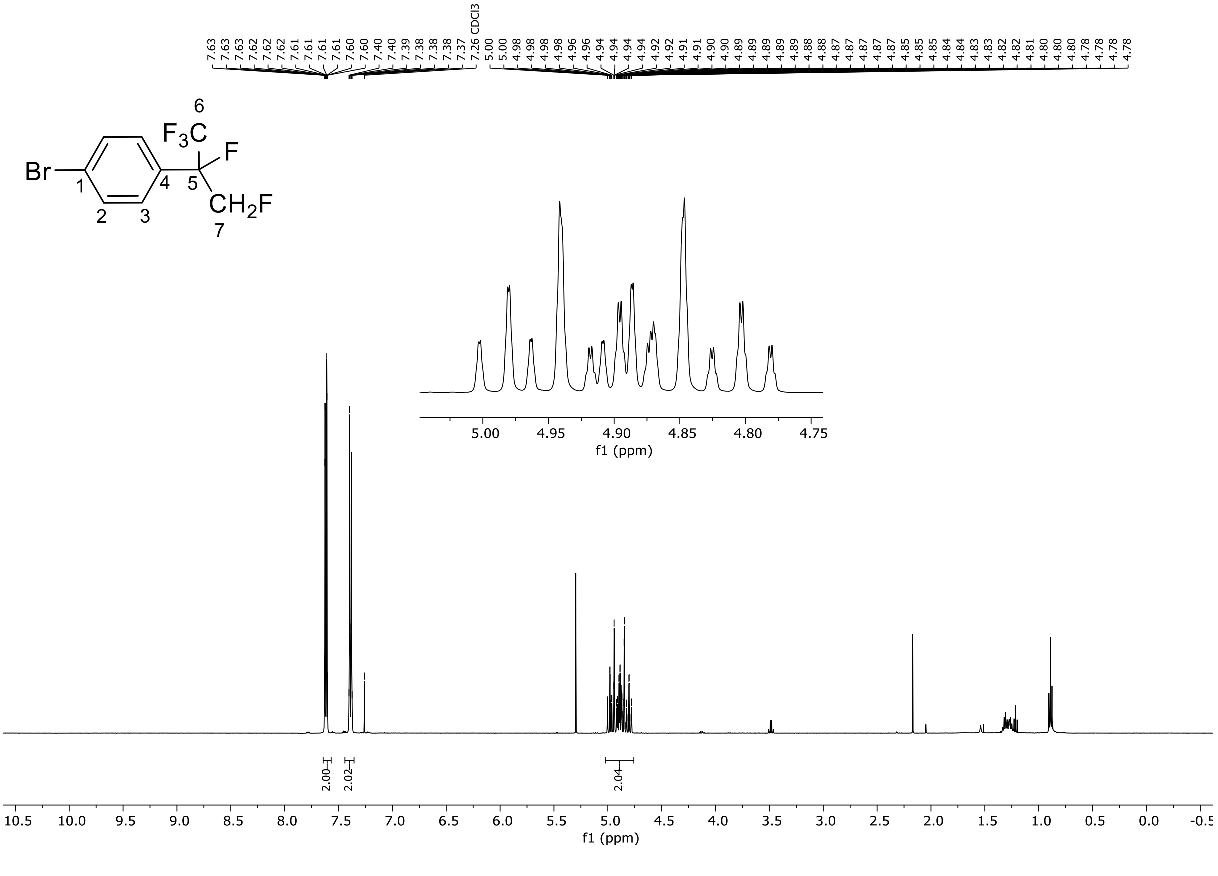

13C NMR (126 MHz, $CDCl_3$)

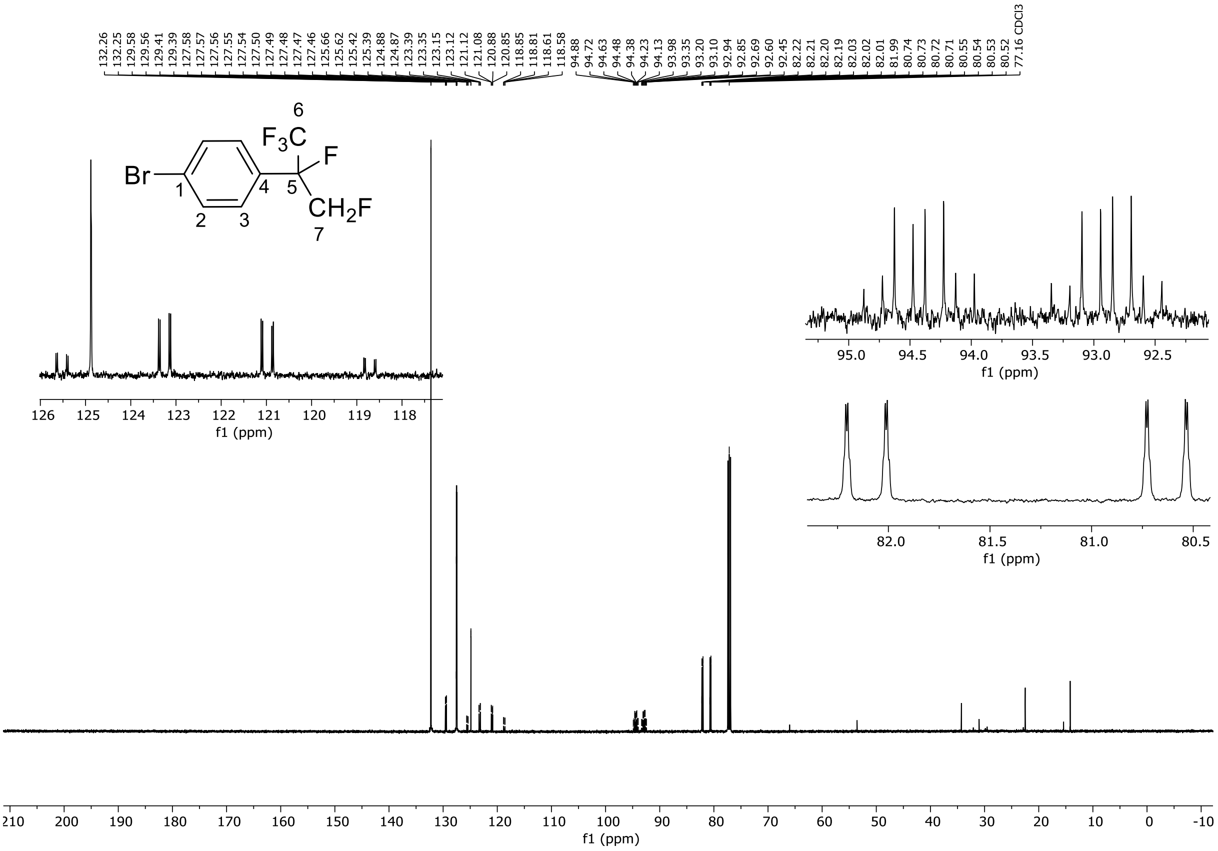

19F NMR (470 MHz, $CDCl_3$)

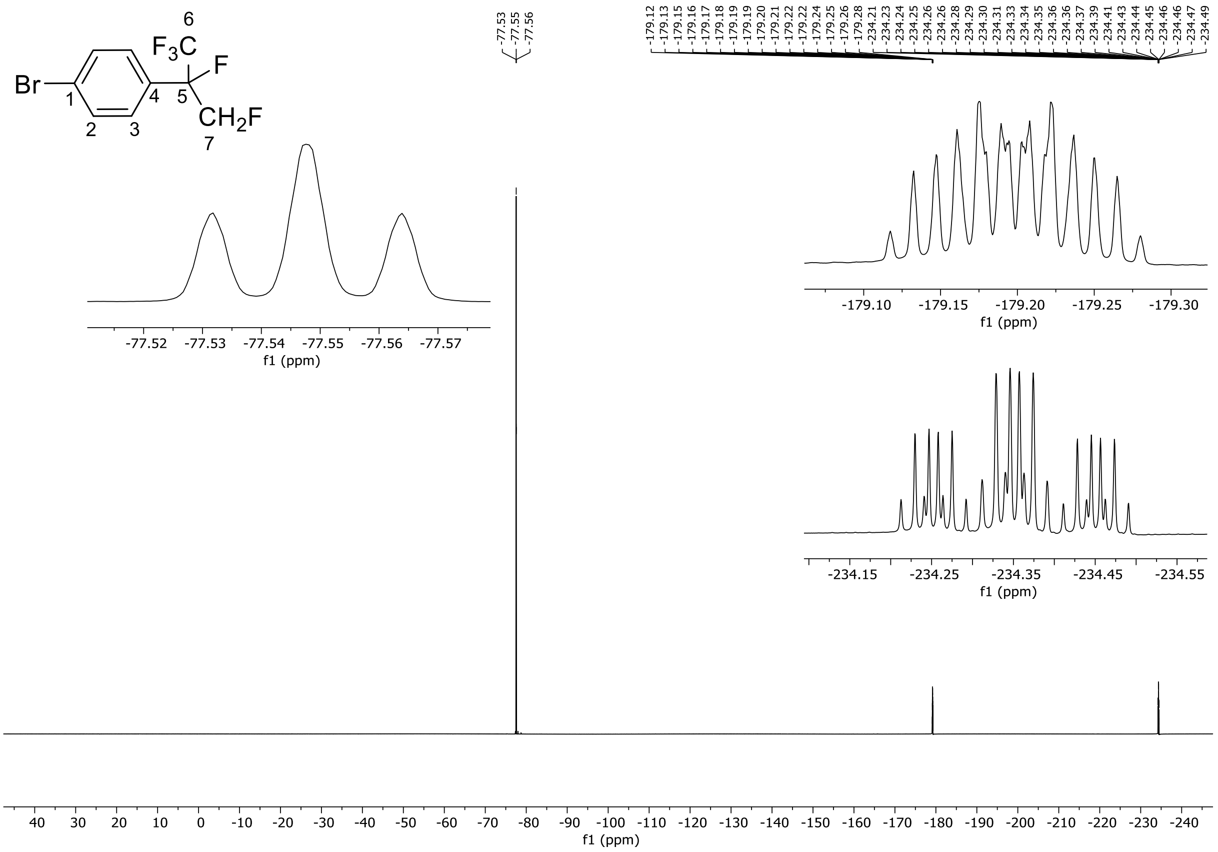

^{19}F{^{1}H} NMR (470 MHz, $CDCl_3$)

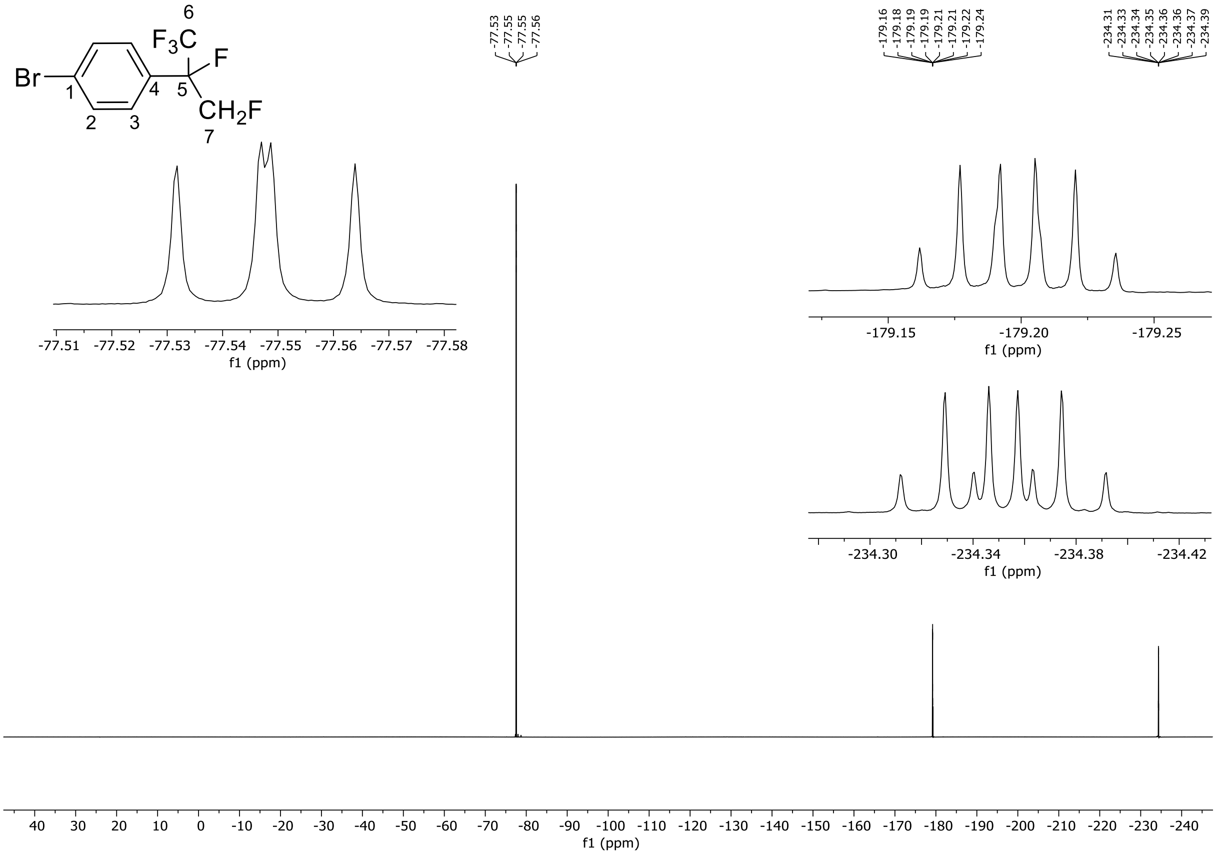

1-(Methoxymethyl)-4-(1,1,1,2,3-pentafluoropropan-2-yl)benzene (2m)

^{1}H NMR (599 MHz, $CDCl_3$)

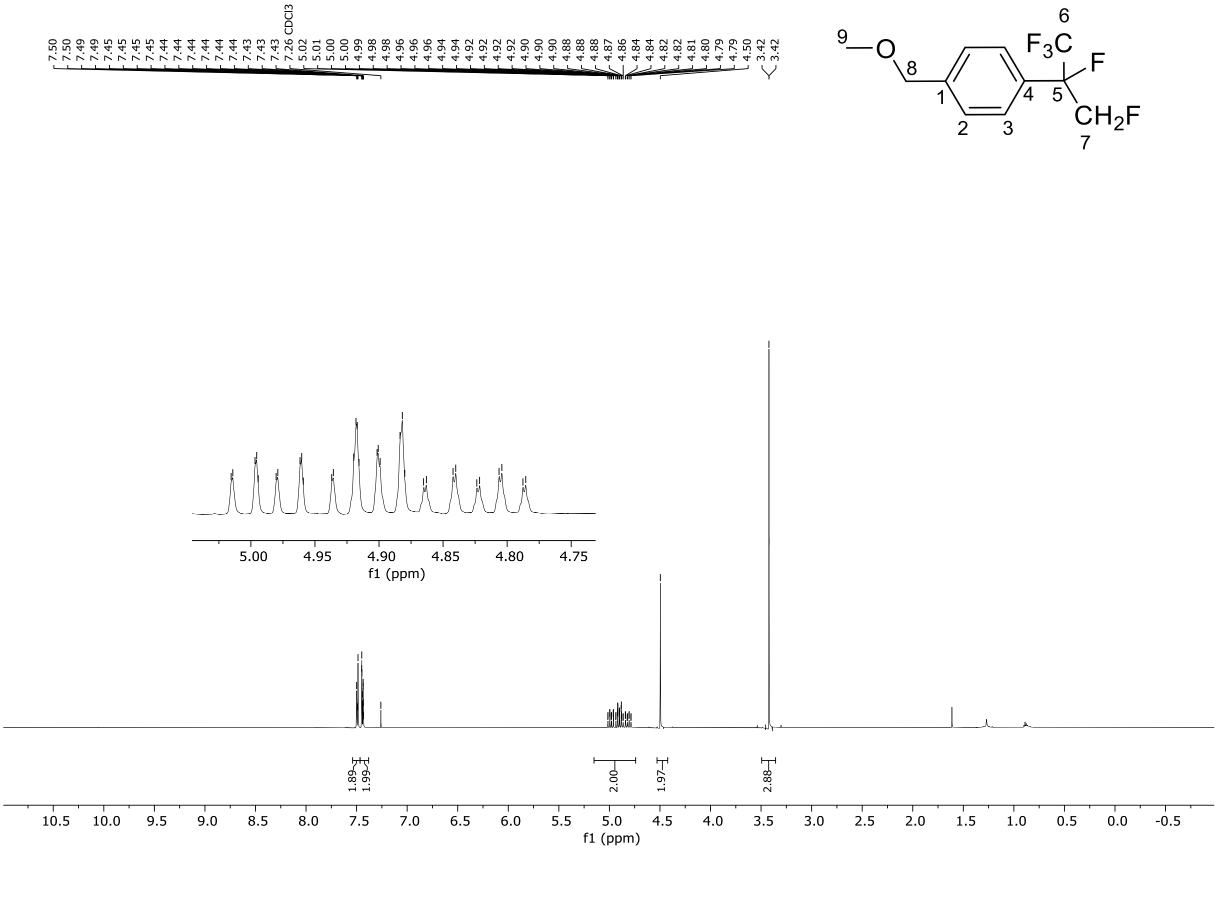

^{13}C NMR (151 MHz, $CDCl_3$)

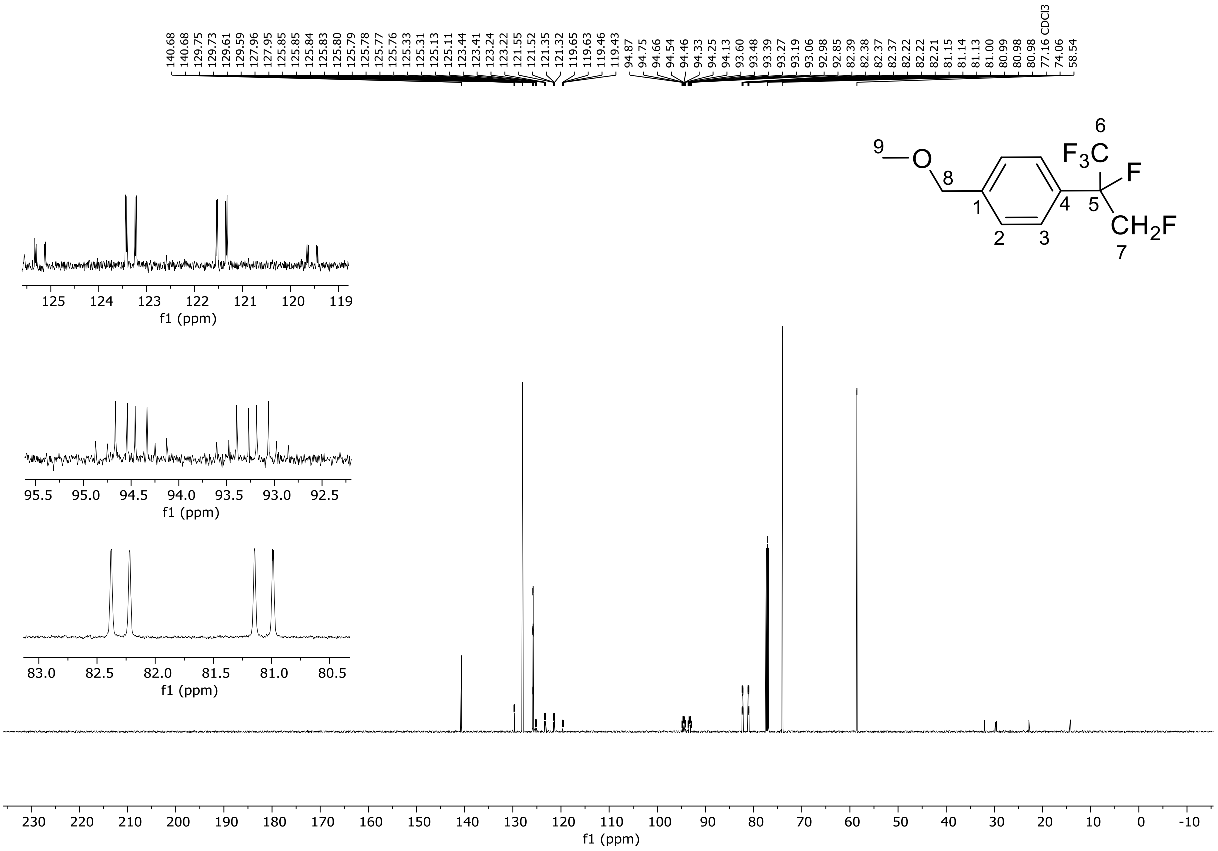

^{19}F NMR (376 MHz, $CDCl_3$)

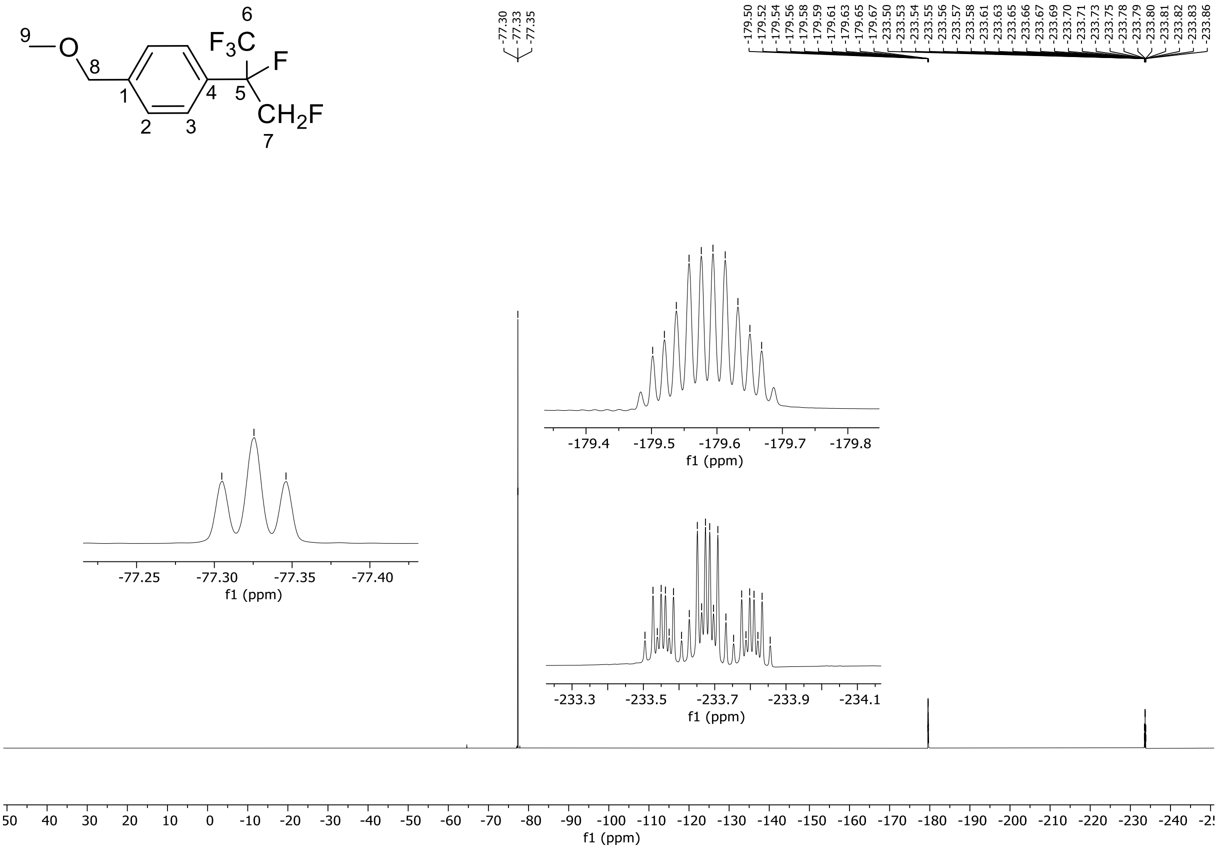

$^{19}F\{^1H\}$ NMR (376 MHz, $CDCl_3$)

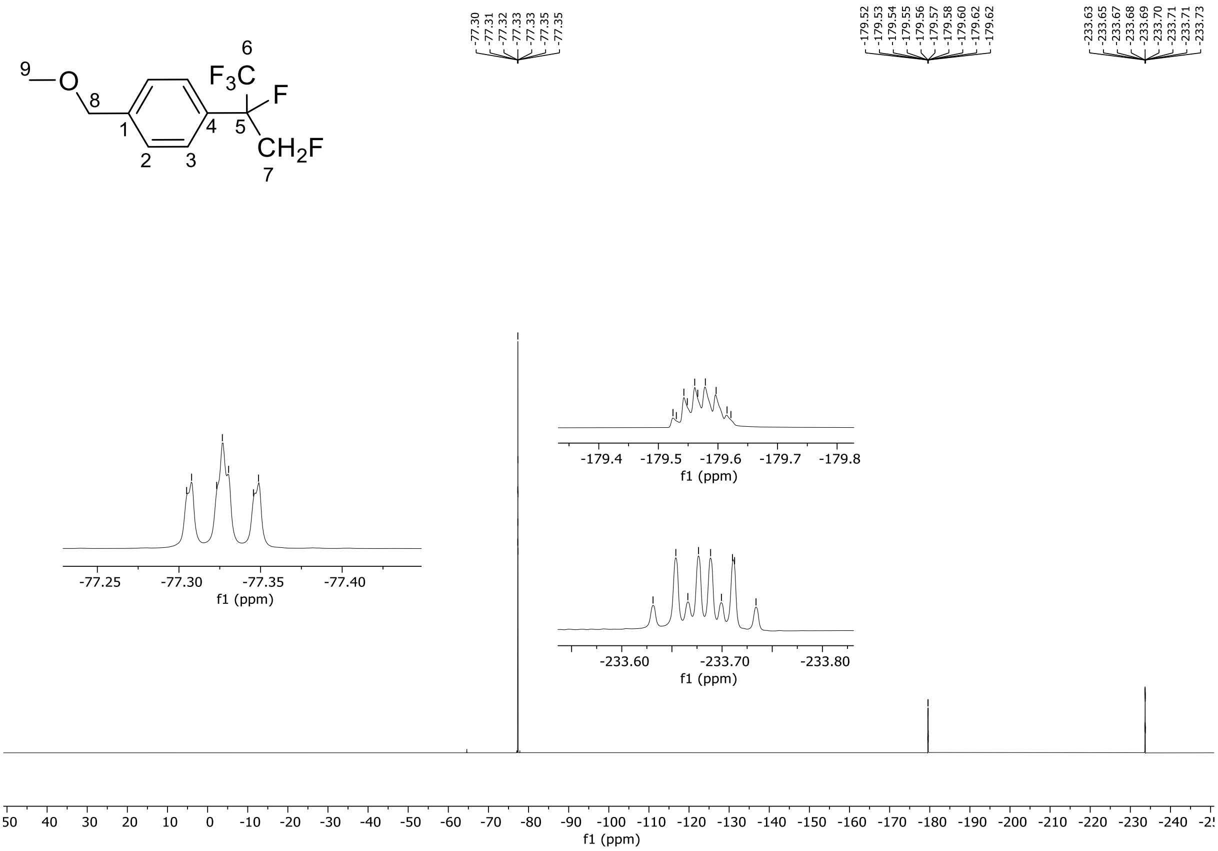

1-(1,1,1,2,3-Pentafluoropropan-2-yl)-4-(trifluoromethoxy)benzene (2n)

^1H NMR (599 MHz, $CDCl_3$)

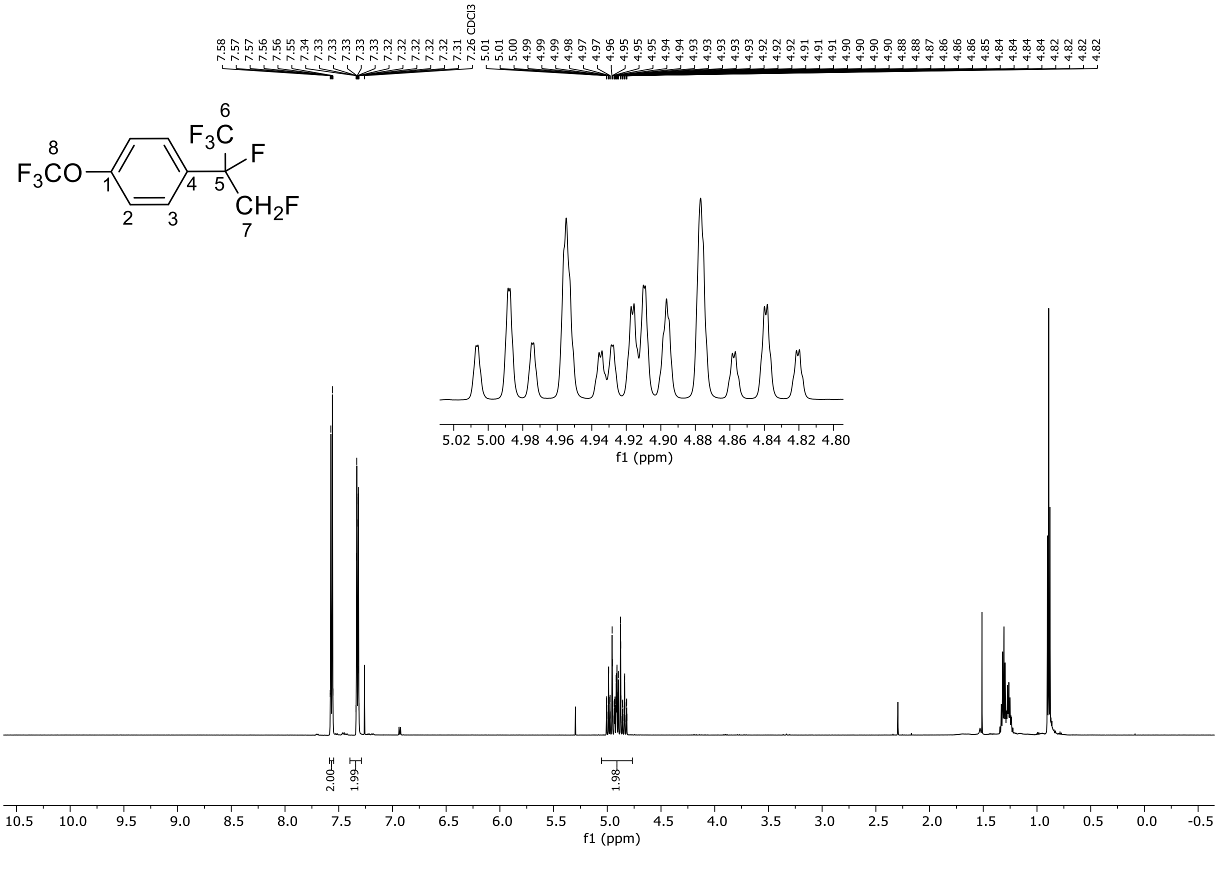

^{13}C NMR (151 MHz, $CDCl_3$)

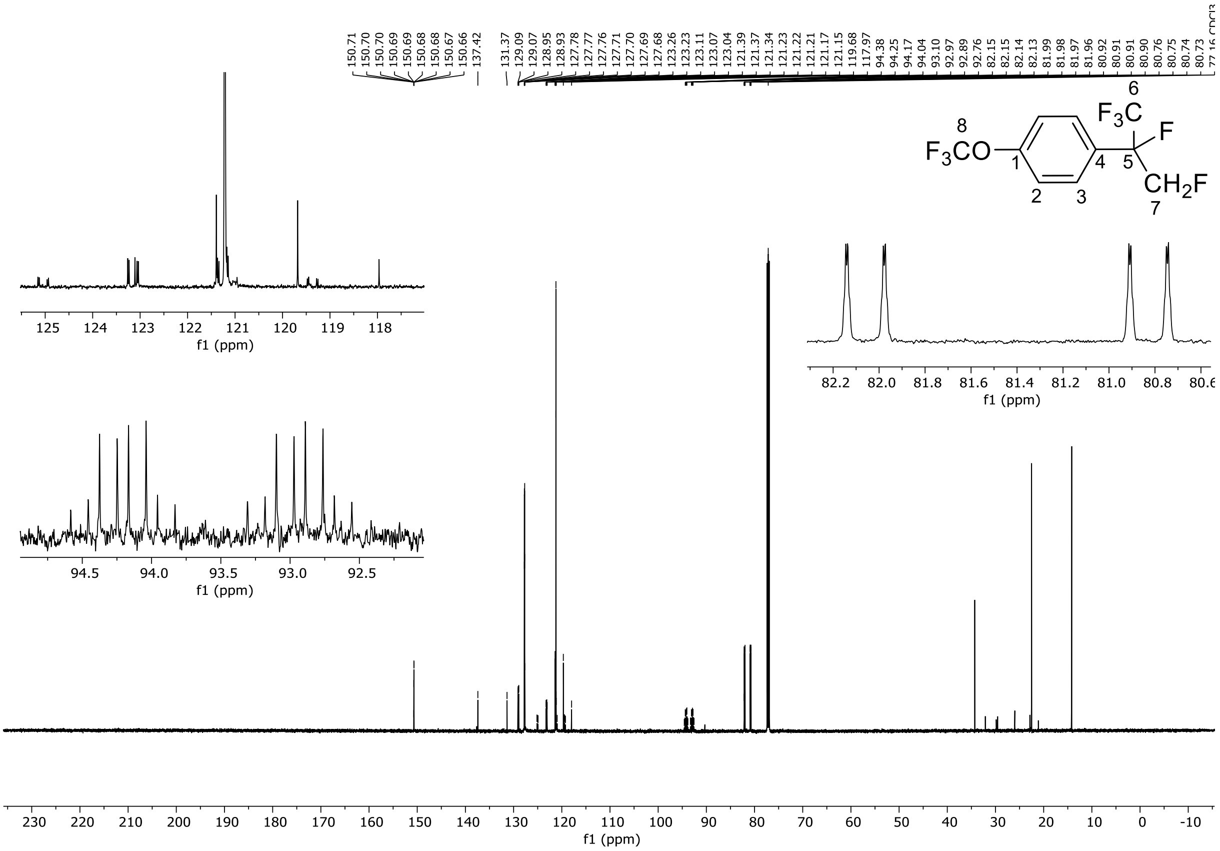

^{19}F NMR (564 MHz, $CDCl_3$)

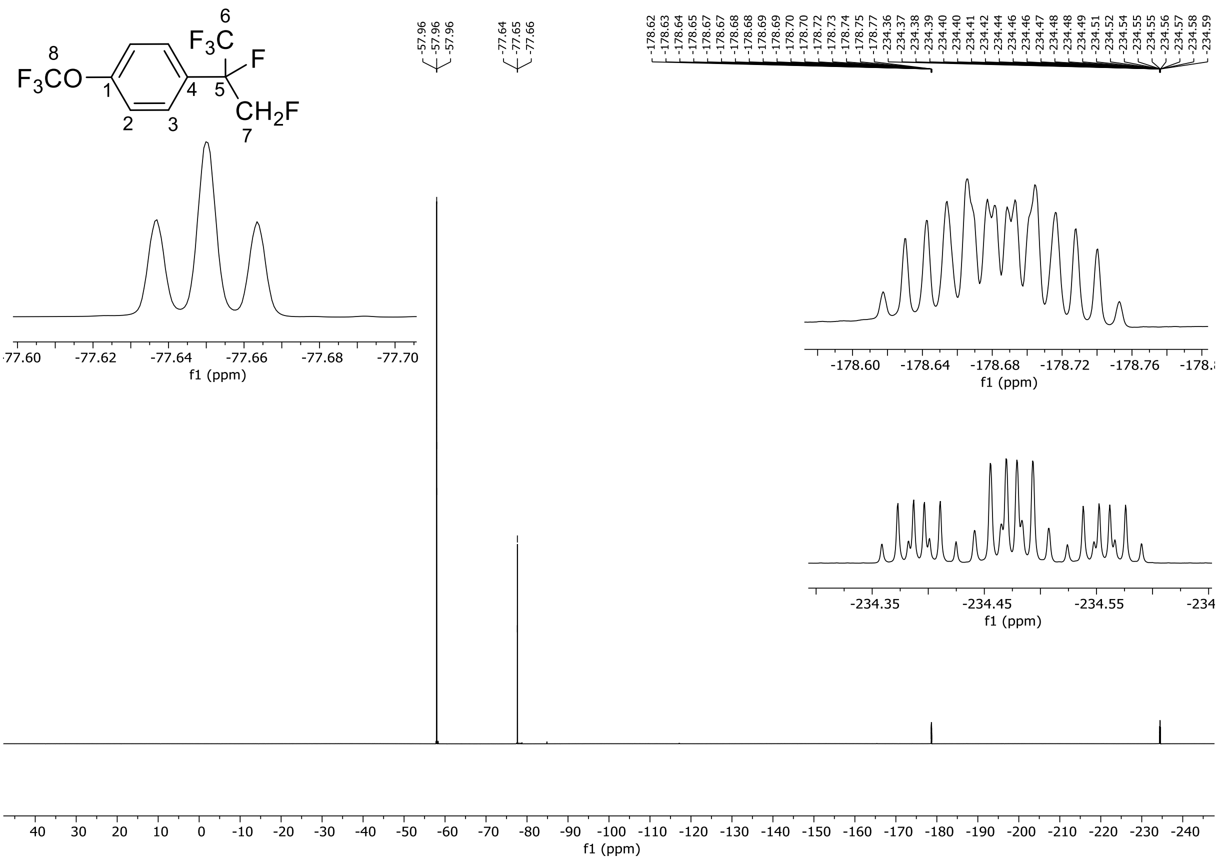

$^{19}F\{^{1}H\}$ NMR (564 MHz, $CDCl_3$)

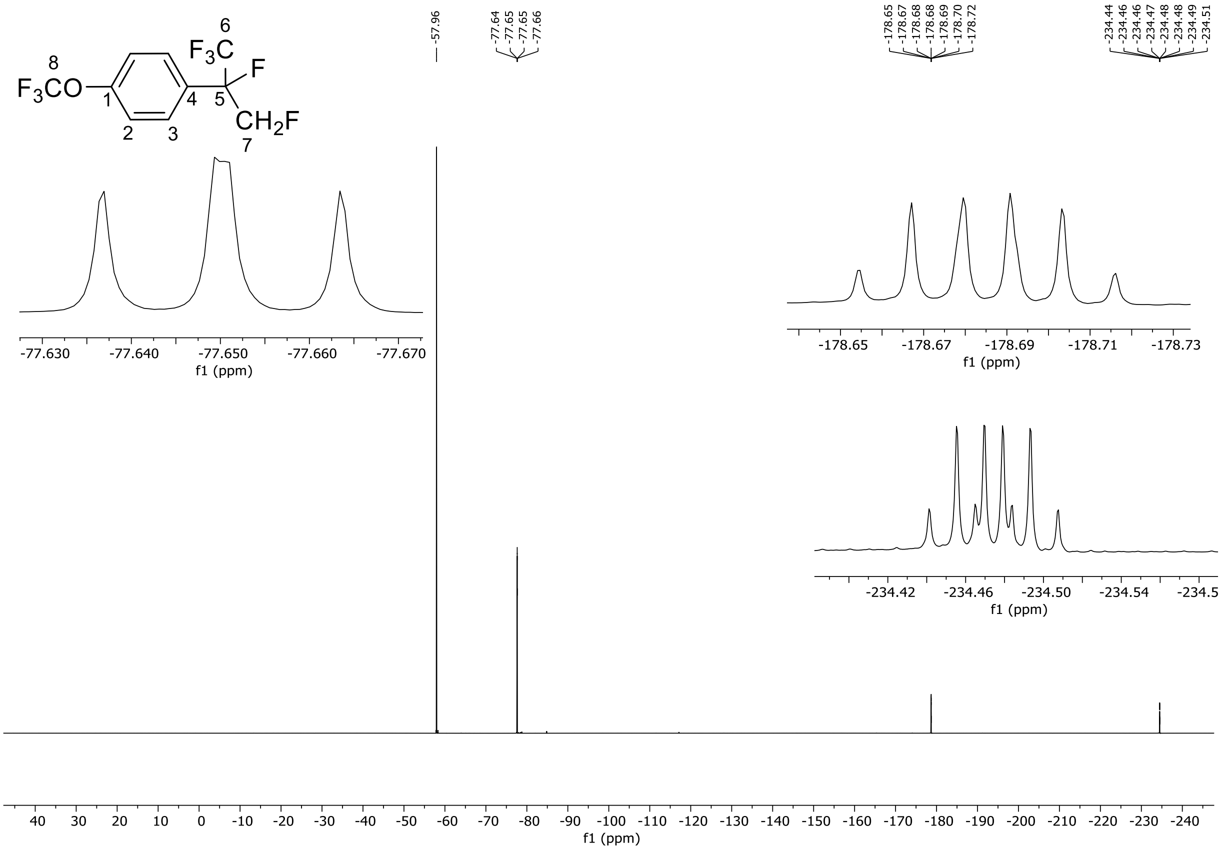

4-Nitro-4'-(1,1,1,2,3-pentafluoropropan-2-yl)-1,1'-biphenyl (2o)

^{1}H NMR (500 MHz, $CDCl_3$)

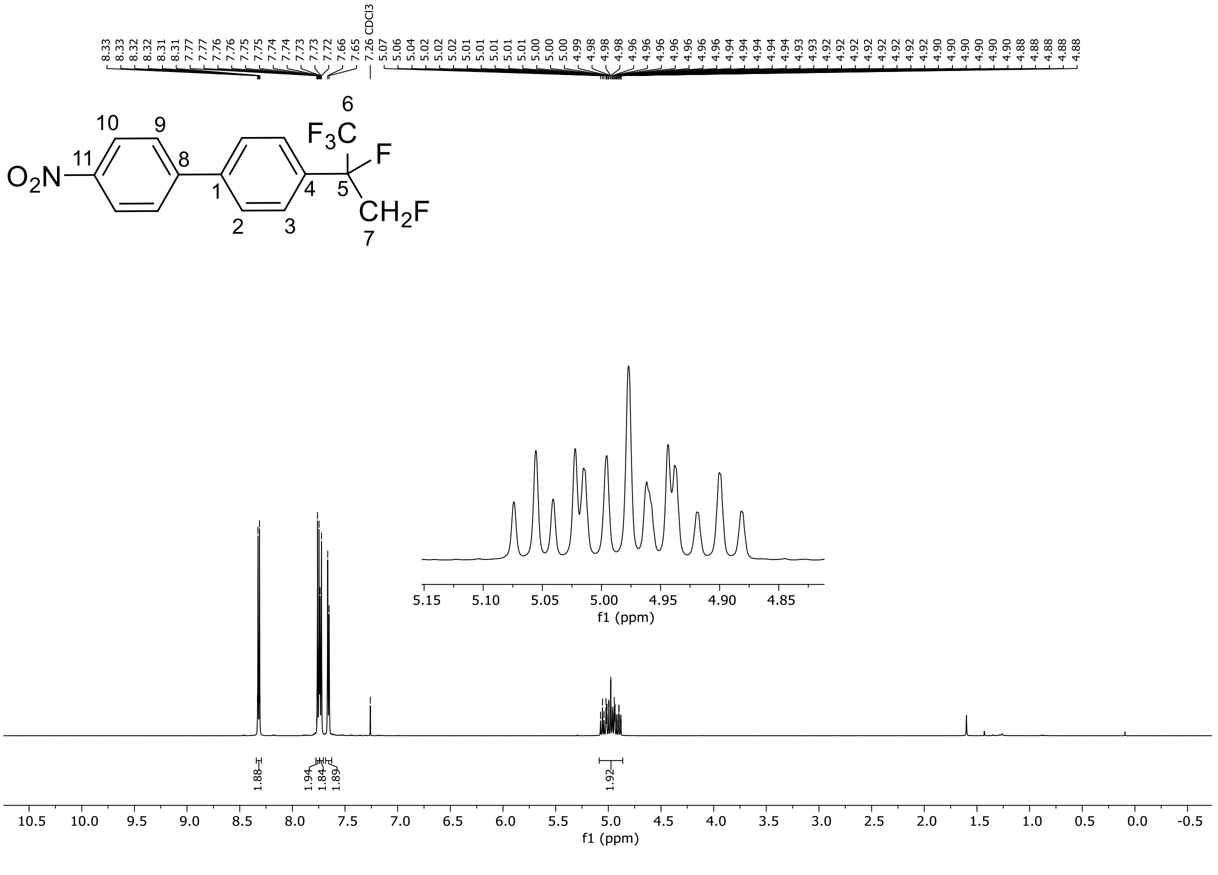

^{13}C NMR (126 MHz, $CDCl_3$)

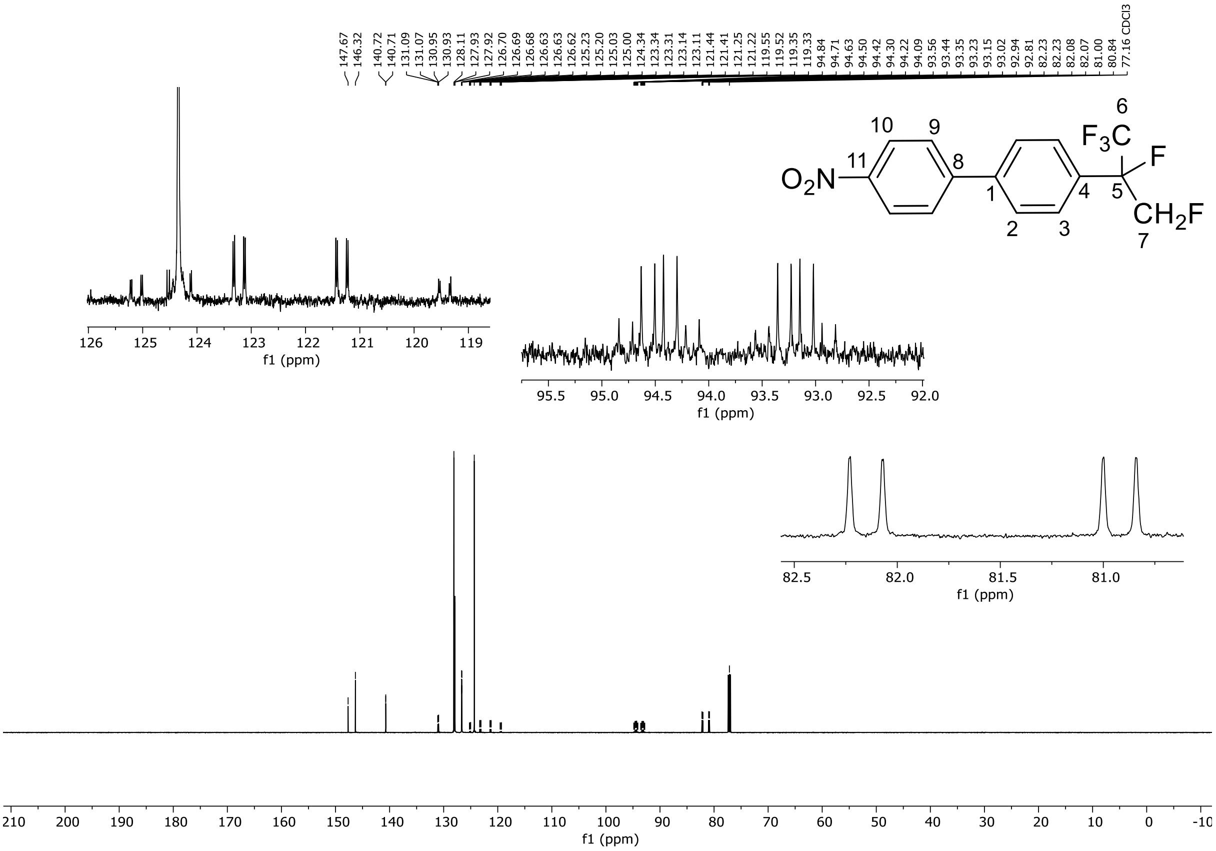

^{19}F NMR (126 MHz, $CDCl_3$)

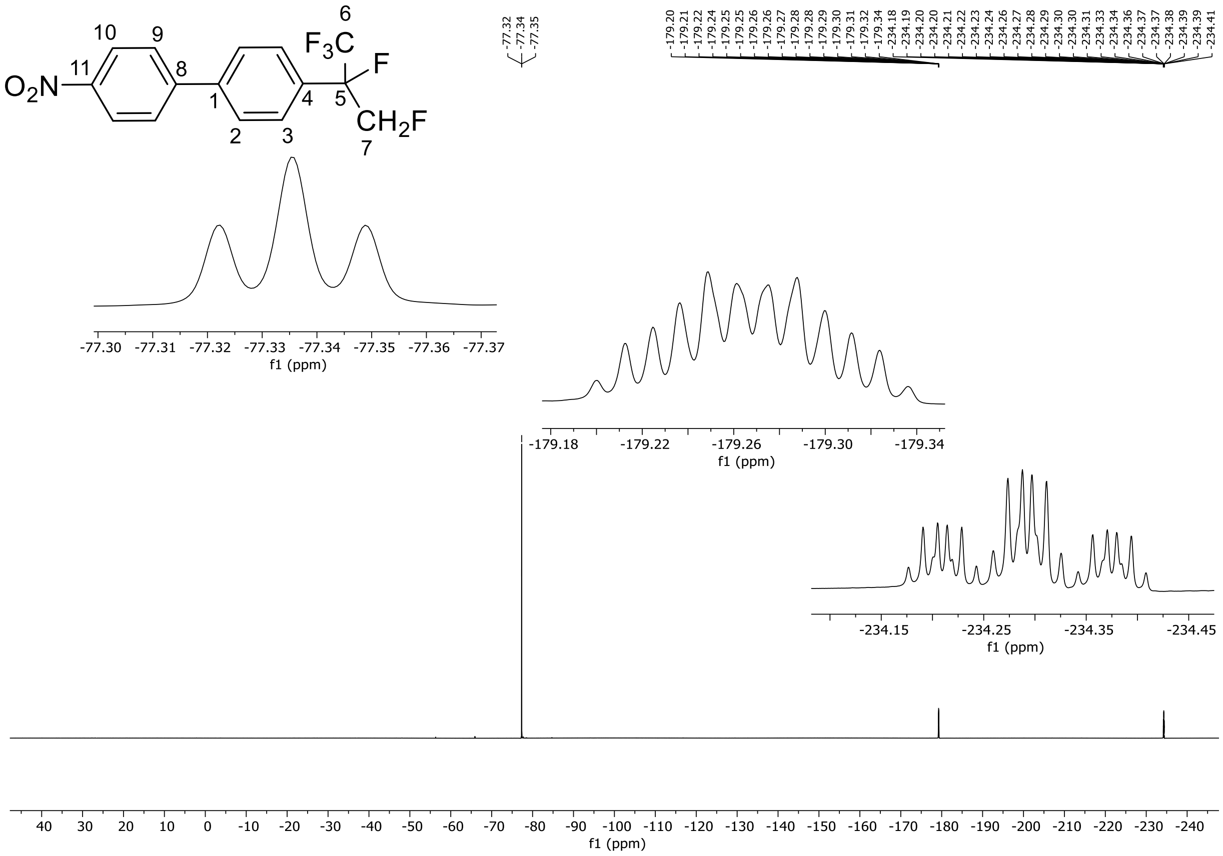

$^{19}F\{^{1}H\}$ NMR (126 MHz, $CDCl_3$)

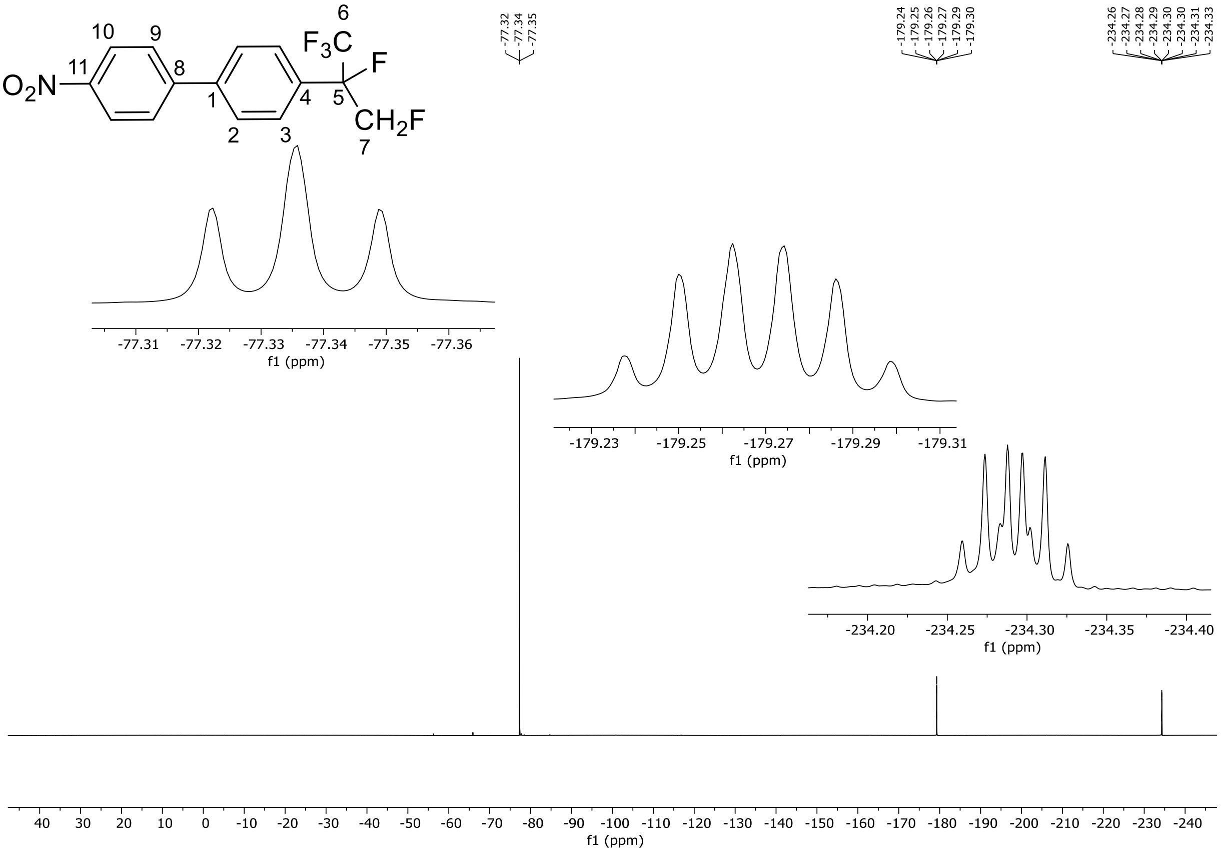

2-(4-(1,1,1,2,3-pentafluoropropan-2-yl)phenyl)isoindoline-1,3-dione (2p)

^{1}H NMR (500 MHz, $CDCl_3$)

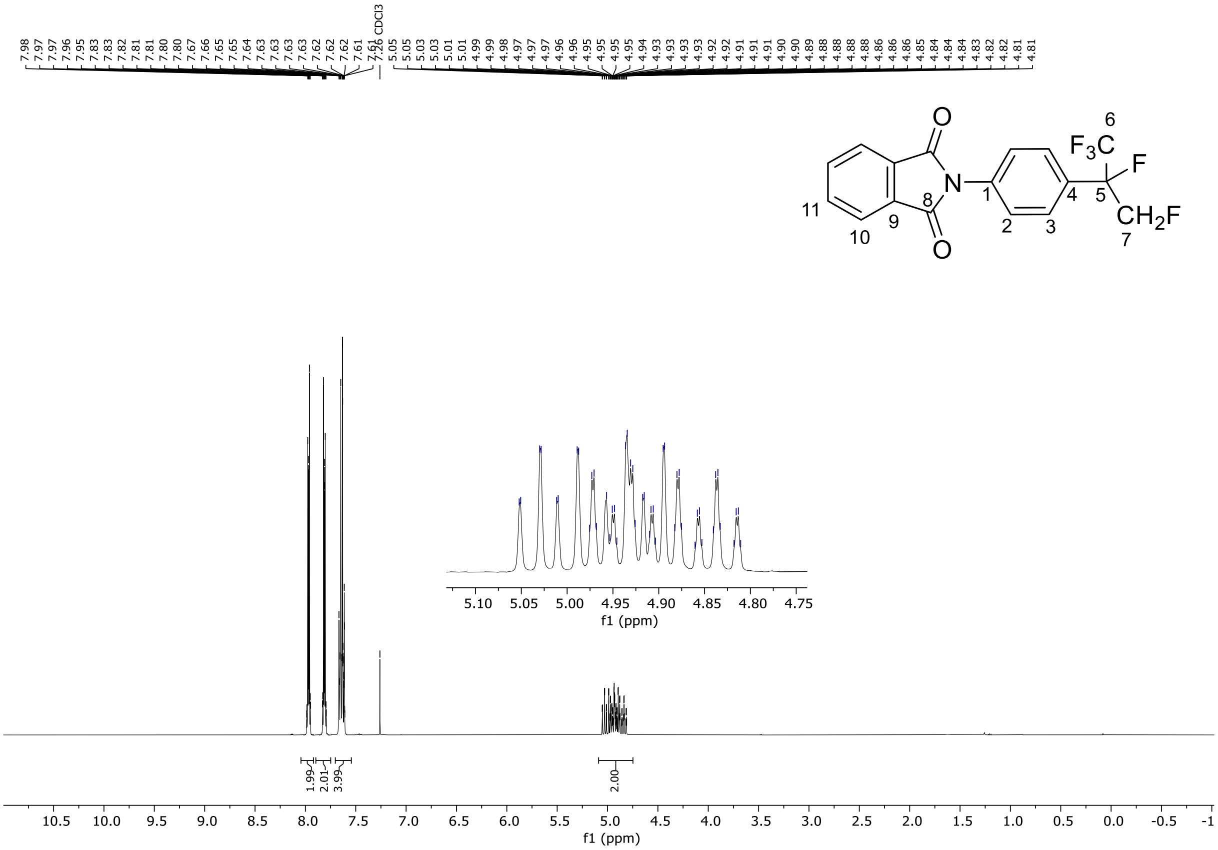

^{13}**C NMR** (126 MHz, $CDCl_3$)

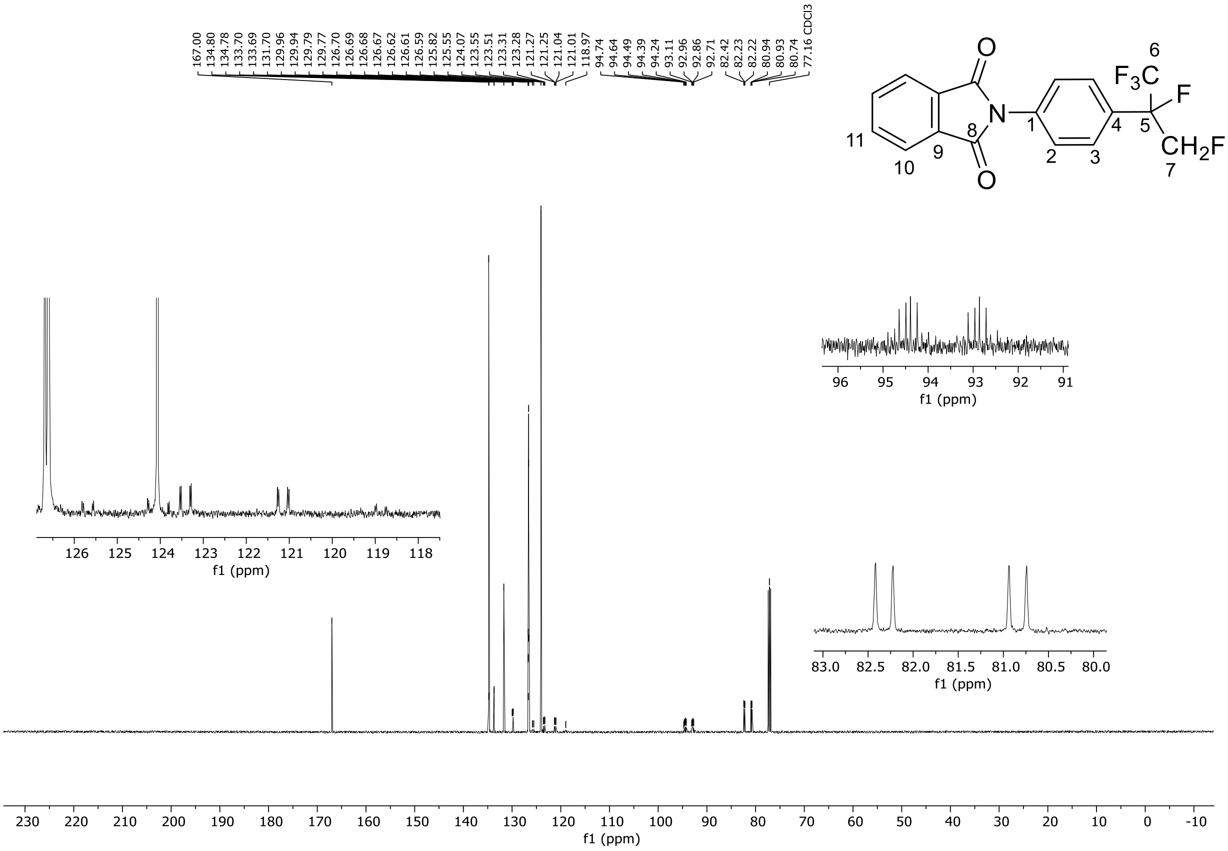

^{19}**F NMR** (126 MHz, $CDCl_3$)

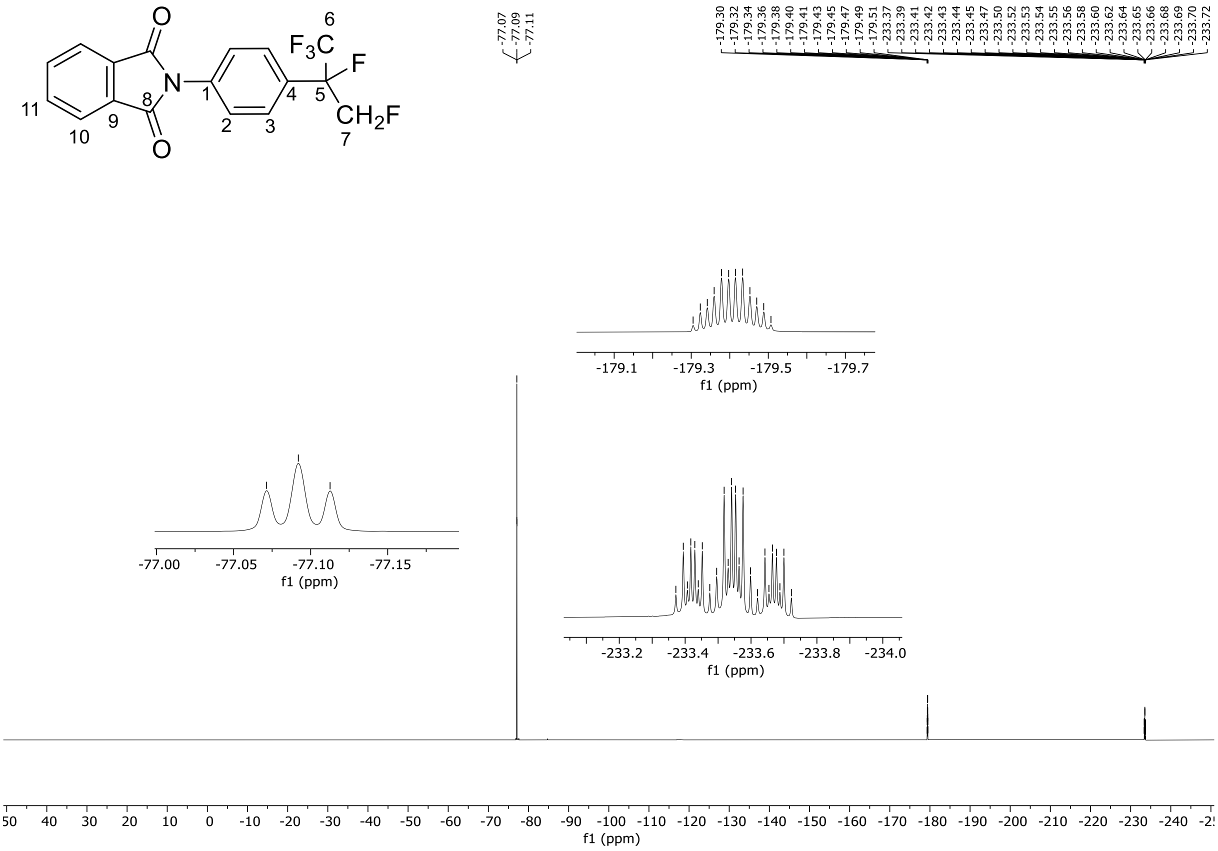

$^{19}F\{^1H\}$ NMR (126 MHz, $CDCl_3$)

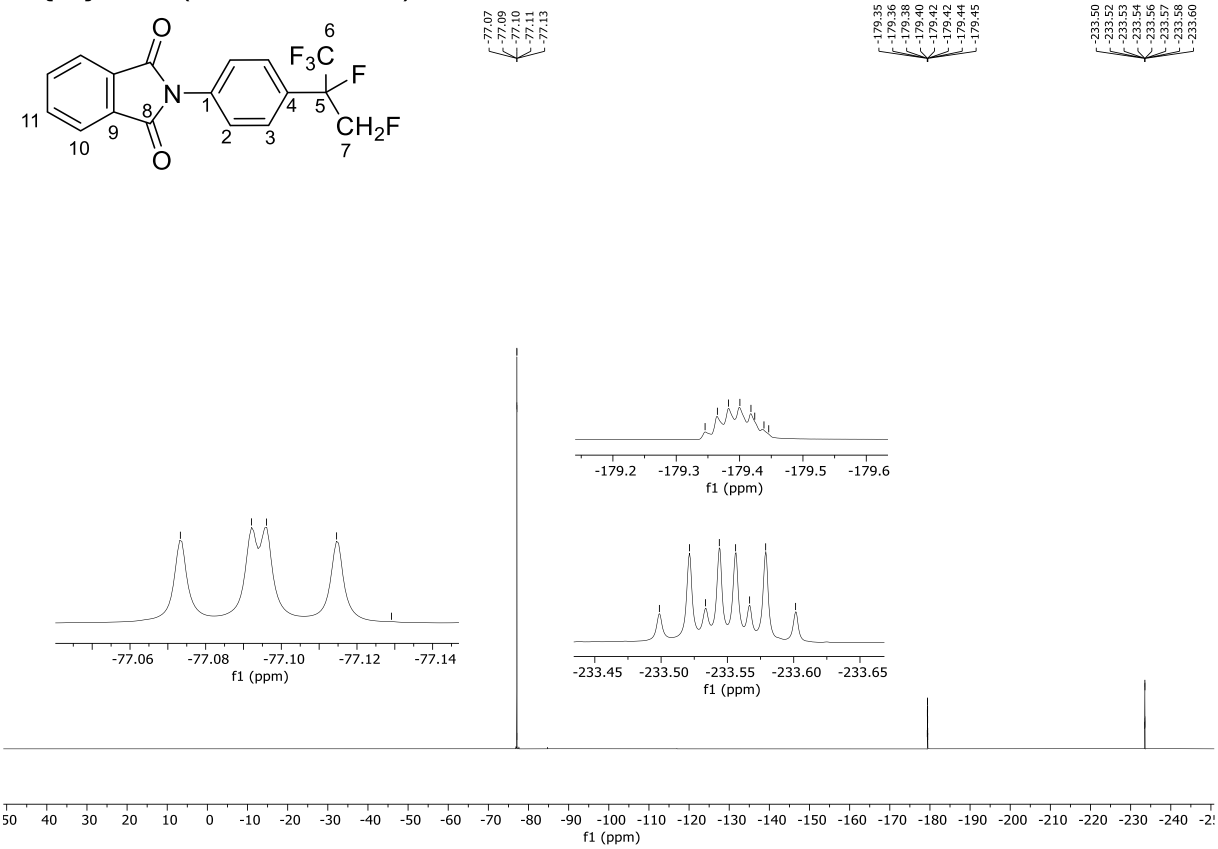

2-Methoxy-5-(1,1,1,2,3-pentafluoropropan-2-yl)phenyl trifluoromethanesulfonate (2q)

^1H NMR (500 MHz, $CDCl_3$)

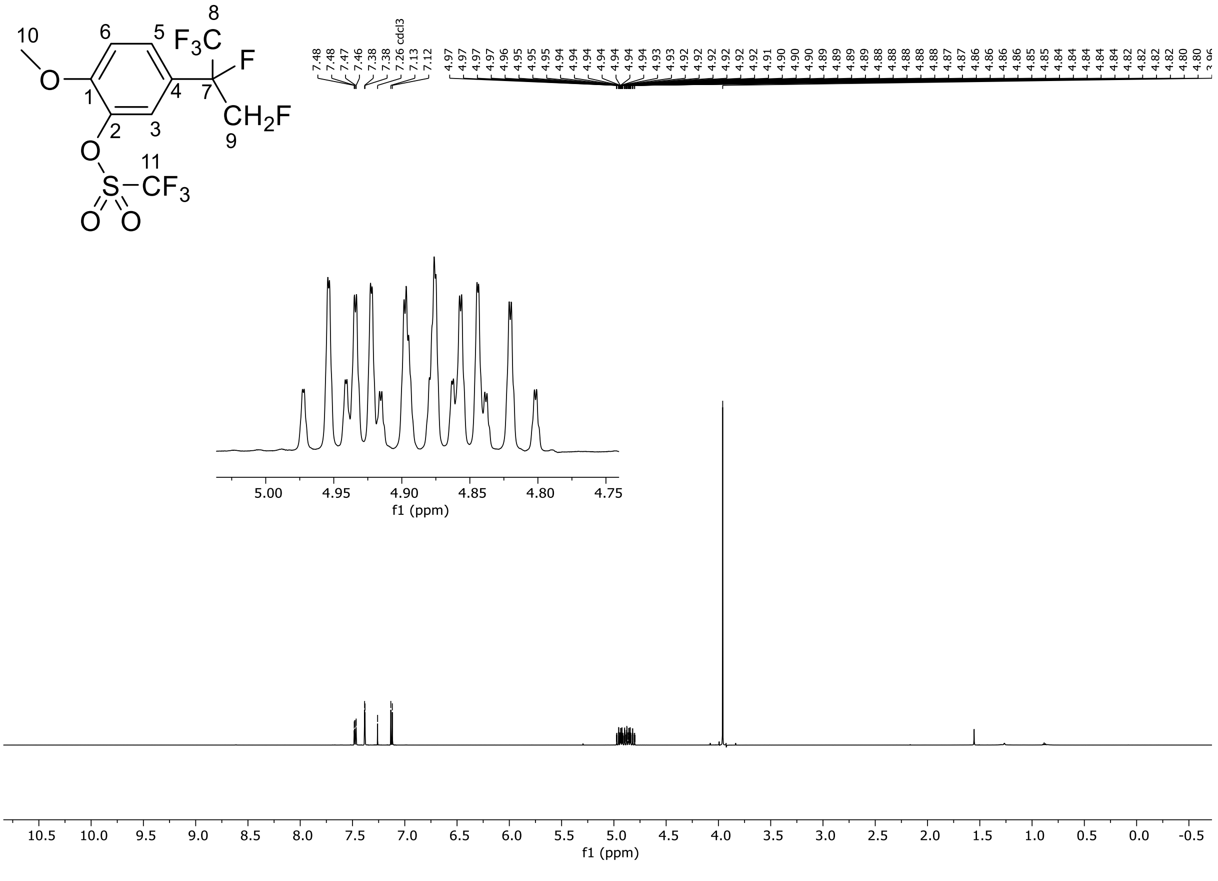

^{13}C NMR (126 MHz, $CDCl_3$)

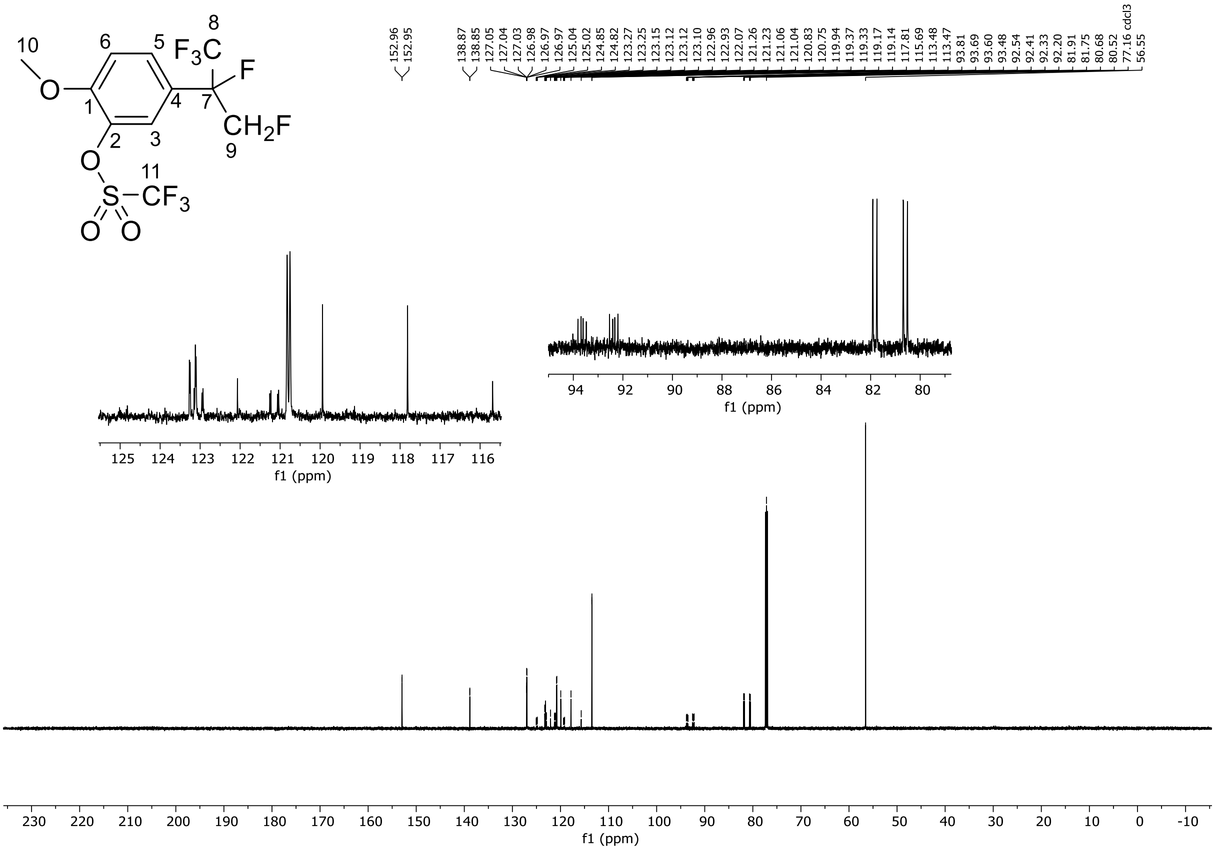

^{19}F NMR (126 MHz, $CDCl_3$)

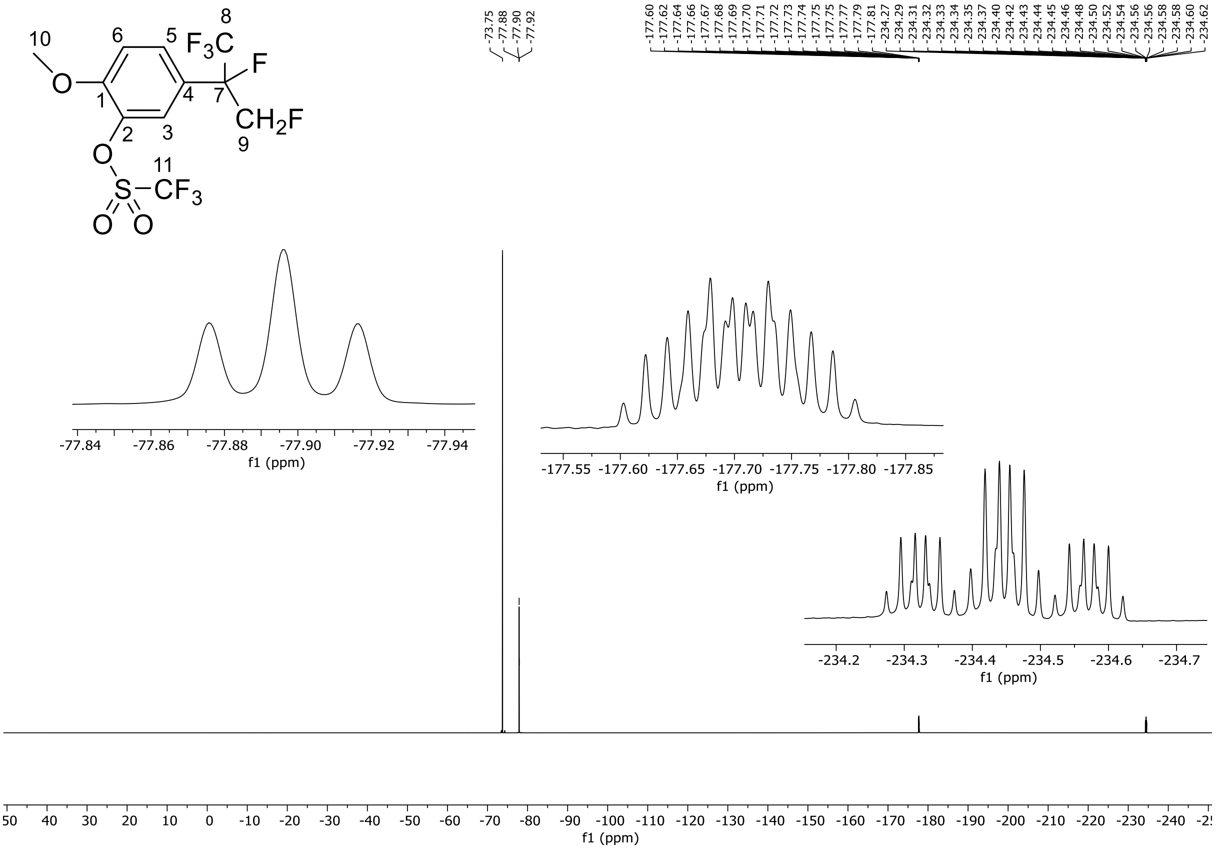

$^{19}F\{^{1}H\}$ NMR (126 MHz, $CDCl_3$)

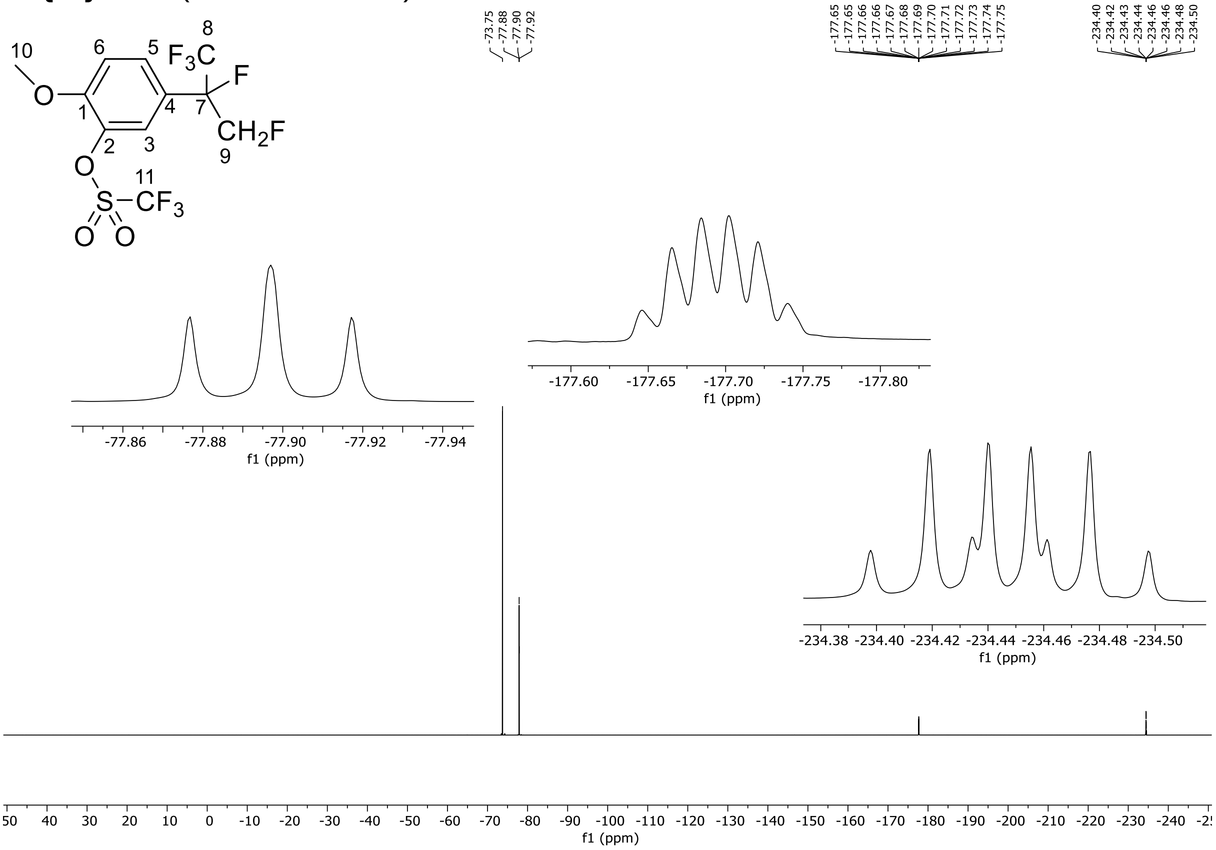

2-(1,3-Dimethyl-2,6-dioxo-1,2,3,6-tetrahydro-7H-purin-7-yl)-N-(4-(1,1,1,2,3-pentafluoropropan-2-yl)phenyl)acetamide (2r)

^{1}H NMR (500 MHz, $CDCl_3$)

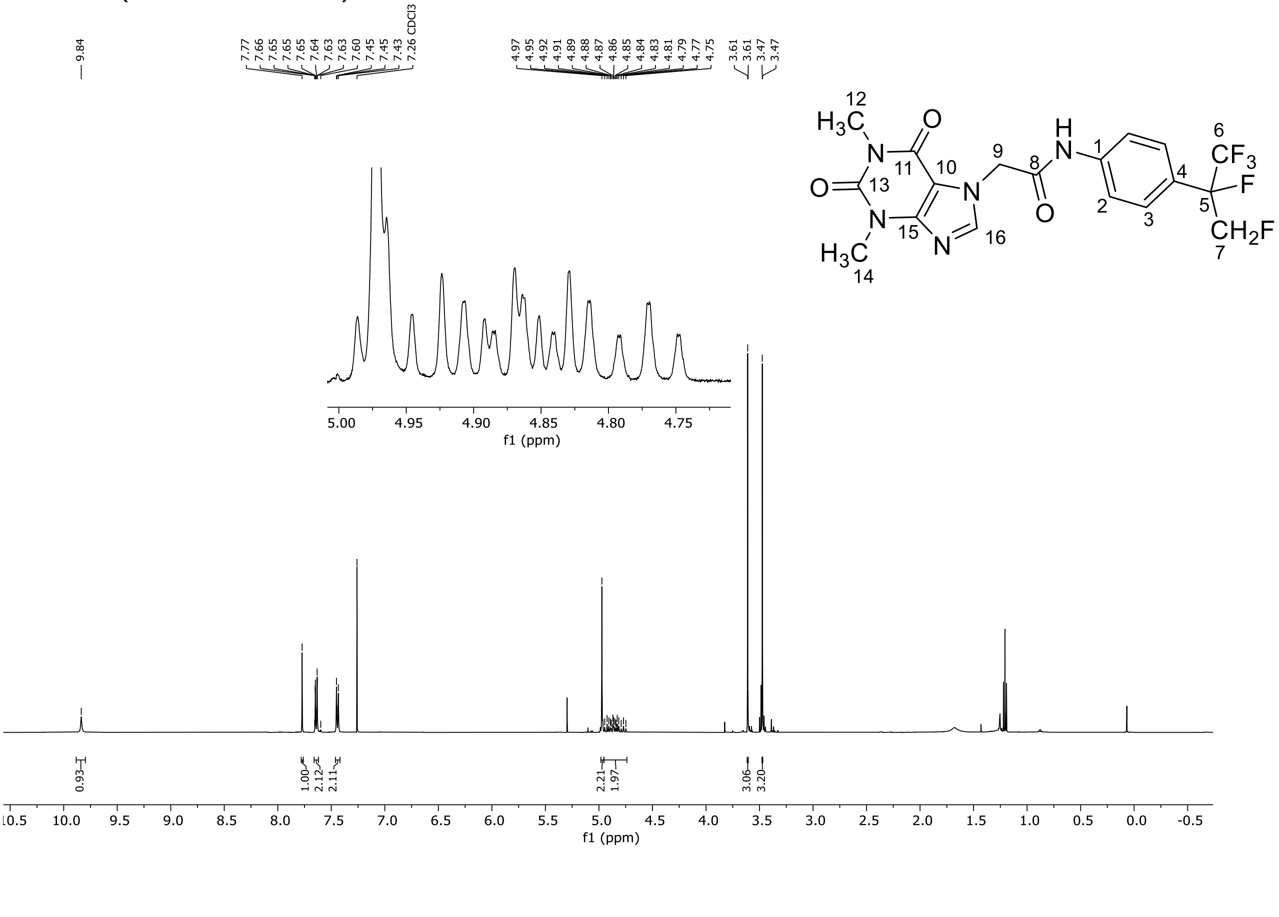

^{13}C NMR (126 MHz, $CDCl_3$)

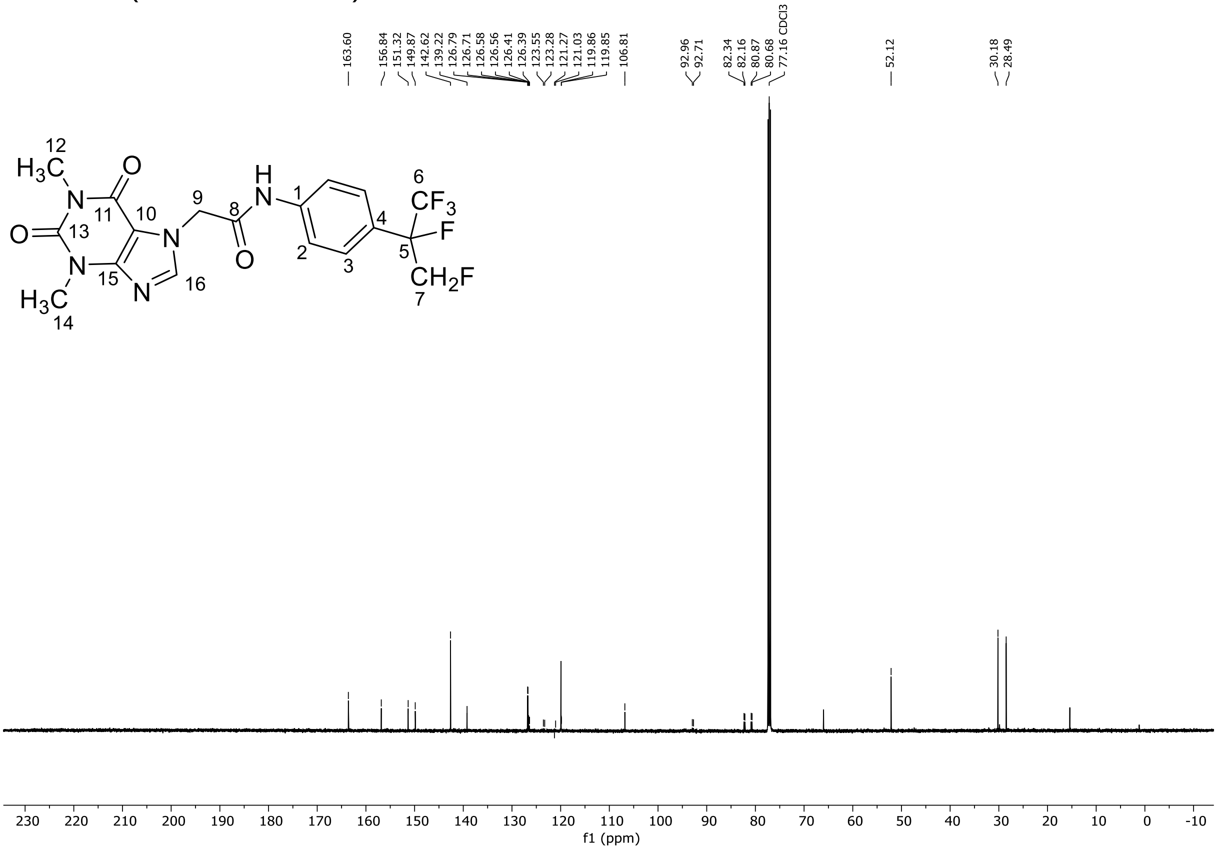

^{19}F NMR (470 MHz, $CDCl_3$)

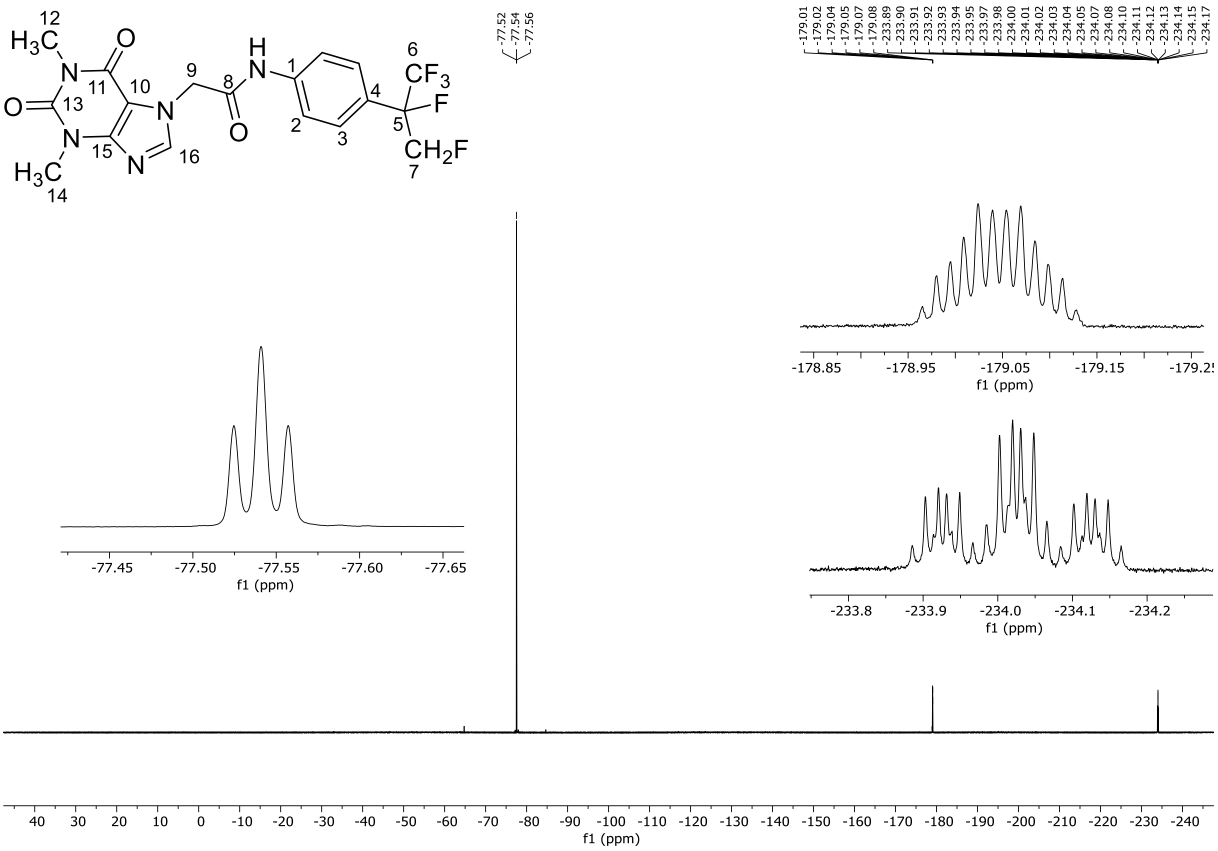

$^{19}F\{^1H\}$ NMR (126 MHz, $CDCl_3$)

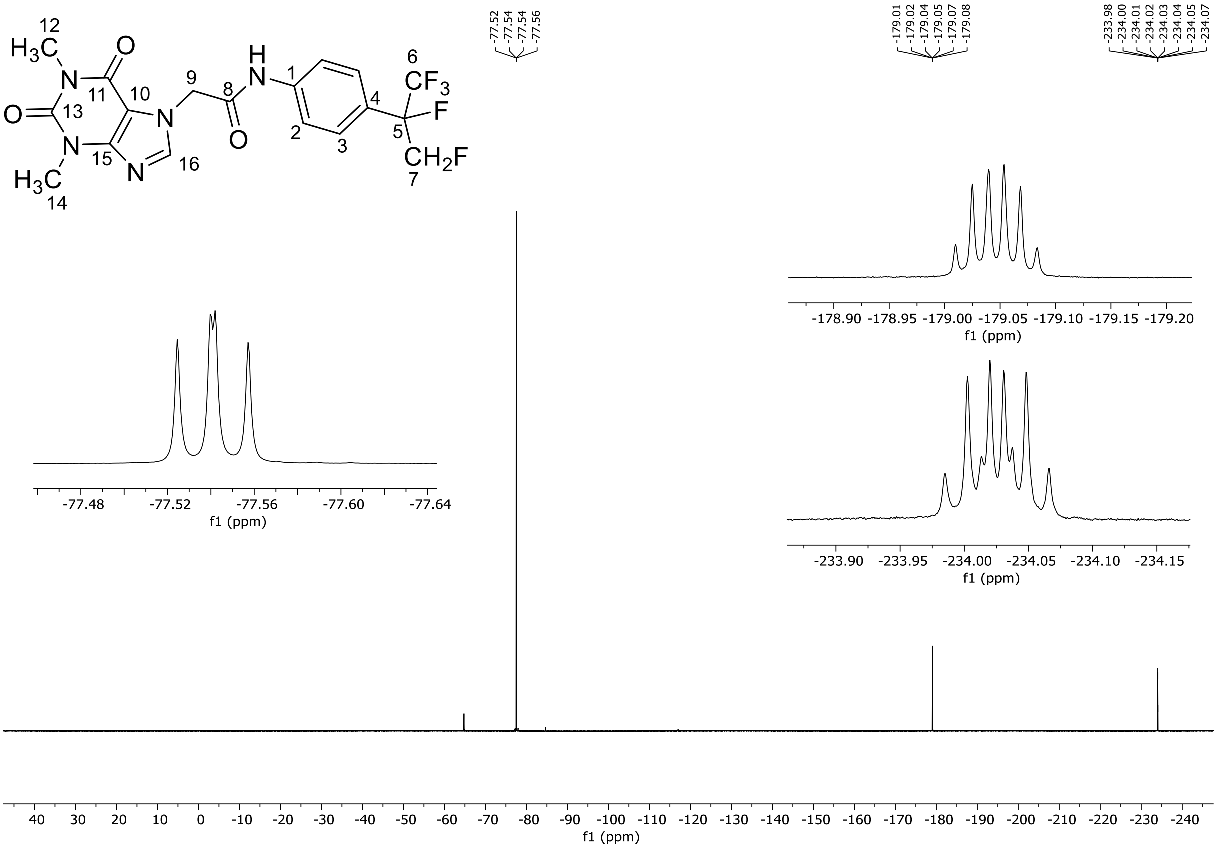

5. References

[1] F. Scheidt, M. Schäfer, J. C. Sarie, C.G. Daniliuc, J. J. Molloy, R. Gilmour *Angew. Chem. Int. Ed*. **2018**, *57*, 16431-16435.

[2] Y. Liu, Y. Zhou, Y. Zhao, J. Qu *Org. Lett.* **2017**, *19*, 946-949.

[3] O. Cussó, X. Ribas, J. Lloret-Fillol, M. Costas, *Angew. Chem. Int. Ed*. **2015**, *54*, 2729-2733.

[4] Y-Q. Guo, R. Wang, H. Song, Y. Liu, Q. Wang*, Org. Lett*. **2020**, *22*, 709-713.

[5] K. Terashima, T. Kawasaki-Takasuka, T. Agou, T. Kubota, T. Yamazaki *Chem. Commun*. **2020**, *56*, 3031.

[6] P. B. Huleatt, M. L. Khoo, Y. Y. Chua, T. W. Tan, R. S. Liew, B. Balogh, R. Deme, F. Gölöncser, K. Magyar, D. Sheela, H. K. Ho, B. Sperlagh, P. Matyus, C. L. L. Chai *J. Med. Chem*. **2015**, *58*, 1400-1419.

[7] Y. Li, B. Zhao, K. Dai, D.-H. Tu, B. Wang, Y.-Y. Wang, Z.-T. Liu, Z.-W. Liu, J. Lu*, Tetrahedron* **2016**, *72*, 5684-5690.

[8] Y. Lan, F. Yang, C. Wang *ACS Catal*. **2018**, *8*, 9245-9251.

[9] T. Fujita, N. Konno, W. Yota, T. Ichitsuka, A. Nagaki, J. Yoshida, J. Ichikawa, *J. Fluor. Chem*. **2018**, *207*, 72-76.

[10] J. P. Phelan, S. B. Lang, J. S. Compton, C. B. Kelly, R. Dykstra, O. Gutierrez. G. A. Molander, *J. Am. Chem. Soc*. **2018**, *140*, 8037-8047.

[11] S. M. Banik, J. W. Medley, E. N. Jacobsen, *Science* **2016**, *353*, 51–54.

[12] S. Haubenreisser, T.H. Wöste, C. Martínez, K. Ishihara, K. Muñiz, *Angew. Chem. Int. Ed*. **2016**, *55*, 413-417.

[13] M.A. Berliner, E.M. Cordi, J. R. Dunetz, K. E. Price, *Org. Process Res. Dev***.** **2010**,*14*,180-187.

[14] Y. Kobayashi, M. Takase, Y. Ito, S. Terashima, *S. Bull. Chem. Soc. Jpn*. **1989**, *62*, 3038-3040.

[15] C. Röben, J.A. Souto, Y. Gonzáles, A. Lishchynskyi, K. Muñiz, *Angew. Chem. Int. Ed*. **2011**, *50*, 9478-9482.

[16] S. Banik, J.W. Medley, E. Jacobsen, *J. Am. Chem. Soc*. **2016**, *138*, 5000-5003.

[17] W.-C. Gao, Z.-Y. Xiong, S. Pirhaghani, T. Wirth, *Synthesis* **2019**, *51*, 276-284.

[18] J.C. Sarie, C. Thiehoff, J. Neufeld, C.G. Daniliuc, R. Gilmour *Angew. Chem. Int. Ed*. **2020**, *59*, 15069-15075.
